# Supplementary material for: Self-assembled conjoined-cages
Source: Nat Commun. 2020 Feb 14;11:880. doi: 10.1038/s41467-020-14703-4 (PMC7021905; doi:10.1038/s41467-020-14703-4)
Supplement: Supplementary file 1 — Supplementary Information [file 41467_2020_14703_MOESM1_ESM.pdf]

Supplementary Information

Self-assembled conjoined-cages

Samantray et al.

Supplementary Methods.

Materials and Instruments

$PdCl_2$, PdI_2, $Pd(NO_3)_2$, AgCl, $AgBF_4$, AgOTf, $AgClO_4$, $AgSbF_6$, $AgPF_6$, tetra-n-butylammonium halides (fluoride, chloride and bromide), tetra-n-butylammonium nitrate, nicotinoylchloride hydrochloride and 3-pyridylcarbinol were obtained from Sigma-Aldrich whereas N-ethyl-N′-(3-dimethylaminopropyl)carbodiimide hydrochloride (EDC·HCl), $AgNO_3$, nicotinic acid, 4-dimethyl aminopyridine, pyridine3,5-dicarboxylic acid, resorcinol and common solvents were obtained from Spectrochem, India. The ligand **L1** was synthesised as reported previously.[1] The deuterated solvent DMSO-d_6 was obtained from Sigma-Aldrich. Nuclear Magnetic Resonance (NMR) spectra were recorded in DMSO-d_6 at room temperature (r.t.) on Bruker AV400 and AV500 spectrometers at 400 and 500 MHz for ^{1}H NMR, COSY, NOESY and at 100 and 125 MHz for ^{13}C NMR. Chemical shifts are reported in parts per million (ppm) relative to residual solvent protons (2.50 ppm for DMSO-d_6 in ^{1}H NMR and 39.50 in ^{13}C NMR). The ESI mass spectra were recorded on Agilent Q-TOF and Micromass Q-TOF spectrometers. Single crystal X-ray diffraction analysis was carried out using a Bruker D8 VENTURE instrument.

Synthesis of the Ligands and the Complexes

Synthesis of the ligand L2

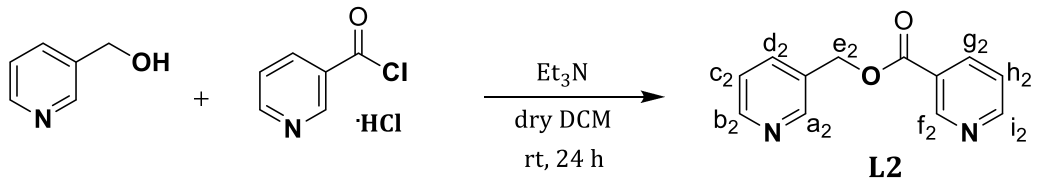

Pyridin-3-ylmethyl nicotinate, L2

Triethylamine (0.24 mL, 1.685 mmol) was added in a dropwise manner to a stirred suspension of nicotinoyl chloride hydrochloride (0.300 g, 1.685 mmol) and 3-pyridylcarbinol (0.184 g, 0.16 mL, 1.685 mmol) in dry dichloromethane (30 mL) maintained at 0-5 °C. The mixture was stirred at room temperature for 24 h under nitrogen atmosphere. In order to neutralise the acid, $NaHCO_3$ solution (10% *w/v*) was added slowly to the mixture until the evolution of CO_2 has ceased. The organic layer was washed with distilled water, separated and dried over anhydrous

sodium sulfate. Purification of the crude product by column chromatography (ethyl acetate:hexane 4:6) yielded a colourless liquid, **L2** (0.288 g, yield 80%).

TLC (Ethyl acetate:Hexane, 40:60 v/v): R_f = 0.4; ^{1}H NMR (500 MHz, DMSO-d_6, room temperature): δ 9.12 (d, J = 1.3 Hz, 1H, H_{f2}), 8.82 (dd, J_1 = 4.8 Hz, J_2 = 1.6 Hz, 1H, H_{i2}), 8.72 (d, J = 1.4 Hz, 1H, H_{a2}), 8.57 (m, 1H, H_{b2}), 8.32 (m, 1H, H_{g2}), 7.93 (d, J = 7.8 Hz, 1H, H_{d2}), 7.57 (m, 1H, H_{h2}), 7.43 (m, 1H, H_{c2}), 5.43 (s, 2H, H_{e2}); ^{13}C NMR (125 MHz, DMSO-d_6, room temperature): δ 164.6, 153.8, 150.1, 149.5, 149.4, 137.0, 136.1, 131.5, 125.4, 124.0, 123.7, 64.3; HRMS (ESI, CH_2Cl_2/CH_3OH): *m/z* Calcd. for $C_{12}H_{10}N_2O_2$: 214.2200, found 215.0820 $[M+H]^+$.

Synthesis of the complex $[NO_3\subset Pd_2(L2)_4](NO_3)_3$, 2a

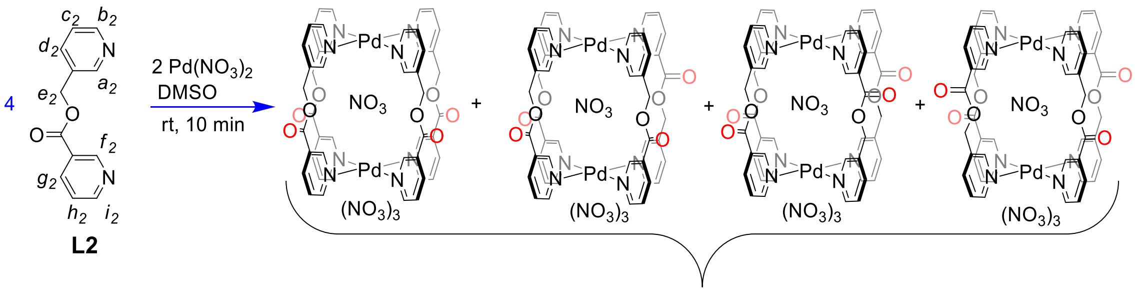

2a (Diastereomers)

The ligand **L2** (12.85 mg, 0.059 mmol) was added to a solution of $Pd(NO_3)_2$ (6.91 mg, 0.029 mmol) in 3 mL of DMSO. The reaction mixture was stirred at room temperature for 10 min. Subsequent, addition of 10 mL of ethyl acetate to the reaction mixture precipitated a white solid which was separated by centrifugation. The solid was washed with 2x2 mL of acetone and dried under vacuum to obtain the complex **2a,** as a mixture of isomers (16.80 mg, isolated yield 85 %).

Melting point: 232 °C (decomposed); ^{1}H NMR (500 MHz, DMSO-d_6, room temperature): δ 9.12 (1H, H_{f2}), 8.82 (1H, H_{i2}), 8.72 (1H, H_{a2}), 8.57 (1H, H_{b2}), 8.33 (1H, H_{g2}), 7.94 (1H, H_{d2}), 7.57 (1H, H_{h2}), 7.44 (1H, H_{c2}), 5.43 (1H, H_{e2}) [Multiplicity has not been given as it is a mixture of isomers]; ^{13}C NMR (125 MHz, DMSO-d_6, room temperature): δ 162.3, 155.0, 153.2, 153.1, 150.6, 149.3, 149.2, 141.9, 139.3, 139.23, 134.9, 128.7, 128.6, 127.6, 126.4, 118.0, 64.7. DOSY NMR (500 MHz, DMSO-d_6, 298 K): D = 1.12 x 10^{-10} m^2 s^{-1}; HRMS (ESI, DMSO): *m/z* Calcd. for $[\textbf{2a}–1•NO_3]^{1+}$ 1256.0705, found 1256.0688; Calcd. For $[\textbf{2a}–2•NO_3]^{2+}$ 597.0416, found 597.0410; Calcd. for $[\textbf{2a}–3•NO_3]^{3+}$ 377.3651, found 377.3639.

Synthesis of the complexes [X⊂$Pd_2(L2)_4](NO_3)_3$, 2b-2d

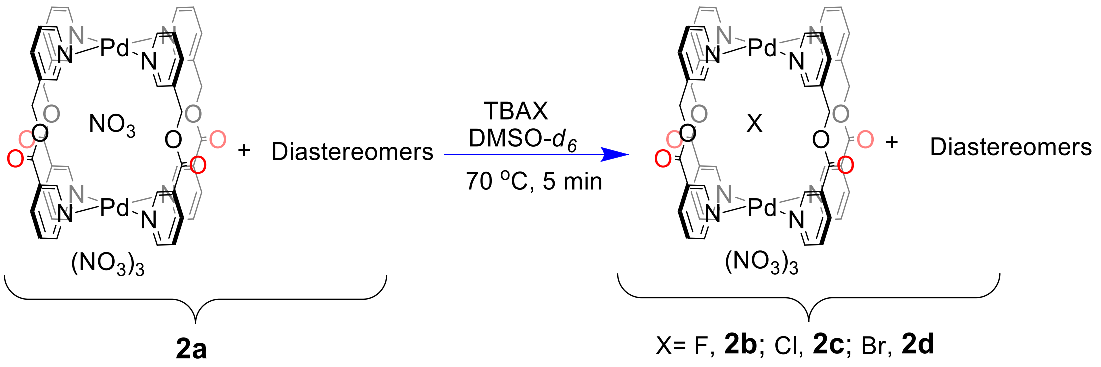

Synthesis of [F⊂$Pd_2(L2)_4](NO_3)_3$, 2b. A solution of tetra-*n*-butylammonium fluoride (1.30 mg, 0.005 mmol) in 0.1 mL of DMSO-d_6 was added to a clear solution of compound **2a** (6.58 g, 0.005 mmol) in 0.4 mL of DMSO-d_6 and heated at 70 °C for 5 mins. ^{1}H NMR spectrum of the solution was recorded at room temperature. The spectrum showed down field shift of pyridine α and β protons as compared to the compound **2a**, which indicated the quantitative formation of compound **2b** as a mixture of isomers. The NO_3^- ion present in **2a** was exchanged with F^- ion as evident from the ^{1}H NMR spectral data.

HRMS (ESI, DMSO): *m/z* Calcd. for [**2b**–1•$NO_3]^{1+}$ 1213.0810, found 1213.0725; Calcd. for [**2b**–3•$NO_3]^{3+}$ 363.0353, found 363.0348.

Synthesis of [Cl⊂$Pd_2(L2)_4](NO_3)_3$, 2c. The complex **2c** was prepared in a similar manner as described for **2b**, using a solution of tetra-*n*-butylammonium chloride (1.39 mg, 0.005 mmol) dissolved in 0.1 mL of DMSO-d_6. The ^{1}H NMR spectrum showed downfield shift of pyridine α and β protons as compared to the compound **2a**, which indicated the quantitative formation of compound **2c** as a mixture of isomers. The NO_3^- ion present in **2a** was exchanged with Cl^- ion as evident from the ^{1}H NMR spectral data.

Synthesis of [Br⊂$Pd_2(L2)_4](NO_3)_3$, 2d. The complex **2d** was prepared in a similar manner as described for **2b**, using a solution of tetra-*n*-butylammonium bromide (1.61 mg, 0.005 mmol) dissolved in 0.1 mL of DMSO-d_6. The ^{1}H NMR spectrum showed downfield shift of pyridine α and β protons as compared to the compound **2a**, which indicated the quantitative formation of compound **2d** as a mixture of isomers. The NO_3^- ion present in **2a** was exchanged with Br^- ion as evident from the ^{1}H NMR spectral data.

Synthesis of the complexes [X⊂$Pd_2(L2)_4$]$(BF_4)_4$, 2a'-2d'

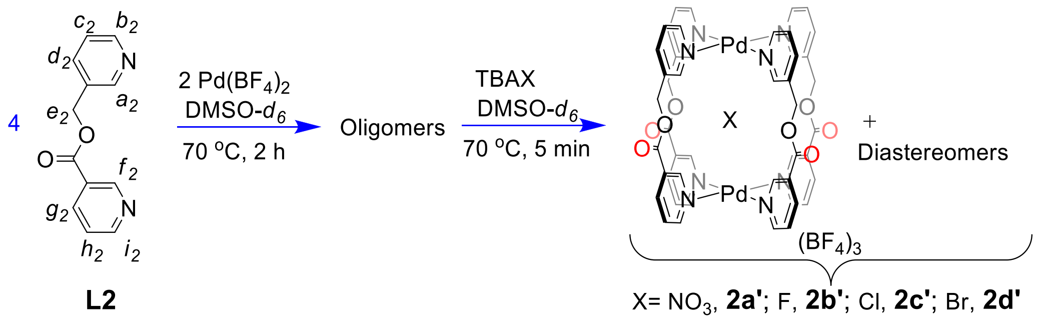

Synthesis of [NO_3⊂$Pd_2(L2)_4$]$(BF_4)_4$, 2a'. A solution of $Pd(BF_4)_2$ was prepared in 0.4 mL of DMSO-d_6 by stirring a mixture of PdI_2 (1.80 mg, 0.005 mmol) and $AgBF_4$ (1.95 mg , 0.010 mmol) at 90 °C for 30 min. The precipitated AgI was separated by centrifugation. The ligand **L2** (2.14 mg, 0.010 mmol) was added to the supernatant and the reaction mixture was stirred at 70 °C for 2 h. To this solution tetra-*n*-butylammonium nitrate (0.76 mg, 0.0026 mmol) was added and heated for 5 min at 70 °C, resulting in the formation of complex **2a'**. The ^{1}H NMR spectrum of the complex **2a'** is closely comparable with that of complex **2a**.

HRMS (ESI, DMSO): *m/z* Calcd. for [**2a'**–1•BF_4]$^{1+}$ 1305.1025, found 1305.0914; Calcd. for [**2a'**–2•BF_4]$^{2+}$ 609.5496, found 609.5459; Calcd. for [**2a'**–3•BF_4]$^{3+}$ 377.3651, found 377.3636.

Synthesis of [F⊂$Pd_2(L2)_4$]$(BF_4)_4$, 2b'. The complex **2b'** was prepared in a similar manner as described for **2a'**, using a solution of tetra-*n*-butylammonium fluoride (0.65 mg, 0.0026 mmol) dissolved in 0.1 mL of DMSO-d_6. The ^{1}H NMR spectrum of the complex **2b'** is closely comparable with that of complex **2b**.

Synthesis of [Cl⊂$Pd_2(L2)_4$]$(BF_4)_4$, 2c'. The complex **2c'** was prepared in a similar manner as described for **2a'**, using a solution of tetra-*n*-butylammonium chloride (0.69 mg, 0.0026 mmol) dissolved in 0.1 mL of DMSO-d_6. The ^{1}H NMR spectrum of the complex **2c'** is closely comparable with that of complex **2c**.

Synthesis of [Br⊂$Pd_2(L2)_4$]$(BF_4)_4$, 2d'. The complex **2d'** was prepared in a similar manner as described for **2a'**, using a solution of tetra-*n*-butylammonium bromide (0.80 mg, 0.0026 mmol) dissolved in 0.1 mL of DMSO-d_6. The ^{1}H NMR spectrum of the complex **2d'** is closely comparable with that of complex **2d**.

Synthesis of the ligand L3

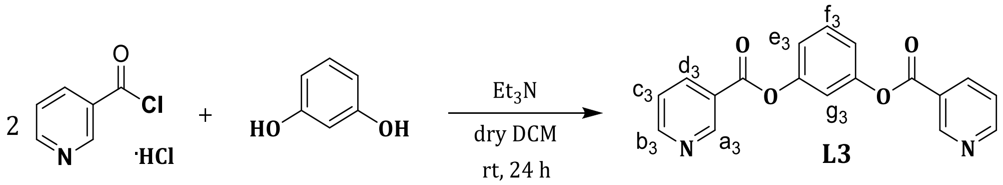

Triethylamine (1.02 mL, 7.246 mmol) was added in a dropwise manner to a stirred suspension of nicotinoyl chloride hydrochloride (1.290 g, 7.246 mmol) and resorcinol (0.400 g, 3.633 mmol) in dry dichloromethane (50 mL) maintained at 0-5 °C. The mixture was stirred at room temperature for 24 h under nitrogen atmosphere. In order to neutralise the acid, $NaHCO_3$ solution (10% *w/v*) was added slowly to the mixture until the evolution of CO_2 has ceased. The organic layer was washed with distilled water, separated and dried over anhydrous sodium sulfate. Complete evaporation of the solvent yielded an off-white solid, **L3** (0.872 g, yield 75%).

Melting point: 235°C; ^{1}H NMR (500 MHz, DMSO-d_6, room temperature): δ 9.28 (m, 2H, H_{a3}), 8.91 (m, 2H, H_{b3}), 8.48 (m, 2H, H_{d3}), 7.66 (m, 2H, H_{c3}), 7.60 (t, J = 8.3 Hz, 1H, H_{f3}), 7.43 (t, J = 2.3 Hz, 1H,H_{g3}), 7.34 (m, 2H, H_{e3}); ^{13}C NMR (125 MHz, DMSO-d_6, room temperature): δ 163.4, 154.3, 150.8, 150.5, 137.5, 130.2, 125.0, 124.1, 119.9, 116.2; HRMS (ESI, CH_2Cl_2/CH_3OH): *m/z* Calcd. for $C_{18}H_{12}N_2O_4$: 320.2989, Found 321.0680 $[M+H]^+$

Synthesis of the complex $[Pd_3(L3)_6](NO_3)_6$, 3a

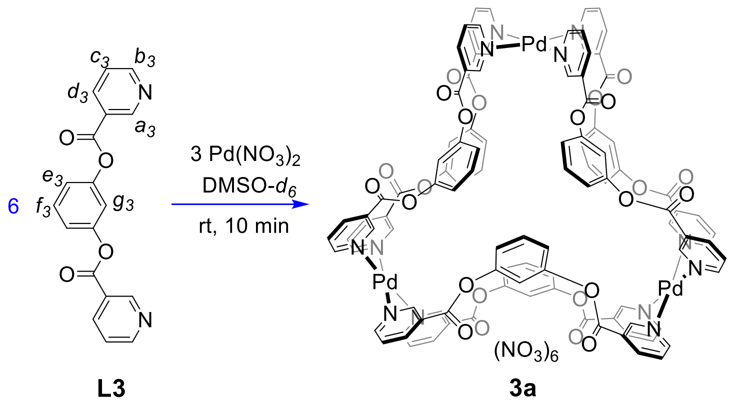

The ligand **L3** (19.21 mg, 0.059 mmol) was added to a solution of $Pd(NO_3)_2$ (6.91 mg, 0.029 mmol) in 3 mL of DMSO. The reaction mixture was stirred at room temperature for 10 min. Subsequent, slow diffusion of toluene vapour into the reaction mixture precipitated a crystalline solid which was separated by filtration. The solid was dried under vacuum to yield the complex **3a** (22.72 mg, isolated yield 58 %).

Melting point: 258 °C (decomposed); ^{1}H NMR (500 MHz, DMSO-d_6, room temperature): δ 10.24 (bs, 2H, H_{a3}), 9.68 (bs, 2H, H_{b3}), 8.65 (bs, 2H, H_{d3}), 7.98 (m, 2H, H_{c3}), 7.67 (t, J = 8.15 Hz, 1H, H_{f3}), 7.37 (m, J = 2.3 Hz, 3H, H_{e3}/H_{g3}); ^{13}C NMR (125 MHz, DMSO-d_6, room temperature): δ 161.7, 154.9, 152.6, 150.7, 141.6, 130.7, 128.6, 127.7, 120.6, 116.8; DOSY NMR (500 MHz, DMSO-d_6, 298 K): D = 9.54 x 10^{-11} m^2 s^{-1}; HRMS (ESI, DMSO): *m/z* Calcd. for [**3a** –2•NO_3]$^{2+}$ 1244.0733, found 1244.0722; Calcd. for [**3a** –5•NO_3]$^{5+}$ 460.4369, found 460.4373.

Synthesis of the complexes [Pd$_3$(L3)$_6$](X)$_6$, 3b-3f

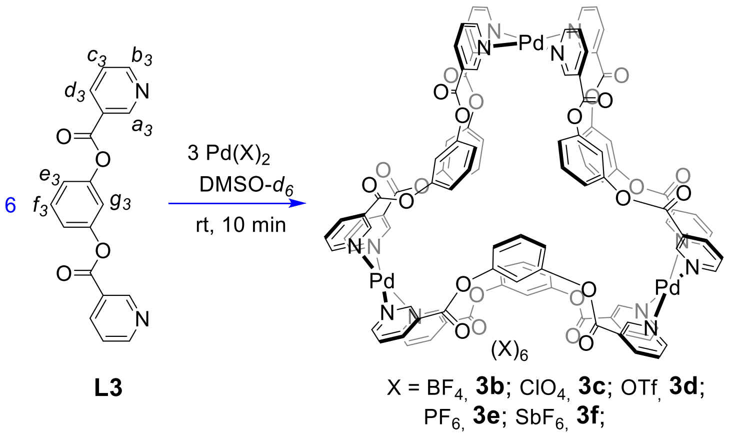

Synthesis of [Pd$_3$(L3)$_6$](BF$_4$)$_6$, 3b. A solution of Pd(BF_4)$_2$ was prepared in 0.5 mL of DMSO-d_6 by stirring a mixture of PdI_2 (1.80 mg, 0.005 mmol) and $AgBF_4$ (1.95 mg , 0.010 mmol) at 90 °C for 30 min. The precipitated AgI was separated by centrifugation. The ligand **L3** (3.20 mg, 0.010 mmol) was added to the supernatant and the reaction mixture was stirred at room temperature for 10 min. The ^{1}H NMR spectrum of the complex **3b** is closely comparable with that of complex **3a** except for H_{g3}, 7.30 (s, 1H, H_{g3}).

HRMS (ESI, DMSO): *m/z* Calcd. for [**3b**–3•BF_4]$^{3+}$ 833.7355, found 833.7359; Calcd. for [**3b**–4•BF_4]$^{4+}$ 603.5511, found 603.5510; Calcd. for [**3b**–5•BF_4]$^{5+}$ 465.4402, found 465.4403.

Synthesis of [Pd$_3$(L3)$_6$](ClO$_4$)$_6$, 3c. A solution of Pd(ClO_4)$_2$ was prepared in 0.5 mL of DMSO-d_6 by stirring a mixture of PdI_2 (1.80 mg, 0.005 mmol) and $AgClO_4$ (2.07 mg , 0.010 mmol) at 90 °C for 30 min. The precipitated AgI was separated by centrifugation. The ligand **L3** (3.20 mg, 0.010 mmol) was added to the supernatant and the reaction mixture was stirred at room temperature for 10 min. The ^{1}H NMR spectrum of the complex **3c** is closely comparable with that of complex **3a** except for H_{g3}, 7.29 (s, 1H, H_{g3}).

Synthesis of $[Pd_3(L3)_6](OTf)_6$, 3d. A solution of $Pd(OTf)_2$ was prepared in 0.5 mL of DMSO-d_6 by stirring a mixture of PdI_2 (1.80 mg, 0.005 mmol) and AgOTf (2.57 mg , 0.010 mmol) at 90 °C for 30 min. The precipitated AgI was separated by centrifugation. The ligand **L3** (3.20 mg, 0.010 mmol) was added to the supernatant and the reaction mixture was stirred at room temperature for 10 min. The ^1H NMR spectrum of the complex **3d** is closely comparable with that of complex **3a** except for H_{g3}, 7.30 (s, 1H, H_{g3}).

Synthesis of $[Pd_3(L3)_6](PF_6)_6$, 3e. A solution of $Pd(PF_6)_2$ was prepared in 0.5 mL of DMSO-d_6 by stirring a mixture of PdI_2 (1.80 mg, 0.005 mmol) and $AgPF_6$ (2.53 mg , 0.010 mmol) at 90 °C for 30 min. The precipitated AgI was separated by centrifugation. The ligand **L3** (3.20 mg, 0.010 mmol) was added to the supernatant and the reaction mixture was stirred at room temperature for 10 min. The ^1H NMR spectrum of the complex **3e** is closely comparable with that of complex **3a** except for H_{g3}, 7.22 (s, 1H, H_{g3}).

Synthesis of $[Pd_3(L3)_6](SbF_6)_6$, 3f. A solution of $Pd(SbF_6)_2$ was prepared in 0.5 mL of DMSO-d_6 by stirring a mixture of PdI_2 (1.80 mg, 0.005 mmol) and $AgSbF_6$ (3.44 mg , 0.010 mmol) at 90 °C for 30 min. The precipitated AgI was separated by centrifugation. The ligand **L3** (3.20 mg, 0.010 mmol) was added to the supernatant and the reaction mixture was stirred at room temperature for 10 min. The ^1H NMR spectrum of the complex **3f** is closely comparable with that of complex **3a** except for H_{g3}, 7.29 (s, 1H, H_{g3}).

Synthesis of the ligand, **L5 via multiple steps.**

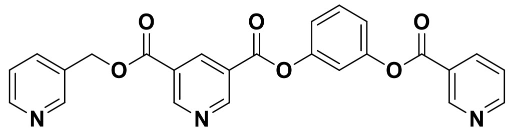

L5

Synthesis of this ligand **L5** involves three steps. The first step involves the synthesis of 3-hydroxyphenyl nicotinate. For the synthesis of 5-((pyridin-3-ylmethoxy)carbonyl)nicotinic acid selective deprotection of the bis(pyridin-3-ylmethyl) pyridine-3,5-dicarboxylate ligand, **L1** was carried out in THF:H_2O using acid and base. Synthesis of bis(pyridin-3-ylmethyl) pyridine-3,5-dicarboxylate was carried out by following literature procedure[1]. Second step involves the synthesis of 3-hydroxyphenyl nicotinate from resorcinol and nicotinic acid in dry DCM using EDC·HCl as coupling reagent.

Synthesis of the ligand, **L4**

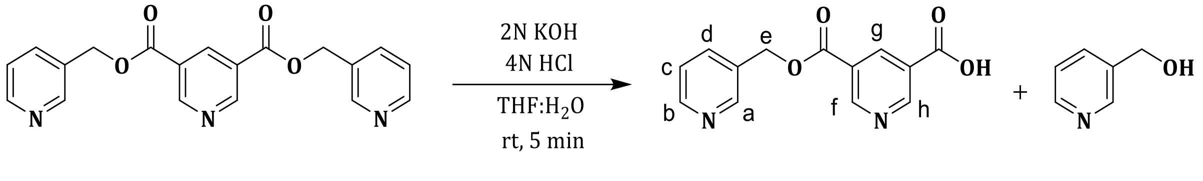

L1 **L4**

Bis(pyridin-3-ylmethyl)pyridine-3,5-dicarboxylate, **L1** (0.500 g, 1.431 mmol) was dissolved in 35 mL of THF:H_2O (1:1) to obtain a clear solution. To this 1 mL of 2N KOH was added and stirred at room temperature for 2 min, followed by addition of 0.5 mL of 4N HCl. The reaction mixture was dried under *vacuo* yielding a solid (0.358 g, 97%) which was further purified by column chromatography using DCM: MeOH (3:7) to obtain ligand **L4**.

Melting point: 223 °C; TLC (DCM: MeOH, 97:3 v/v): R_f = 0.3; ^{1}H NMR (500 MHz, DMSO-d_6, room temperature): δ 9.31 (s, 1H, H_f/H_h), 9.28 (s, 1H, H_f/H_h), 8.75 (m, 1H, H_a), 8.65 (m, 1H, H_g), 8.60 (m, 1H, H_b), 7.98 (m, 1H, H_d), 7.48 (m, 1H, H_c), 5.46 (s, 2H, H_e); ^{13}C NMR (125 MHz, DMSO-d_6, room temperature): δ 165.3, 163.9, 154.0, 153.4, 149.4, 149.3, 137.3, 136.4, 131.4, 126.8, 125.5, 123.8, 64.7; HRMS (ESI, CH_2Cl_2/CH_3OH): *m/z* Calcd. for $C_{13}H_{10}N_2O_4$: 258.2295, Found 259.0722 $[M+H]^+$.

Synthesis of the ligand, **L4'**

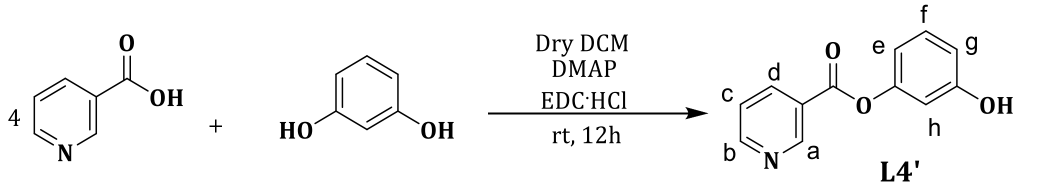

L4'
3-hydroxyphenyl nicotinate

To a suspension of nicotinic acid (0.500 g, 4.065 mmol) and resorcinol (0.112 g, 1.018 mmol) in 50 ml dry DCM maintained at 0-5 °C, DMAP (0.062 g, 0.507 mmol) was added followed by EDC·HCl (0.195 g, 1.017 mmol). The reaction mixture was stirred for 12 h under nitrogen atmosphere. In order to neutralise the acid, $NaHCO_3$ solution (10% *w/v*) was added slowly to the mixture until the evolution of CO_2 has ceased. The organic layer was washed with distilled water, separated and dried over anhydrous sodium sulfate. On standing the product precipitated out. It was isolated by filtration and dried under *vacuo* to obtain a pale pink powder.

^{1}H NMR (500 MHz, DMSO-d_6, room temperature): δ 9.80 (d, J = 7.5 Hz, 1H, -OH), 9.24 (m, 1H, H_a), 8.89 (m, 1H, H_b), 8.44 (m, 1H, H_d), 7.64 (m, 1H, H_c), 7.25 (m, 1H), 6.71 (dd, J_1 = 8.2 Hz, J_2 = 2.0 Hz, 3H); ^{13}C NMR (125 MHz, DMSO-d_6, room temperature): δ 163.5, 158.4, 154.2, 151.2 150.5, 137.5, 130.0, 125.2, 124.1, 113.3, 112.2, 109.1.

Synthesis of the ligand, **L5**

The final step involves the condensation of 5-((pyridine-3-ylmethoxy)carbonyl)nicotinic acid and 3-hydroxyphenyl nicotinate using EDC·HCl as coupling reagent under nitrogen atmosphere at room temperature.

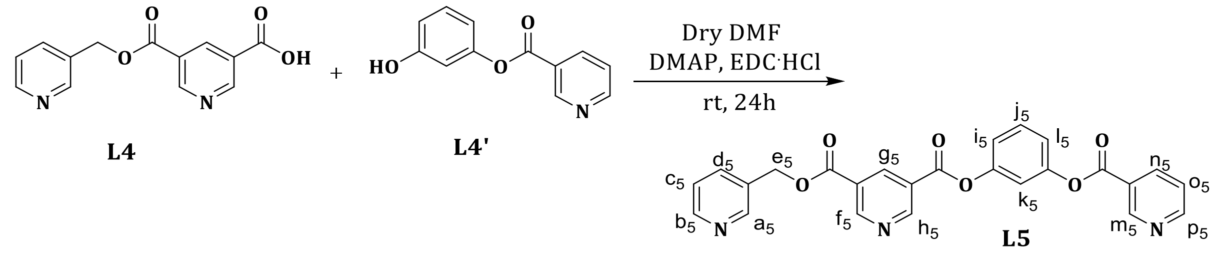

To a suspension of 5-((pyridine-3-ylmethoxy)carbonyl)nicotinic acid (0.250 g, 0.968 mmol) and 3-hydroxyphenyl nicotinate (0.208 g, 0.968 mmol) in 15 ml dry DMF maintained at 0-5 °C, DMAP (0.059 g, 0.484 mmol) was added followed by addition of EDC•HCl (0.186 g, 0.968 mmol). The reaction mixture was stirred for 24 h under nitrogen atmosphere. Addition of water to the reaction mixture resulted in the precipitation of product. The product was isolated by filtration and dried under *vacuo* to obtain an off white solid powder (0.287 g, 65%).

Melting point: 238 °C; ^{1}H NMR (500 MHz, DMSO-d_6, room temperature): δ 9.48 (d, J = 2.1 Hz, 1H, H_{f5}/H_{h5}), 9.41 (d, J = 2.0 Hz, 1H, H_{f5}/H_{h5}), 9.27 (d, J = 1.4 Hz, 1H, H_{m5}), 8.91 (m, 1H, H_{p5}), 8.83 (s, 1H, H_{g5}), 8.75 (s, 1H, H_{a5}), 8.58 (m, 1H, H_{b5}), 8.49 (m, 1H, H_{n5}), 7.88 (m, 1H, H_{d5}), 7.67 (m, 1H, H_{o5}), 7.61 (t, J = 8.2 Hz, 1H), 7.45 (m, 2H), 7.36 (m, 2H), 5.49 (s, 2H, H_{e5}); ^{13}C NMR (125 MHz, DMSO-d_6, room temperature): δ 163.7, 163.4, 162.6, 154.3, 154.2, 150.8, 150.7, 150.6, 149.5, 149.5, 137.7, 137.5, 136.2, 131.3, 130.2, 125.7, 125.3, 124.9, 124.1, 123.7, 120.0, 119.8, 116.1, 64.8; HRMS (ESI, CH_2Cl_2/CH_3OH): *m/z* Calcd. for $C_{25}H_{17}N_3O_6$: 455.4190, Found 456.1196 $[M+H]^{+}$.

Synthesis of the complex [NO_3⊂$Pd_4(L3)_2(L5)_4$]$(NO_3)_7$, 4a

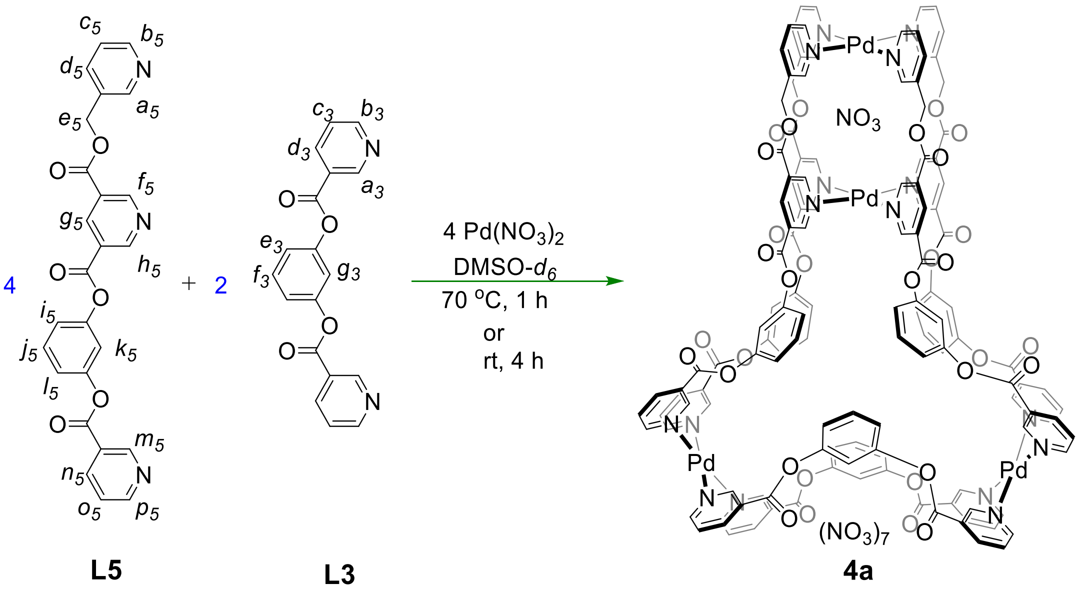

The ligands **L3** (8.00 mg, 0.025 mmol) and **L5** (22.77 mg, 0.050 mmol) were added to a solution of $Pd(NO_3)_2$ (11.52 mg, 0.050 mmol) in 5 mL of DMSO. The reaction mixture was stirred at 70 °C for 1 h to obtain **4a**. Subsequent, slow diffusion of toluene vapour to the resulting solution precipitated a crystalline solid which was separated by filtration. The solid was dried under vacuum to obtain the complex **4a** (18.98 mg, isolated yield 45 %).

Melting point: 260 °C (decomposed); ^{1}H NMR (500 MHz, DMSO-d_6, room temperature): δ 11.02 (s, 4H, H_{f5}), 10.65 (s, 4H,H_{h5}), 10.25 (d, J = 3.5 Hz, 8H, H_{m5}, H_{a3}), 10.05 (s, 4H, H_{a5}), 9.66 (d, J = 4.2 Hz, 8H, H_{p5}, H_{b3}), 9.35 (d, J = 4.5 Hz, 4H, H_{b5}), 8.92 (s, 4H, H_{g5}), 8.66 (d, J = 6.5 Hz, 8H, H_{n5}, H_{d3}), 8.16 (m, 4H, H_{d5}), 8.00-7.97 (m, 8H, H_{o5}, H_{c3}), 7.82 (m, 4H, H_{c5}), 7.70-7.66 (m, 4H), 7.41-7.35 (m, 16H), 5.56 (m, 8H, H_{e5}); ^{13}C NMR (125 MHz, DMSO-d_6, room temperature): δ 161.9, 161.8, 161.3, 156.6, 155.0, 152.6, 150.8, 150.8, 149.4, 141.6, 139.5, 134.8, 130.8, 129.1, 129.0, 128.7, 128.6, 127.8, 126.7, 120.9, 120.7, 117.0, 65.3; DOSY NMR (500 MHz, DMSO-d_6, 298 K): D = 8.51 x 10^{-11} m^2 s^{-1}; HRMS (ESI, DMSO): m/z Calcd for [**4a** –3•NO_3]$^{3+}$ 1065.7228, found 1065.7184; Calcd for [**4a** –6•NO_3]$^{6+}$ 501.8678, found 501.8661.

Synthesis of the complexes [X⊂$Pd_4(L1)_2(L2)_4](NO_3)_7$, 4b-4d

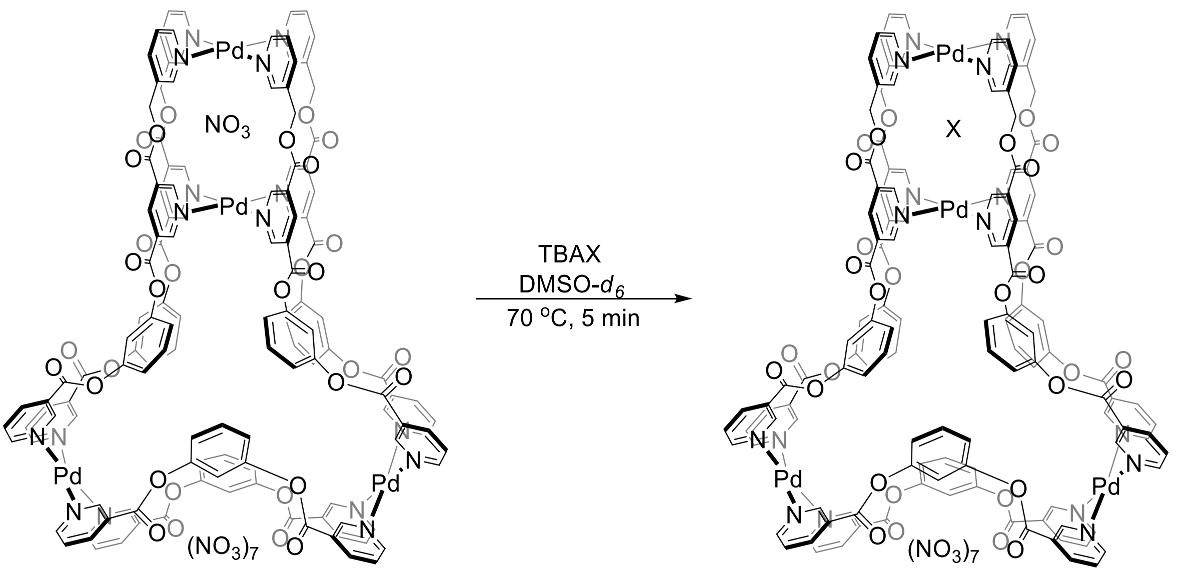

4a

X = F, **4b**; Cl, **4c**; Br, **4d**

Synthesis of [F⊂$Pd_4(L1)_2(L2)_4](NO_3)_7$, 4b. The ligands **L5** (2.28 mg, 0.005 mmol) and **L3** (0.80 mg, 0.0025 mmol) were dissolved in 0.4 mL of DMSO-d_6, to which $Pd(NO_3)_2$ (1.15 mg, 0.005 mmol) was added. The solution was stirred at 70 °C for 1 h to obtain the complex [(NO_3)⊂Pd_4(**L3**)$_2$(**L5**)$_4$]$(NO_3)_7$, **4a**. A solution of tetra-*n*-butylammonium fluoride (0.33 mg, 0.0012 mmol) in 0.1 mL of DMSO-d_6 was added to the *in situ* prepared complex **4a** and heated at 70 °C for 5 min. ^{1}H NMR spectrum of the solution was recorded at room temperature. The spectrum showed a single set of peaks and downfield shift of pyridine α and β protons as compared to the complex **4a**, which indicated the quantitative formation of complex **4b**. The NO_3^- ion present in **4a** was exchanged with F^- ion as evident from the ^{1}H NMR spectral data.

Synthesis of [Cl⊂$Pd_4(L1)_2(L2)_4](NO_3)_7$, 4c. The complex **4c** was prepared in a similar manner as described for **4b**, using a solution of tetra-*n*-butylammonium chloride (0.35 mg, 0.0012 mmol) in 0.1 mL of DMSO-d_6. The ^{1}H NMR spectrum showed a single set of peaks and downfield shift of pyridine α and β protons as compared to the complex **4a**, which indicated the quantitative formation of complex **4c**. The NO_3^- ion present in **4a** was exchanged with Cl^- ion as evident from the ^{1}H NMR spectral data. HRMS (ESI, DMSO): *m/z* Calcd for [**4c** – 6•$NO_3]^{6+}$ 497.5311, found 497.5617.

Synthesis of [Br⊂$Pd_4(L1)_2(L2)_4](NO_3)_7$, 4d. The complex **4d** was prepared in a similar manner as described for **4b**, using a solution of tetra-*n*-butylammonium bromide (0.40 mg,

0.0012 mmol) in 0.1 mL of DMSO-d_6. The ^{1}H NMR spectrum showed a single set of peaks and downfield shift of pyridine α and β protons as compared to the complex **4a**, which indicated the quantitative formation of complex **4d**. The NO_3^- ion present in **4a** was exchanged with Br^- ion as evident from the ^{1}H NMR spectral data.

HRMS (ESI, DMSO): *m/z* Calcd for [**4d** –2•NO_3]$^{2+}$ 1638.5427, found 1638.5401; Calcd for [**4d** –3•NO_3]$^{2+}$ 1071.6994, found 1071.6998; Calcd for [**4d** –4•NO_3]$^{4+}$ 788.2777, found 788.2782.

Synthesis of the complexes [X⊂Pd_4(L3)$_2$(L5)$_4$](BF_4)$_7$, 4a'-4d'

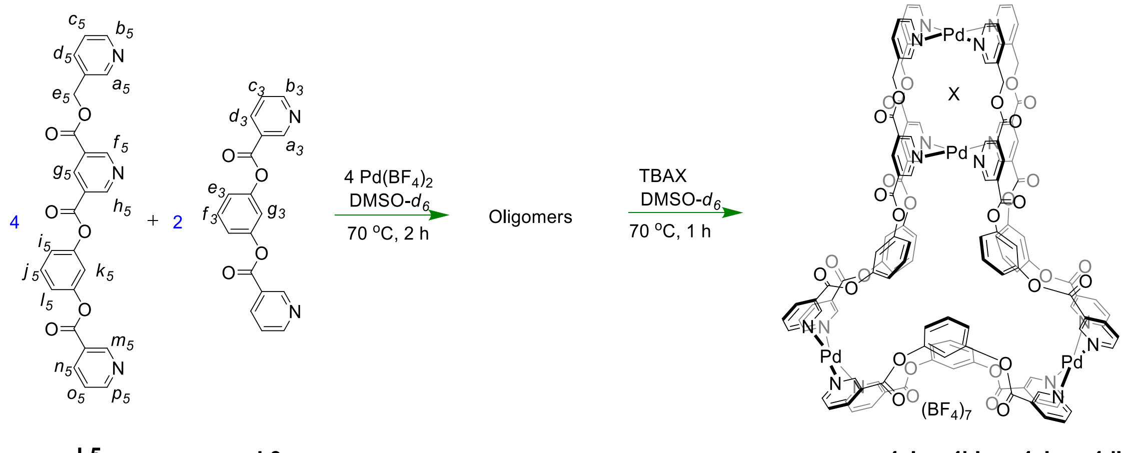

Synthesis of [NO_3⊂Pd_4(L3)$_2$(L5)$_4$](BF_4)$_7$, 4a'. A solution of $Pd(BF_4)_2$ was prepared in 0.4 mL of DMSO-d_6 by stirring a mixture of PdI_2 (1.80 mg, 0.005 mmol) and $AgBF_4$ (1.95 mg , 0.010 mmol) at 90 °C for 30 min. The precipitated AgI was separated by centrifugation. The ligands **L3** (0.80 mg, 0.003 mmol) and **L5** (2.28 mg, 0.005 mmol) were added the supernatant and heated at 70 °C for 2 h, yielding an oligomer. To the solution containing the oligomer, tetra-*n*-butylammonium nitrate (0.38 mg, 0.0012 mmol) in 0.1 mL of DMSO-d_6 was added and heated at 70 °C for 1 h resulting in the formation of complex **4a'**. The ^{1}H NMR spectrum of the complex **4a'** is closely comparable with the data of complex **4a**.

Synthesis of [F⊂Pd_4(L3)$_2$(L5)$_4$](BF_4)$_7$, 4b'. The complex **4b'** was prepared in a similar manner as described for **4a'**, using a solution of tetra-*n*-butylammonium fluoride (0.33 mg, 0.0012 mmol) in 0.1 mL of DMSO-d_6. The ^{1}H NMR spectrum of the complex **4b'** is closely comparable with the data of complex **4b**.

Synthesis of [Cl⊂Pd_4(L3)$_2$(L5)$_4$](BF_4)$_7$, 4c'. The complex **4c'** was prepared in a similar manner as described for **4a'**, using a solution of tetra-*n*-butylammonium chloride (0.35 mg, 0.0012 mmol) in 0.1 mL of DMSO-d_6. The ^{1}H NMR spectrum of the complex **4c'** is closely comparable with the data of complex **4c**.

Synthesis of [Br⊂Pd_4(L3)$_2$(L5)$_4$](BF_4)$_7$, 4d'. The complex **4d'** was prepared in a similar manner as described for **4a'**, using a solution of tetra-*n*-butylammonium bromide (0.41 mg, 0.0012 mmol) in 0.1 mL of DMSO-d_6. The ^{1}H NMR spectrum of the complex **4d'** is closely comparable with the data of complex **4d**.

Synthesis of ligand L6

The synthesis of ligand **L6** is a two-step process. The first step involves the synthesis of 5-((pyridine-3-ylmethoxy)carbonyl)nicotinic acid, followed by its condensation with resorcinol under nitrogen atmosphere at room temperature, using EDC·HCl as coupling reagent.

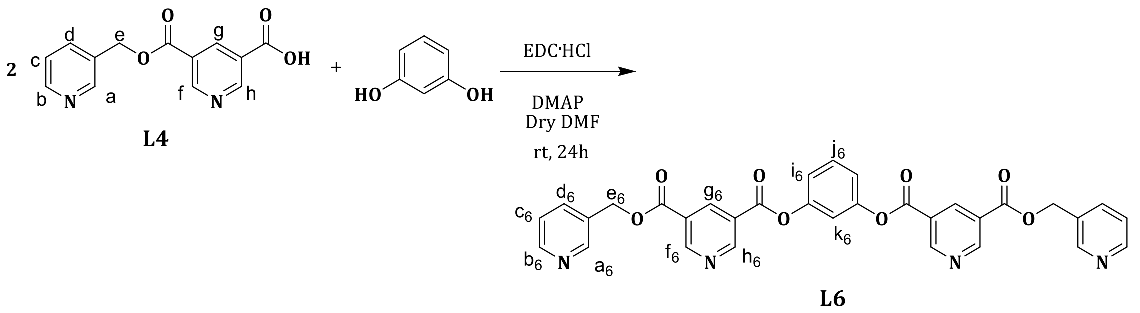

To a suspension of 5-((pyridine-3-ylmethoxy)carbonyl)nicotinic acid (0.469 g, 1.816 mmol) and resorcinol (0.100 g, 0.908 mmol) in 15 ml dry DMF maintained at 0-5 °C, DMAP (0.055 g, 0.4541 mmol) was added followed by EDC·HCl (0.348 g, 1.816 mmol). The reaction mixture was stirred for 24 h under nitrogen atmosphere. Addition of water to the reaction mixture resulted in the precipitation of product. The product was isolated by filtration and dried under *vacuo* to obtain an off white solid.

Melting point: 251 °C; ^{1}H NMR (500 MHz, DMSO-d_6, room temperature): δ 9.49 (s, 2H, H_{f6}/ H_{h6}), 9.42 (s, 2H, H_{h6}/ H_{f6}), 8.83 (s, 2H, H_{g6}), 8.75 (s, 2H, H_{a6}), 8.58 (m, 2H, H_{b6}), 7.97 (m, 2H, H_{d6}), 7.62 (t, J = 8.3 Hz, 3H, H_{j6}), 7.46 (m, 2H, H_{c6}, H_{k6}), 7.37 (m, 2H, H_{i6}), 5.49 (s, 4H, H_{e6}); ^{13}C NMR (125 MHz, DMSO-d_6, room temperature): δ 163.7, 162.6, 154.2, 150.6, 149.6, 149.5, 137.7, 136.2, 131.3, 130.3, 125.7, 125.3, 123.7, 123.7, 120.0, 116.1, 64.8; HRMS (ESI, CH_2Cl_2/CH_3OH): *m/z* Calcd. for $C_{32}H_{22}N_4O_8$: 590.5391, Found 591.1501 [M+H]$^+$.

Synthesis of the complex [$(NO_3)_2$⊂$Pd_5(L5)_4(L6)_2$]$(NO_3)_8$, 5a

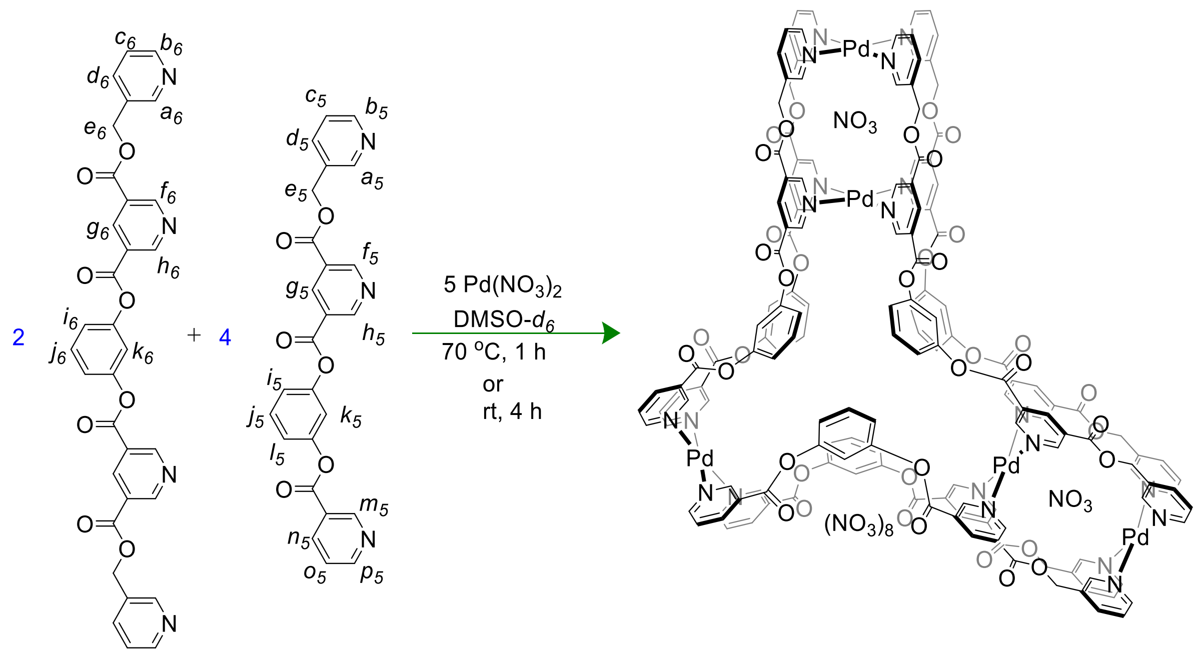

L6 **L5** **5a**

The ligands **L5** (18.22 mg, 0.040 mmol) and **L6** (11.81 mg, 0.020 mmol) were added to a solution of $Pd(NO_3)_2$ (11.52 mg, 0.050 mmol) in 5 mL of DMSO. The reaction mixture was stirred at 70 °C temperature for 2 h. Subsequently, slow diffusion of toluene vapour to the reaction mixture precipitated a crystalline solid which was separated by filtration. The solid was dried under vacuum to obtain the complex **5a** (15.76 mg, isolated yield 38 %).

Melting point: 272 °C (decomposed); ^{1}H NMR (500 MHz, DMSO-d_6, room temperature): δ 11.04 (s, 8H, H_{f5}, H_{f6}), 10.67 (s, 8H, H_{h5}, H_{h6}), 10.26 (d, 4H, H_{m5}), 10.06 (s, 8H, H_{a5}, H_{a6}), 9.68 (s, 4H, H_{p5}), 9.36 (d, 8H, H_{b5}, H_{b6}), 8.94 (s, 8H, H_{g5}, H_{g6}), 8.67 (d, J = 8.2 Hz, 4H, H_{n5}), 8.18 (bs, 8H, H_{d5}, H_{d6}), 8.01-7.98 (m, 4H, H_{o5}), 7.85-7.82 (m, 8H, H_{c5}, H_{c6}), 7.75-7.71 (m, 4H), 7.48-7.41 (m, 16H), 5.64-5.53 (m, 16H, H_{e5}, H_{e5}); ^{13}C NMR (125 MHz, DMSO-d_6, room temperature): δ 161.8, 161.3, 156.5, 150.8, 149.3, 141.5, 139.5, 134.8, 130.8, 128.9, 128.6, 126.6, 120.9, 116.8, 65.2; DOSY NMR (500 MHz, DMSO-d_6, 298 K): D = 5.75 x 10^{-11} m^2 s^{-1}; HRMS (ESI, DMSO): m/z Calcd. for [**5a** –3•NO_3]$^{3+}$ 1322.7259, found 1322.7248; Calcd. for [**5a** –4•NO_3]$^{4+}$ 976.5475, found 976.5474.

Synthesis of the complexes [(X)$_2$⊂Pd$_5$(L5)$_4$(L6)$_2$](NO$_3$)$_8$, 5b-5d

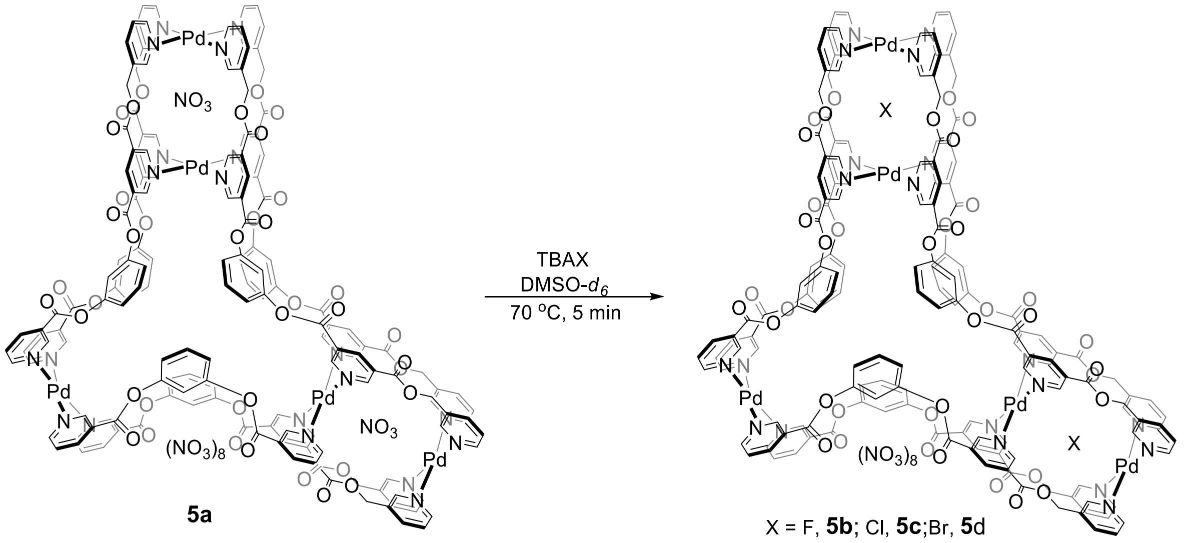

Synthesis of [(F)$_2$⊂Pd$_5$(L5)$_4$(L6)$_2$](NO$_3$)$_8$, 5b. Mixture of ligands **L5** (1.82 mg, 0.004 mmol) and **L6** (1.18 mg, 0.002 mmol) was dissolved in 0.4 mL of DMSO-d_6, to which Pd(NO$_3$)$_2$ (1.15 mg, 0.005 mmol) was added and stirred at 70 °C for 1 h to obtain the complex [(NO$_3$)$_2$⊂Pd$_5$(**L5**)$_4$(**L6**)$_2$](NO$_3$)$_8$, **5a**. A solution of tetra-*n*-butylammonium fluoride (0.52 mg, 0.002 mmol) in 0.1 mL of DMSO-d_6 was added (in four portions of 25 µL each) to the *in situ* prepared compound **5a** and heated at 70 °C for 5 min. ^{1}H NMR spectra (room temperature) were recorded after the addition of each portion of the guest. The final spectrum showed a single set of peaks and downfield shift of pyridine α and β protons as compared to the complex **5a**, which indicated the quantitative formation of complex **5b**. The NO$_3^-$ ions present in **5a** were exchanged with F$^-$ ions as evident from the ^{1}H NMR spectral data.

Synthesis of [(Cl)$_2$⊂Pd$_5$(L5)$_4$(L6)$_2$](NO$_3$)$_8$, 5c. The complex **5c** was prepared in a similar manner as described for **5b**. A solution of tetra-*n*-butylammonium chloride (0.56 mg, 0.002 mmol) in 0.1 mL of DMSO-d_6 was added (in four portions of 25 µL each) to the *in situ* prepared compound **5a** and heated at 70 °C for 5 min. ^{1}H NMR spectra (room temperature) were recorded after the addition of each portion of the guest. The final spectrum showed a single set of peaks and downfield shift of pyridine α and β protons as compared to the complex **5a**, which indicated the quantitative formation of complex **5c**. The NO$_3^-$ ions present in **5a** were exchanged with Cl$^-$ ions as evident from the ^{1}H NMR spectral data.

Synthesis of [(Br)$_2$⊂Pd$_5$(L5)$_4$(L6)$_2$](NO$_3$)$_8$, 5d. The complex **5d** was prepared in a similar manner as described for **5b**. A solution of tetra-*n*-butylammonium bromide (0.65 mg, 0.002 mmol) in 0.1 mL of DMSO-*d*$_6$ was added (in four portion of 25 μL each) to the *in situ* prepared compound **5a** and heated at 70 °C for 5 min. ^{1}H NMR spectra (room temperature) were recorded after the addition of each portion of the guest. The final spectrum showed a single set of peaks and downfield shift of pyridine α and β protons as compared to the complex **5a**, which indicated the quantitative formation of complex **5d**. The NO_3^- ions present in **5a** were exchanged with Br^- ions as evident from the ^{1}H NMR spectral data.

Synthesis of the complexes [(X)$_2$⊂Pd$_5$(L5)$_2$(L6)$_4$](NO$_3$)$_8$, 5a'-5d'

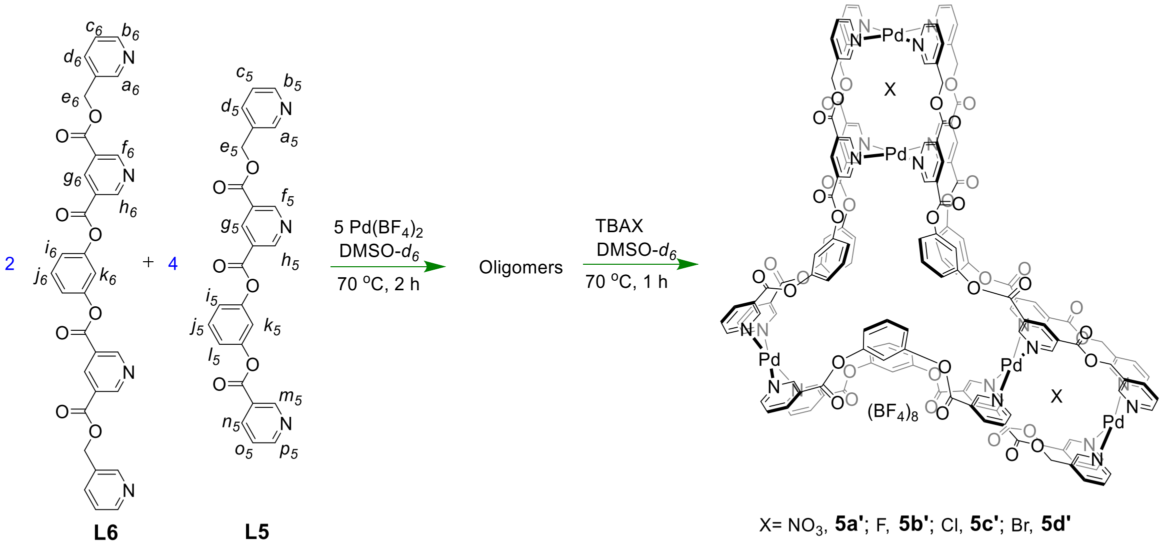

Synthesis of [(NO$_3$)$_2$⊂Pd$_5$(L5)$_2$(L6)$_4$](NO$_3$)$_8$, 5a'. A solution of Pd(BF$_4$)$_2$ was prepared in 0.4 mL of DMSO-*d*$_6$ by stirring a mixture of PdI$_2$ (1.80 mg, 0.005 mmol) and AgBF$_4$ (1.95 mg , 0.010 mmol) at 90 °C for 30 min. The precipitated AgI was separated by centrifugation. The ligands **L5** (1.82 mg, 0.004 mmol) and **L6** (1.18 mg, 0.002 mmol) were added the supernatant and heated at 70 °C for 2 h to obtain the oligomer. To the solution containing the oligomer, a solution of tetra-*n*-butylammonium nitrate (0.61 mg, 0.002 mmol) in 0.1 mL of DMSO-*d*$_6$ was added and heated at 70 °C for 1 h resulting in the formation of the complex **5a'**. The ^{1}H NMR spectrum of the complex **5a'** is closely comparable with the data of complex **5a**.

Synthesis of [(F)$_2$⊂Pd$_5$(L5)$_2$(L6)$_4$](NO$_3$)$_8$, 5b'. The complex **5b'** was prepared in a similar manner as described for **5a'**, using a solution of tetra-*n*-butylammonium fluoride (0.52 mg, 0.002 mmol) in 0.1 mL of DMSO-*d*$_6$. The ^{1}H NMR spectrum of the complex **5b'** is closely comparable with the data of complex **5b**.

Synthesis of $[(Cl)_2\subset Pd_5(L5)_2(L6)_4](NO_3)_8$, 5c'. The complex **5c'** was prepared in a similar manner as described for **5a'**, using a solution of tetra-*n*-butylammonium chloride (0.56 mg, 0.002 mmol) in 0.1 mL of DMSO-d_6. The ^1H NMR spectrum of the complex **5c'** is closely comparable with the data of complex **5c**.

Synthesis of $[(Br)_2\subset Pd_5(L5)_2(L6)_4](NO_3)_8$, 5d'. The complex **5d'** was prepared in a similar manner as described for **5a'**, using a solution of tetra-*n*-butylammonium bromide (0.65 mg, 0.002 mmol) in 0.1 mL of DMSO-d_6. The ^1H NMR spectrum of the complex **5d'** is closely comparable with the data of complex **5d**.

Synthesis of the complex $[(NO_3)_3\subset Pd_6(L6)_6](NO_3)_9$, 6a

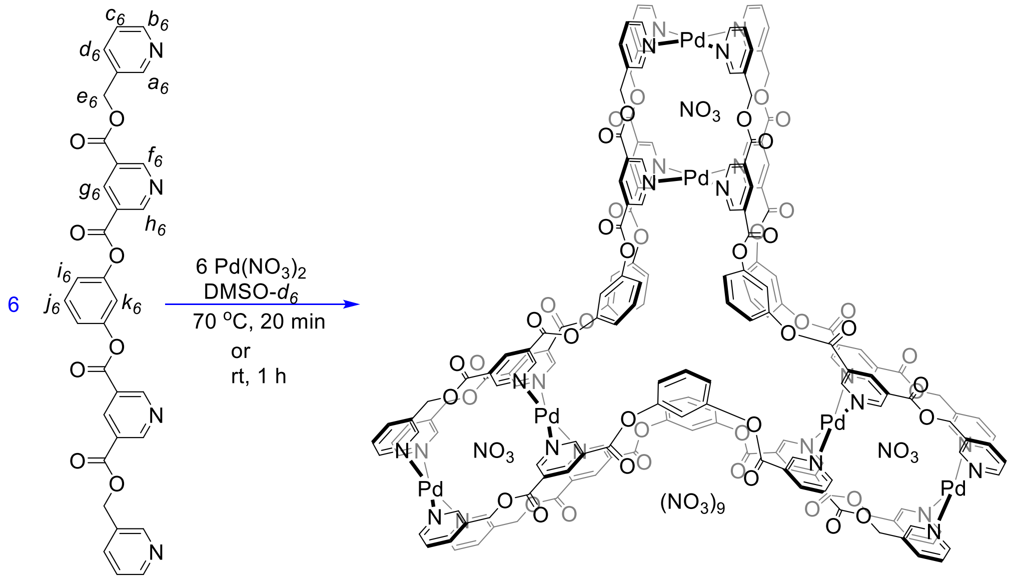

L6 **6a**

The ligand **L6** (29.53 mg, 0.050 mmol) was added to a solution of $Pd(NO_3)_2$ (11.52 mg, 0.050 mmol) in 5 mL of DMSO. The reaction mixture was stirred at 70 °C for 20 min to obtain **6a**. Subsequently, addition of 15 mL of ethyl acetate to the reaction mixture precipitated a white solid which was separated by centrifugation. The solid was washed with 3x3 mL of acetone and dried under vacuum to obtain the complex **6a** (36.98 mg, isolated yield 90 %).

Melting point: 288 °C (decomposed); ^1H NMR: 11.04 (s, 12H, H_{f6}), 10.68 (s, 12H, H_{h6}), 10.06 (s, 12H, H_{a6}), 9.37 (d, J = 8.2 Hz, 12H, H_{b6}), 8.94 (s, 12H, H_{g6}), 8.19 (d, J = 7.2 Hz, 12H, H_{d6}), 7.80 (s, 12H, H_{c6}), 7.75 (bs, 6H), 7.48 (bs, 18H), 5.58 (bs, 24H, H_{e5}); HRMS (ESI, DMSO): *m/z* Calcd. for $[\mathbf{6a} - 5{\bullet}NO_3]^{5+}$ 923.0424, found 923.0411.

Synthesis of the complexes [(X)$_3$⊂Pd$_6$(L6)$_6$](NO$_3$)$_9$, 6b-6d

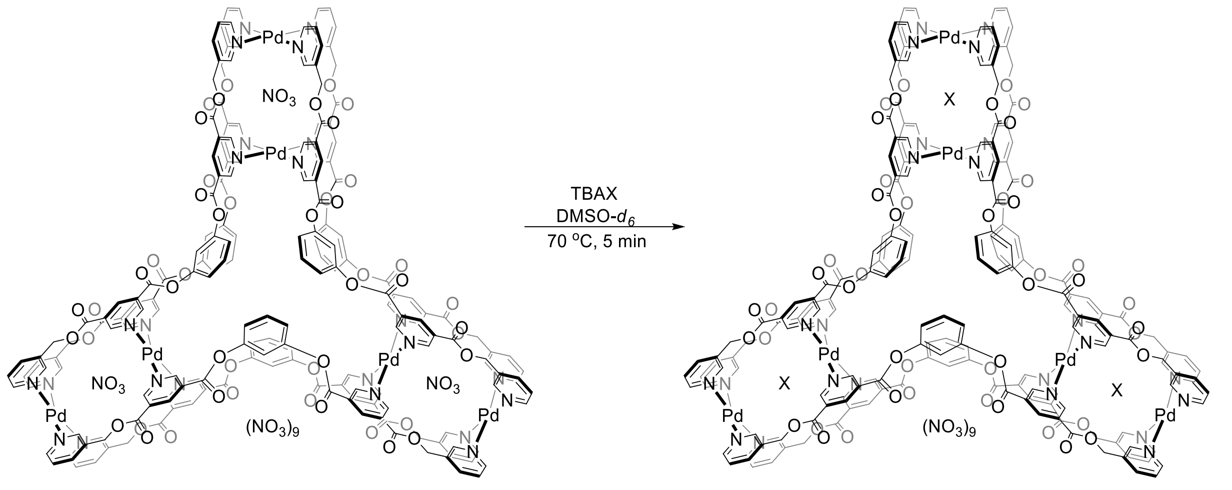

6a X = F, **6b**; Cl, **6c**; Br, **6d**

Synthesis of [(NO$_3$)$_3$⊂Pd$_6$(L6)$_6$](NO$_3$)$_9$, 6b. To a solution ligand **L6** (2.95 mg, 0.005 mmol) in 0.5 mL of DMSO-*d$_6$*, Pd(NO$_3$)$_2$ (1.15 mg, 0.005 mmol) was added and stirred at 70 °C for 20 min to obtain the complex [(NO$_3$)$_3$⊂Pd$_6$(**L6**)$_6$](NO$_3$)$_9$, **6a**. A solution of tetra-*n*-butylammonium fluoride (0.65 mg, 0.0025 mmol) in 0.1 mL of DMSO-*d$_6$* was added (in four portions of 25 µL each) to the *in situ* prepared complex **6a** and was heated at 70 ℃ for 5 min. ^{1}H NMR spectra (room temperature) were recorded after the addition of each portion of the guest. The final spectrum showed a single set of peaks and downfield shift of pyridine α and β protons as compared to the complex **6a**, which indicated the quantitative formation of complex **6b**. The NO_3^- ions present in **6a** were exchanged with F^- ions as evident from the ^{1}H NMR spectral data.

Synthesis of [(NO$_3$)$_3$⊂Pd$_6$(L6)$_6$](NO$_3$)$_9$, 6c. The complex **6c** was prepared in a similar manner as described for **6b**. A solution of tetra-*n*-butylammonium chloride (0.70 mg, 0.0025 mmol) dissolved in 0.1 mL of DMSO-*d$_6$* was added (in four portions of 25 µL each) to the *in situ* prepared complex **6a** and heated at 70 ℃ for 5 min. ^{1}H NMR spectra (room temperature) were recorded after the addition of each portion of the guest. The final spectrum showed a single set of peaks and downfield shift of pyridine α and β protons as compared to the complex **6a**, which indicated the quantitative formation of complex **6c**. The NO_3^- ions present in **6a** were exchanged with Cl^- ions as evident from the ^{1}H NMR spectral data.

HRMS (ESI, DMSO): *m/z* Calcd for [**6c**–4•NO$_3$]$^{4+}$ 1149.5352, found 1149.5310.

Synthesis of $[(NO_3)_3\subset Pd_6(L6)_6](NO_3)_9$, 6d. The complex **6d** was prepared in a similar manner as described for **6b**. A solution of tetra-*n*-butylammonium bromide (0.81 mg, 0.0025 mmol) in 0.1 mL of DMSO-d_6 was added (in four portions of 25 µL each) to the *in situ* prepared complex **6a** and heated at 70 °C for 5 min. ^{1}H NMR spectra (room temperature) were recorded after the addition of each portion of the guest. The final spectrum showed a single set of peaks and downfield shift of pyridine α and β protons as compared to the complex **6a**, which indicated the quantitative formation of single complex **6d**. The NO_3^- ions present in **6a** were exchanged with Br^- ions as evident from the ^{1}H NMR spectral data.

Synthesis of the complexes $[(X)_3\subset Pd_6(L6)_6](BF_4)_9$, 6a'-6d'

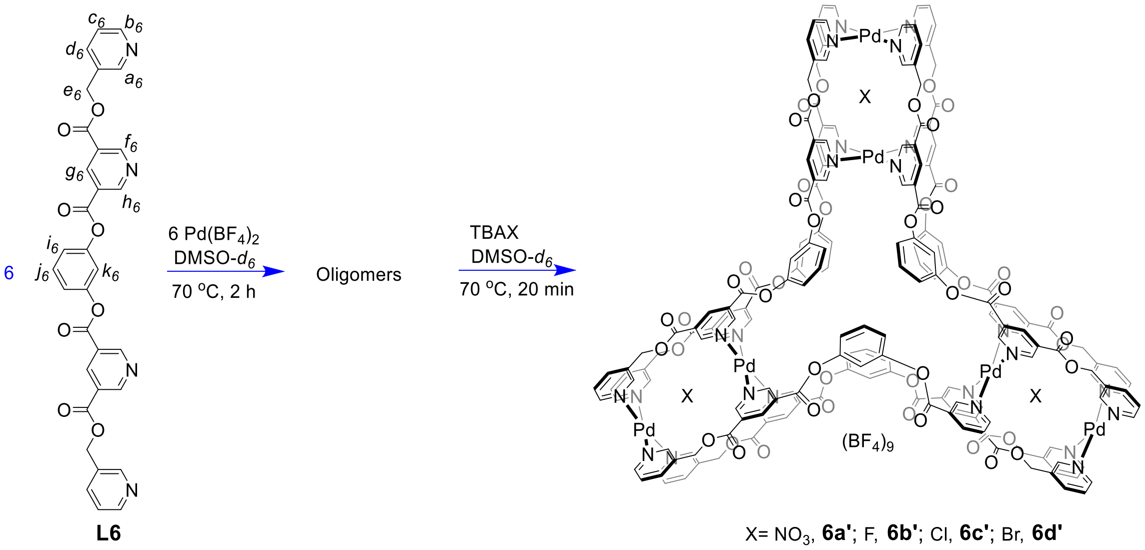

Synthesis of $[(NO_3)_3\subset Pd_6(L6)_6](BF_4)_9$, 6a'. A solution of $Pd(BF_4)_2$ in 0.4 mL of DMSO-d_6 was prepared by stirring a mixture of PdI_2 (1.80 mg, 0.005 mmol) and $AgBF_4$ (1.95 mg , 0.010 mmol) at 90 °C for 30 min. The precipitated AgI was separated by centrifugation. The ligand **L6** (2.95 mg, 0.005 mmol) was added to the supernatant and heated at 70 °C for 2 h to obtain the oligomer. To the solution containing the oligomer, a solution of tetra-*n*-butylammonium nitrate (0.76 mg, 0.0025 mmol) was added and heated at 70 °C for 20 min resulting in the formation of complex **6a'**. The ^{1}H NMR spectrum of the complex **6a'** is closely comparable with the data of complex **6a**.

Synthesis of $[(F)_3\subset Pd_6(L6)_6](BF_4)_9$, 6b'. The complex **6b'** was prepared in a similar manner as described for **6a'**, using a solution of tetra-*n*-butylammonium fluoride (0.65 mg, 0.0025

mmol) in 0.1 mL of DMSO-d_6. The ^{1}H NMR spectrum of the complex **6b'** is closely comparable with the data of complex **6b**.

Synthesis of [(Cl)$_3$⊂Pd$_6$(L6)$_6$](BF$_4$)$_9$, 6c'. The complex **6c'** was prepared in a similar manner as described for **6a'**, using a solution of tetra-*n*-butylammonium chloride (0.70 mg, 0.0025 mmol) in 0.1 mL of DMSO-d_6. The ^{1}H NMR spectrum of the complex **6c'** is closely comparable with the data of complex **6c**.

Synthesis of [(Br)$_3$⊂Pd$_6$(L6)$_6$](BF$_4$)$_9$, 6d'. The complex **6d'** was prepared in a similar manner as described for **6a'**, using a solution of tetra-*n*-butylammonium bromide (0.81 mg, 0.0025 mmol) in 0.1 mL of DMSO-d_6. The ^{1}H NMR spectrum of the complex–**6d'** is closely comparable with the data of complex **6d**.

Synthesis of a mixture of the complexes

(i) [Pd$_2$(L3)$_4$](NO$_3$)$_4$, 3g and [Pd$_3$(L3)$_6$](NO$_3$)$_6$, 3a

(ii) [Pd$_3$(L3)$_6$](NO$_3$)$_6$, 3a and [Pd$_4$(L3)$_8$](NO$_3$)$_8$, 3h

Minor proportions of other complexes that coexist with **3a** at lower/higher concentration were detected and identified. The formation of negligible amounts of the binuclear complex, **3g** and the tetranuclear complex, **3h** along with complex **3a** was detected at 1mM and 30 mM concentration of palladium(II) respectively. The percentages are with respect to the proportion of ligand **L3**.

❖ **Concentration of palladium(II): 1 mM**

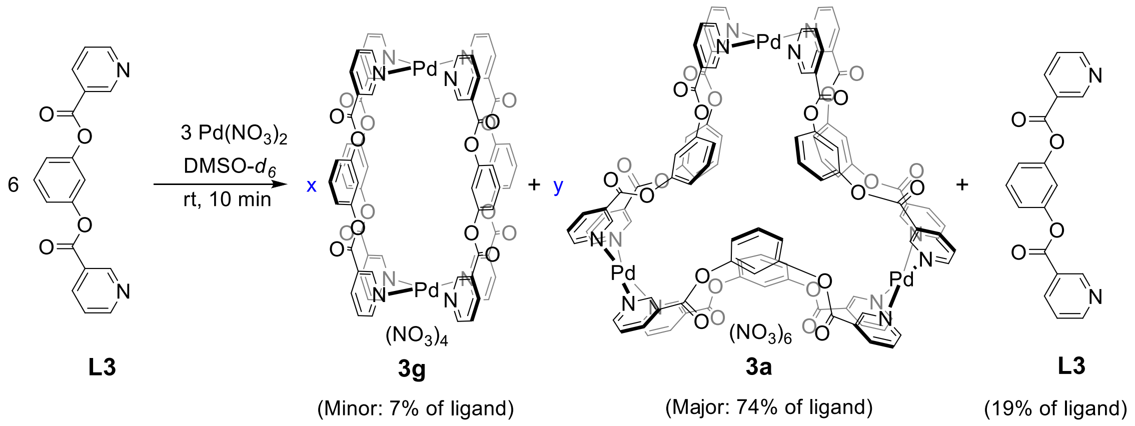

❖ Concentration of palladium(II): 30 mM

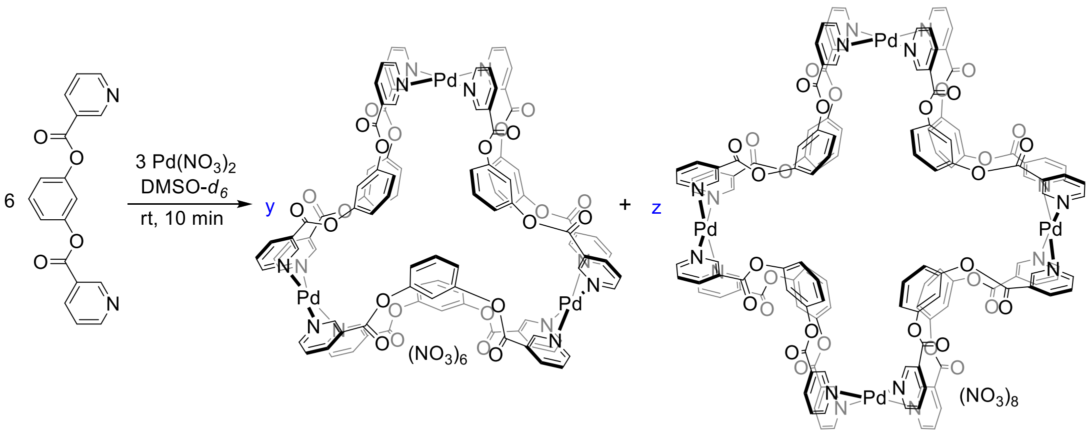

Synthesis of a mixture of the complexes $[NO_3 \subset Pd_3(L5)_4](NO_3)_5$, 4e and $[(NO_3)_2 \subset Pd_6(L5)_8](NO_3)_{10}$, 4f.

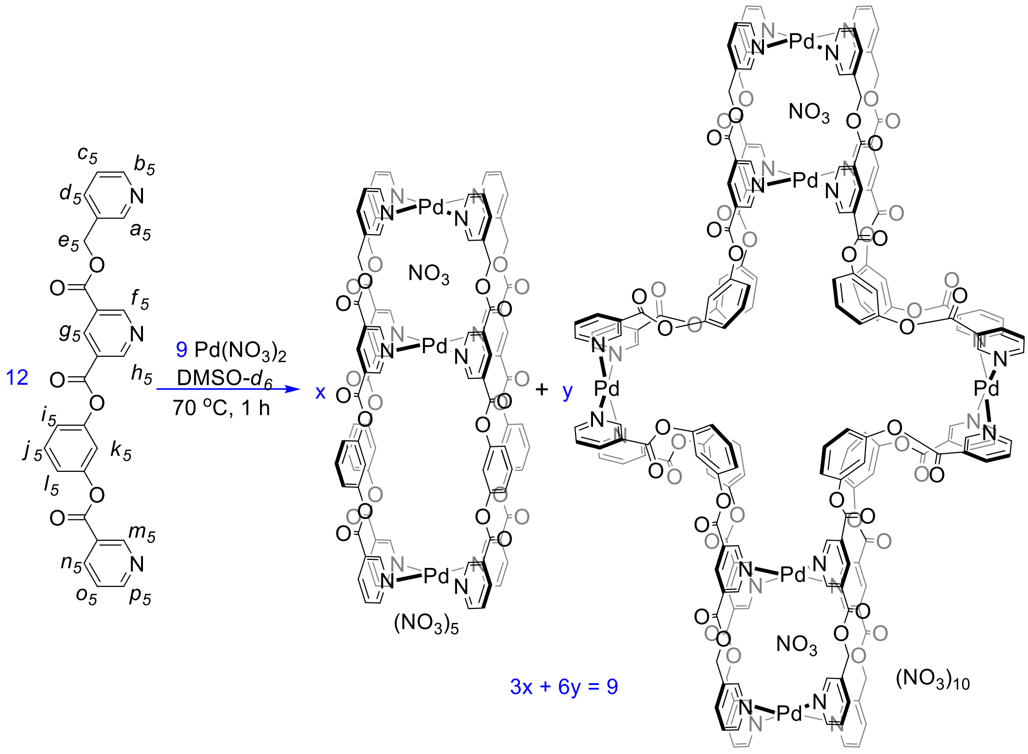

The ligand **L5** (3.04 mg, 0.007 mmol) was added to a solution of $Pd(NO_3)_2$ (1.15 mg, 0.005 mmol) in 0.5 mL of DMSO. The reaction mixture was stirred at 70 °C temperature for 1 h. The ^1H NMR spectrum indicated the formation of a mixture of two complexes which were identified as **4e** and **4f** by ESI-MS.

^{1}H NMR: 11.04, 11.00 (s, 2H, H_{f5}), 10.68, 10.55 (bs, 2H, H_{h5}), 10.26, 10.10 (bs, 1H, H_{m5}), 10.06 (bs, 3H, H_{a5}, H_{m5}), 9.70 (bs, 2H, H_{p5}), 9.37 (bs, 2H, H_{b5}), 8.95, 8.92 (s, 2H, H_{g5}), 8.71 (d, *J* = 6.5 Hz, 1H, H_{n5}), 8.67 (d, *J* = 5.1 Hz, 1H, H_{n5}), 8.17 (bs, 2H, H_{d5}), 7.99 (bs, 2H, H_{o5}), 7.83 (bs, 2H, H_{c5}), 7.78-7.70 (m, 4H), 7.43 (bs, 6H), 5.57 (bs, 4H, H_{e5}, H_{e5}); HRMS (ESI, DMSO): *m/z* Calcd. for [**4e**–3•NO_3]$^{3+}$ 775.3762, found 775.3746; Calcd. for [**4e**–4•NO_3]$^{4+}$ 566.0351, found 566.0339; Calcd. for [**4f**–5•NO_3]$^{5+}$ 943.0497, found 943.0465.

Synthesis of a mixture of the complexes [Cl⊂Pd_3(L5)$_4$](NO_3)$_5$, 4g and [(Cl)$_2$⊂Pd_6(L5)$_8$](NO_3)$_{10}$, 4h.

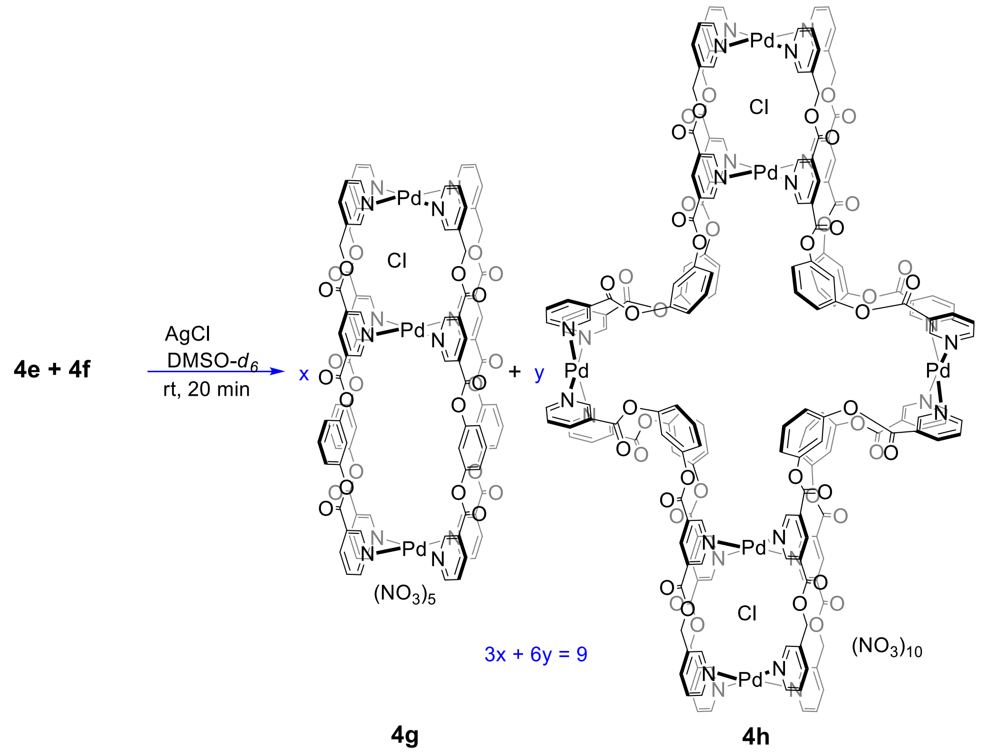

A mixture of complexes **4e** and **4f** in DMSO-*d6*, when stirred with a two-fold excess of AgCl at room temperature for 30 min, yielded the corresponding complexes **4g** and **4h** with encapsulation of Cl^- ions.

^{1}H NMR: 11.5 (bs, 2H, H_{f5}), 10.55 (bs, 2H, H_{h5}), 10.50 (s, 2H, H_{a5}), 10.26, 10.10 (bs, 1H, H_{m5}), 9.70 (bs, 4H, H_{p5}, H_{b5}), 8.95, 8.92 (s, 2H, H_{g5}), 8.71 (d, *J* = 6.5 Hz, 1H, H_{n5}), 8.67 (d, *J* = 5.1 Hz, 1H, H_{n5}), 8.17 (bs, 2H, H_{d5}), 7.99 (bs, 2H, H_{o5}), 7.83 (bs, 2H, H_{c5}), 7.78-7.70 (m, 4H), 7.43 (bs, 6H), 5.57 (bs, 4H, H_{e5}, H_{e5}); HRMS (ESI, DMSO): *m/z* Calcd. for [**4g**–3•NO_3]$^{3+}$ 766.7028, found 766.7018; Calcd. for [**4h**–7•NO_3]$^{7+}$ 648.3184, found 648.3199.

Characterisation of the ligands and the complexes

Characterisation of the ligand L2

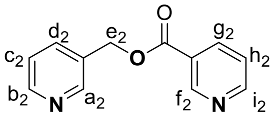

L2

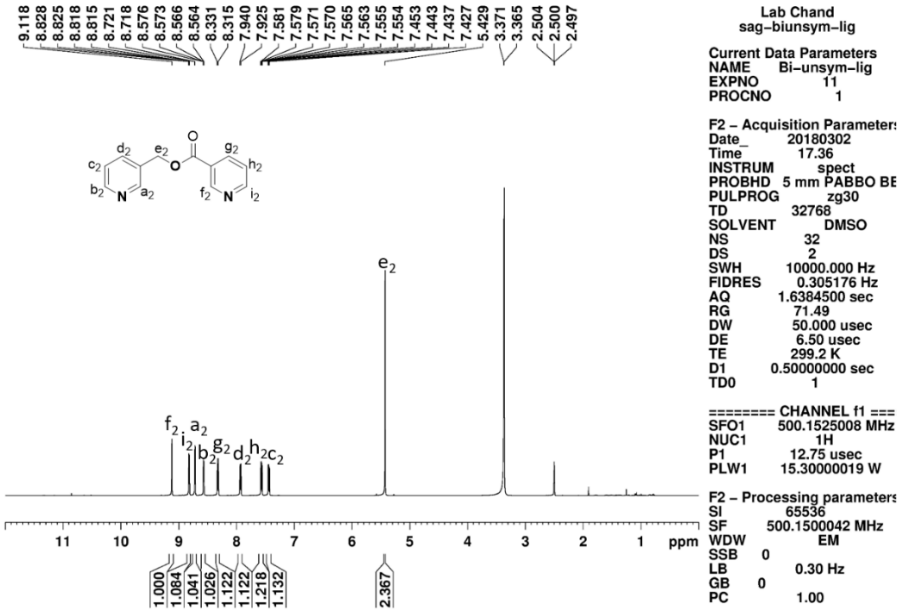

Supplementary Figure 1. ^{1}H NMR spectrum (500 MHz, DMSO-d_6, 300 K) for the ligand **L2**.

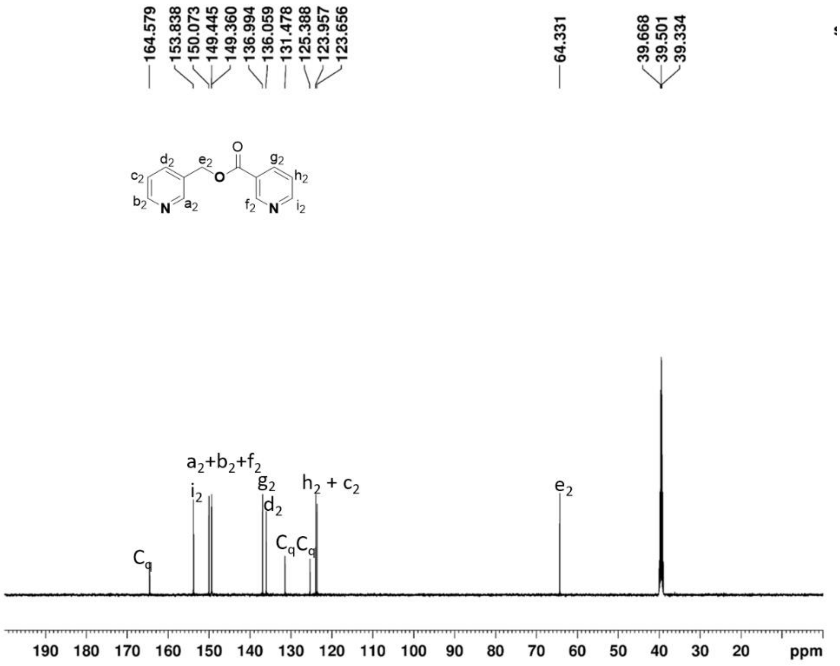

Supplementary Figure 2. ^{13}C NMR spectrum (125 MHz, DMSO-d_6, 300 K) for the ligand **L2**.

a

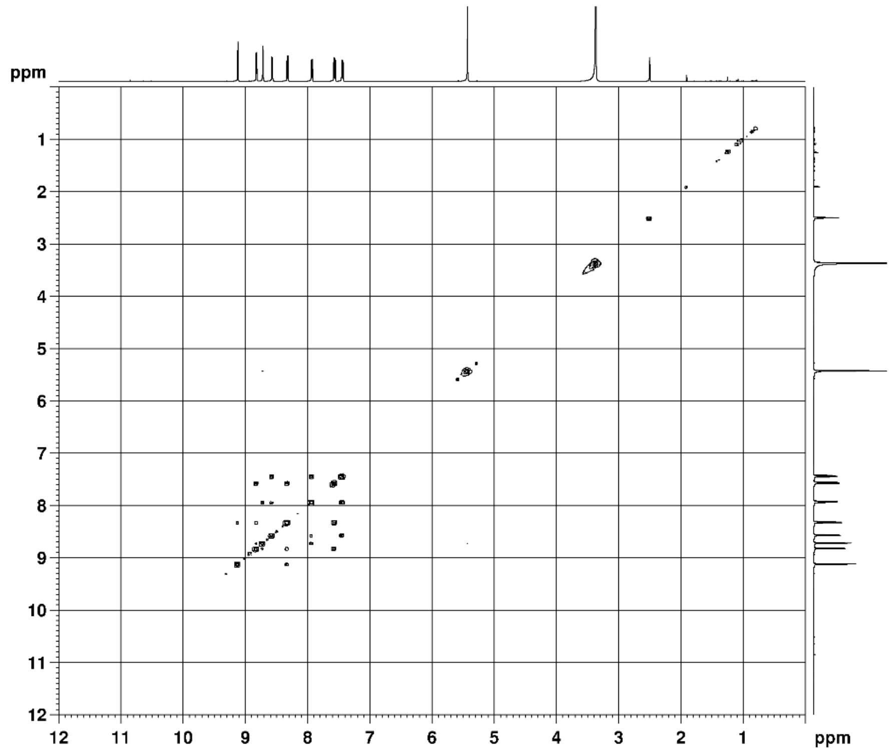

b

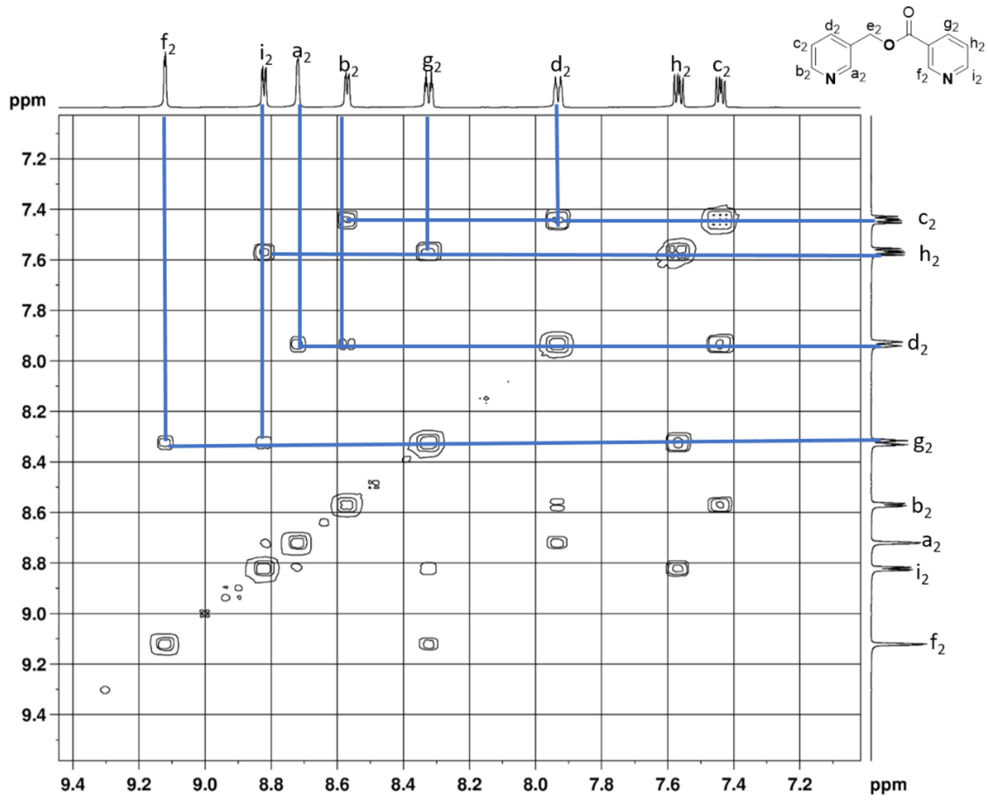

Supplementary Figure 3. H-H COSY spectrum (500 MHz, DMSO-d_6, 300 K) for **a** the ligand **L2**, and **b** expansion of the spectrum.

a

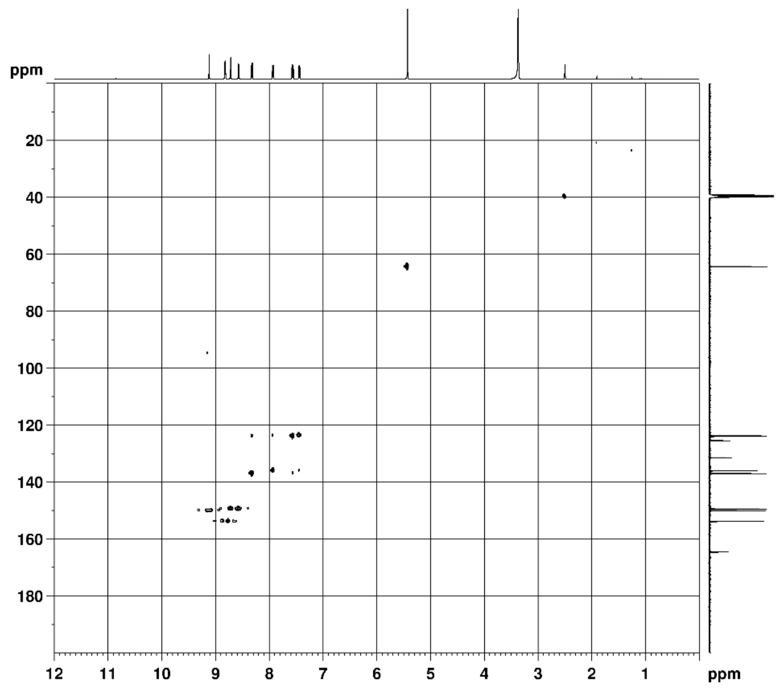

b

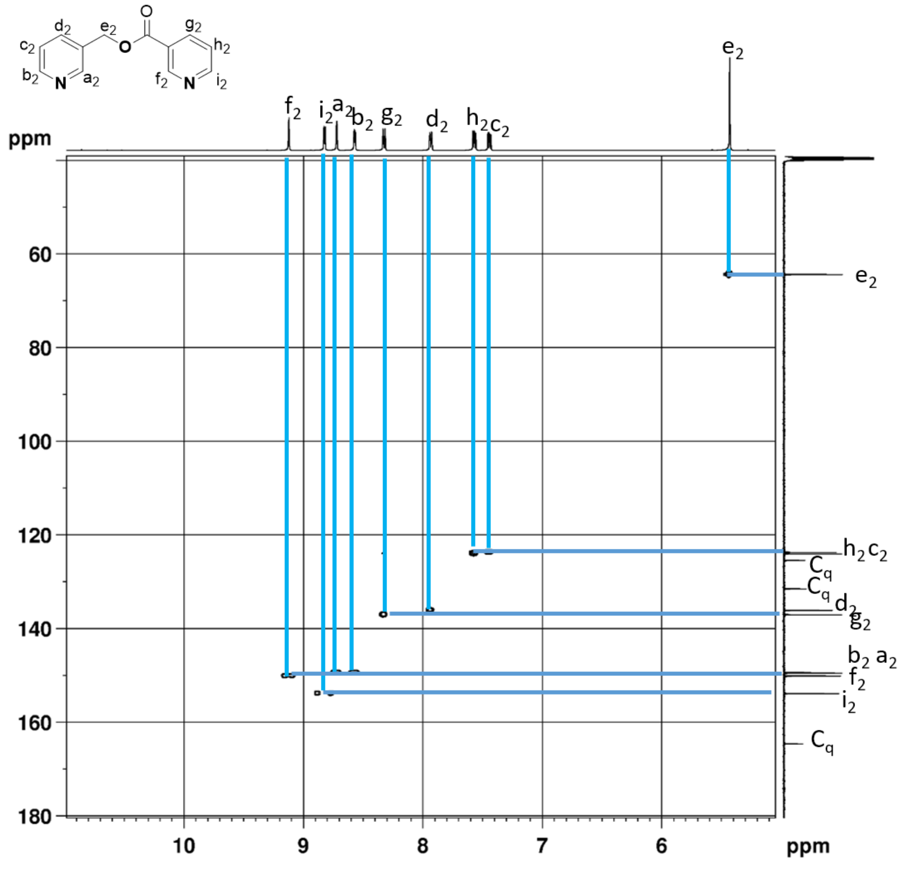

Supplementary Figure 4. C-H COSY spectrum (500 MHz, DMSO-d_6, 300 K) for **a** the ligand **L2**, and **b** expansion of the spectrum.

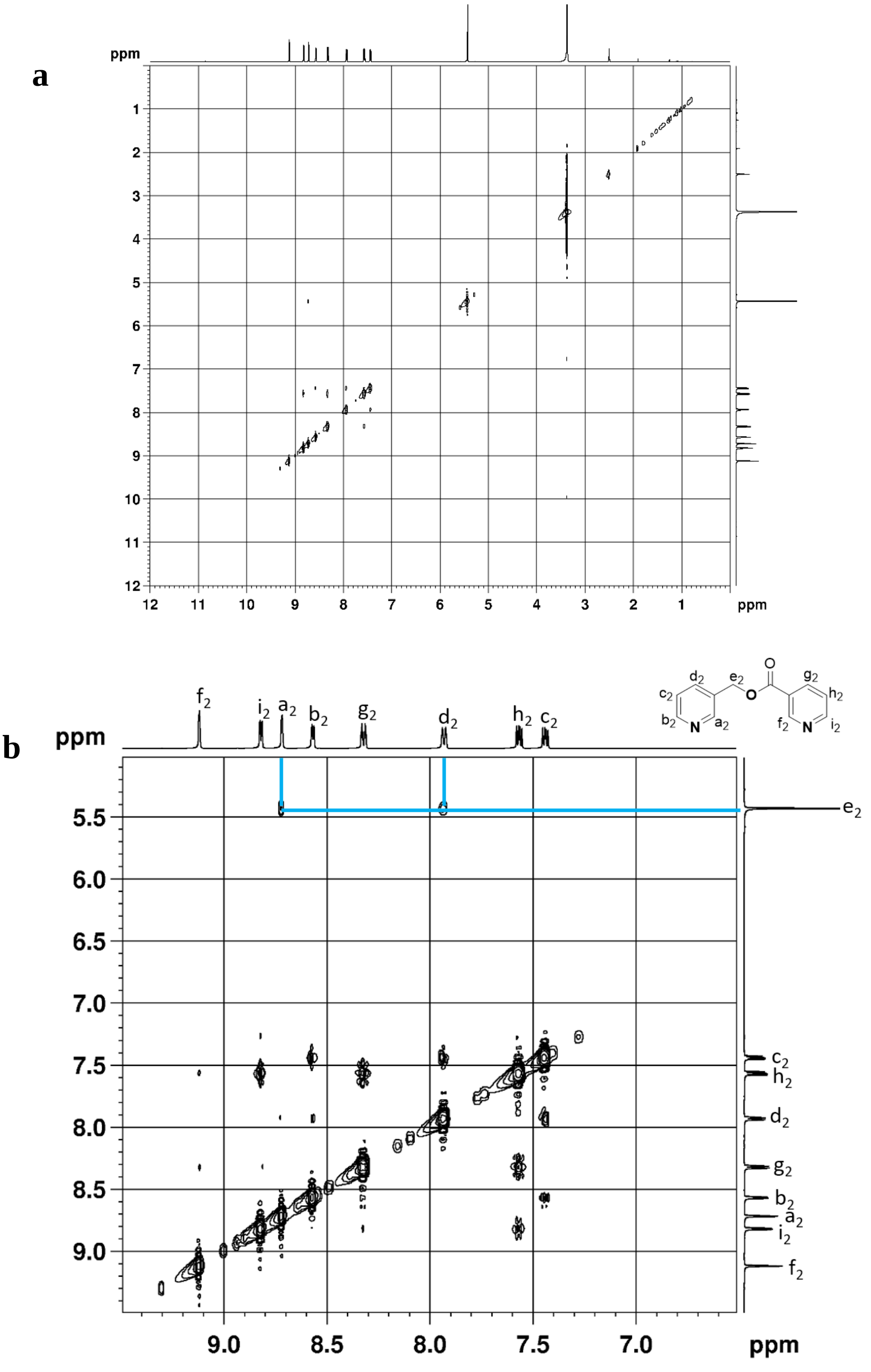

Supplementary Figure 5. NOESY spectrum (500 MHz, DMSO-d_6, 300 K) for **a** the ligand **L2**, and **b** expansion of the spectrum.

Compound Details

Cpd. 1: C12 H10 N2 O2

Compound Spectra (overlaid)

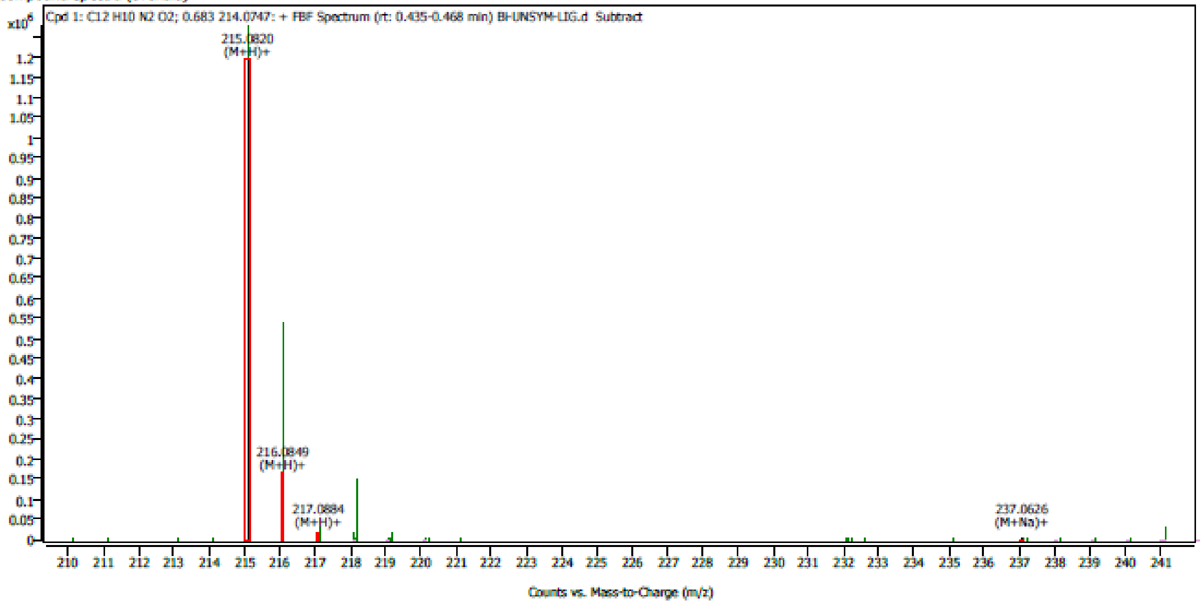

Compound ID Table

Cpd	Formula	Mass (Tgt)	Calc. Mass	Mass	Species	Diff(Tgt.ppm)	mDa
1	C12 H10 N2 O2	214.0742	214.0747	215.0820<br>237.0626	(M+H)+<br>(M+Na)+	2.28	0.49

Supplementary Figure 6. ESI mass spectrum for the ligand **L2**.

Characterisation of [NO3⊂Pd2(L2)4](NO3)3, 2a mixture of isomers

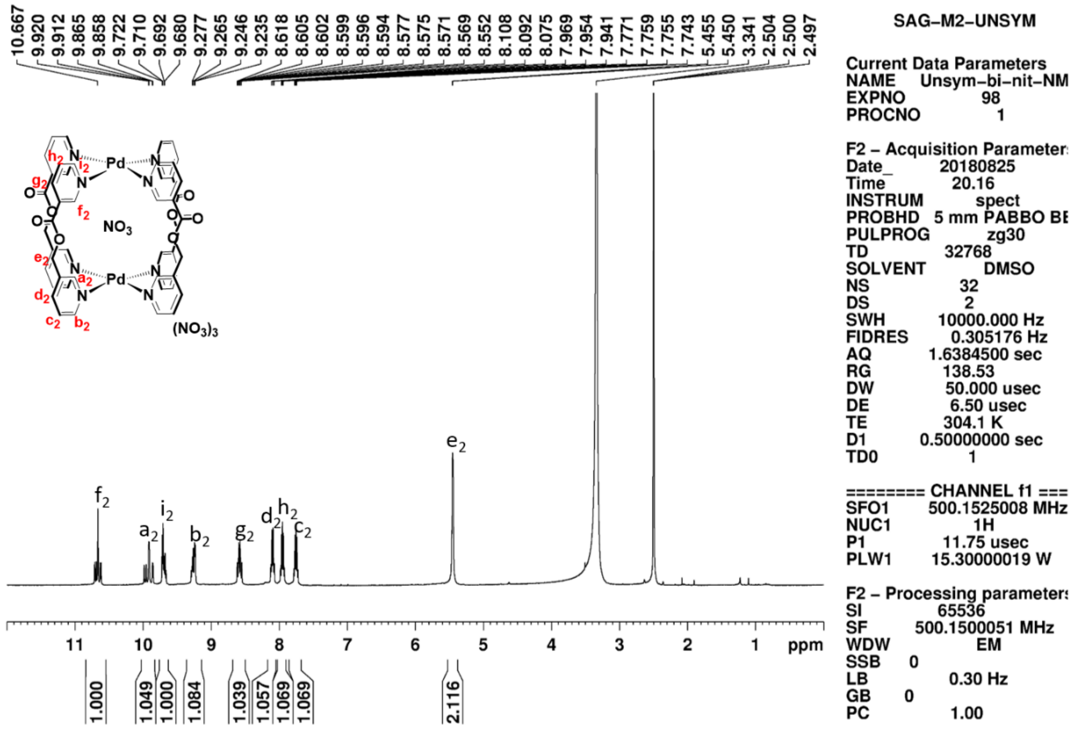

Supplementary Figure 7. ^{1}H NMR spectrum (500 MHz, DMSO-d_6, 300 K) for mixture of diasteromers of the complex **2a**. (Concentration: 10 mM with respect to palladium(II) source).

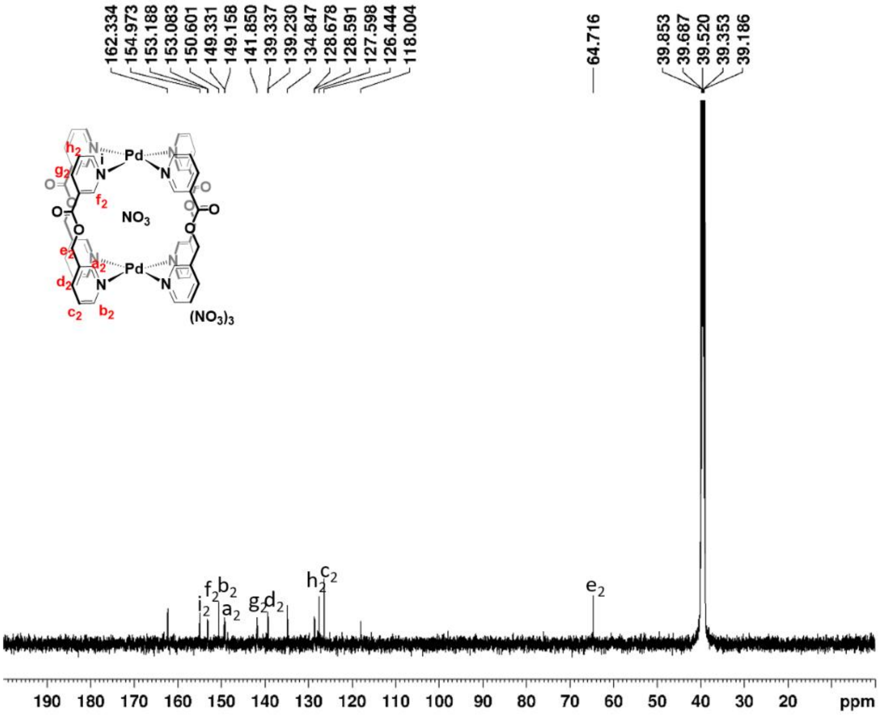

Supplementary Figure 8. ^{13}C NMR spectrum (125 MHz, DMSO-d_6, 300 K) for mixture of diastereomers of the complex **2a**.

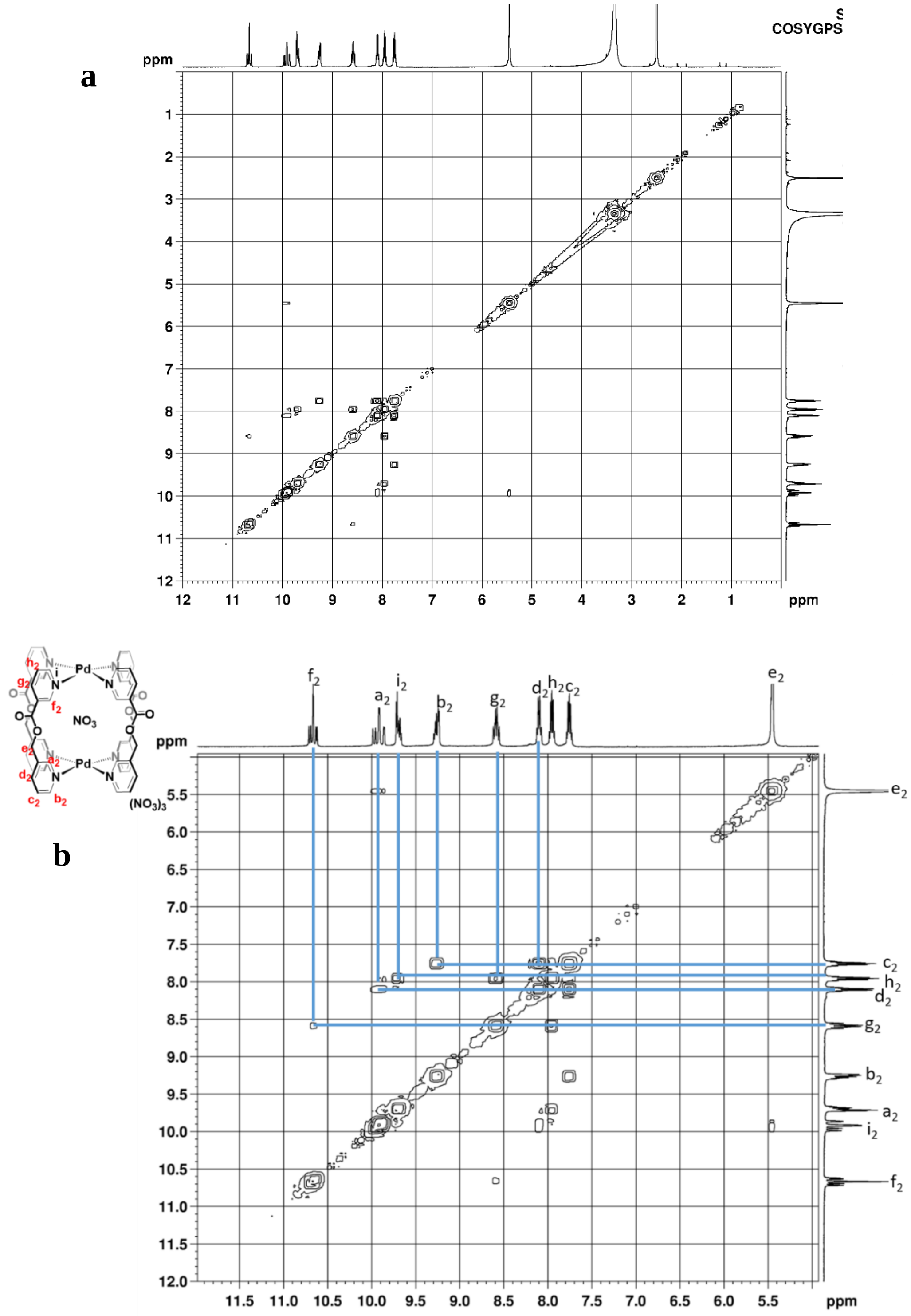

Supplementary Figure 9. H-H COSY spectrum (500 MHz, DMSO-d_6, 300 K) for **a** mixture of diastereomers of **2a**, and **b** expansion of the spectrum.

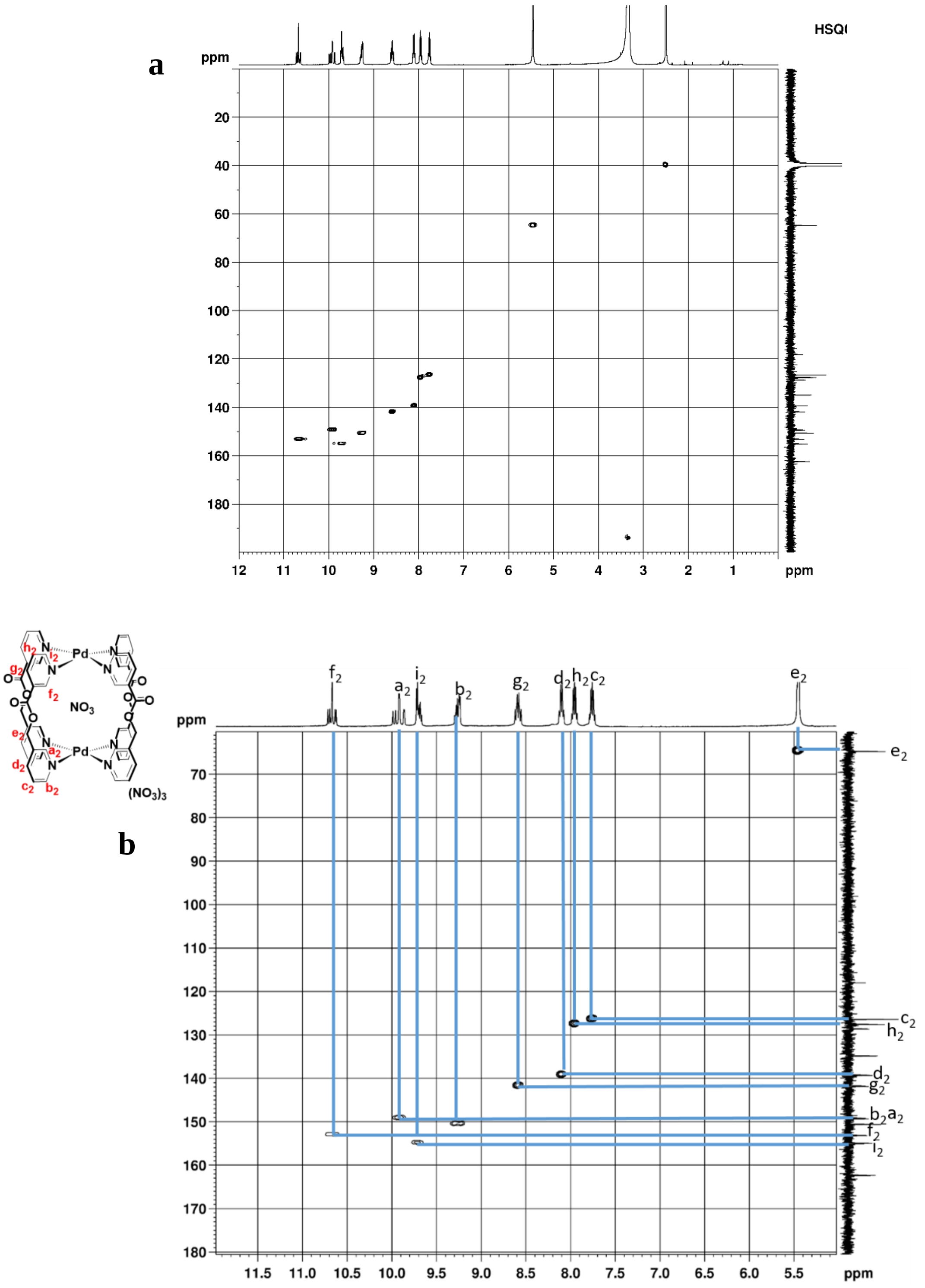

Supplementary Figure 10. C-H COSY spectrum (500 MHz, DMSO-d_6, 300 K) for **a** mixture of diastereomers of the complex **2a**, and **b** expansion of the spectrum.

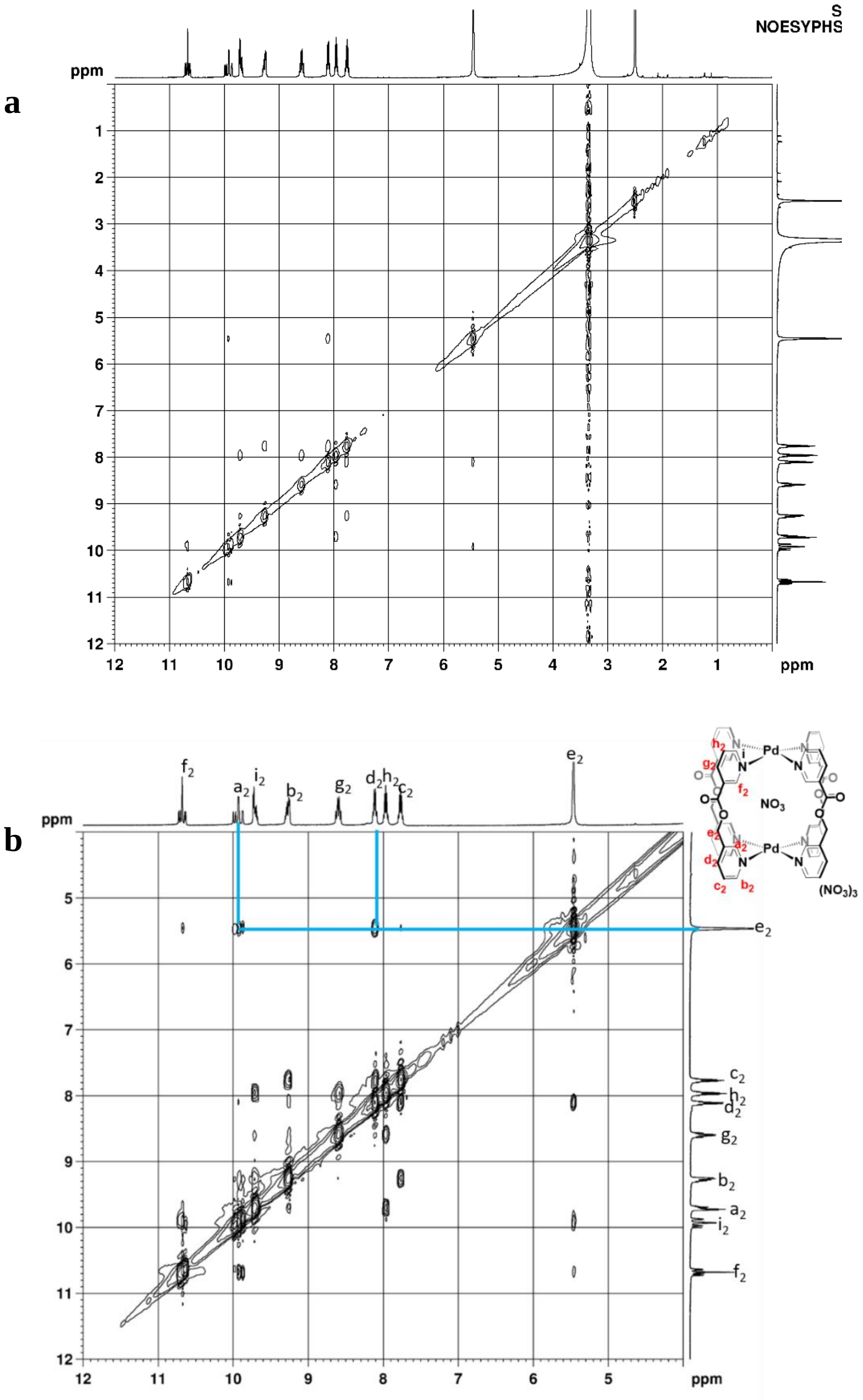

Supplementary Figure 11. NOESY spectrum (500 MHz, DMSO-d_6, 300 K) for **a** mixture of diastereomers of the complex **2a**, and **b** expansion of the spectrum.

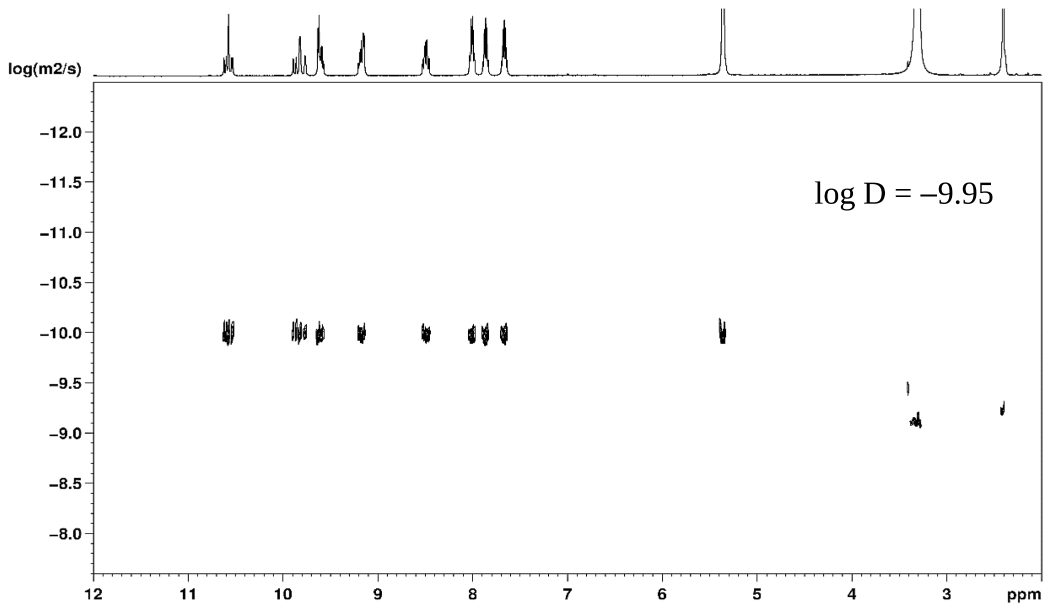

Supplementary Figure 12. DOSY NMR spectrum for mixture of diastereomers of the complex **2a** showing single band with diffusion co-efficient (D) value of 1.12 x 10^{-10} m^2 s^{-1}.

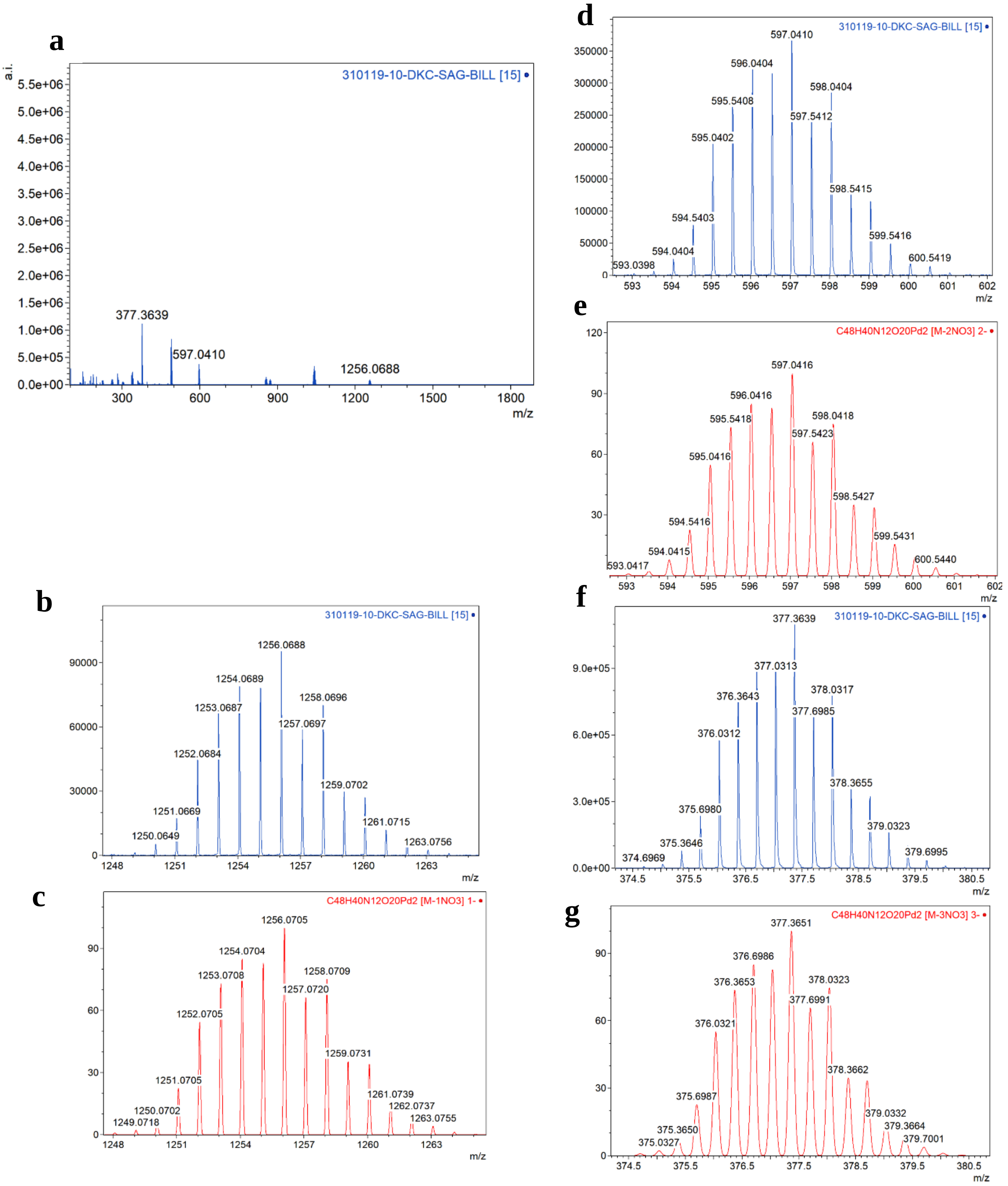

Supplementary Figure 13. ESI mass spectrum for $[NO_3\subset Pd_2(\mathbf{L2})_4](NO_3)_3$, **2a** showing **a** full spectrum, **b** experimental isotopic pattern for $[\mathbf{2a}{-}1NO_3]^{1+}$, **c** theoretical isotopic pattern for $[\mathbf{2a}{-}1NO_3]^{1+}$, **d** experimental isotopic pattern for $[\mathbf{2a}{-}2NO_3]^{2+}$, **e** theoretical isotopic pattern for $[\mathbf{2a}{-}2NO_3]^{2+}$, **f** experimental isotopic pattern for $[\mathbf{2a}{-}3NO_3]^{3+}$, and **g** theoretical isotopic pattern for $[\mathbf{2a}{-}3NO_3]^{3+}$.

Characterization of mixture of isomers $[X\subset Pd_2(L2)_4](NO_3)_3$, 2b-2d

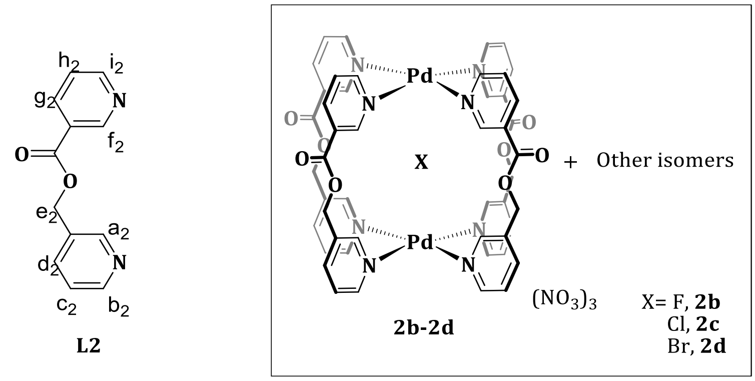

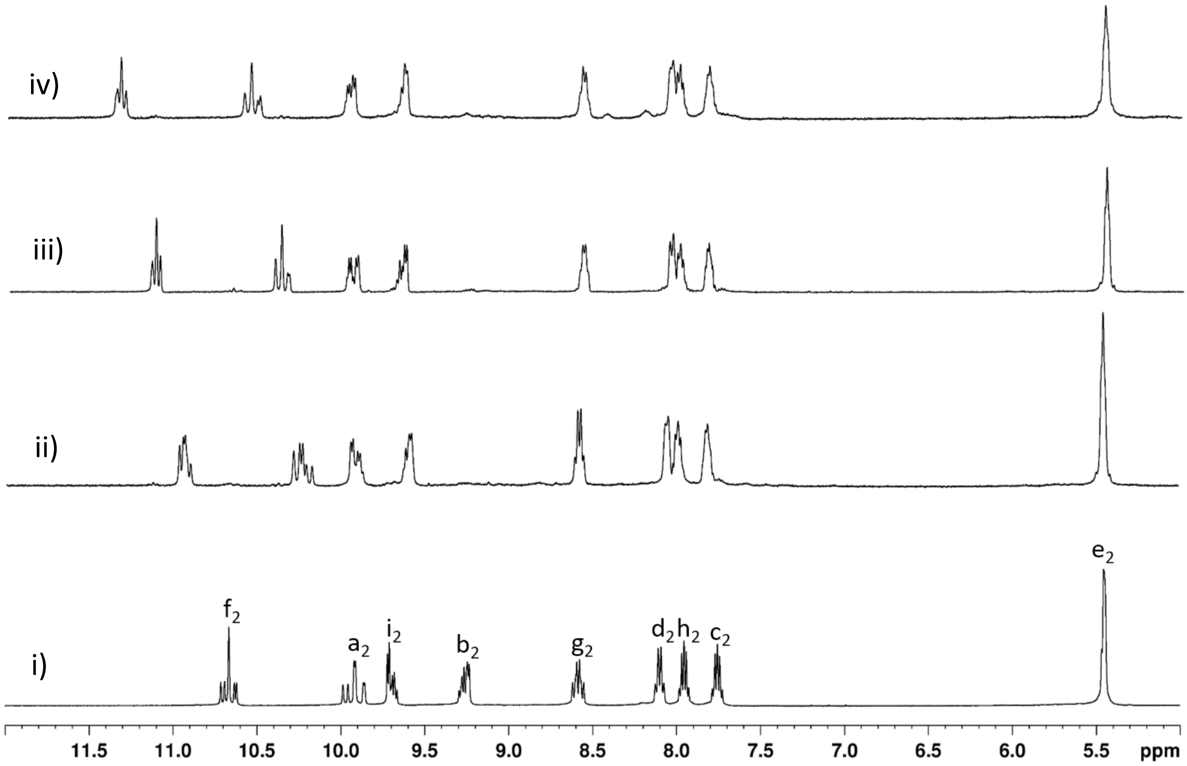

Supplementary Figure 14. Partial ^{1}H NMR spectra (400 MHz, DMSO-d_6, 300 K) for (i) $[NO_3\subset Pd_2(\mathbf{L2})_4](NO_3)_3$, **2a**; after addition of 1 equivalent of appropriate TBAX tetra-butylammonium salts forming (ii) $[F\subset Pd_2(\mathbf{L2})_4](NO_3)_3$, **2b** (when X= F); (iii) $[Cl\subset Pd_2(\mathbf{L2})_4](NO_3)_3$, **2c** (when X= Cl) and (iv) $[Br\subset Pd_2(\mathbf{L2})_4](NO_3)_3$, **2d** (when X= Br).

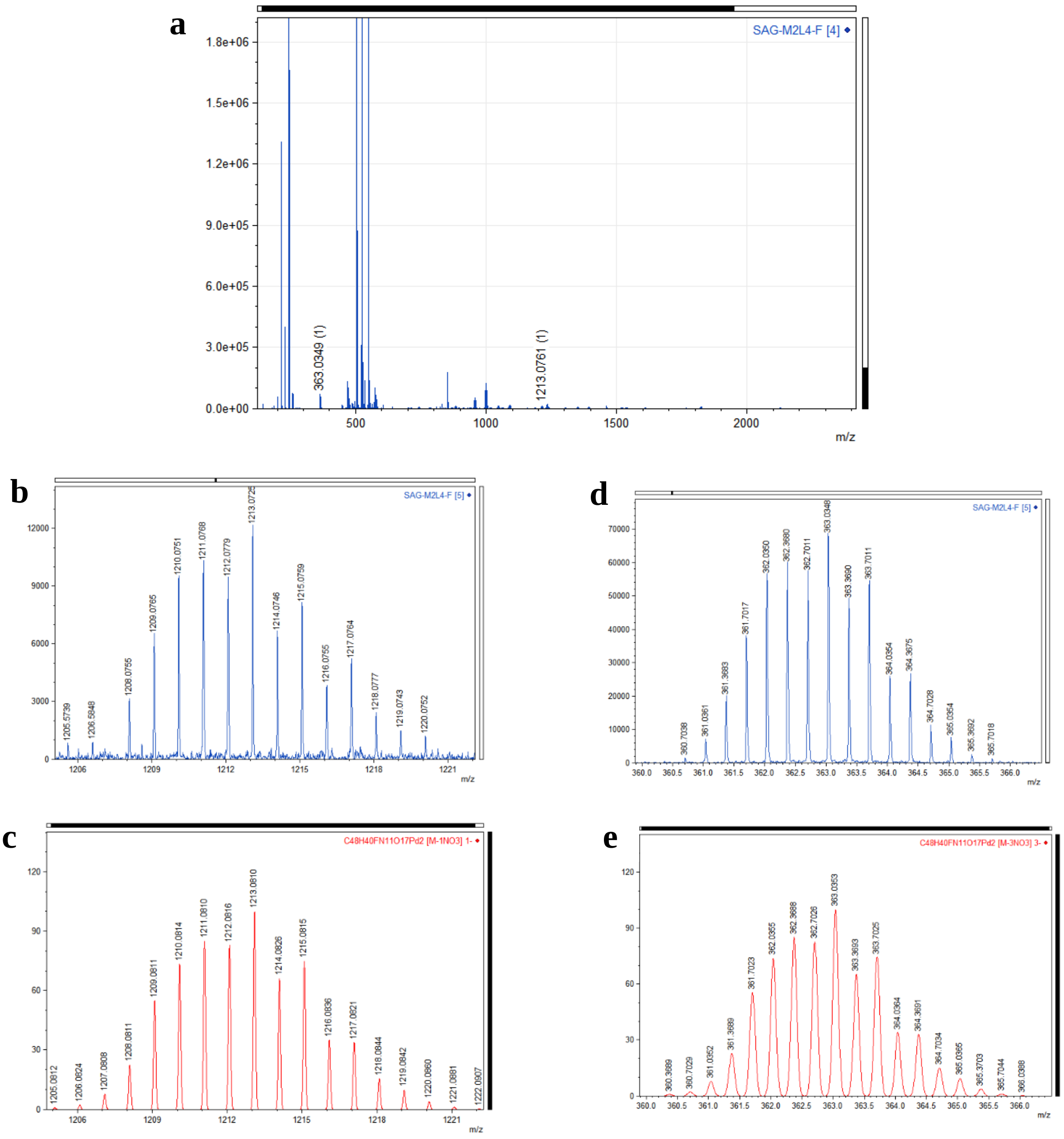

Supplementary Figure 15. ESI mass spectrum for [F⊂Pd_2(**L2**)$_4$](NO_3)$_3$, **2b** showing **a** full spectrum, **b** experimental isotopic pattern for [**2b**–1NO_3]$^{1+}$, **c** theoretical isotopic pattern for [**2b**–1NO_3]$^{1+}$, **d** experimental isotopic pattern for [**2b**–3NO_3]$^{3+}$, and **e** theoretical isotopic pattern for [**2b**–3NO_3]$^{3+}$.

Characterization of mixture of isomers [X⊂Pd_2(L2)$_4$](BF_4)$_3$, 2a'-2d'

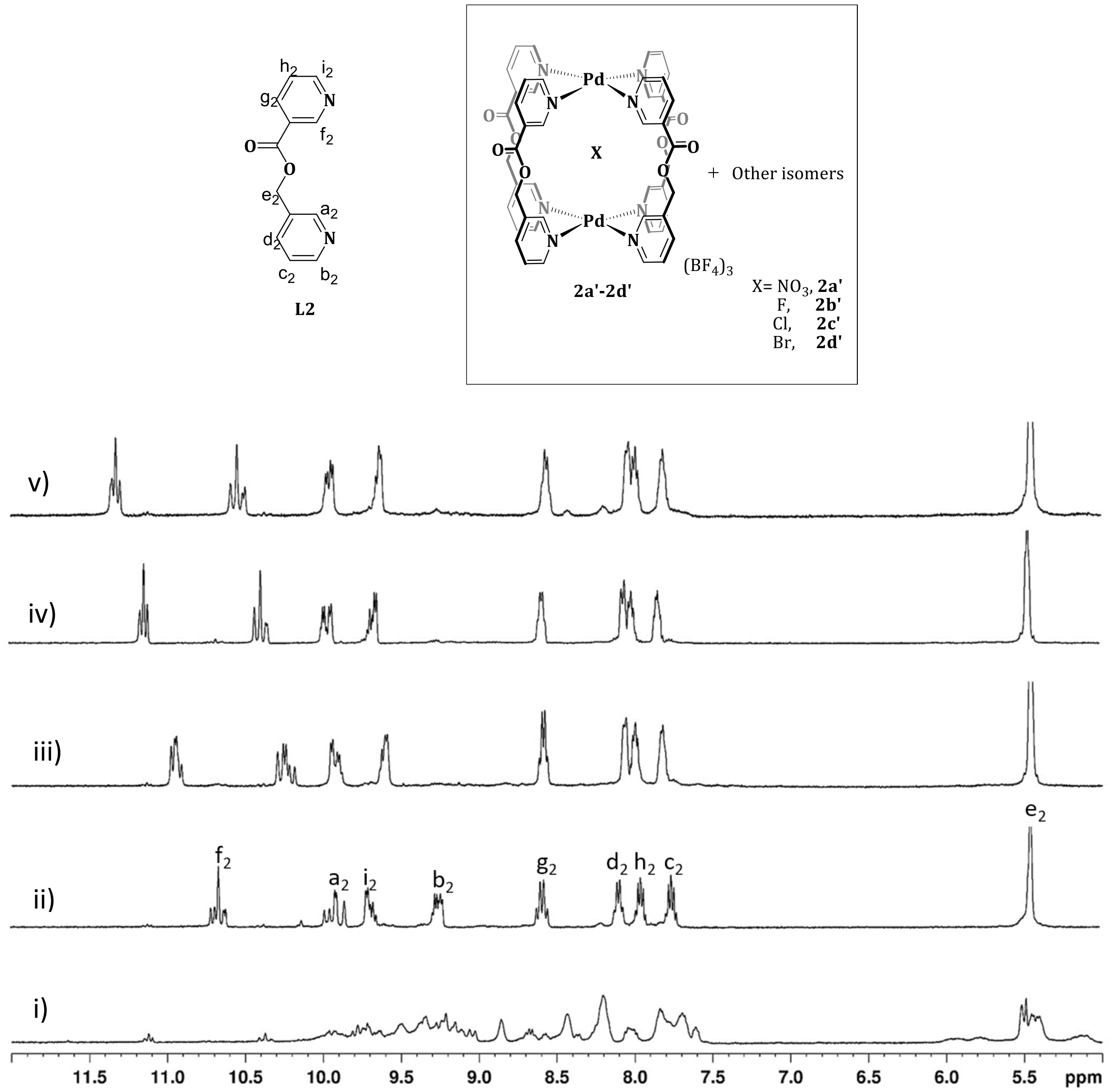

Supplementary Figure 16. Partial ^{1}H NMR spectra (400 MHz, DMSO-d_6, 300 K) for (i) Oligomers formed due to complexation of Pd(BF_4)$_2$ with **L2** at 2:4 ratio; upon addition of 1 equivalent of (ii) TBANO_3 forming [NO_3⊂Pd_2(**L2**)$_4$](BF_4)$_3$, **2a'**; (iii) TBAF forming [F⊂Pd_2(**L2**)$_4$](BF_4)$_3$, **2b'**; (iv) TBACl forming [Cl⊂Pd_2(**L2**)$_4$](BF_4)$_3$, **2c'** and (v) TBABr forming [Br⊂Pd_2(**L2**)$_4$](BF_4)$_3$, **2d'**.

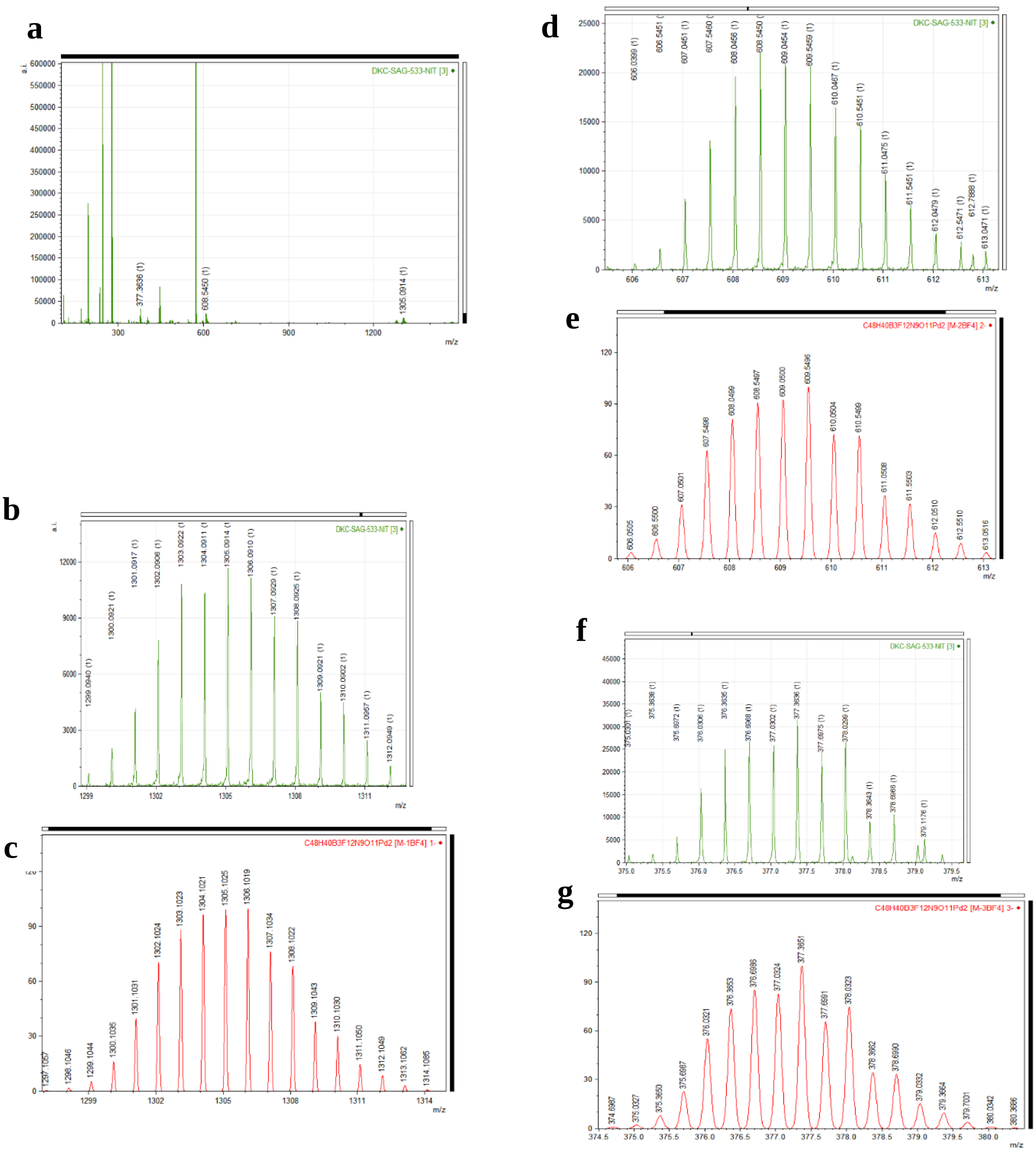

Supplementary Figure 17. ESI mass spectrum for [NO_3⊂Pd_2(**L2**)$_4$](BF_4)$_3$, **2a'**showing **a** full spectrum, **b** experimental isotopic pattern for [**2a'**−1BF_4]$^{1+}$, **c** theoretical isotopic pattern for [**2a'**−1BF_4]$^{1+}$, **d** experimental isotopic pattern for [**2a'**−2BF_4]$^{2+}$, **e** theoretical isotopic pattern for [**2a'**−2BF_4]$^{2+}$, **f** experimental isotopic pattern for [**2a'**−3BF_4]$^{3+}$, and **g** theoretical isotopic pattern for [**2a'**−3BF_4]$^{3+}$.

Characterisation of the ligand L3

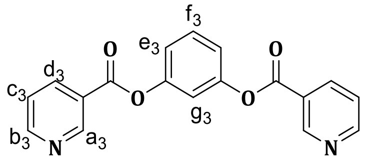

L3

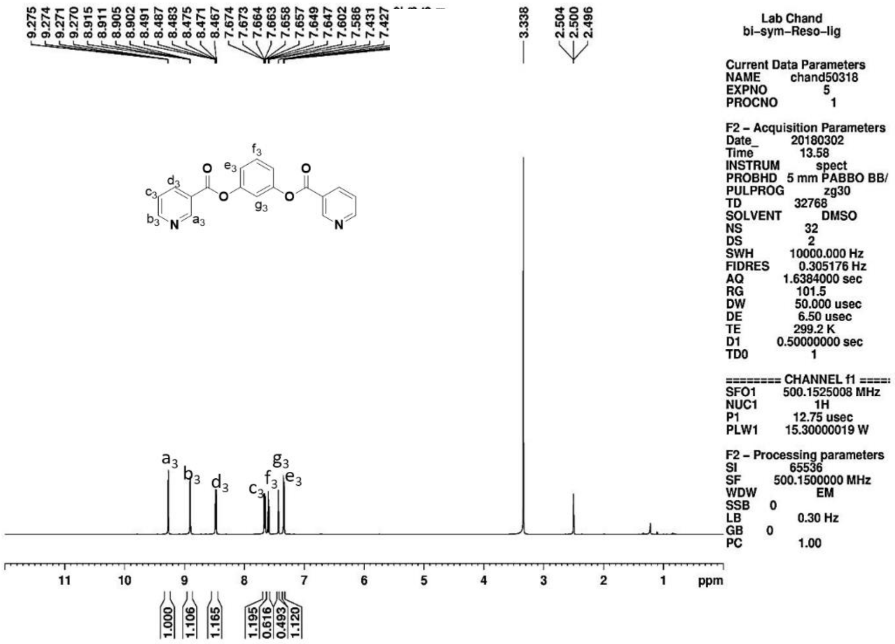

Supplementary Figure 18. ^{1}H NMR spectrum (500 MHz, DMSO-d_6, 300 K) for the ligand **L3**.

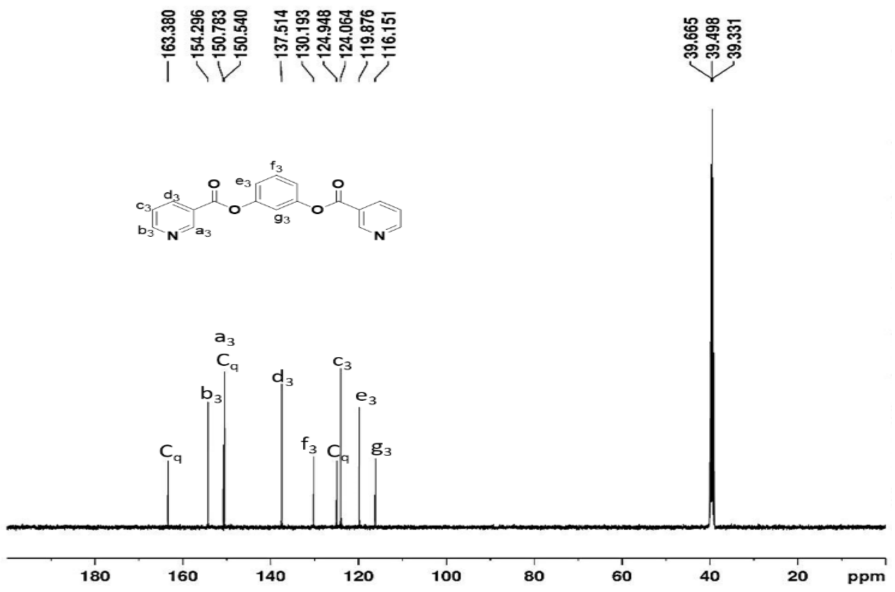

Supplementary Figure 19. ^{13}C NMR spectrum (125 MHz, DMSO-d_6, 300 K) for the ligand **L3**.

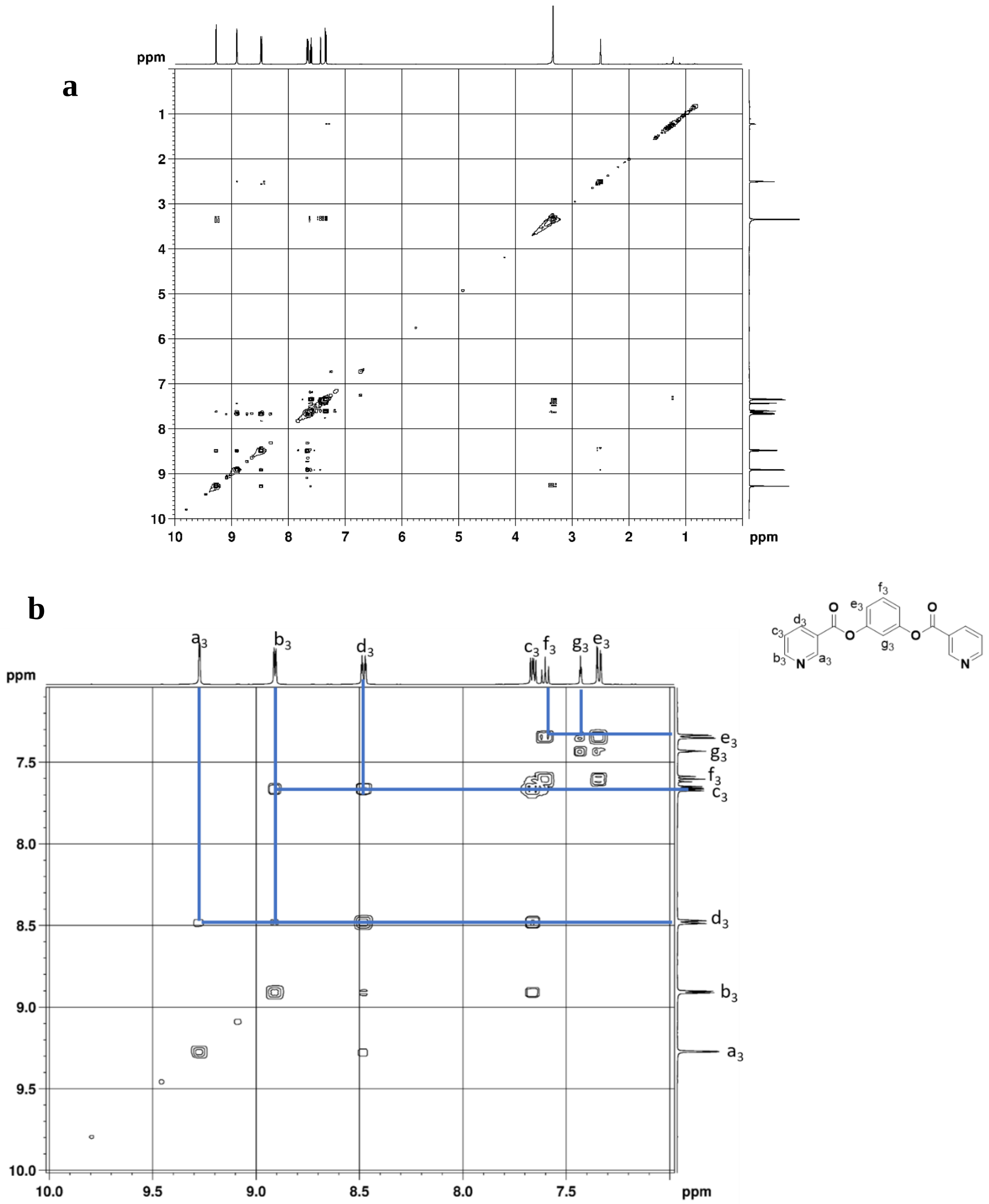

Supplementary Figure 20. H-H COSY spectrum (500 MHz, DMSO-d_6, 300 K) for **a** the ligand **L3**, and **b** expansion of the spectrum.

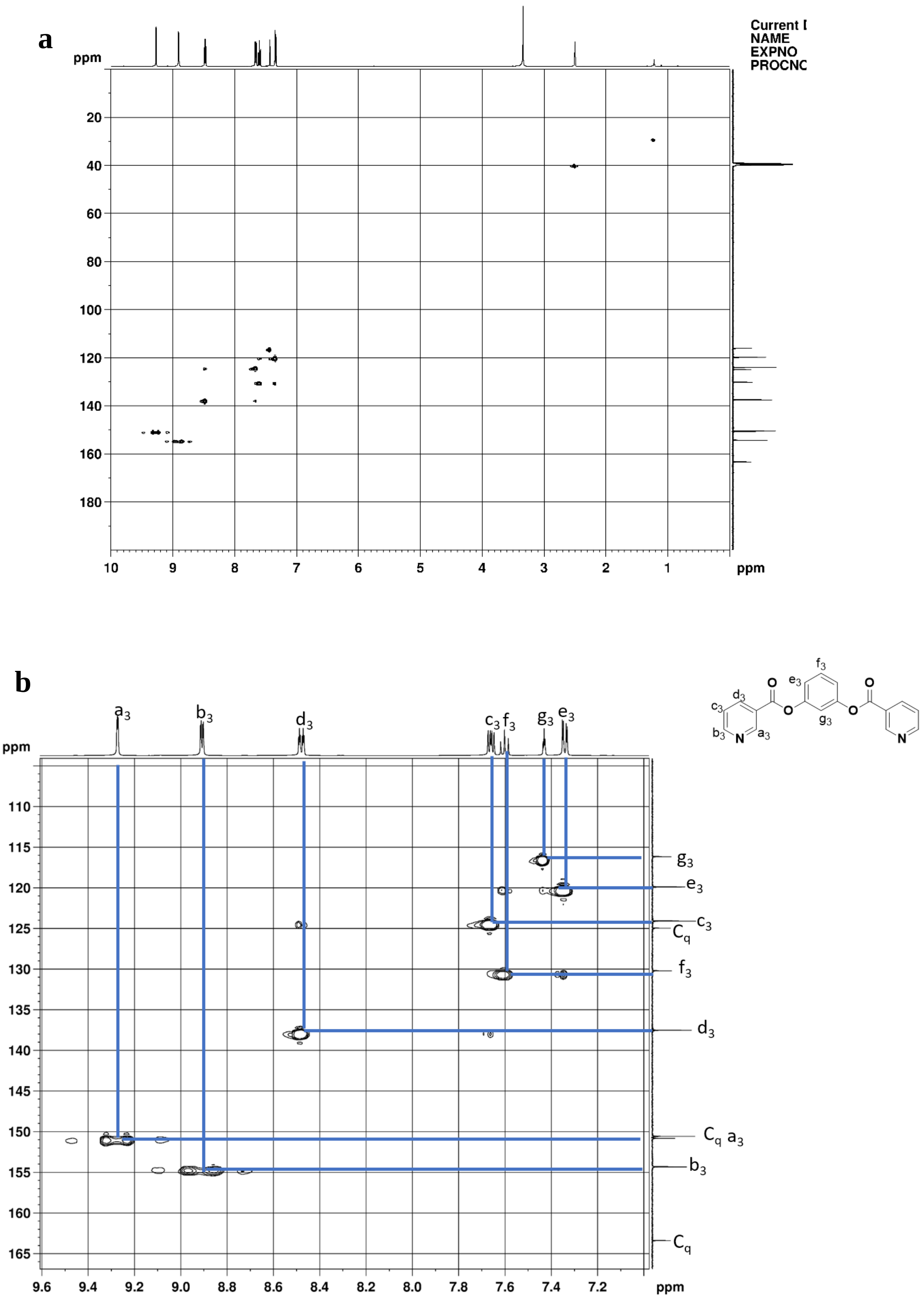

Supplementary Figure 21. C-H COSY spectrum (500 MHz, DMSO-d_6, 300 K) for **a** the ligand **L3**, and **b** expansion of the spectrum.

Compound Details

Cpd. 1: C18 H12 N2 O4

Compound Spectra (overlaid)

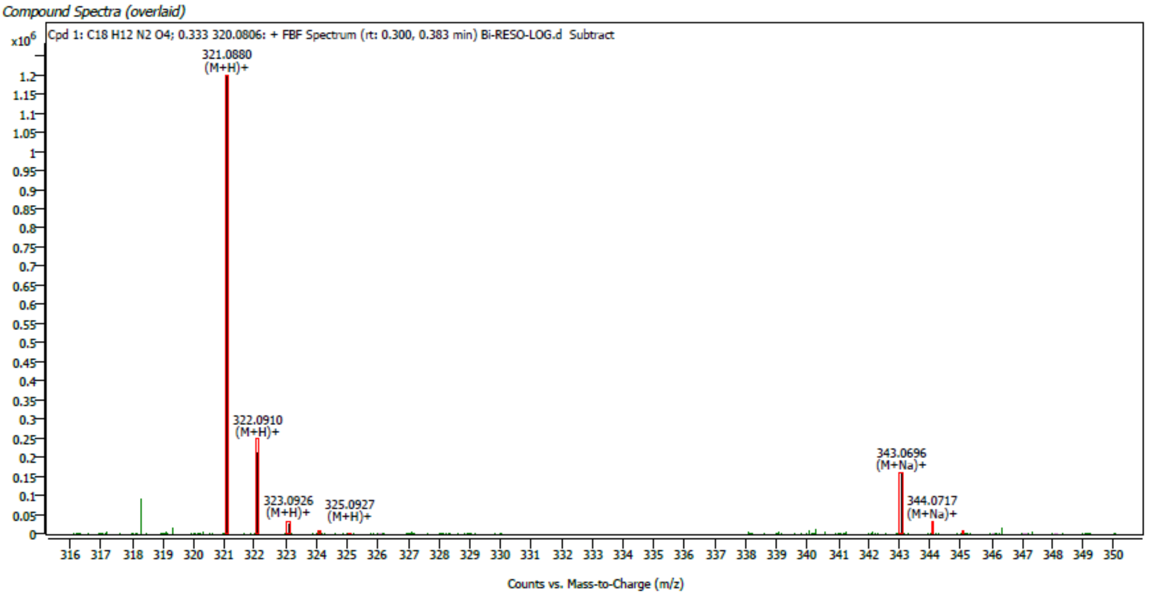

Compound ID Table

Cpd	Formula	Mass (Tgt)	Calc. Mass	Mass	Species	Diff(Tgt.ppm)	mDa
1	C18 H12 N2 O4	320.0797	320.0806	321.0880<br>343.0696	(M+H)+<br>(M+Na)+	2.88	0.92

Supplementary Figure 22. ESI mass spectrum for the ligand **L3**.

Characterisation of the complex [$Pd_3(L3)_6](NO_3)_6$, 3a

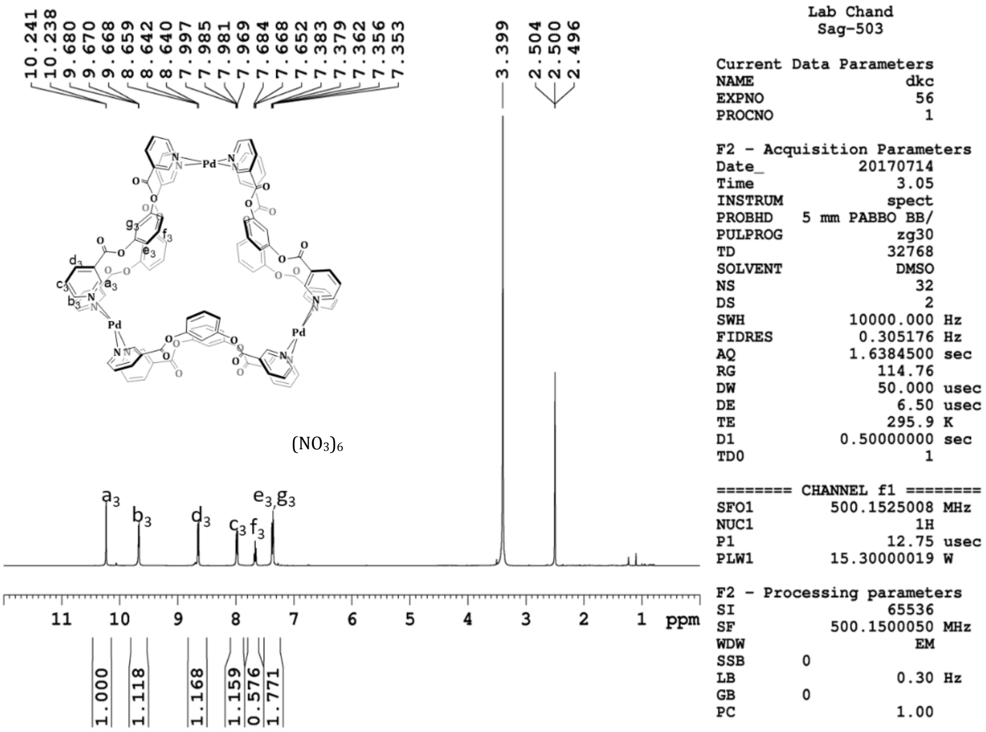

Supplementary Figure 23. ^{1}H NMR spectrum (500 MHz, DMSO-d_6, 300 K) for the complex **3a**. (Concentration: 10 mM with respect to palladium(II) source).

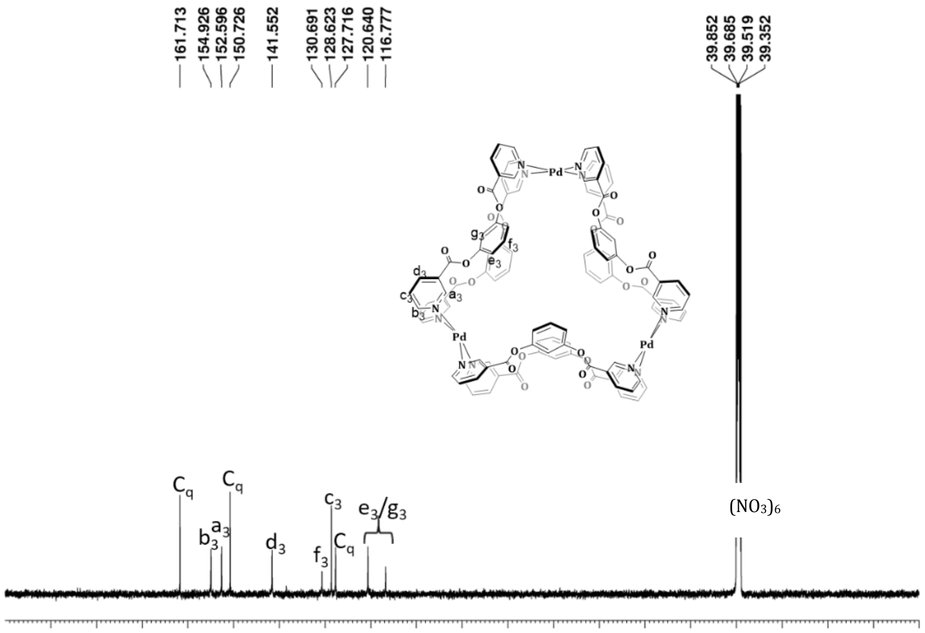

Supplementary Figure 24. ^{13}C NMR spectrum (125 MHz, DMSO-d_6, 300 K) for the complex **3a**.

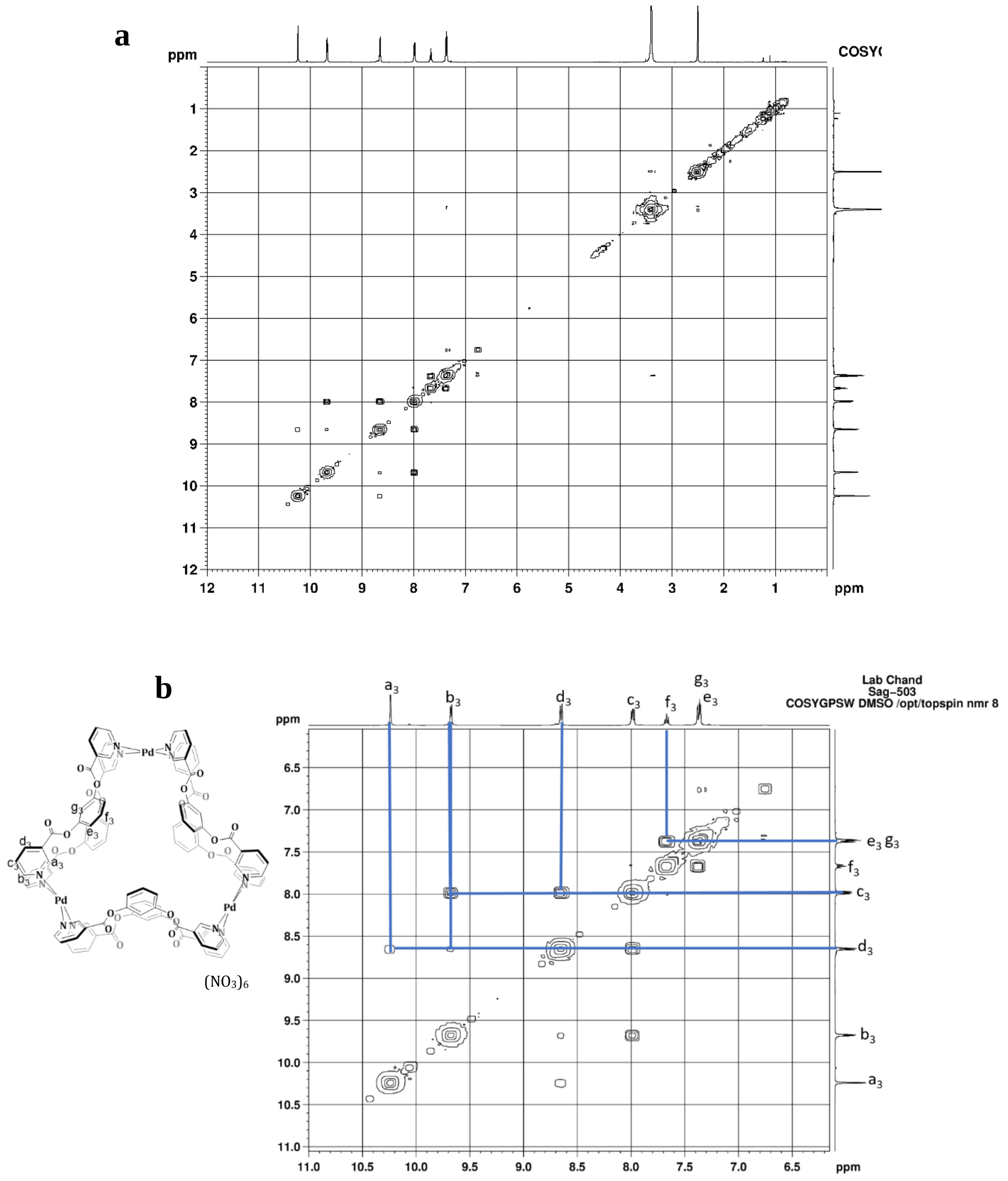

Supplementary Figure 25. H-H COSY spectrum (500 MHz, DMSO-d_6, 300 K) for **a** the complex **3a**, and **b** expansion of the spectrum.

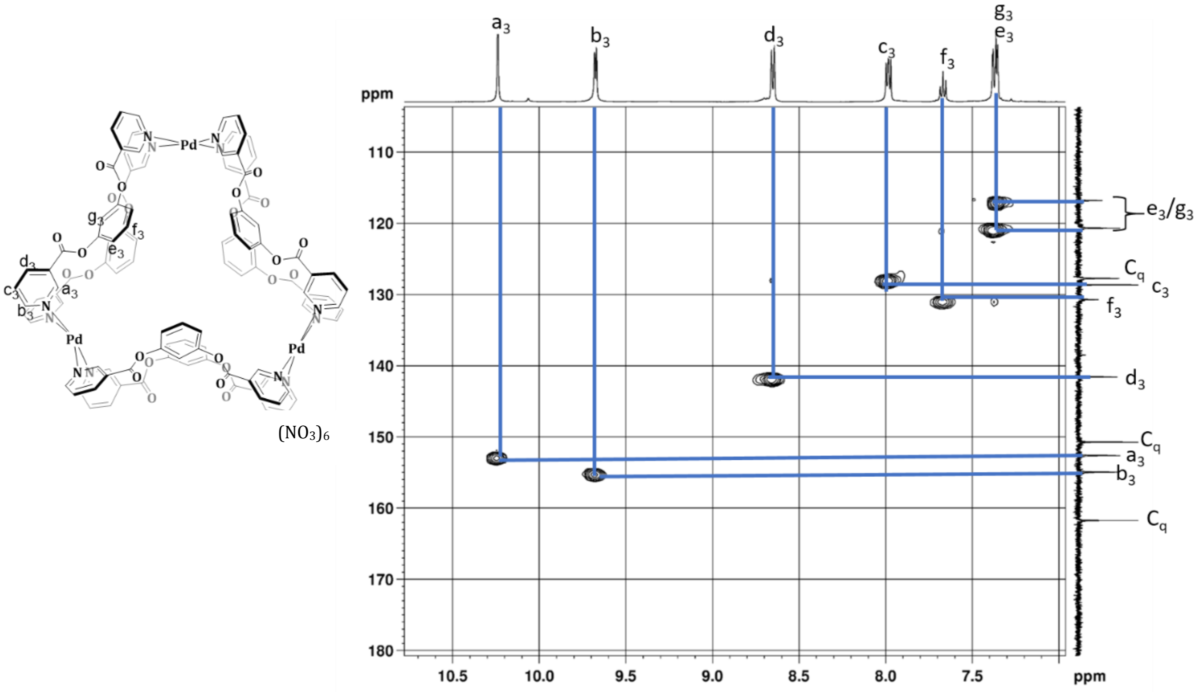

Supplementary Figure 26. C-H COSY spectrum (500 MHz, DMSO-d_6, 300 K) expanded for the complex **3a**.

.

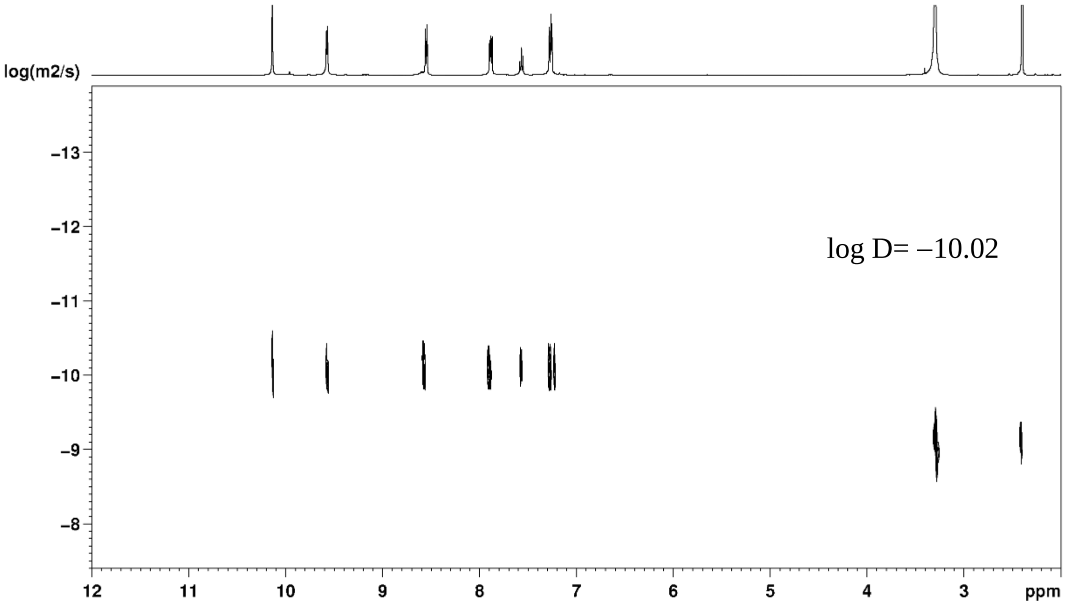

Supplementary Figure 27. ^{1}H DOSY NMR spectrum for complex $[Pd_3(\mathbf{L3})_6](NO_3)_6$, **3a** showing single band with diffusion co-efficient (D) value of 9.54 x 10^{-11} m^2 s^{-1}.

Counter anion variation study for the complexation of palladium(II) with L3: shows No influence of anions.

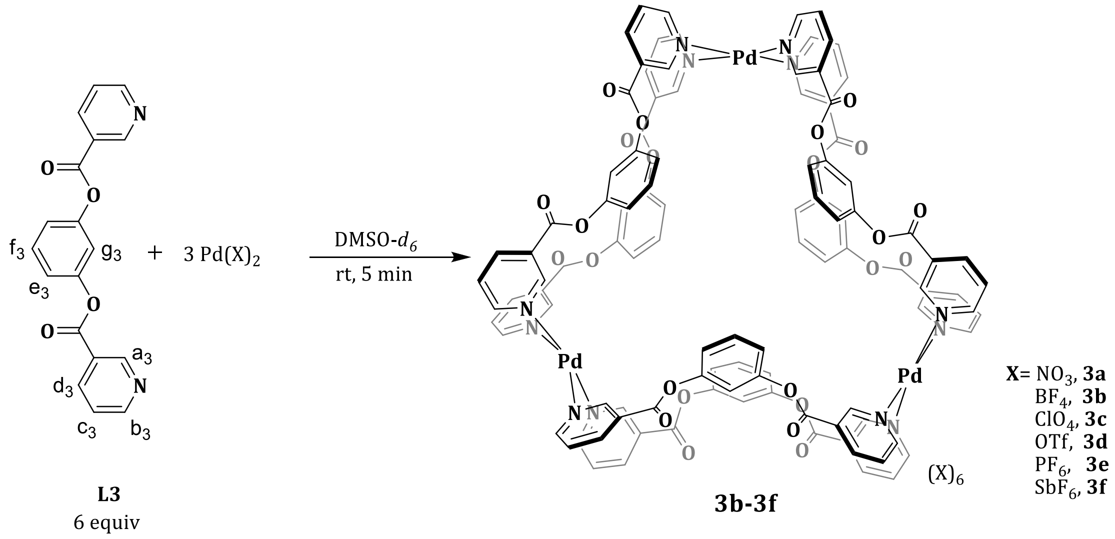

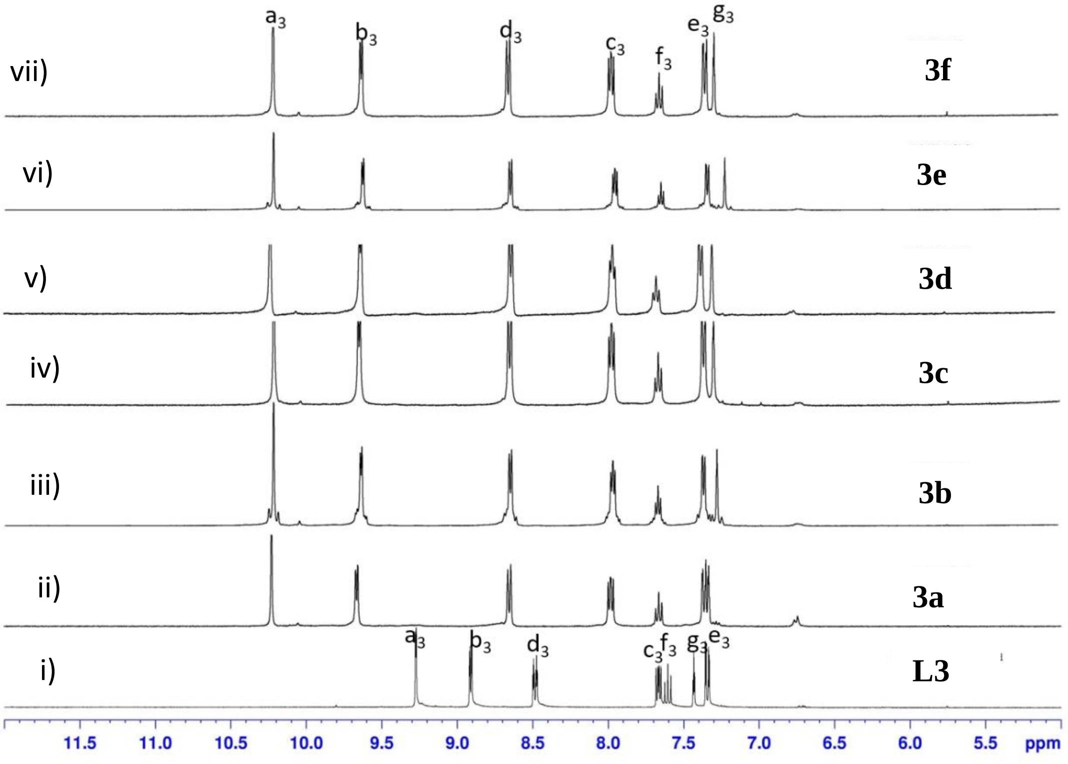

Supplementary Figure 28. Partial ^{1}H NMR spectra (400 MHz, DMSO-d_6, 300 K) for (i) ligand **L3**; (ii) $[Pd_3(\mathbf{L3})_6](NO_3)_6$, **3a** (iii) $[Pd_3(\mathbf{L3})_6](BF_4)_6$, **3b** (iv) $[Pd_3(\mathbf{L3})_6](ClO_4)_6$, **3c** (v) $[Pd_3(\mathbf{L3})_6](OTf)_6$, **3d** (vi) $[Pd_3(\mathbf{L3})_6](PF_6)_6$, **3e** and (vii) $[Pd_3(\mathbf{L3})_6](SbF_6)_6$, **3f**.

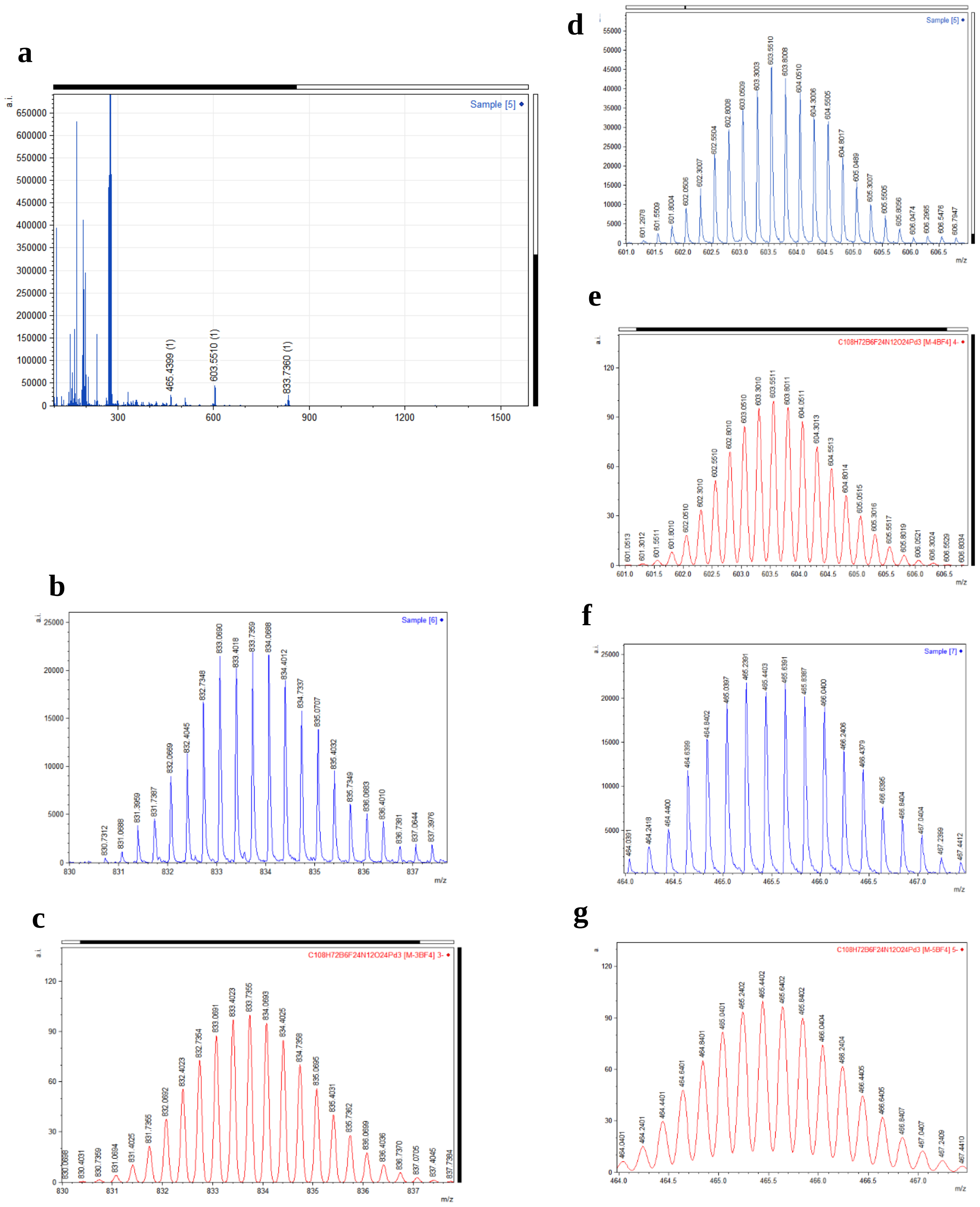

Supplementary Figure 29. ESI mass spectrum for $[Pd_3(\mathbf{L3})_6](BF_4)_6$, **3b** showing **a** full spectrum, **b** experimental isotopic pattern for $[\mathbf{3b}{-}3BF_4]^{3+}$, **c** theoretical isotopic pattern for $[\mathbf{3b}{-}3BF_4]^{3+}$, **d** experimental isotopic pattern for $[\mathbf{3b}{-}4BF_4]^{4+}$, **e** theoretical isotopic pattern for $[\mathbf{3b}{-}4BF_4]^{4+}$, **f** experimental isotopic pattern for $[\mathbf{3b}{-}5BF_4]^{5+}$, and **g** theoretical isotopic pattern for $[\mathbf{3b}{-}5BF_4]^{5+}$.

Concentration variation study for the complexation of palladium(II) with L3: Minor role of concentration

Supplementary Discussion 1. Minor proportion of other complexes coexisting with **3a** at lower/higher concentration were detected and identified. Along with the trinuclear complex **3a**, formation of negligible amount of binuclear, **3g** and tetranuclear, **3h** were detected at 1mM and 30 mM concentration of palladium(II) respectively. The percentages indicated are with respect to the proportion of ligand **L3**.

❖ **Concentration of palladium(II): 1 mM**

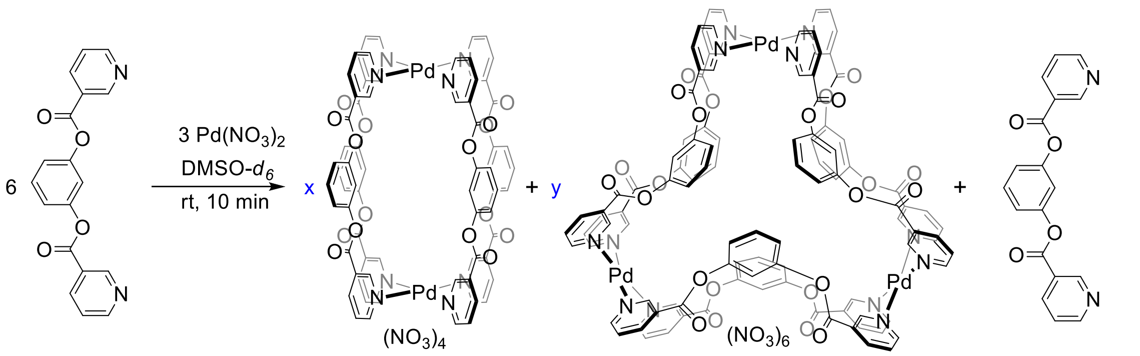

L3 | 3g (Minor: 7% of ligand) | 3a (Major: 74% of ligand) | L3 (19% of ligand)

❖ **Concentration of palladium(II): 30 mM**

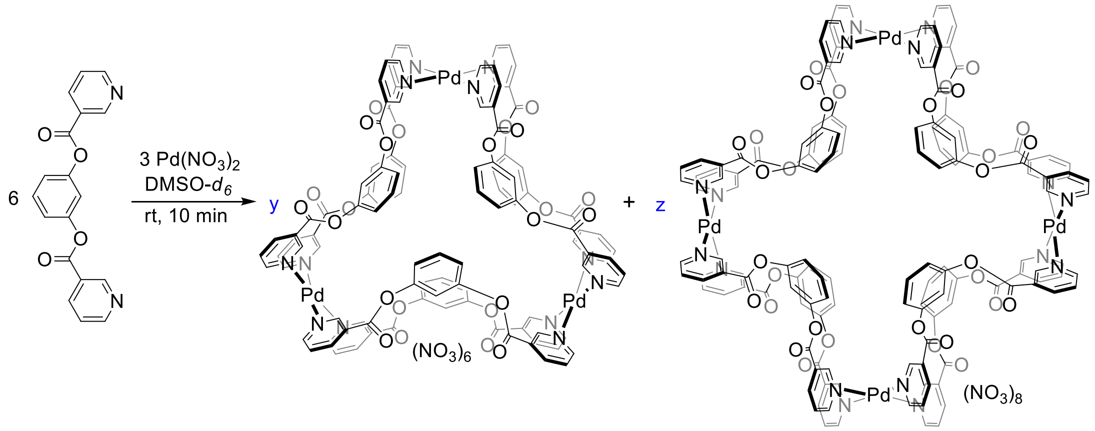

L3 | 3a (Major: 94% of ligand) | 3h (Minor: 6% of ligand)

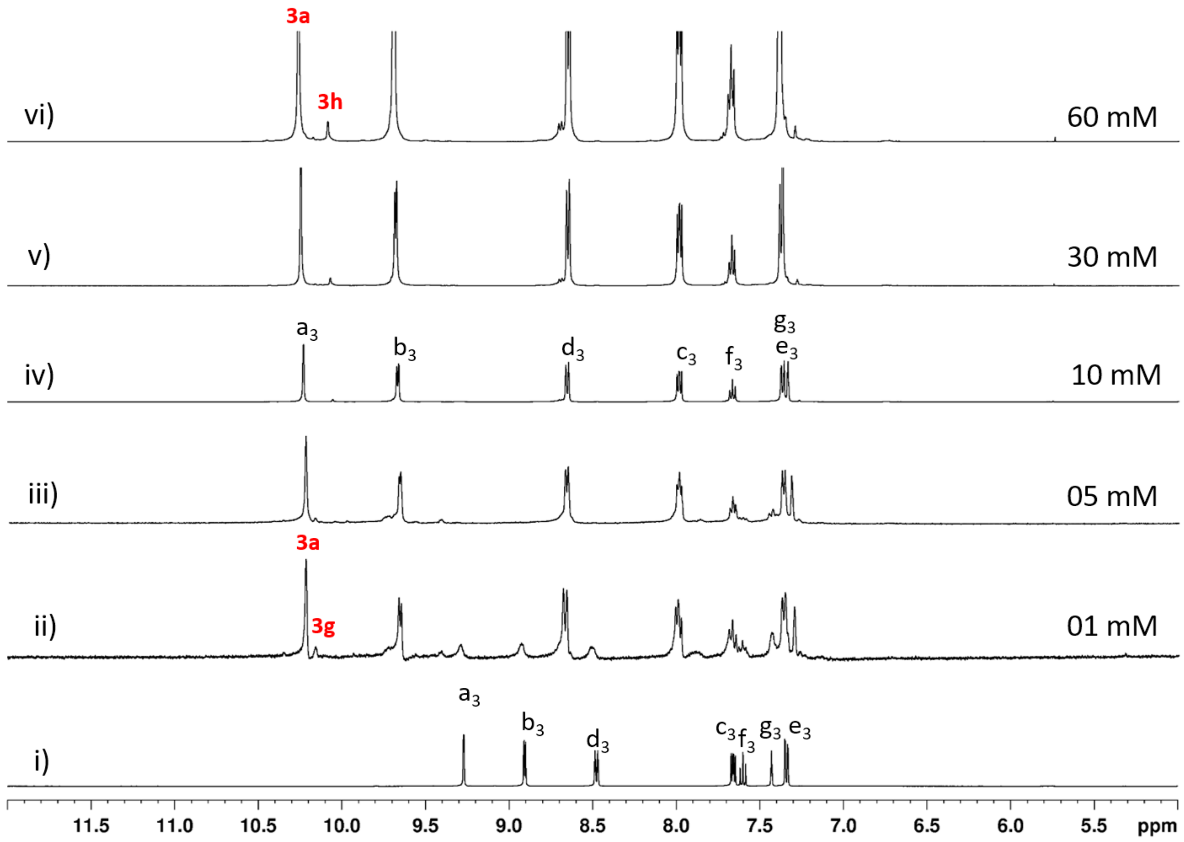

Supplementary Figure 30. Partial ^{1}H NMR spectra (400 MHz, DMSO-d_6, 300 K) for (i) ligand **L3**; [Pd_3(**L3**)$_6$](NO_3)$_6$, **3a** @ (ii) 1 mM ; (iii) 5 mM ; (iv) 10 mM; (v) 30 mM and (vi) 60 mM concentration with respect to $Pd(NO_3)_2$. [**3g** represents binuclear and **3h** represents tetranuclear species]

Supplementary Table 1. Approximate percentage of **L3** in the complexes **3g**, **3h** and **3a**.

Concentration of palladium(II)	Approximate percentage of ligand **L3**			
	[Pd_2(**L3**)$_4$](NO_3)$_4$, **3g**	[Pd_3(**L3**)$_6$](NO_3)$_6$, **3a**	[Pd_4(**L3**)$_8$](NO_3)$_8$, **3h**	Ligand **L3**
1 mM	7	74	-	19
5 mM	Negligible	100	-	-
10 mM	-	100	Negligible	-
30 mM	-	94	6	-
60 mM	-	93	7	-

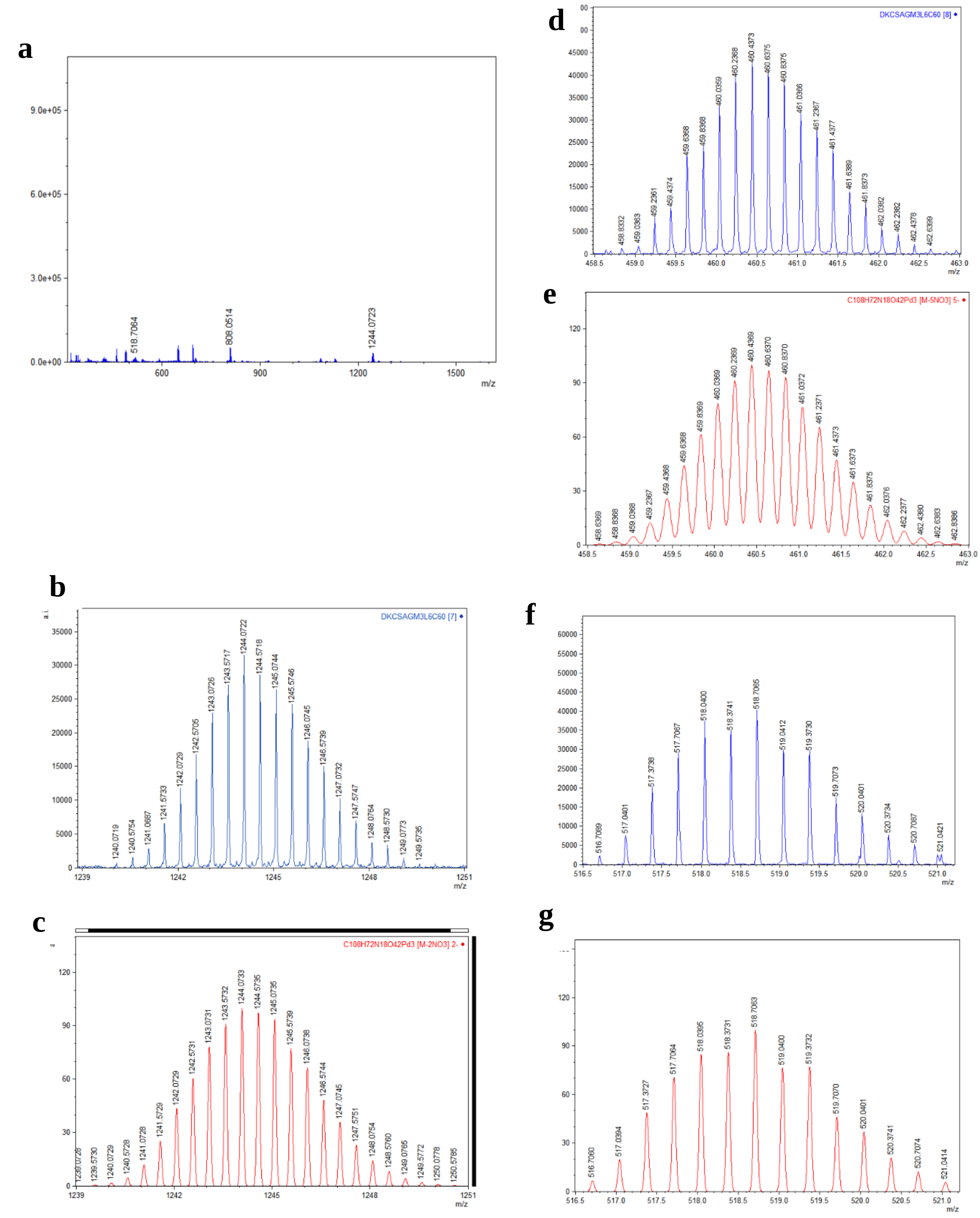

Supplementary Figure 31. ESI mass spectrum for a mixture of $[Pd_3(\mathbf{L3})_6](NO_3)_6$, **3a** and $[Pd_2(\mathbf{L3})_4](NO_3)_4$, **3g** showing **a** full spectrum, **b** experimental isotopic pattern for $[\mathbf{3a}–2NO_3]^{2+}$, **c** theoretical isotopic pattern for $[\mathbf{3a}–2NO_3]^{2+}$, **d** experimental isotopic pattern for $[\mathbf{3a}–5NO_3]^{5+}$, **e** theoretical isotopic pattern for $[\mathbf{3a}–5NO_3]^{5+}$, **f** experimental isotopic pattern for $[\mathbf{3g}–3NO_3]^{3+}$, and **g** theoretical isotopic pattern for $[\mathbf{3g}–3NO_3]^{3+}$.

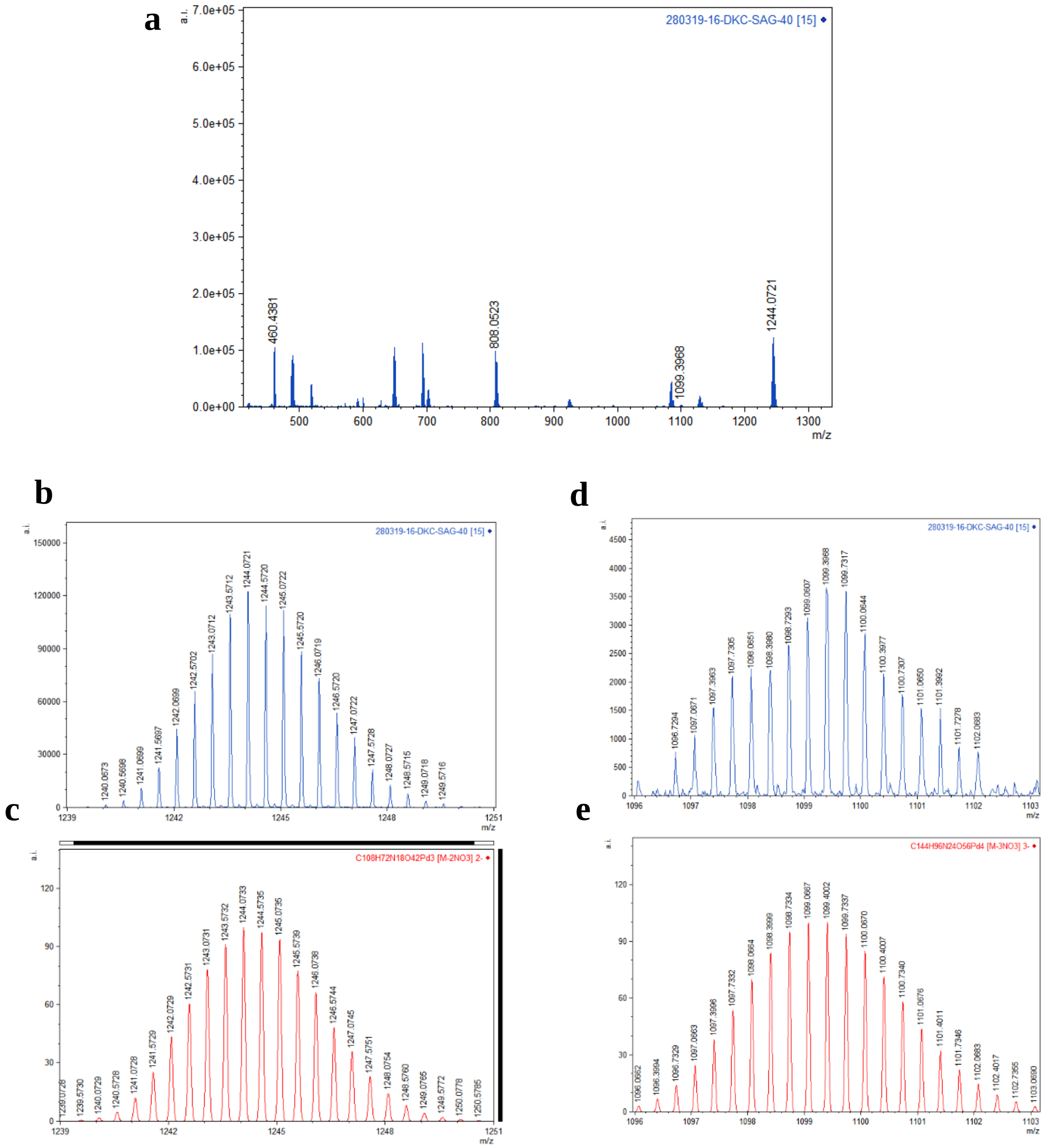

Supplementary Figure 32. ESI mass spectrum for a mixture of [Pd_3(**L3**)$_6$](NO_3)$_6$, **3a** and [Pd_4(**L3**)$_8$](NO_3)$_8$, **3h** showing **a** full spectrum, **b** experimental isotopic pattern for [**3a**–2NO_3]$^{2+}$, **c** theoretical isotopic pattern for [**3a**–2NO_3]$^{2+}$, **d** experimental isotopic pattern for [**3h**–3NO_3]$^{3+}$, and **e** theoretical isotopic pattern for [**3h**–3NO_3]$^{3+}$.

Characterisation of the ligand, L4

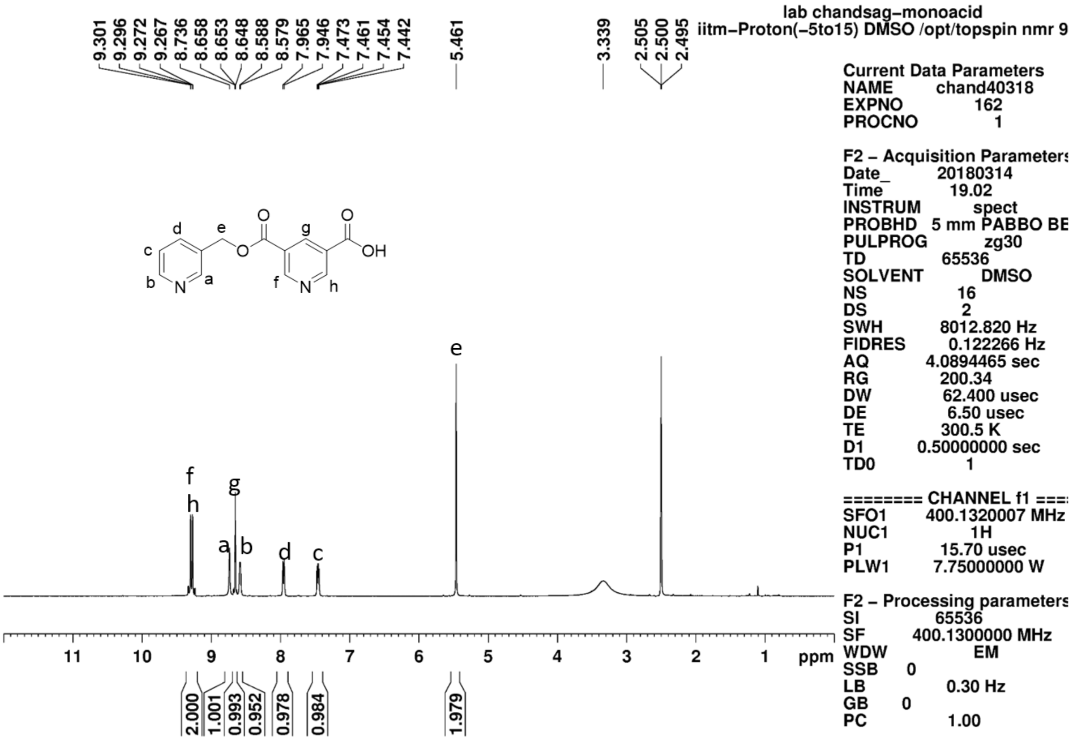

Supplementary Figure 33. ^{1}H NMR spectrum (500 MHz, DMSO-d_6, 300 K) for the ligand **L4**.

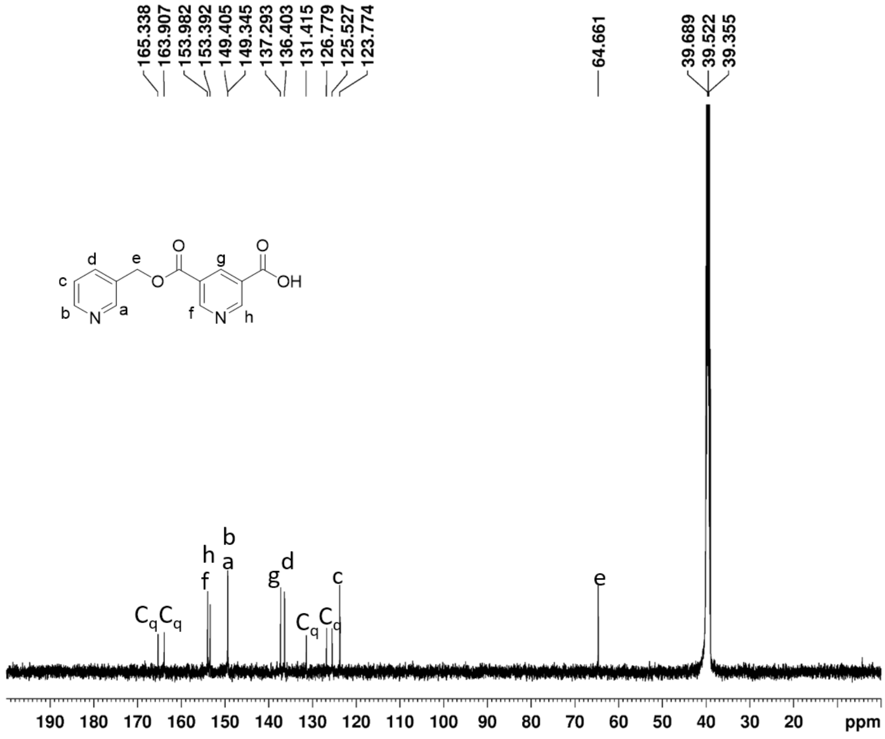

Supplementary Figure 34. ^{13}C NMR spectrum (125 MHz, DMSO-d_6, 300 K) for the ligand **L4**.

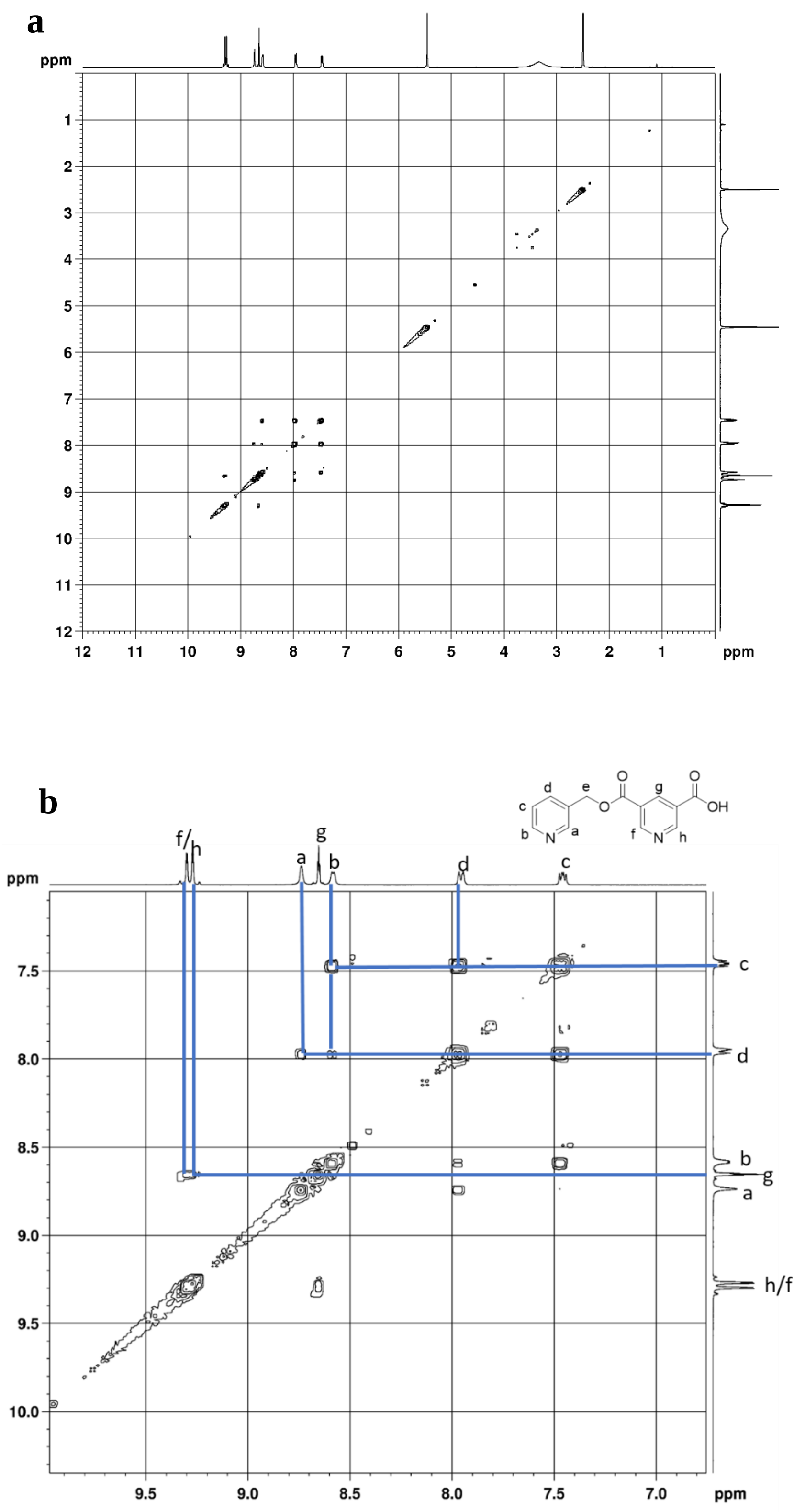

Supplementary Figure 35. H-H COSY spectrum (500 MHz, DMSO-d_6, 300 K) for **a** the ligand **L4**, and **b** expansion of the spectrum.

a

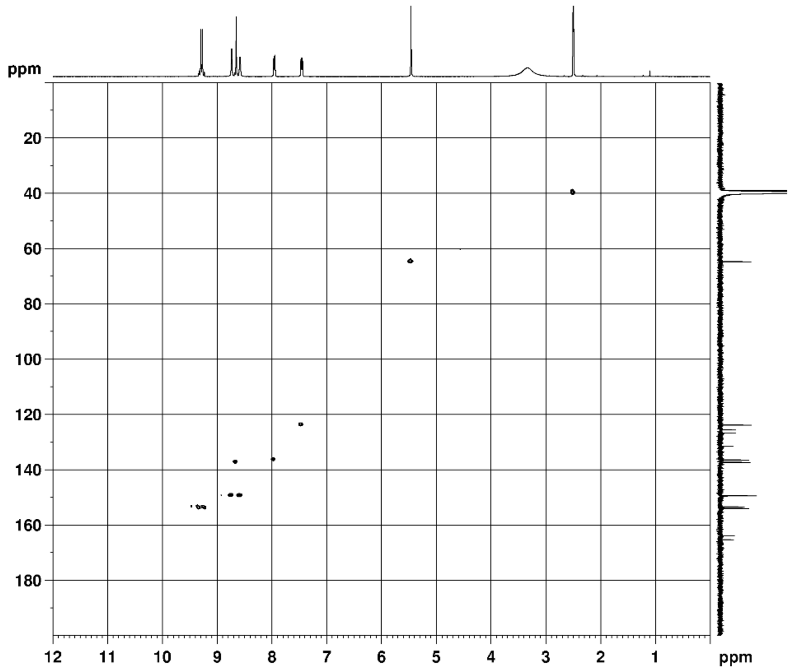

b

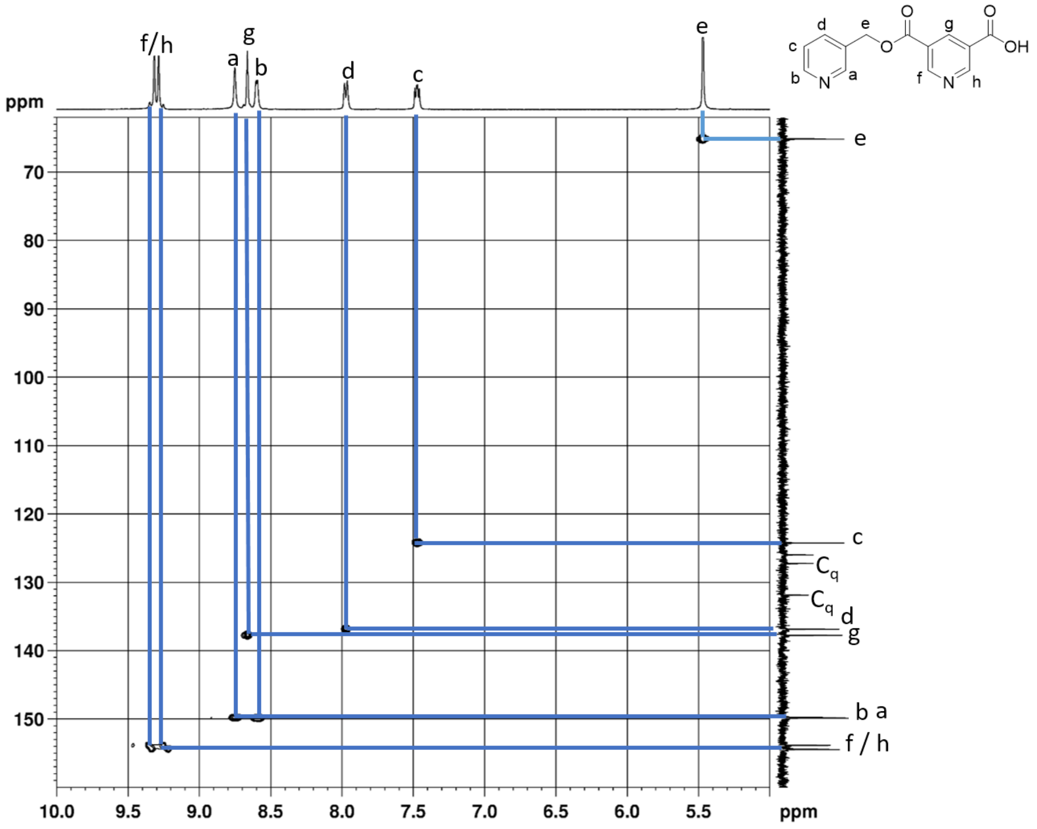

Supplementary Figure 36. C-H COSY spectrum (500 MHz, DMSO-d_6, 300 K) for **a** the ligand **L4**, and **b** expansion of the spectrum.

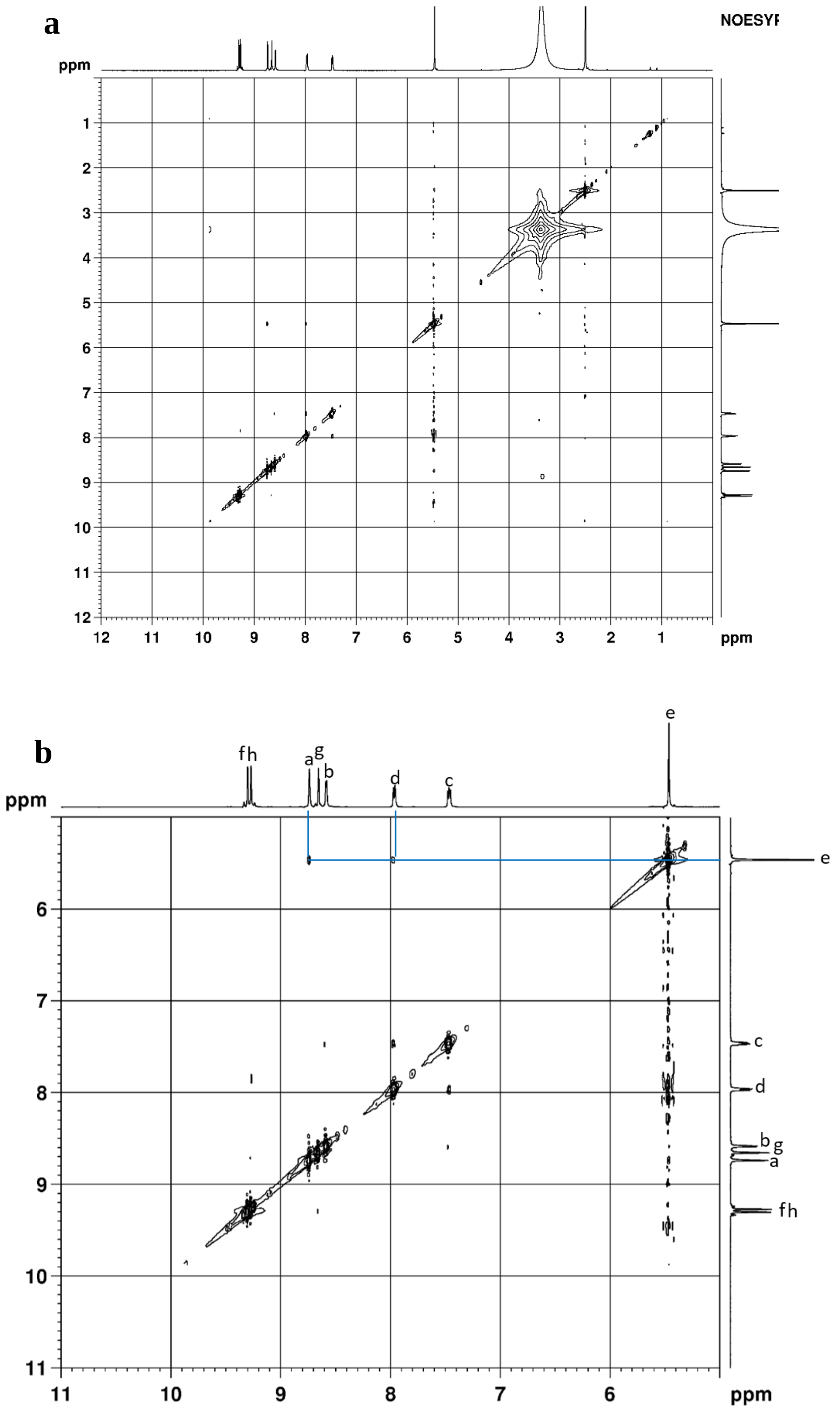

Supplementary Figure 37. NOESY spectrum (500 MHz, DMSO-d_6, 300 K) for **a** the ligand **L4**, and **b** expansion of the spectrum.

Compound Details

Cpd. 1: C13 H10 N2 O4

Compound Spectra (overlaid)

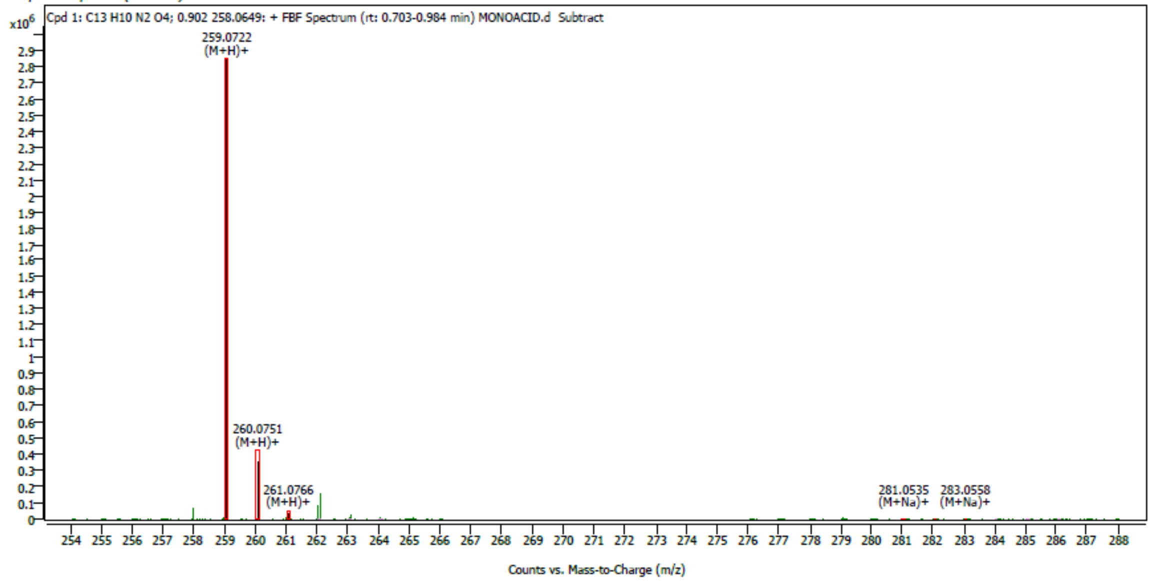

Compound ID Table

Cpd	Formula	Mass (Tgt)	Calc. Mass	Mass	Species	Diff(Tgt.ppm)	mDa
1	C13 H10 N2 O4	258.0641	258.0649	259.0722<br>281.0535	(M+H)+<br>(M+Na)+	3.24	0.84

Supplementary Figure 38. ESI mass spectrum for the ligand **L4**.

Characterisation of the ligand, L4'

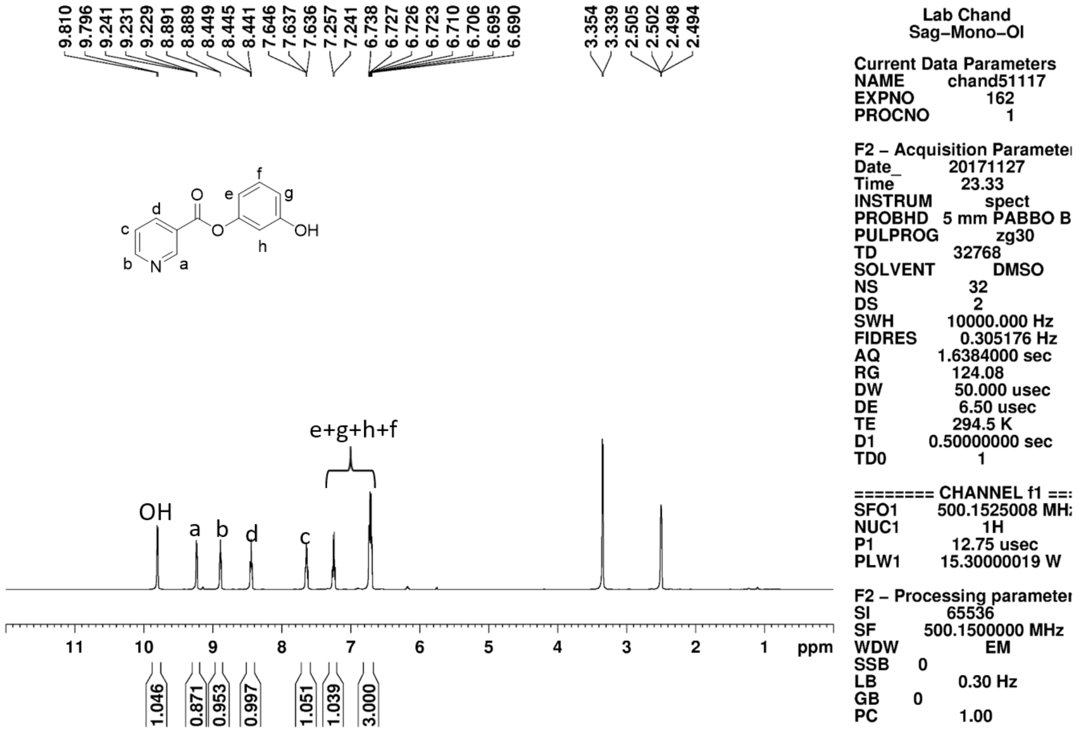

Supplementary Figure 39. ^{1}H NMR spectrum (500 MHz, DMSO-d_6, 300 K) for **L4'**.

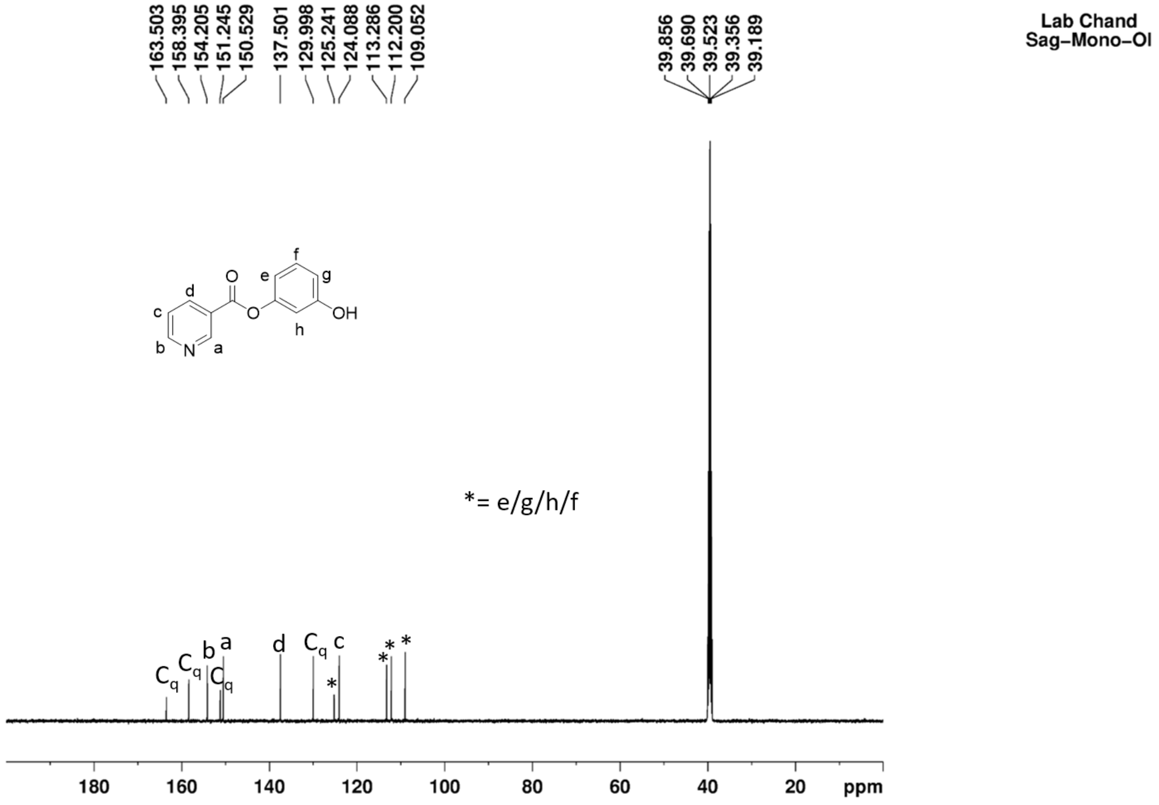

Supplementary Figure 40. ^{13}C NMR spectrum (125 MHz, DMSO-d_6, 300 K) for **L4'**.

a

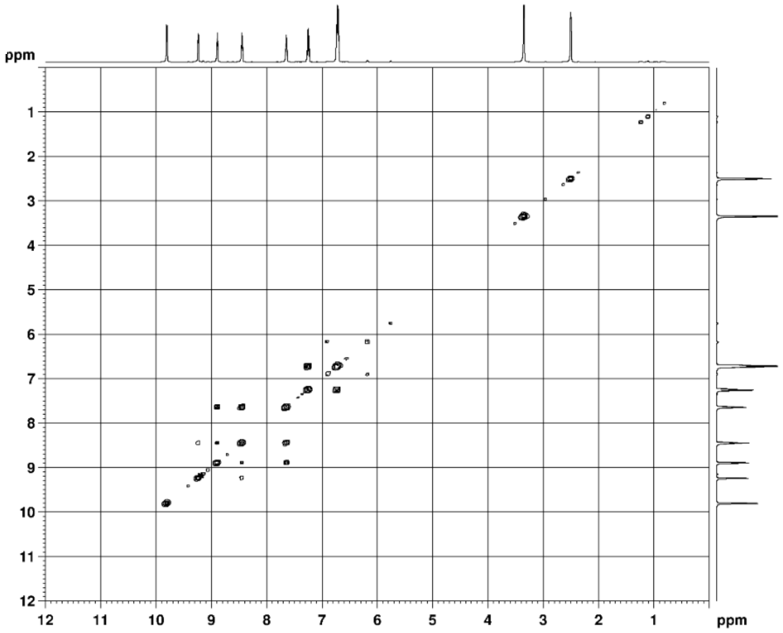

b

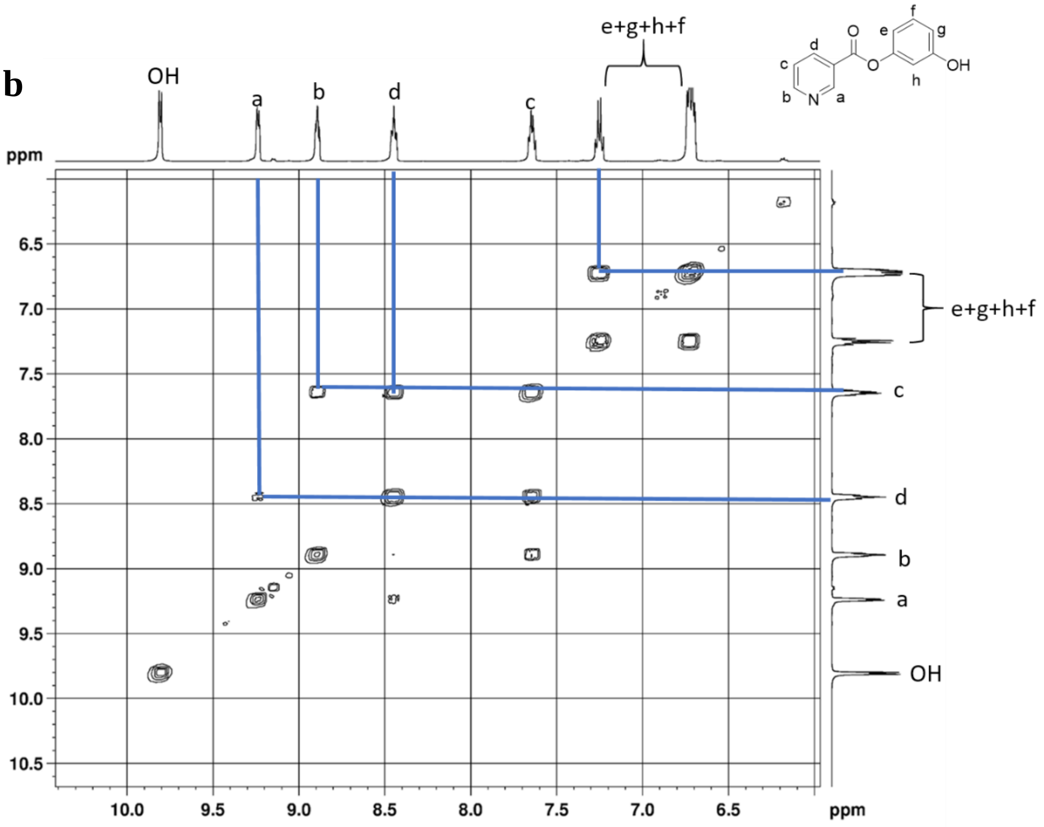

Supplementary Figure 41. H-H COSY spectrum (500 MHz, DMSO-d_6, 300 K) for **a** the ligand **L4'**, and **b** expansion of the spectrum.

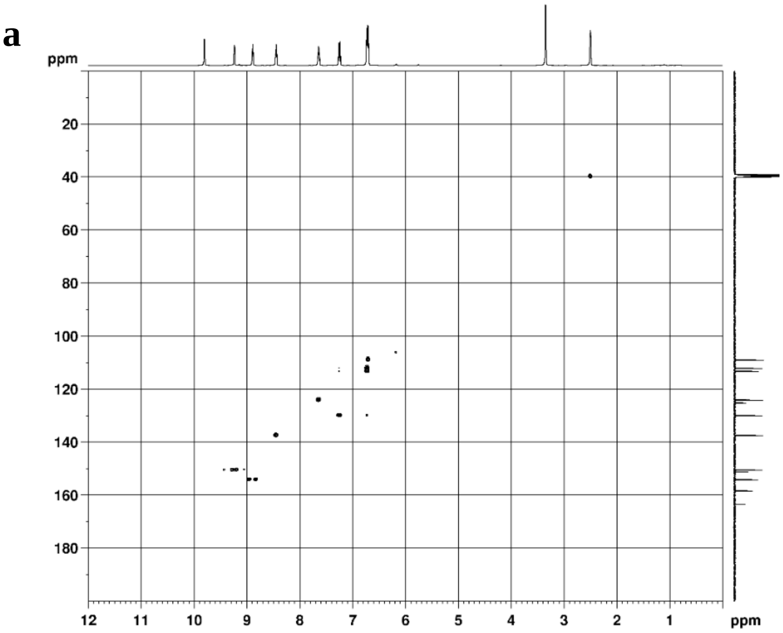

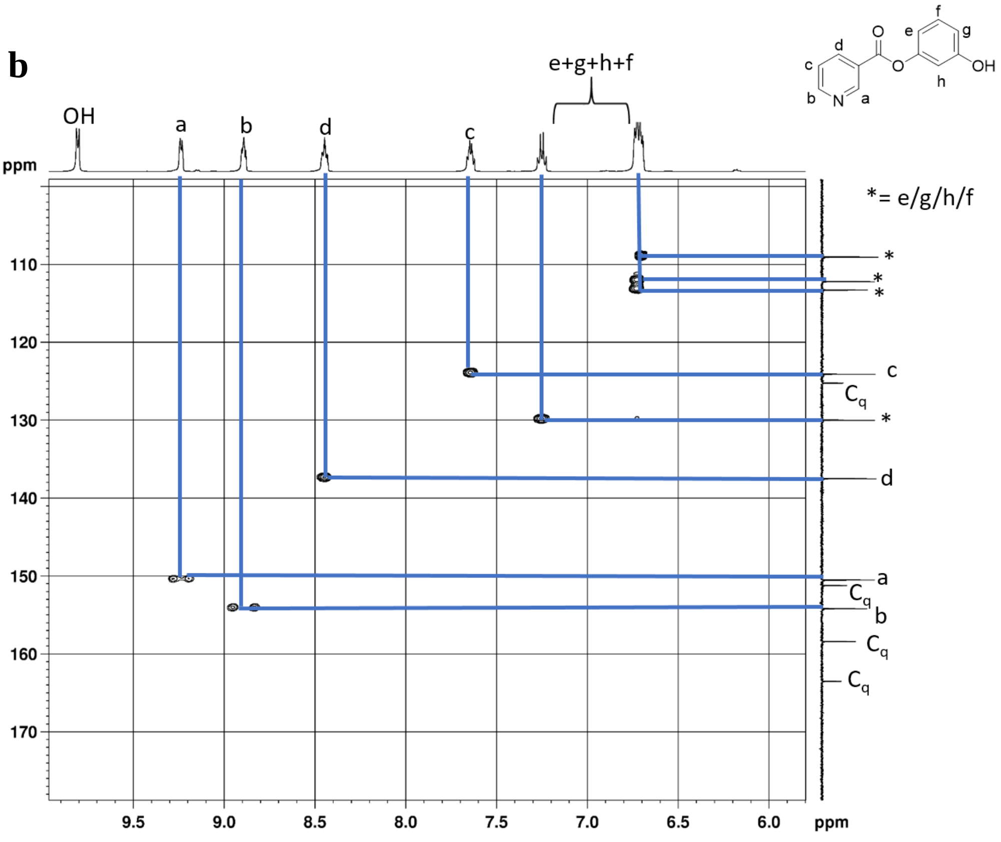

Supplementary Figure 42. C-H COSY spectrum (500 MHz, DMSO-d_6, 300 K) for **a** the ligand **L4'**, and **b** expansion of the spectrum.

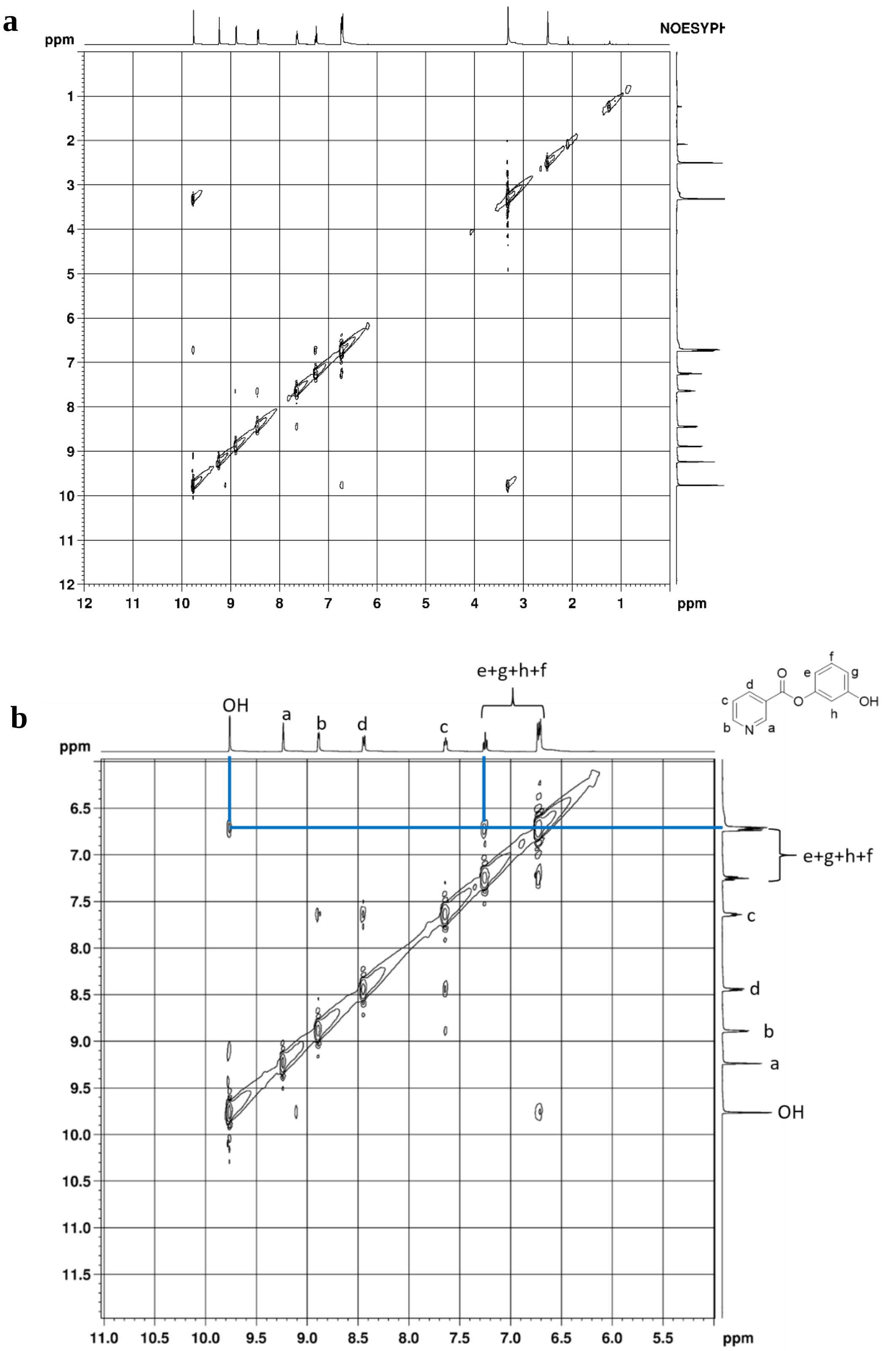

Supplementary Figure 43. NOESY spectrum (500 MHz, DMSO-d_6, 300 K) for **a** the ligand **L4'**, and **b** expansion of the spectrum.

Characterisation of the ligand, L5

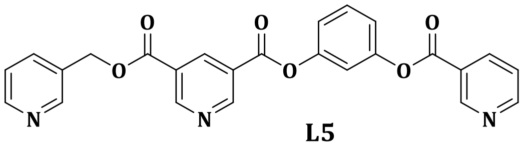

L5

a

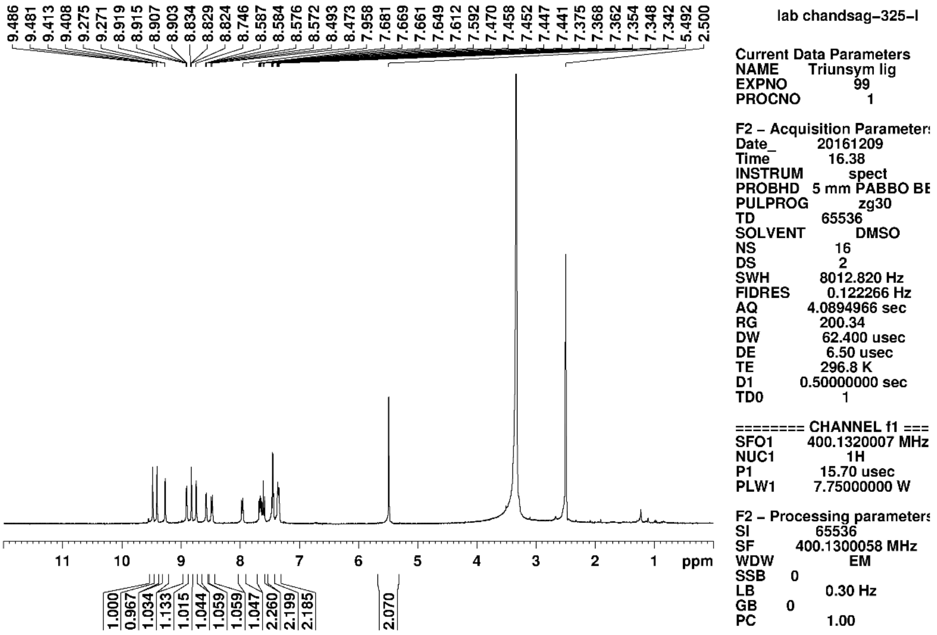

b

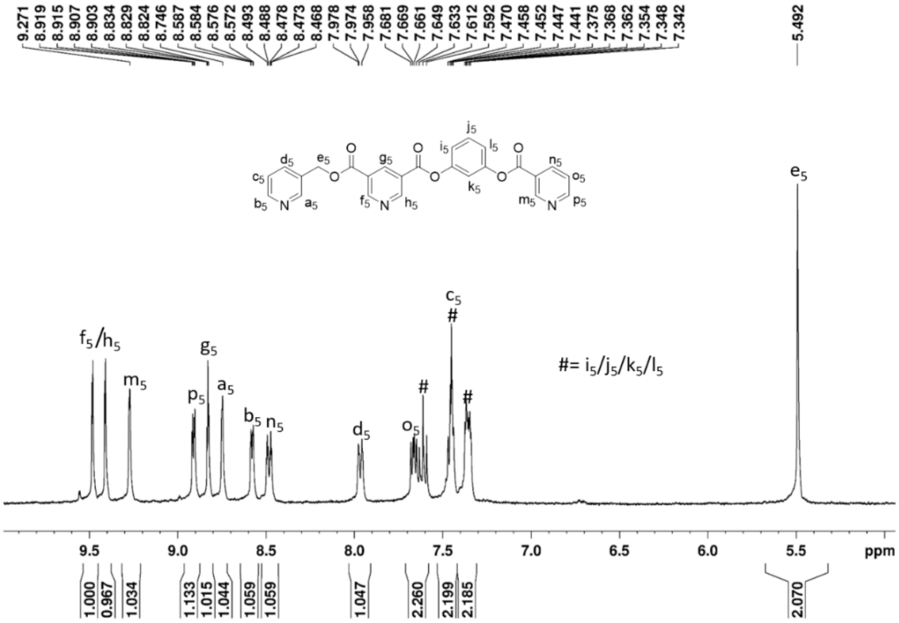

Supplementary Figure 44. ^{1}H NMR spectrum (500 MHz, DMSO-d_6, 300 K) for **a** the ligand **L5**, and **b** expansion of the spectrum.

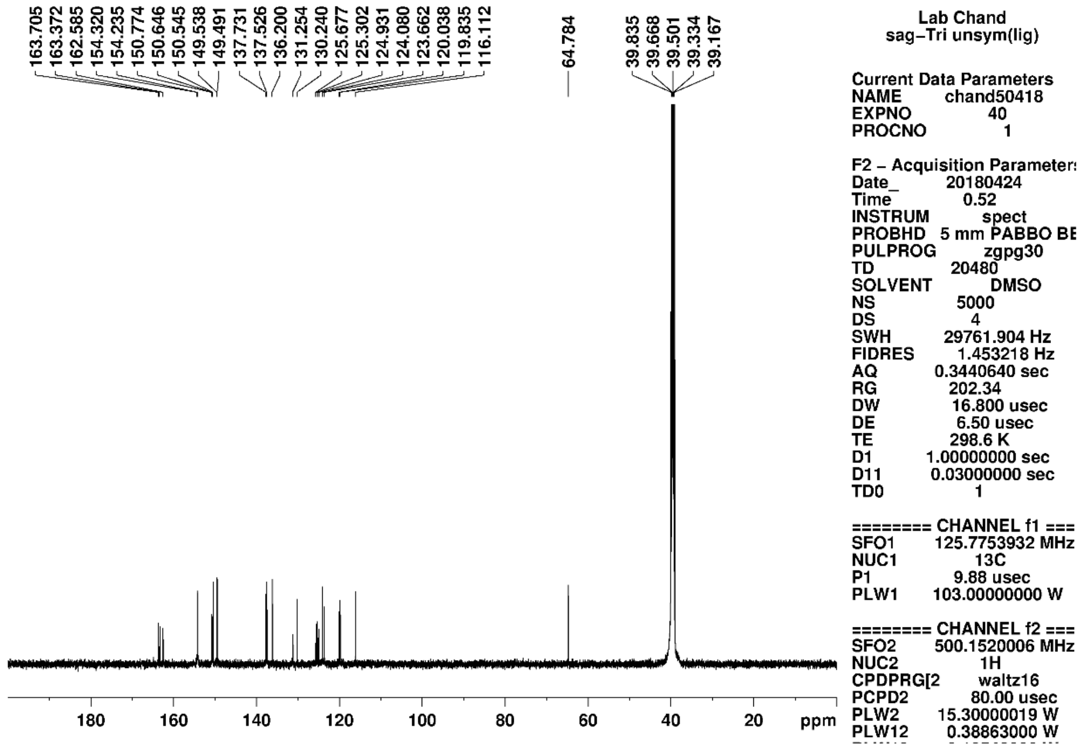

Supplementary Figure 45. ^{13}C NMR spectrum (125 MHz, DMSO-d_6, 300 K) for **L5**.

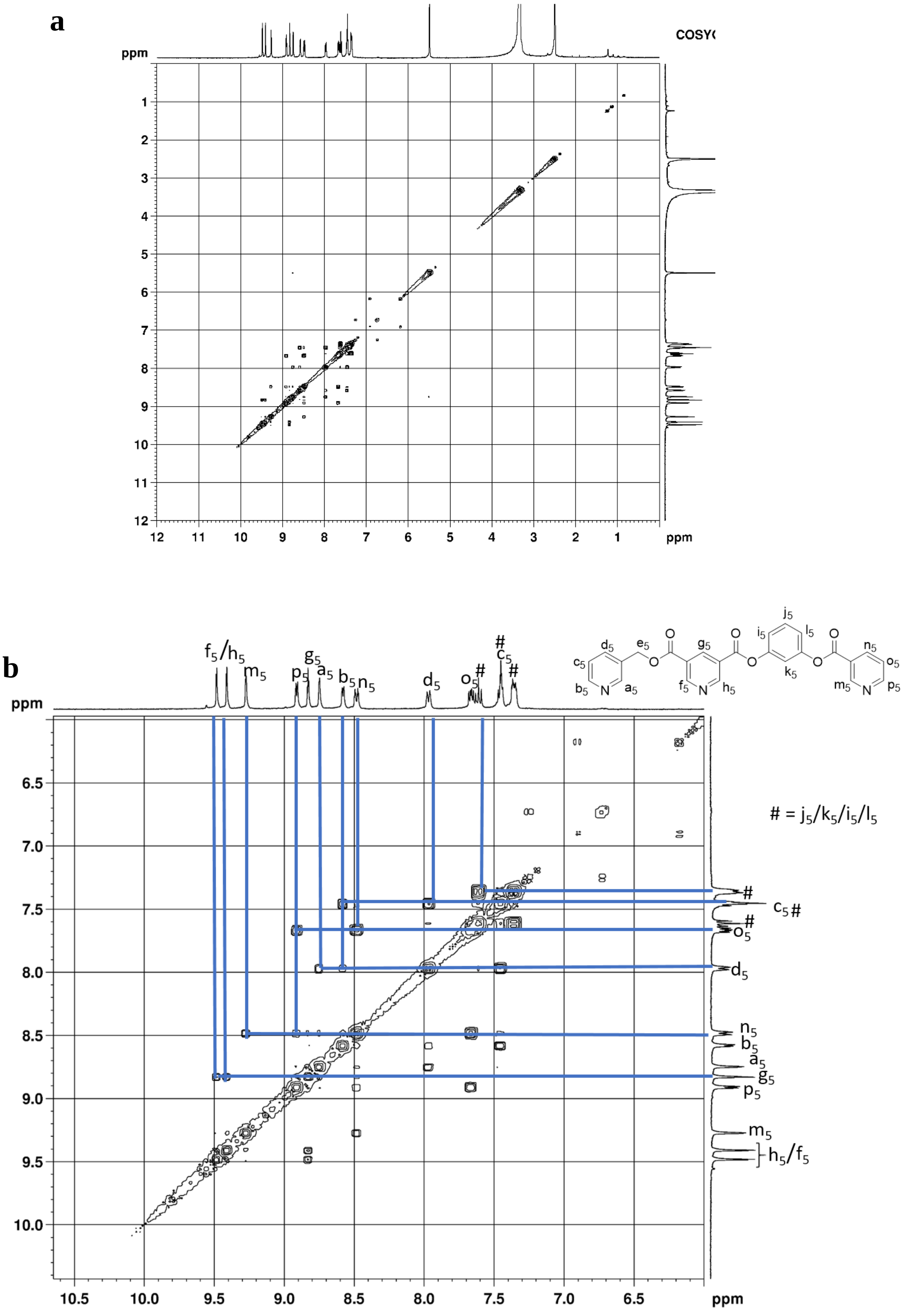

Supplementary Figure 46. H-H COSY spectrum (500 MHz, DMSO-d_6, 300 K) for **a** the ligand **L5**, and **b** expansion of the spectrum.

.

a

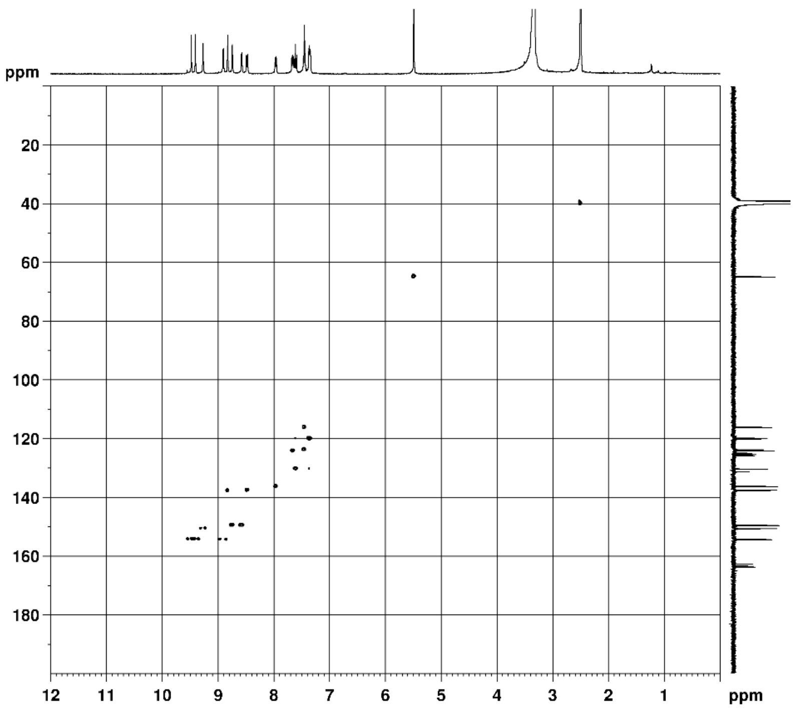

b

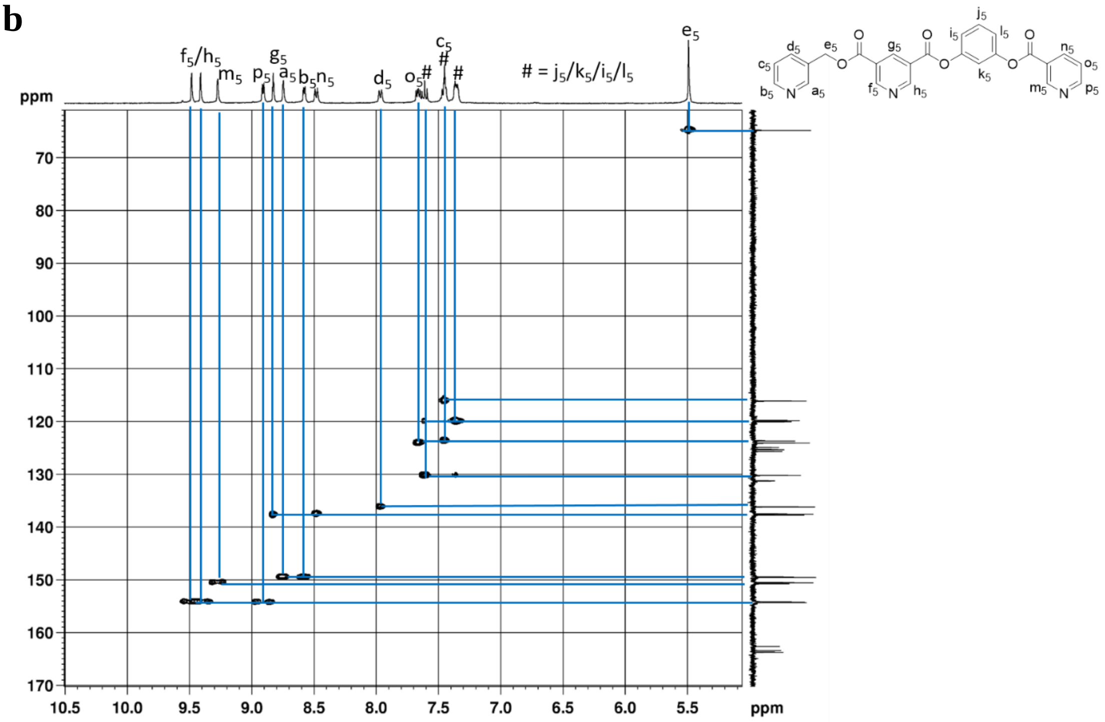

Supplementary Figure 47. C-H COSY spectrum (500 MHz, DMSO-d_6, 300 K) for **a** the ligand **L5**, and **b** expansion of the spectrum.

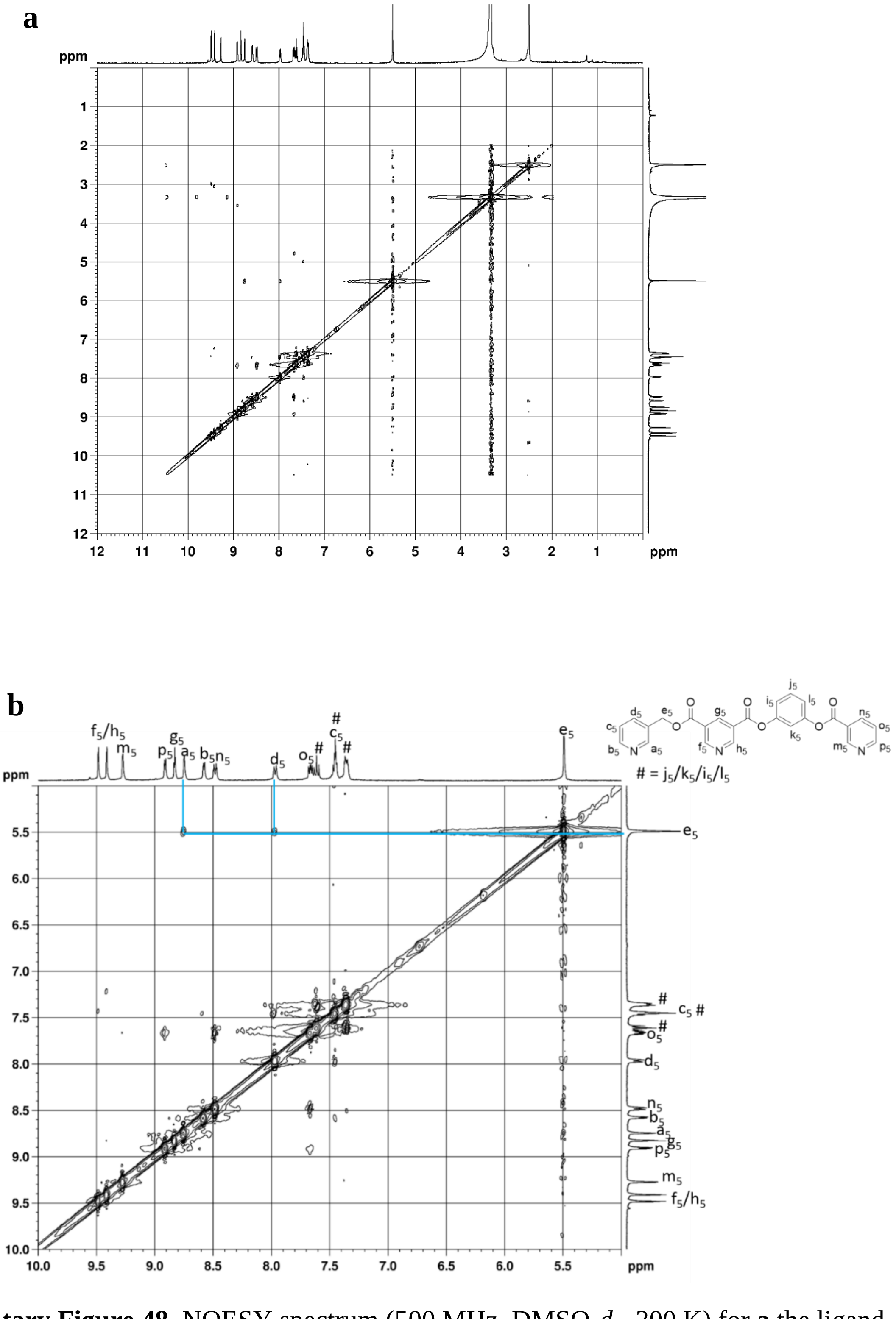

Supplementary Figure 48. NOESY spectrum (500 MHz, DMSO-d_6, 300 K) for **a** the ligand **L5**, and **b** expansion of the spectrum.

Compound Details

Cpd. 1: C25 H17 N3 O6

Compound Spectra (overlaid)

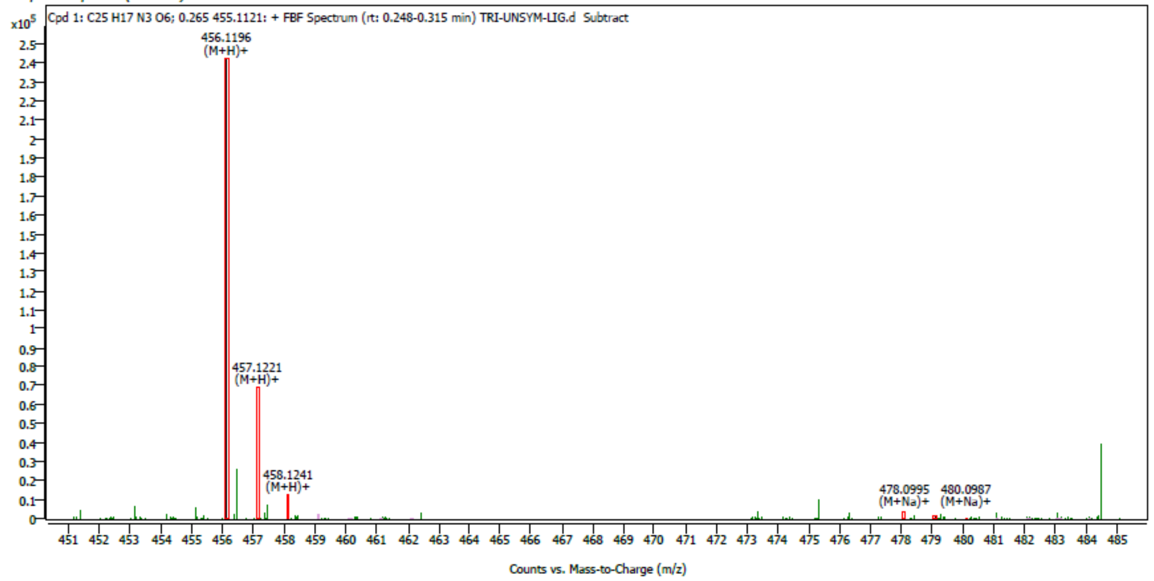

Compound ID Table

Cpd	Formula	Mass (Tgt)	Calc. Mass	Mass	Species	Diff(Tgt.ppm)	mDa
1	C25 H17 N3 O6	455.1117	455.1121	456.1196<br>478.0995	(M+H)+<br>(M+Na)+	0.87	0.40

Supplementary Figure 49. ESI mass spectrum for the ligand **L5**.

Monitoring the formation of the complex [NO$_3$⊂Pd$_4$(L3)$_2$(L5)$_4$](NO$_3$)$_7$, 4a at room temperature

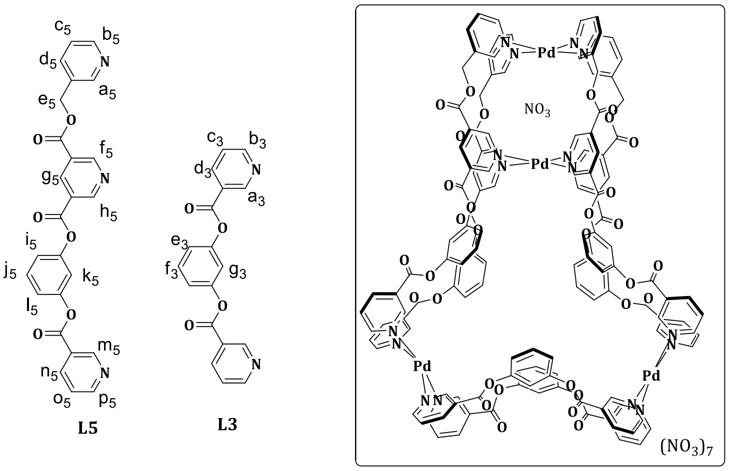

4a

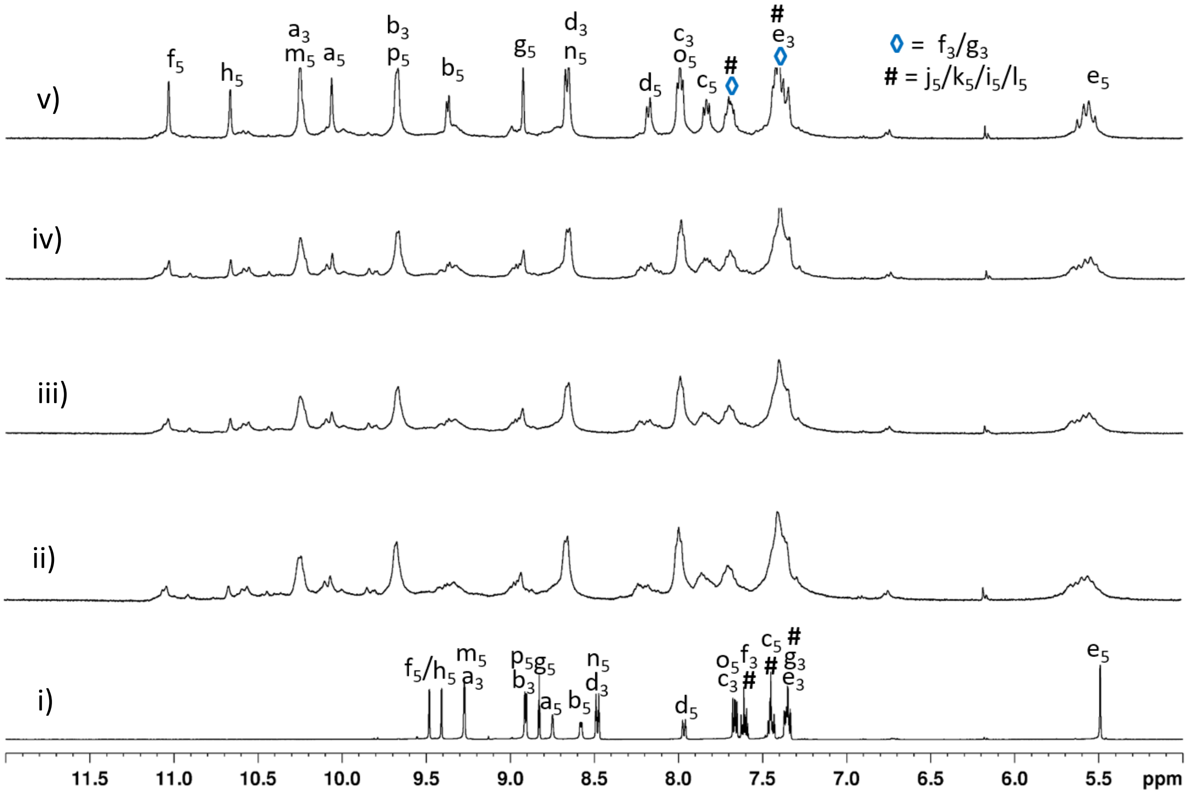

Supplementary Figure 50. 400 MHz partial ^{1}H NMR spectra (400 MHz, DMSO-d_6, 300 K) of: (i) mixture of **L3** and **L5** at 2:4 ratio (ii)-(v) monitoring the reaction of **L3**, **L5** and Pd(NO$_3$)$_2$ in 2 : 4: 4 ratio after mixing (ii) 30 min., (iii) 60 min., (iv) 2 h, and (v) 4 h, complex [NO$_3$⊂Pd$_4$(**L3**)$_2$(**L5**)$_4$](NO$_3$)$_7$, **4a**.

Monitoring the formation of the complex $[NO_3\subset Pd_4(L3)_2(L5)_4](NO_3)_7$, 4a at 70 °C

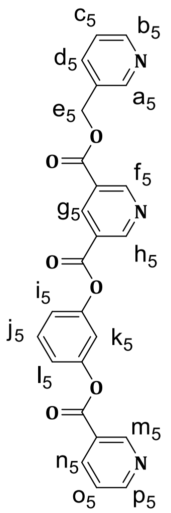

L5

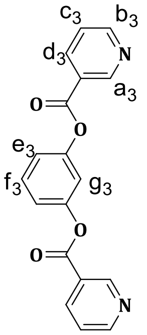

L3

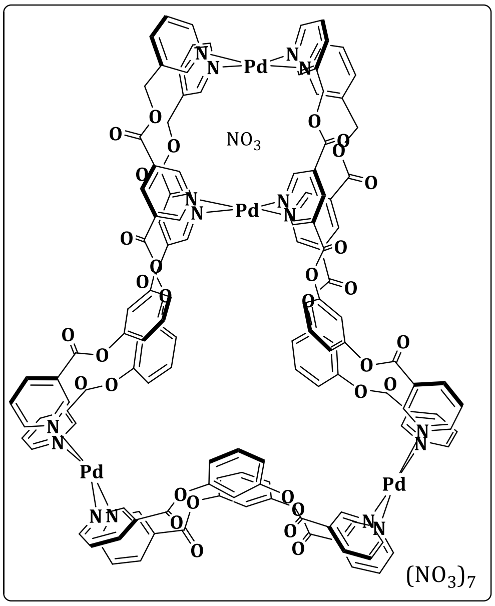

4a

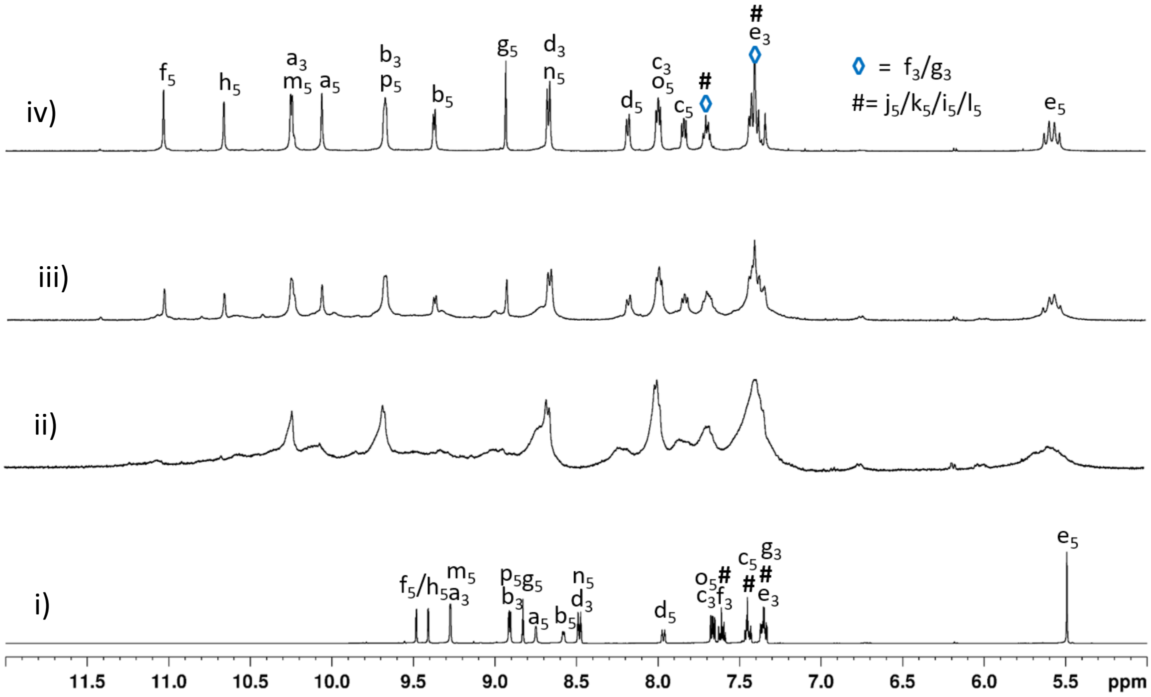

Supplementary Figure 51. 400 MHz partial ^{1}H NMR spectra (400 MHz, DMSO-d_6, 300 K) of: (i) mixture of **L3** and **L5** at 2:4 ratio (ii)-(iii) monitoring the reaction of **L3**, **L5** and $Pd(NO_3)_2$ in 2 : 4: 4 ratio (ii) immediately after mixing at room temperature (iii) 30 min of heating and (iv) 60 min of heating, complex $[NO_3\subset Pd_4(\mathbf{L3})_2(\mathbf{L5})_4](NO_3)_7$, **4a**.

Characterisation of the complex [NO$_3$⊂Pd$_4$(L3)$_2$(L5)$_4$](NO$_3$)$_7$, 4a.

a

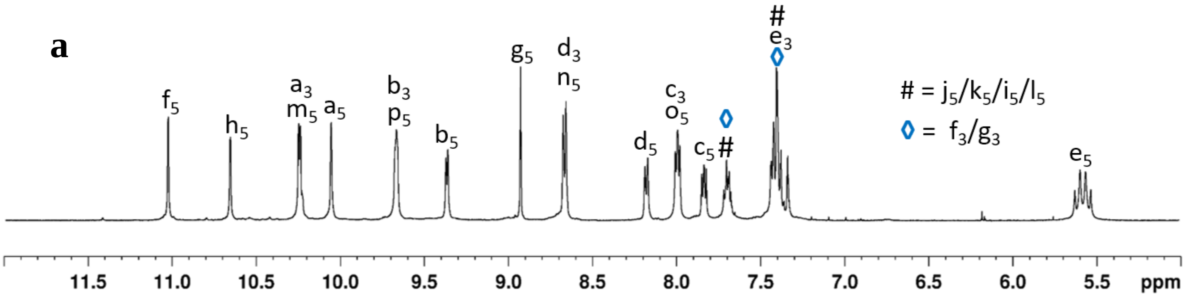

b

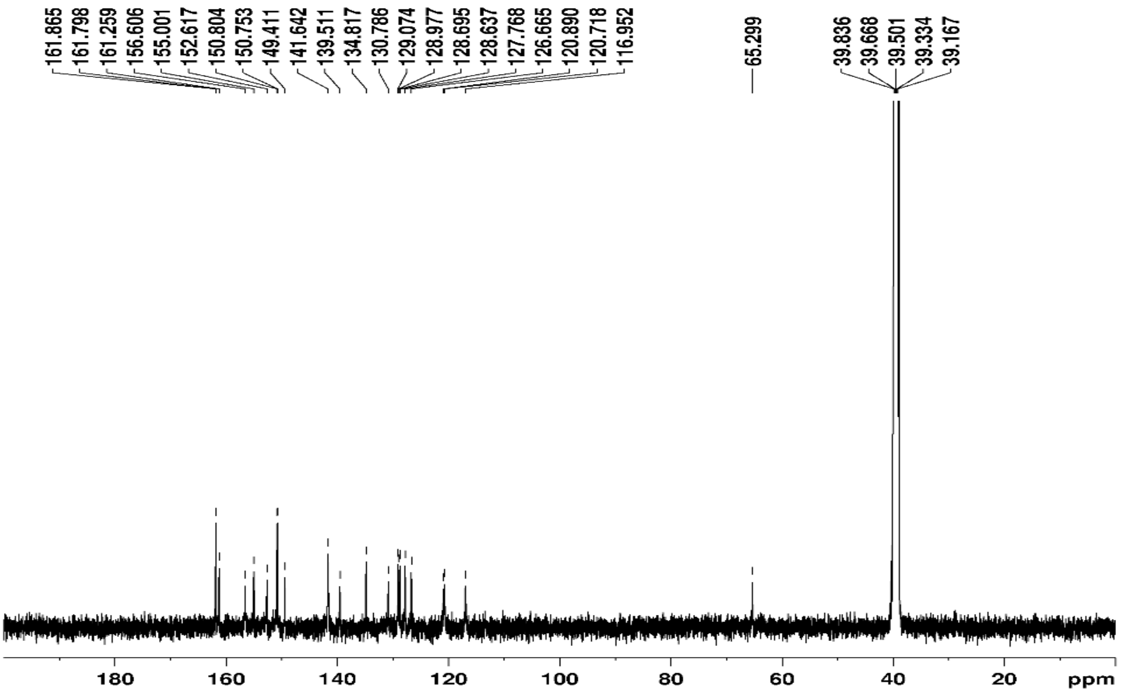

Supplementary Figure 52. NMR spectra of [NO$_3$⊂Pd$_4$(**L3**)$_2$(**L5**)$_4$](NO$_3$)$_7$, **4a** including **a** ^{1}H NMR spectrum (500 MHz, DMSO-*d*$_6$, 300 K) for the complex (Concentration: 10 mM with respect to palladium(II) source), and **b** ^{13}C NMR spectrum (125 MHz, DMSO-*d*$_6$, 300 K) for **4a**, some of the peaks are overlapped.

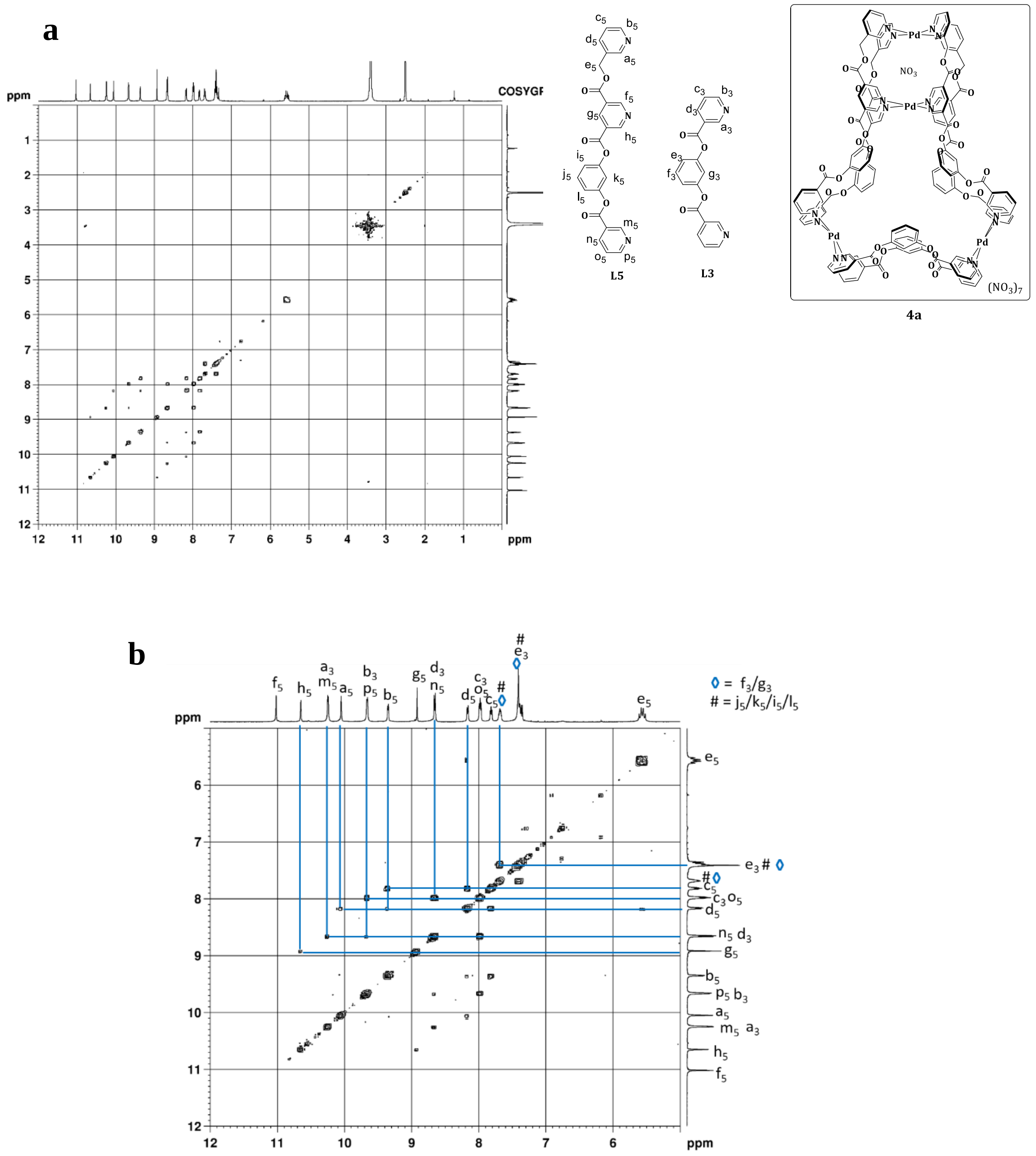

Supplementary Figure 53. H-H COSY spectrum (500 MHz, DMSO-d_6, 300 K) for **a** the complex **4a**, and **b** expansion of the spectrum.

a

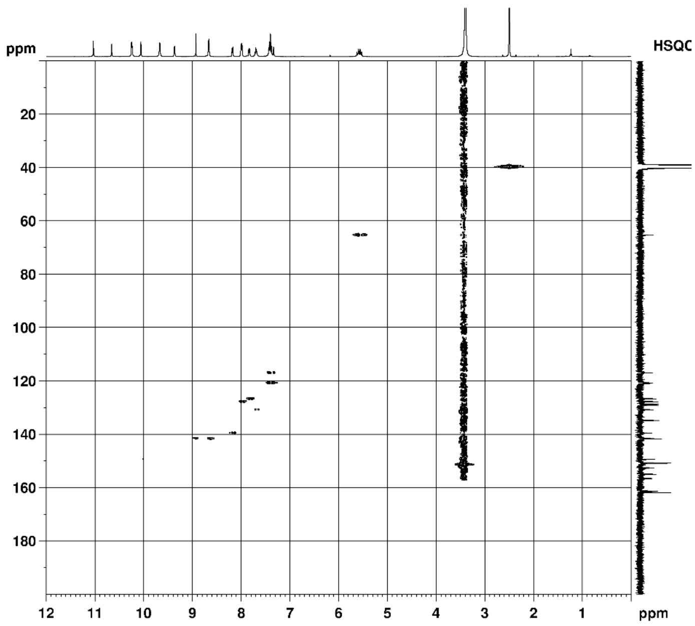

b

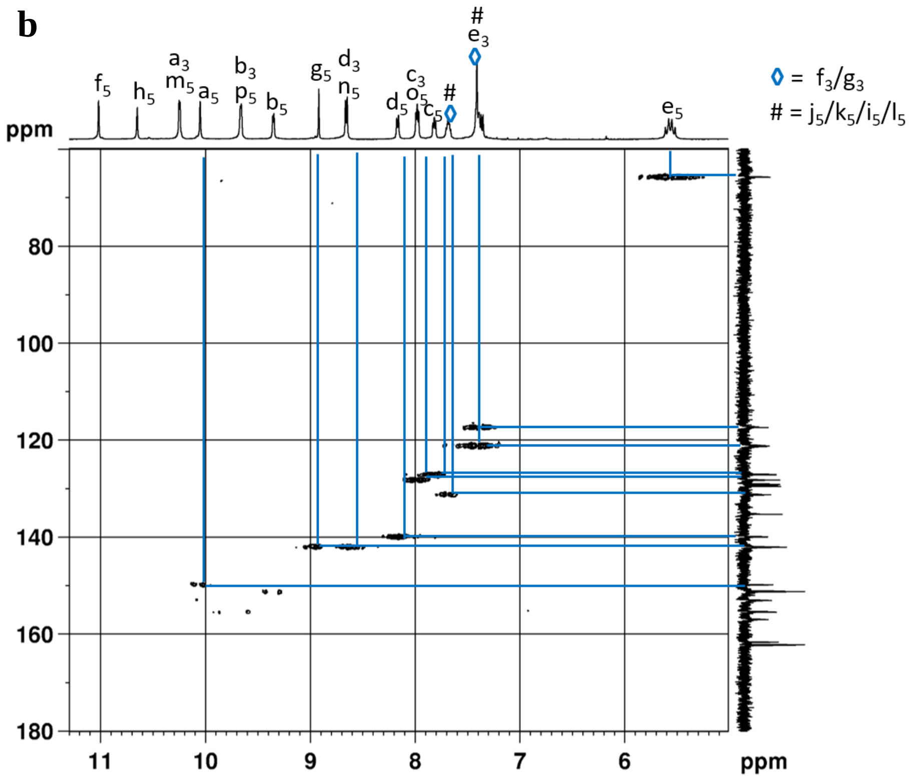

Supplementary Figure 54. C-H COSY spectrum (500 MHz, DMSO-d_6, 300 K) for **a** the complex **4a**, and **b** expansion of the spectrum.

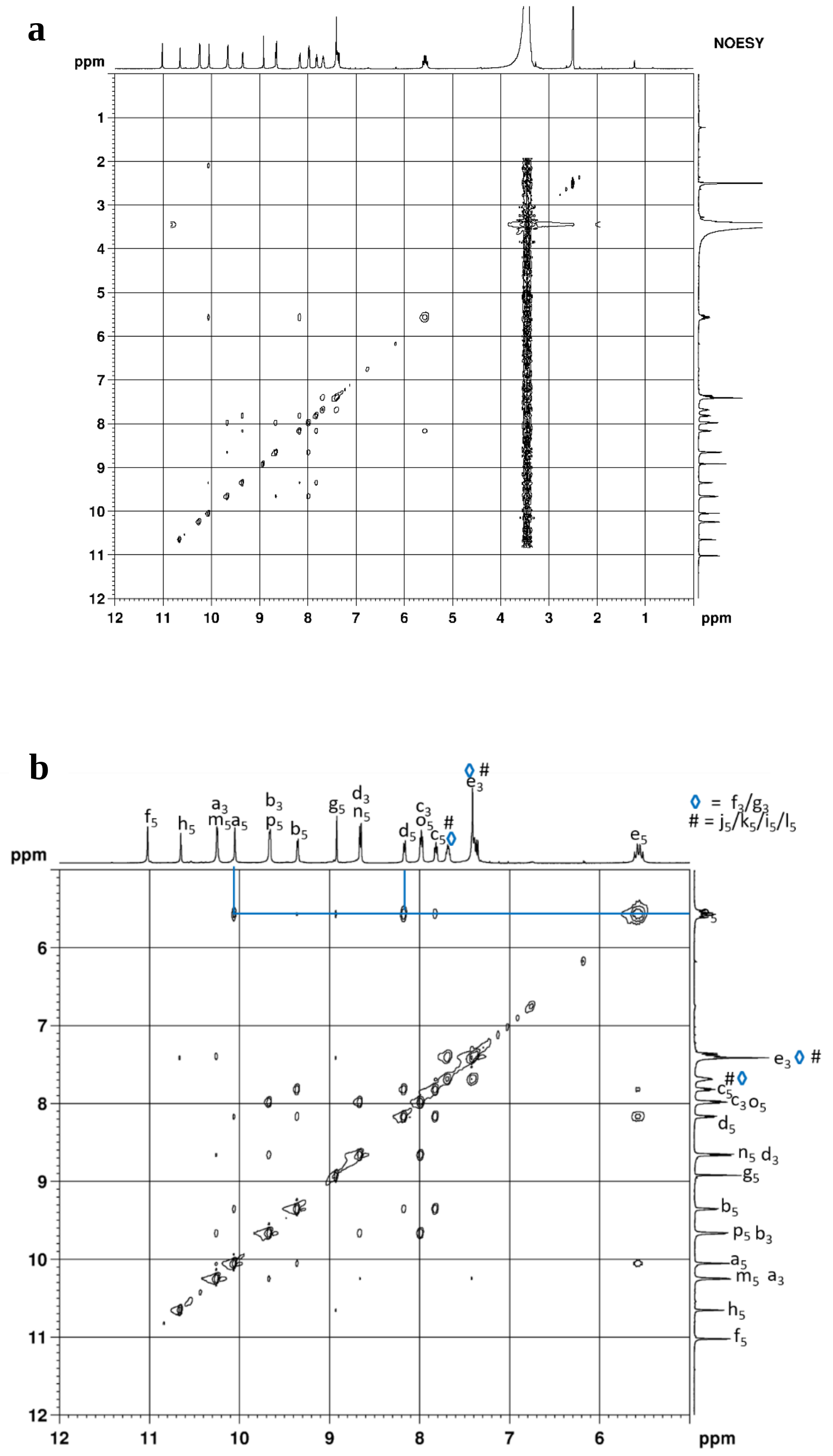

Supplementary Figure 55. NOESY spectrum (500 MHz, DMSO-d_6, 300 K) for **a** the complex **4a**, and **b** expansion of the spectrum.

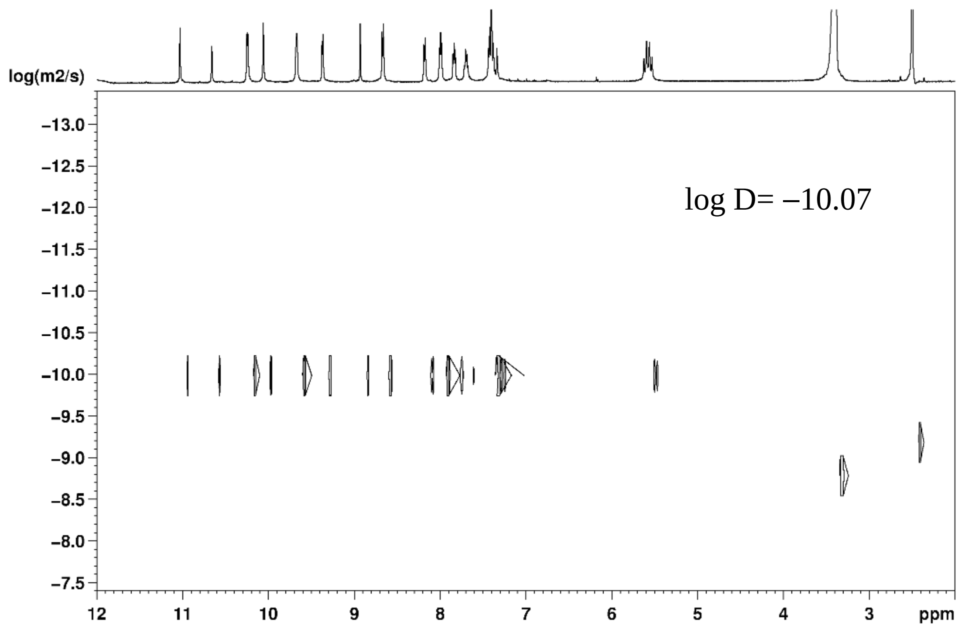

Supplementary Figure 56. ^{1}H DOSY NMR spectrum for complex $[NO_3 \subset Pd_4(\mathbf{L3})_2(\mathbf{L5})_4](NO_3)_7$, **4a** showing single band with diffusion co-efficient (D) value of $8.51 \times 10^{-11}\ m^2\ s^{-1}$.

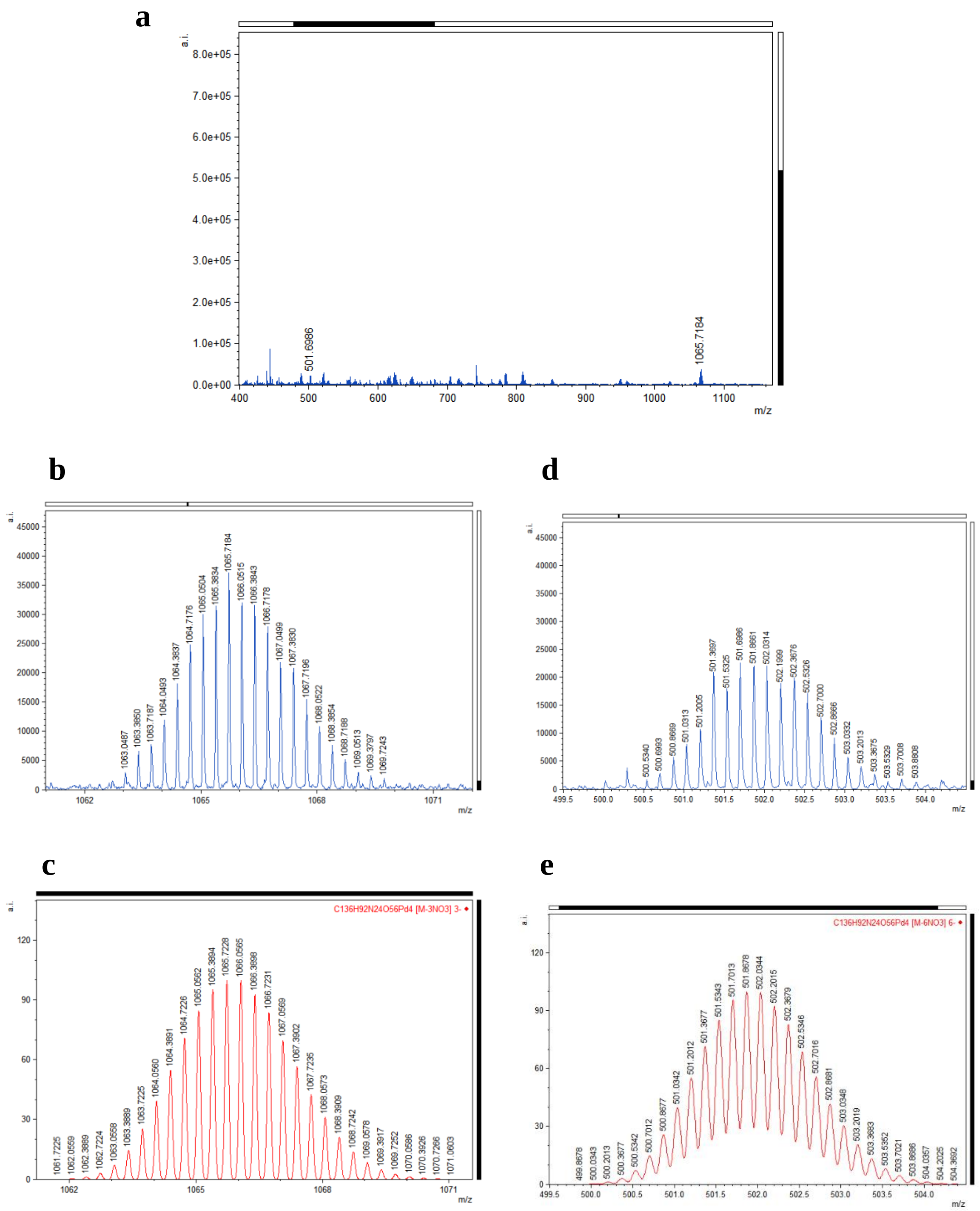

Supplementary Figure 57. ESI mass spectrum for a mixture of [NO_3⊂Pd_4(**L3**)$_2$(**L5**)$_4$](NO_3)$_7$, **4a** showing **a** full spectrum, **b** experimental isotopic pattern for [**4a**–3NO_3]$^{3+}$, **c** theoretical isotopic pattern for [**4a**–3NO_3]$^{3+}$, **d** experimental isotopic pattern for [**4a**–6NO_3]$^{6+}$, and **e** theoretical isotopic pattern for [**4a**–6NO_3]$^{6+}$.

Characterization of the complexes [X⊂Pd_4(**L3**)$_2$(**L5**)$_4$](NO_3)$_7$, 4b-4d

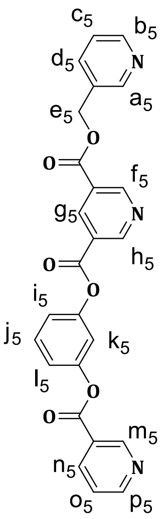

L5

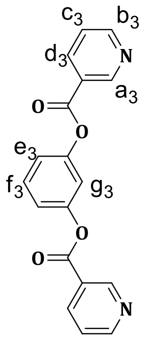

L3

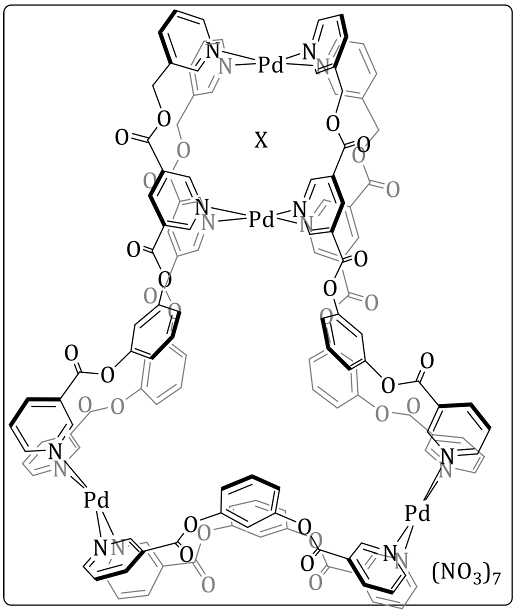

4b-4d

X= F, **4b**
Cl, **4c**
Br, **4d**

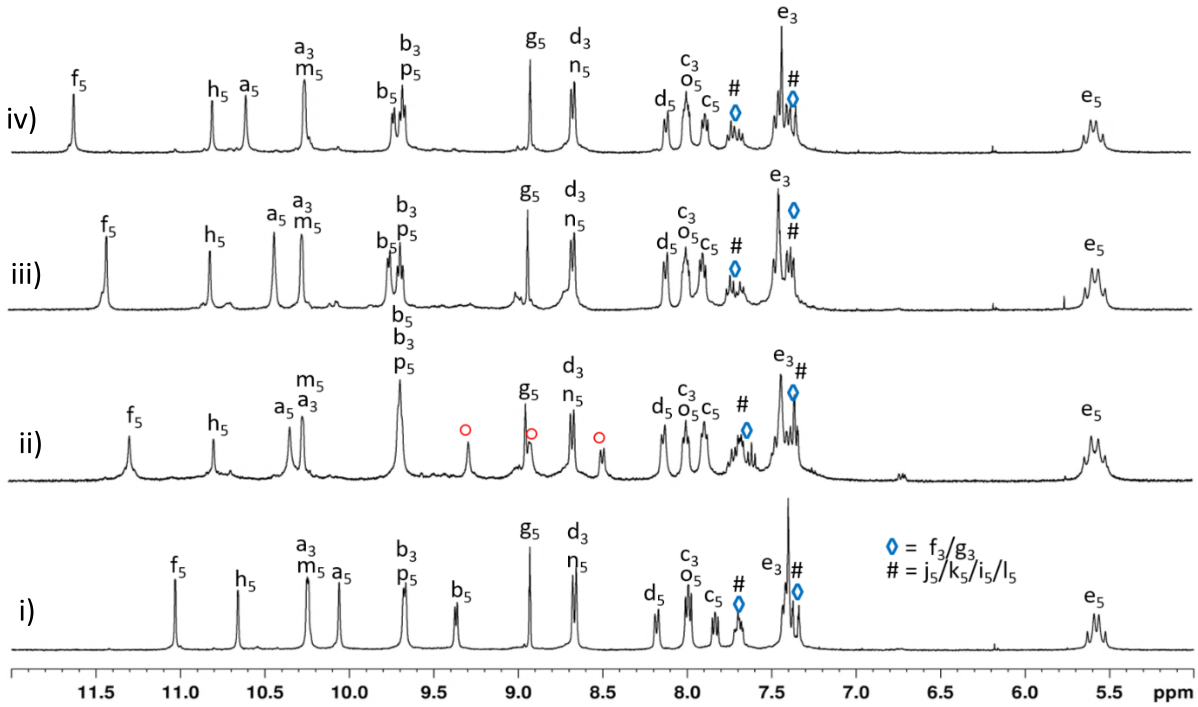

Supplementary Figure 58. 400 MHz partial ^{1}H NMR spectra in DMSO-d_6 for (i) [NO_3⊂Pd_4(**L3**)$_2$(**L5**)$_4$](NO_3)$_7$, **4a**; (ii) [F⊂Pd_4(**L3**)$_2$(**L5**)$_4$](NO_3)$_7$, **4b**; (iii) [Cl⊂Pd_4(**L3**)$_2$(**L5**)$_4$](NO_3)$_7$, **4c** and (iii) [Br⊂Pd_4(**L3**)$_2$(**L5**)$_4$](NO_3)$_7$, **4d**. [The peaks highlighted with red circle represents free ligand **L3**]

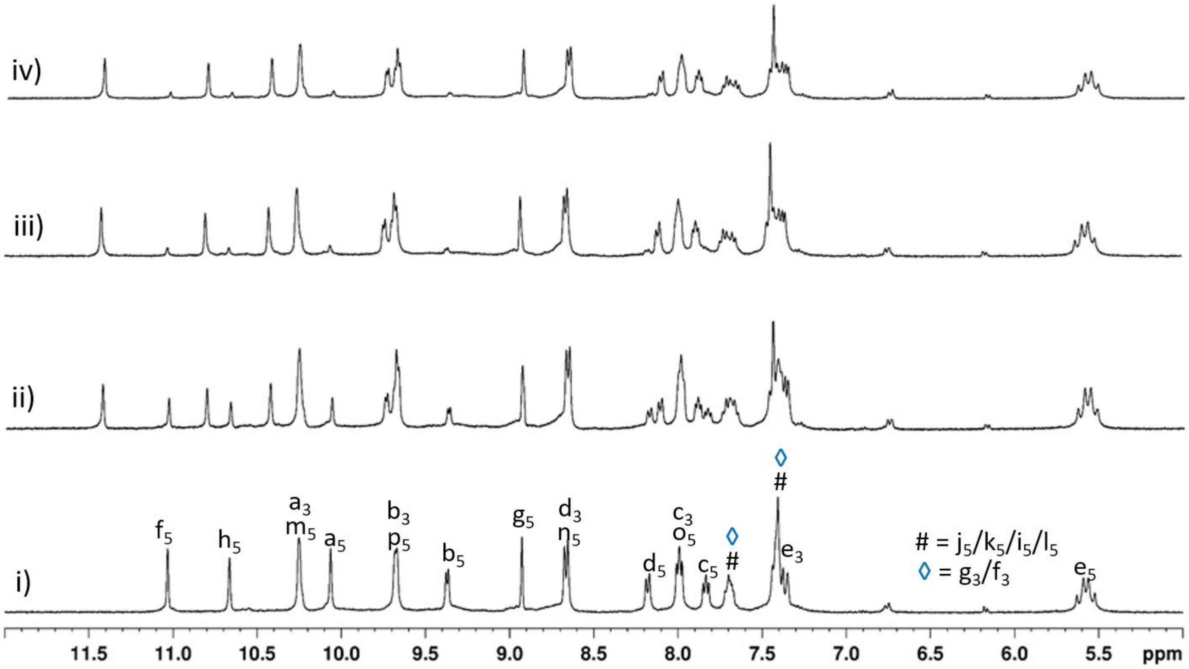

Supplementary Figure 59. Partial ^{1}H NMR spectra (400 MHz DMSO-d_6, 300 K) for (i) $[NO_3{\subset}Pd_4(\mathbf{L3})_2(\mathbf{L5})_4](NO_3)_7$, **4a**; addition of 2 equiv. of AgCl to **4a** followed by stirring at room temperature and monitoring after (ii) 30 min (iii) 60 min that showed a mixture of $[Cl{\subset}Pd_4(\mathbf{L3})_2(\mathbf{L5})_4](NO_3)_7$, **4c** and $[NO_3{\subset}Pd_4(\mathbf{L3})_2(\mathbf{L5})_4](NO_3)_7$, **4a**. Almost exclusive formation of (iv) $[Cl{\subset}Pd_4(\mathbf{L3})_2(\mathbf{L5})_4](NO_3)_7$, **4c** after 2h.

Supplementary Table 2. ^{1}H NMR chemical shift of selected protons in ppm for various complexes **4a-4d** (DMSO-d_6) for comparison with **L5**.

Proton	**L5**	$[NO_3{\subset}Pd_4(\mathbf{L3})_2(\mathbf{L5})_4](NO_3)_7$, **4a**	$[F{\subset}Pd_4(\mathbf{L3})_2(\mathbf{L5})_4](NO_3)_7$, **4b**	$[Cl{\subset}Pd_4(\mathbf{L3})_2(\mathbf{L5})_4](NO_3)_7$, **4c**	$[Br{\subset}Pd_4(\mathbf{L3})_2(\mathbf{L5})_4](NO_3)_7$, **4d**
H_{a5}	8.75	10.06	10.34	10.43	10.61
H_{b5}	8.58	9.37	9.68	9.75	9.73
H_{f5}	9.41 or 9.48	11.03	11.28	11.42	11.63
H_{h5}	9.41 or 9.48	10.66	10.79	10.81	10.81

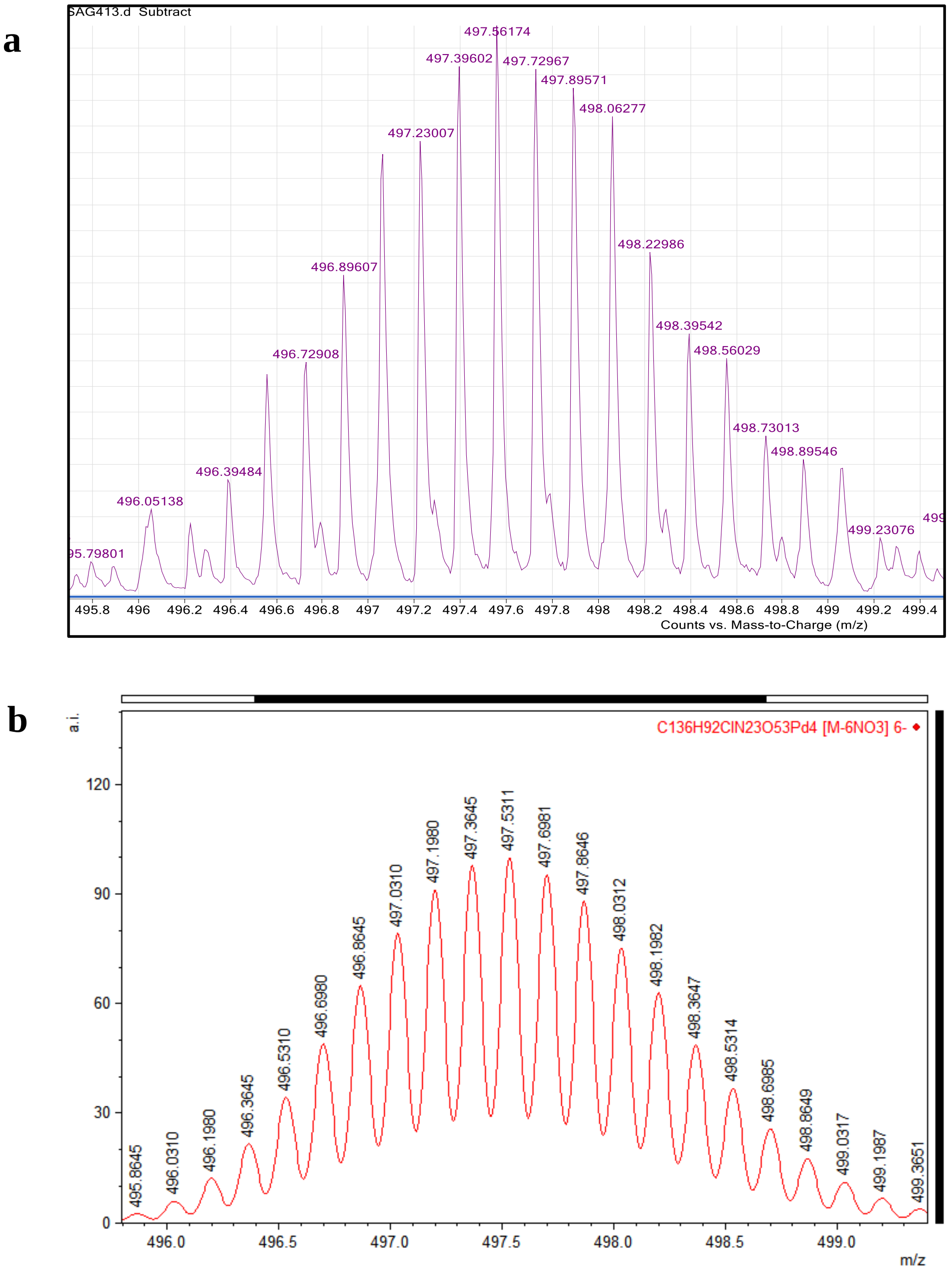

Supplementary Figure 60. ESI mass spectrum for [Cl⊂Pd_4(**L3**)$_2$(**L5**)$_4$](NO_3)$_7$, **4c** showing **a** experimental isotopic pattern for [**4c**–6NO_3]$^{+6}$, **b** theoretical isotopic pattern for [**4c**–6NO_3]$^{+6}$,

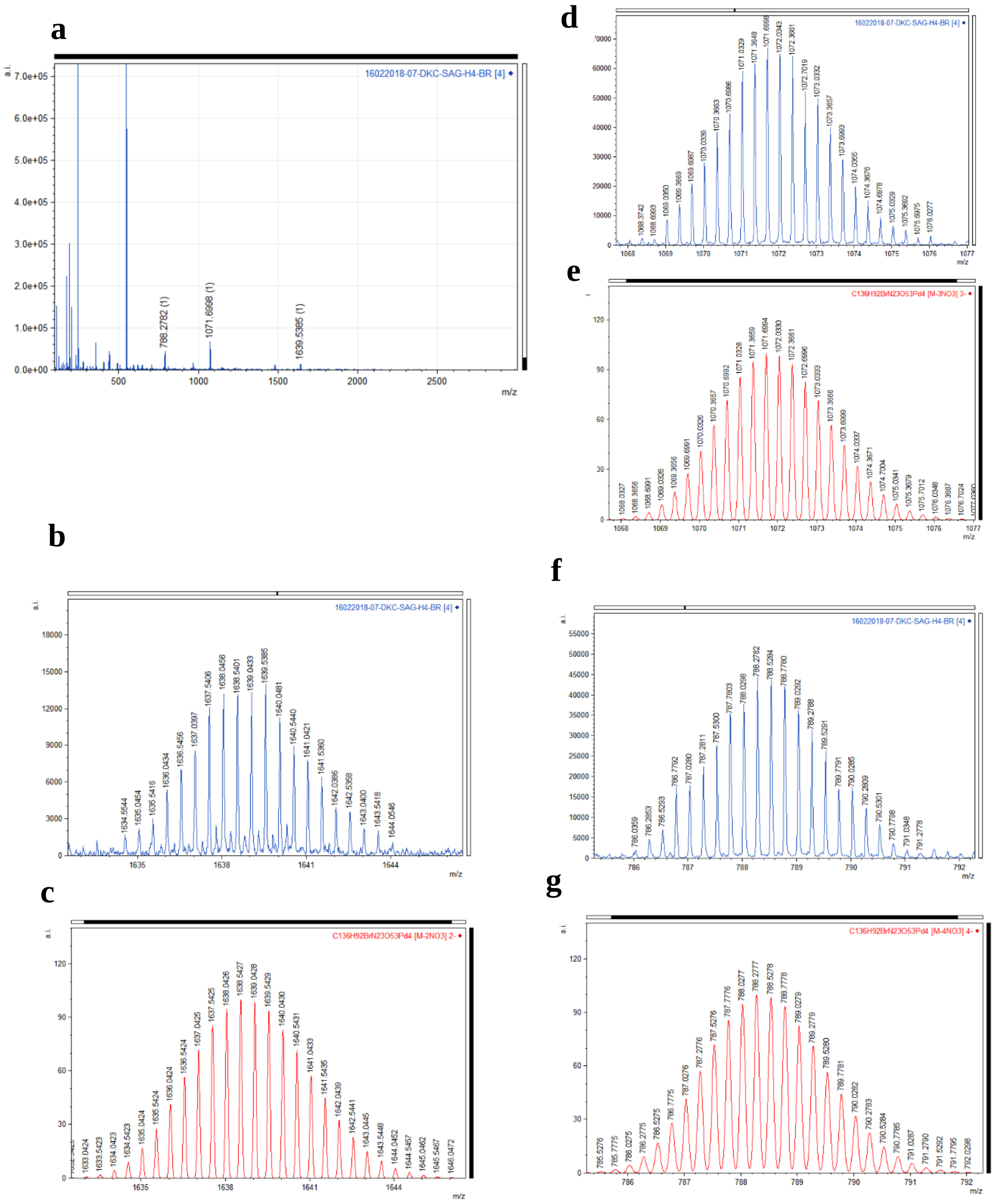

Supplementary Figure 61. ESI mass spectrum for [Br⊂Pd_4(**L3**)$_2$(**L5**)$_4$]$(NO_3)_7$, **4d** showing **a** full spectrum, **b** experimental isotopic pattern for [**4d**–$2NO_3$]$^{2+}$, **c** theoretical isotopic pattern for [**4d**–$2NO_3$]$^{2+}$, **d** experimental isotopic pattern for [**4d**–$3NO_3$]$^{3+}$, **e** theoretical isotopic pattern for [**4d**–$4NO_3$]$^{4+}$, **f** experimental isotopic pattern for [**4d**–$4NO_3$]$^{4+}$, and **g** theoretical isotopic pattern for [**4d**–$4NO_3$]$^{4+}$.

Characterization of the complexes [X⊂Pd_4(L3)$_2$(L5)$_4$](BF_4)$_7$, 4a'-4d'

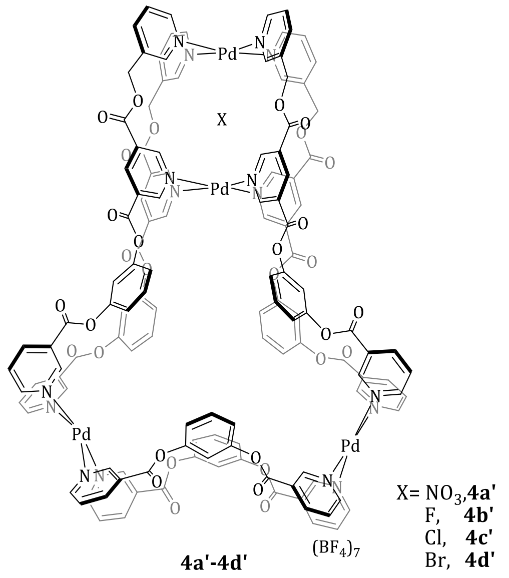

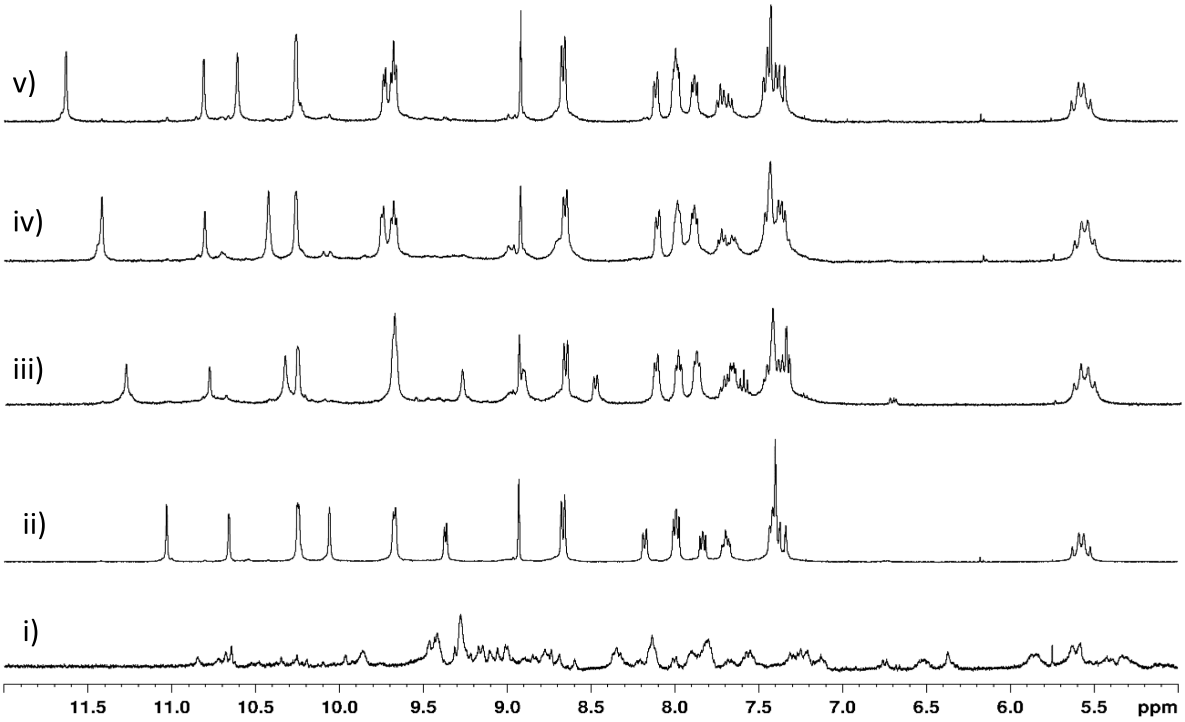

Supplementary Figure 62. 400 MHz partial ^{1}H NMR spectra in DMSO-d_6 for (i) oligomers formed due to complexation of Pd(BF_4)$_2$ with **L3** and **L5** at 4:2:4 ratio; upon addition of appropriate TBAX to the oligomer resulting in the formation of (ii) [NO_3⊂Pd_4(**L3**)$_2$(**L5**)$_4$](BF_4)$_7$, **4a'** (when X=NO_3); (iii) [F⊂Pd_4(**L3**)$_2$(**L5**)$_4$](BF_4)$_7$, **4b'** (when X=F); (iv) [Cl⊂Pd_4(**L3**)$_2$(**L5**)$_4$](BF_4)$_7$, **4c'** (when X=Cl) and (v) [Br⊂Pd_4(**L3**)$_2$(**L5**)$_4$](BF_4)$_7$, **4d'** (when X=Br).

Characterisation of the ligand, L6.

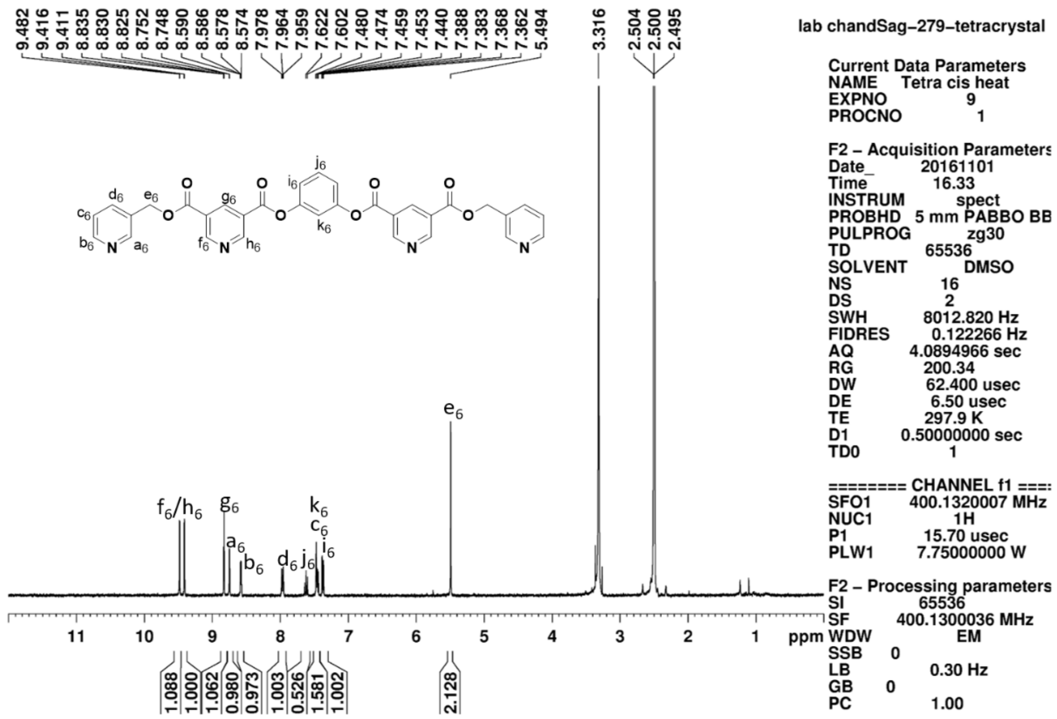

Supplementary Figure 63. ^{1}H NMR spectrum (500 MHz, DMSO-d_6, 300 K) for **L6**.

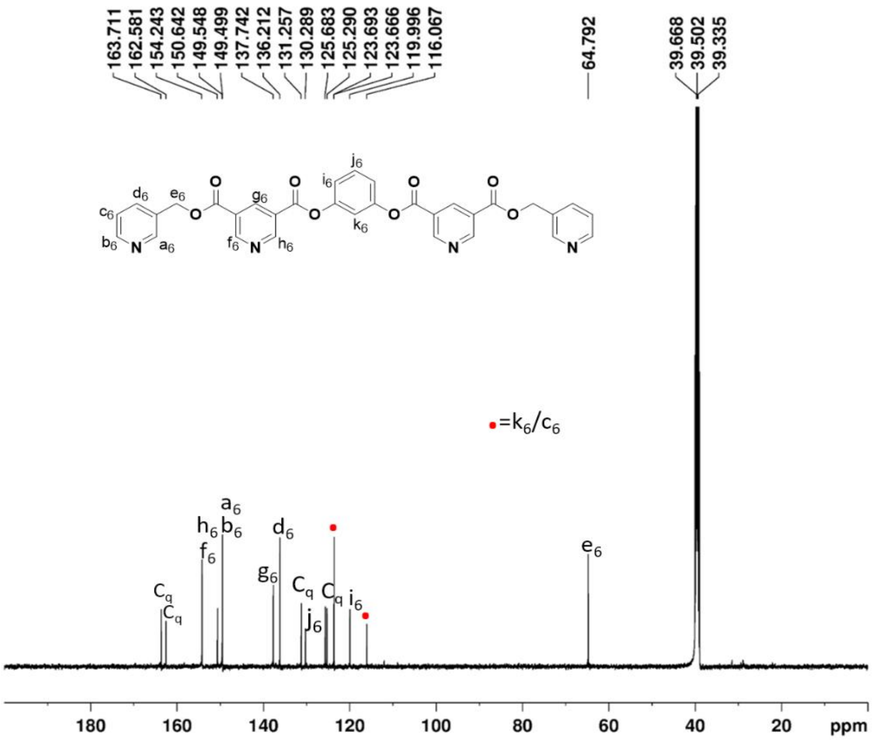

Supplementary Figure 64. ^{13}C NMR spectrum (125 MHz, DMSO-d_6, 300 K) for **L6**.

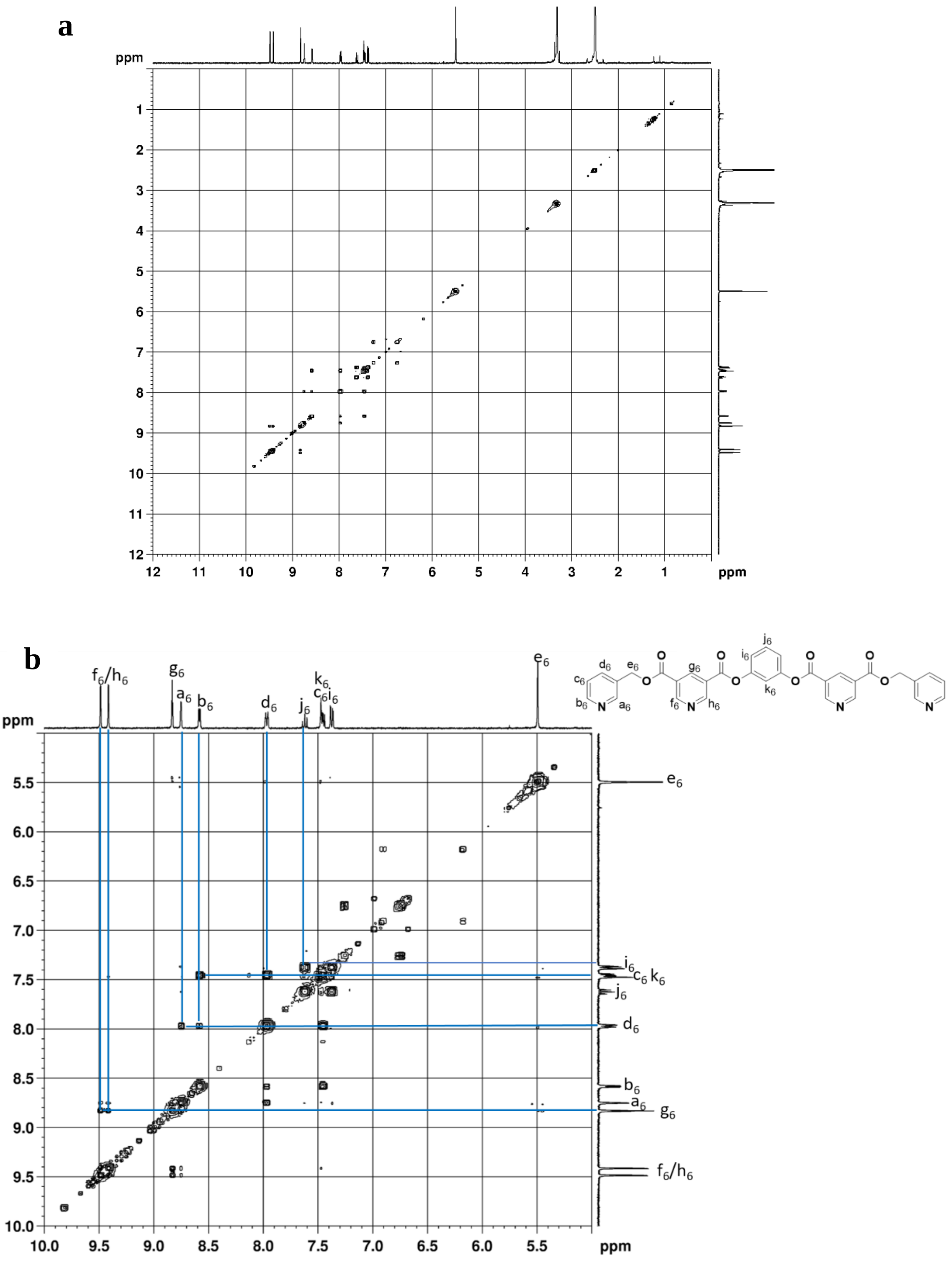

Supplementary Figure 65. H-H COSY spectrum (500 MHz, DMSO-d_6, 300 K) for **a** the ligand **L6**, and **b** expansion of the spectrum.

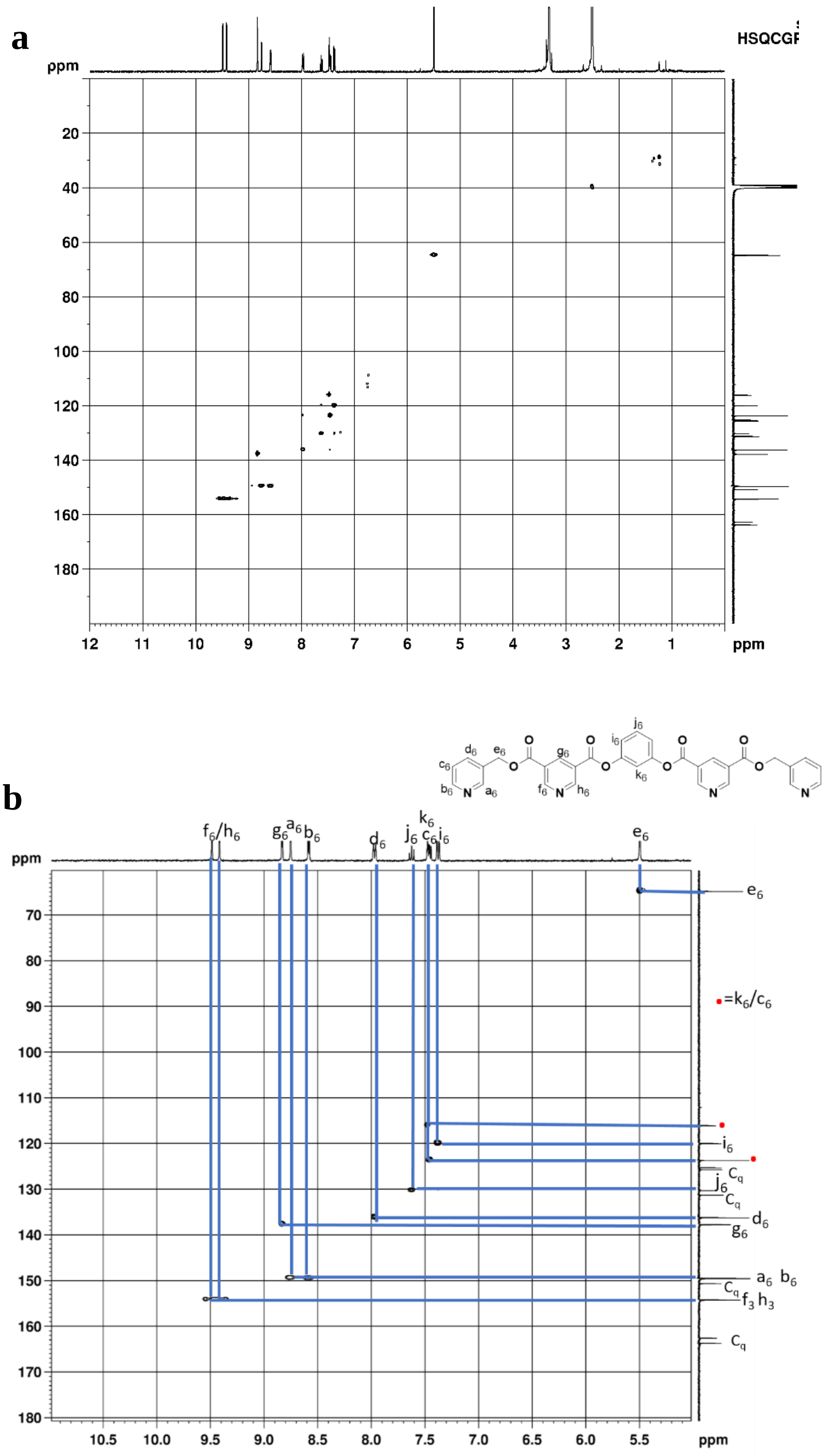

Supplementary Figure 66. C-H COSY spectrum (500 MHz, DMSO-d_6, 300 K) for **a** the ligand **L6**, and **b** expansion of the spectrum.

a

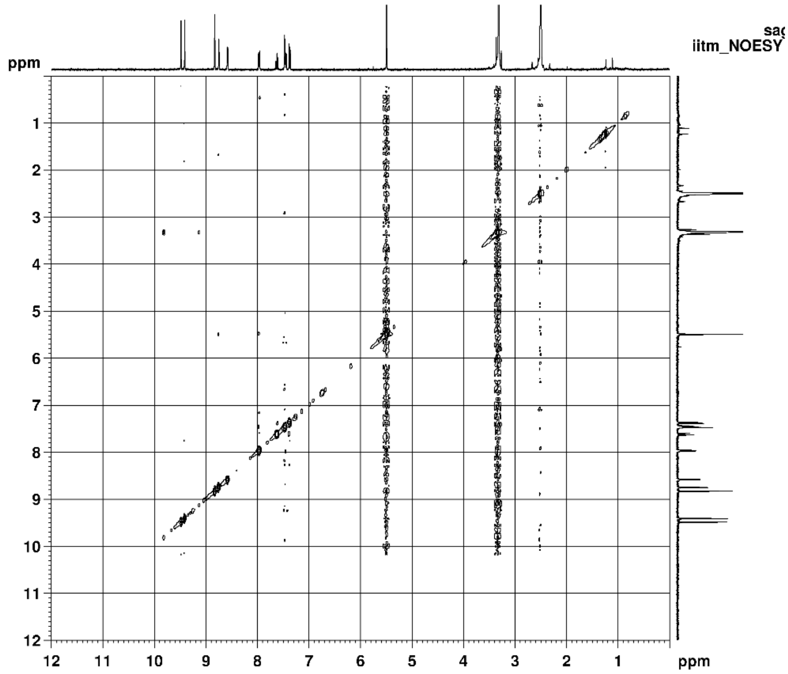

b

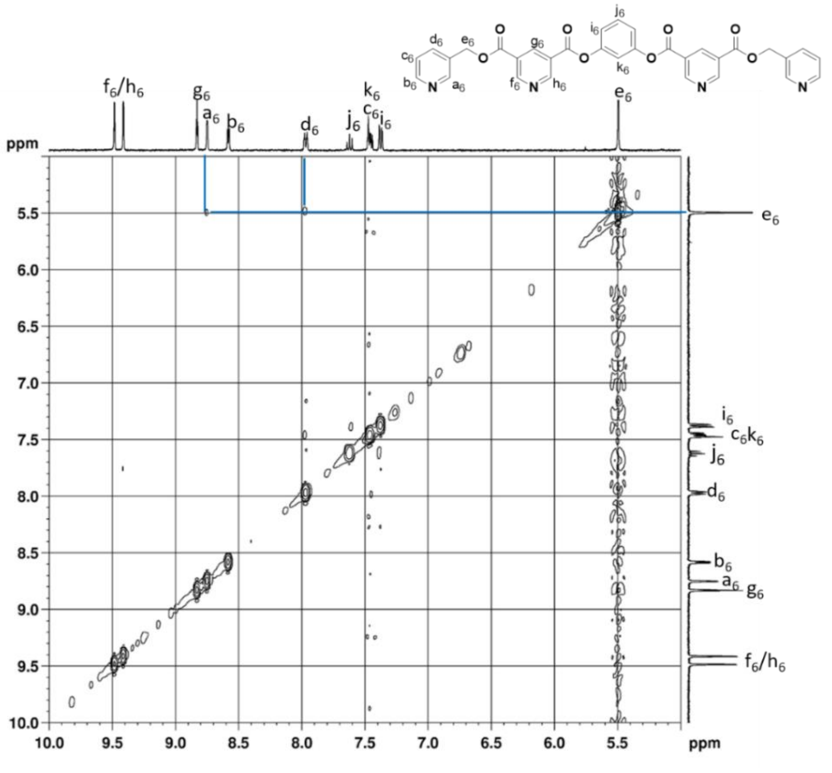

Supplementary Figure 67. NOESY spectrum (500 MHz, DMSO-d_6, 300 K) for **a** the ligand **L6**, and **b** expansion of the spectrum.

Compound Details

Cpd. 1: C32 H22 N4 O8

Compound Spectra (overlaid)

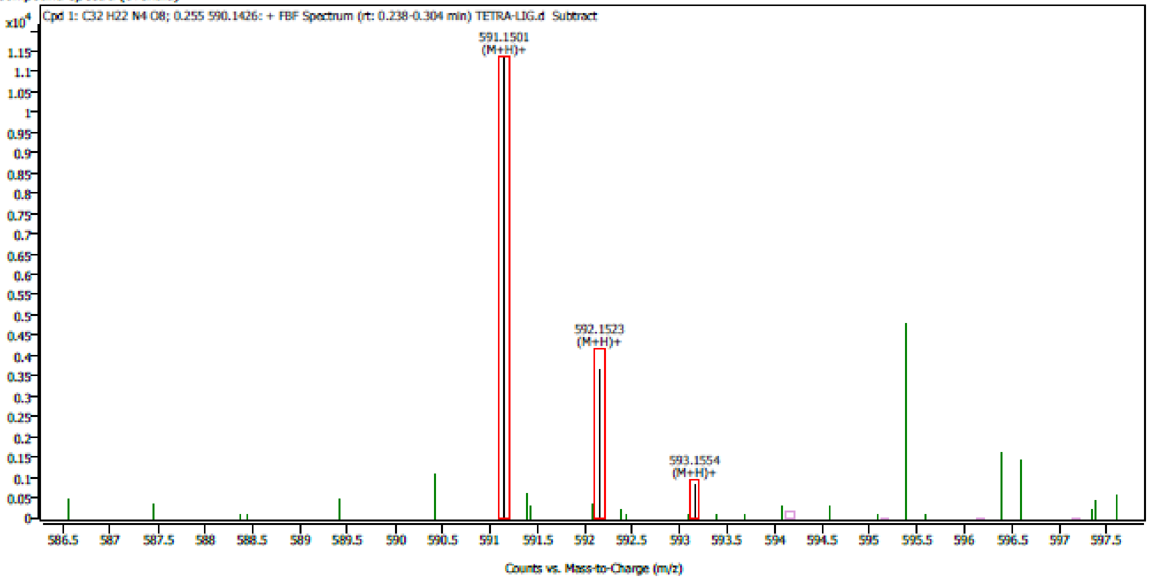

Compound ID Table

Cpd	Formula	Mass (Tgt)	Calc. Mass	Mass	Species	Diff(Tgt.ppm)	mDa
1	C32 H22 N4 O8	590.1438	590.1426	591.1501	(M+H)+	-1.95	-1.15

Supplementary Figure 68. ESI mass spectrum for the ligand **L6**.

Monitoring the formation of the complex [$(NO_3)_2\subset Pd_5(L5)_4(L6)_2$]$(NO_3)_8$, 5a at room temperature

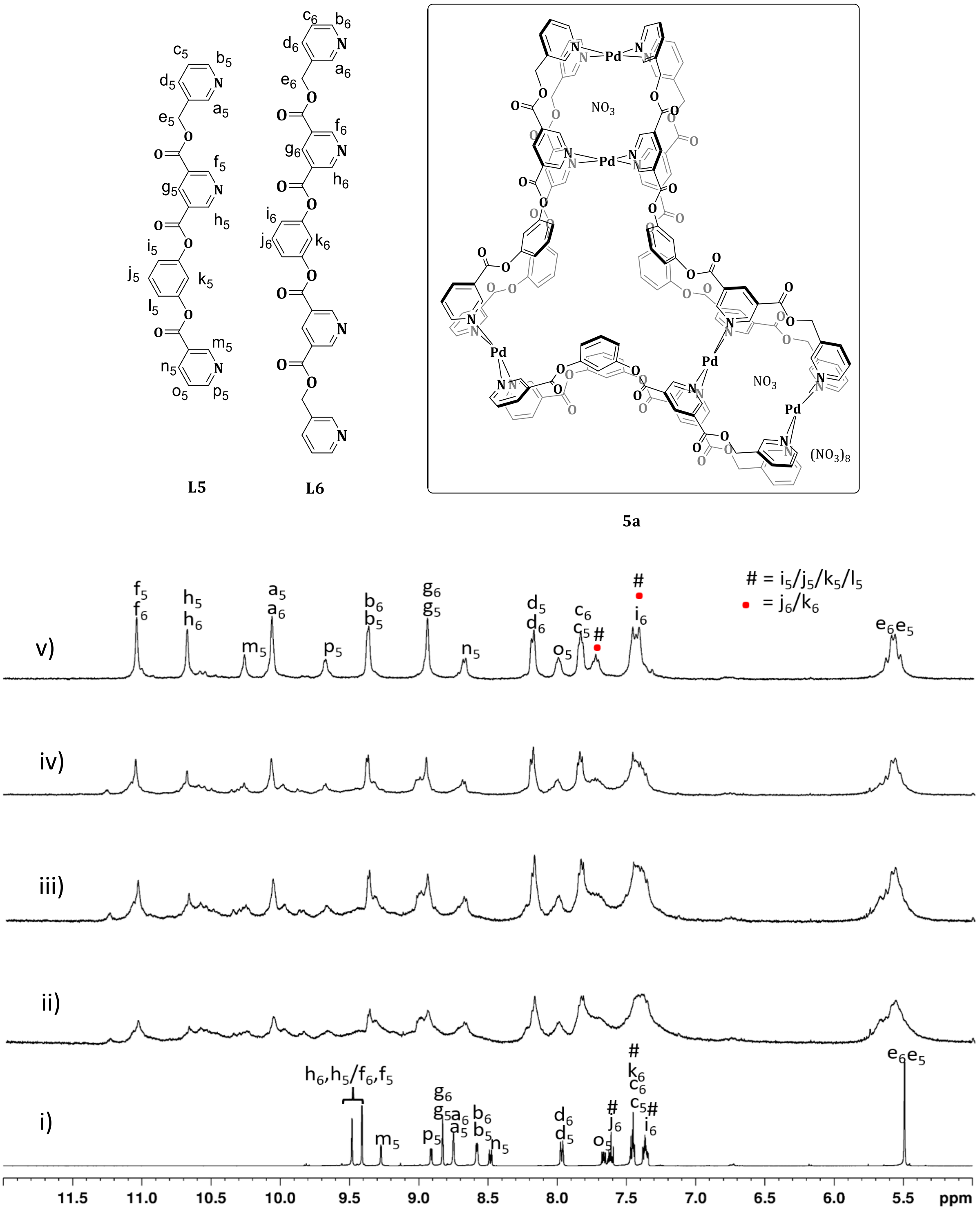

Supplementary Figure 69. 400 MHz partial ^{1}H NMR spectra (400 MHz, DMSO-d_6, 300 K) of: (i) mixture of **L5** and **L6** at 4:2 ratio, (ii)-(v) monitoring the reaction of **L5**, **L6** and $Pd(NO_3)_2$ in 4 : 2: 5 ratio (ii) immediately after mixing (iii) 30 min., (iv) 2 h, (v) 4 h complex [$(NO_3)_2\subset Pd_5$(**L5**)$_4$(**L6**)$_2$]$(NO_3)_8$, **5a**.

Monitoring the formation of the complex [$(NO_3)_2$⊂Pd_5(L5)$_4$(L6)$_2$]$(NO_3)_8$, 5a at 70 °C

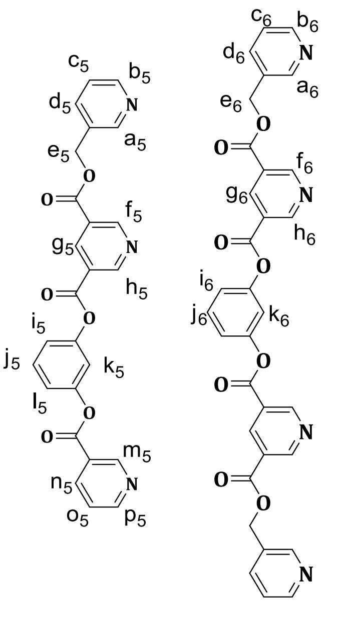

L5 **L6**

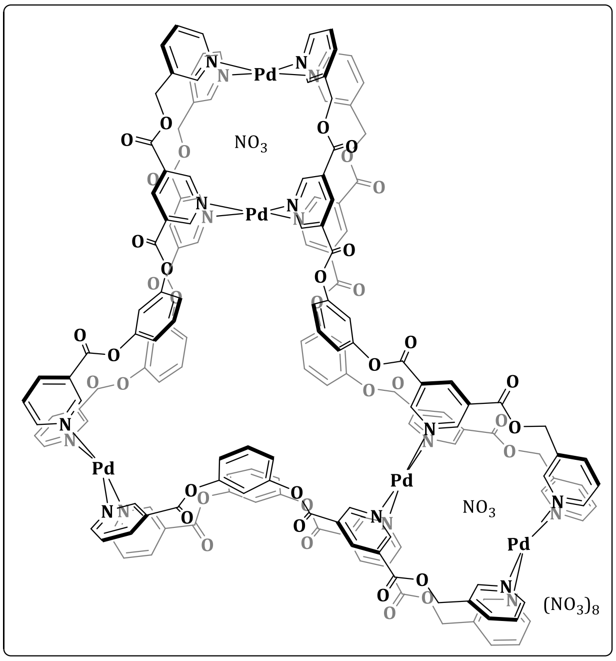

5a

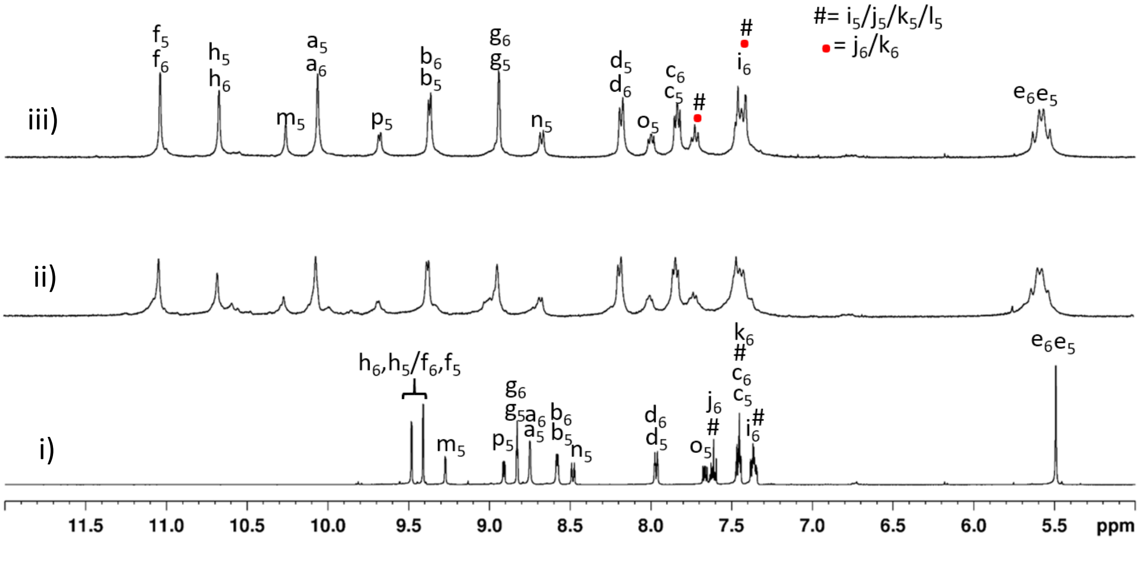

Supplementary Figure 70. 400 MHz partial ^{1}H NMR spectra (400 MHz, DMSO-d_6, 300 K) of: (i) mixture of **L5** and **L6** at 4:2 ratio, (ii)-(iii) monitoring the reaction of **L5**, **L6** and $Pd(NO_3)_2$ in 4 : 2: 5 ratio after (ii) 30 min of heating; (iii) 60 min of heating, complex [$(NO_3)_2$⊂Pd_5(**L5**)$_4$(**L6**)$_2$]$(NO_3)_8$, **5a**.

Characterisation of the complex [$(NO_3)_2$⊂$Pd_5(L5)_4(L6)_2$]$(NO_3)_8$, 5a.

a

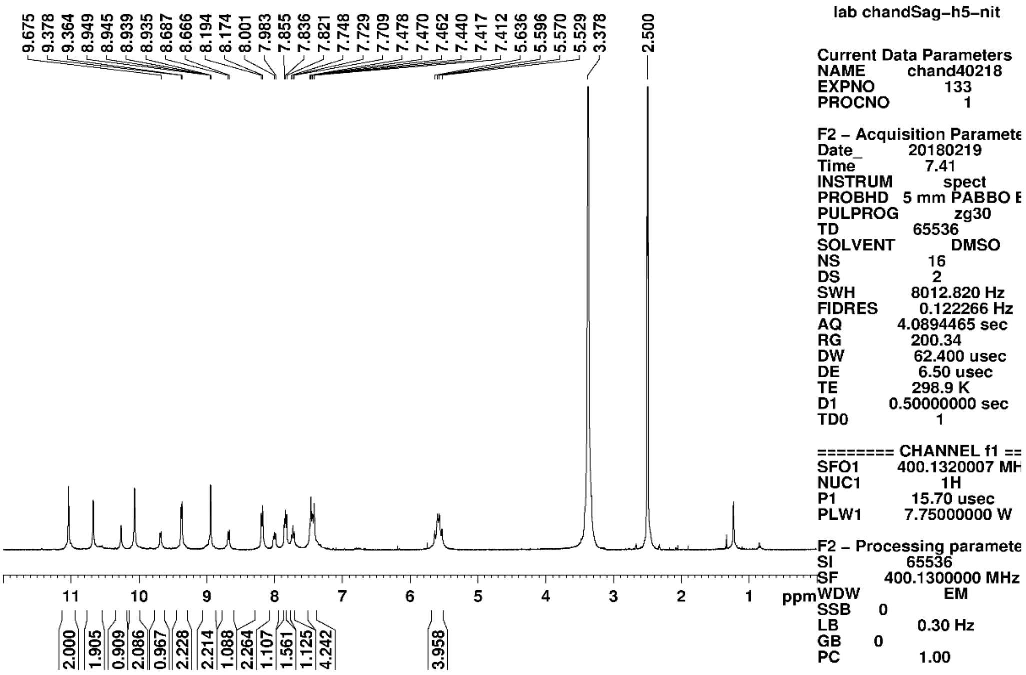

b

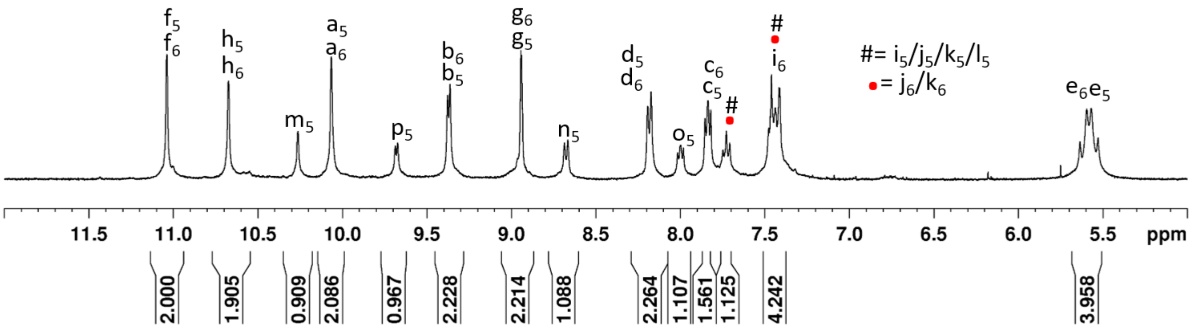

Supplementary Figure 71. ^{1}H NMR spectrum (500 MHz, DMSO-d_6, 300 K) for **a** the complex **5a** (Concentration: 10 mM with respect to palladium(II) source), and **b** expansion of the spectrum.

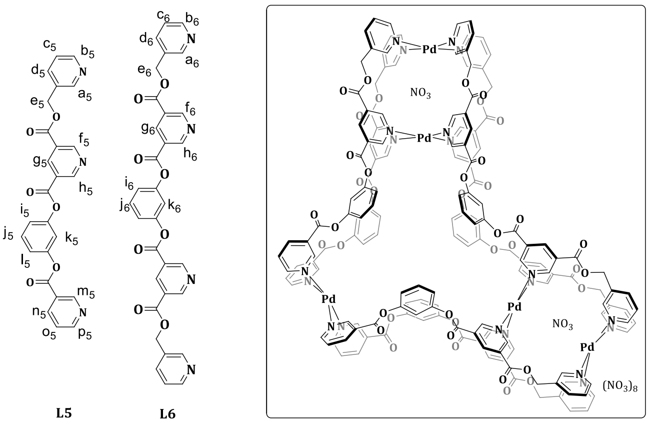

L5 L6

5a

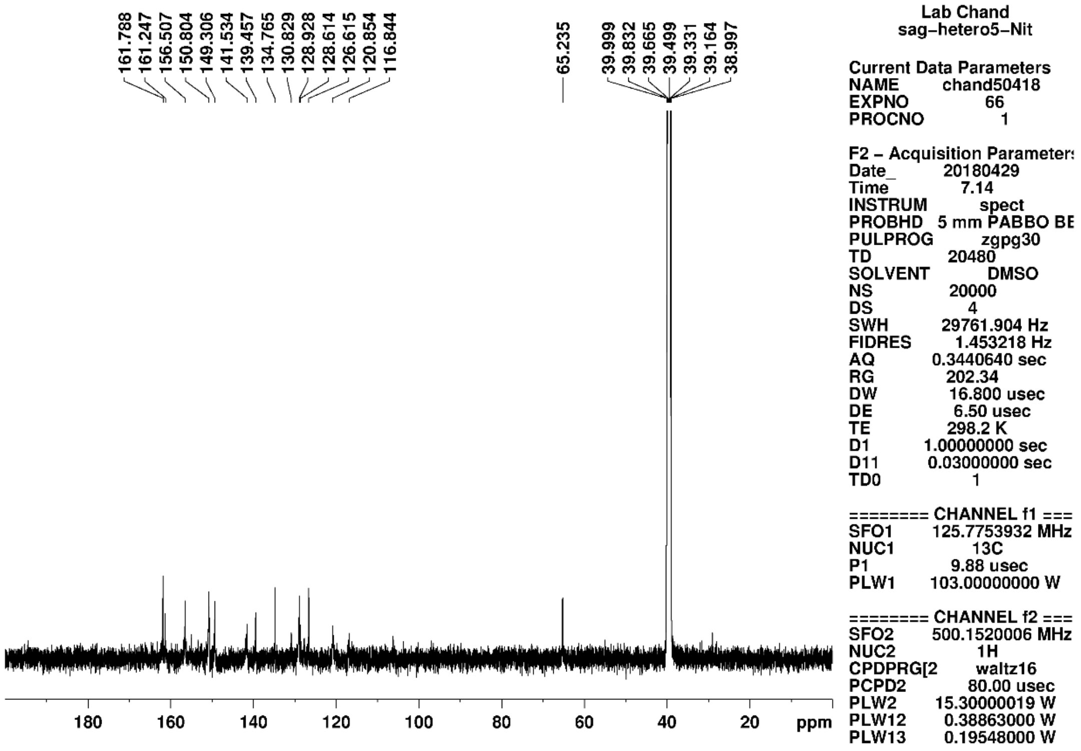

Supplementary Figure 72. ^{13}C NMR spectrum (125 MHz, DMSO-d_6, 300 K) for **5a**, some of the peaks are overlapped.

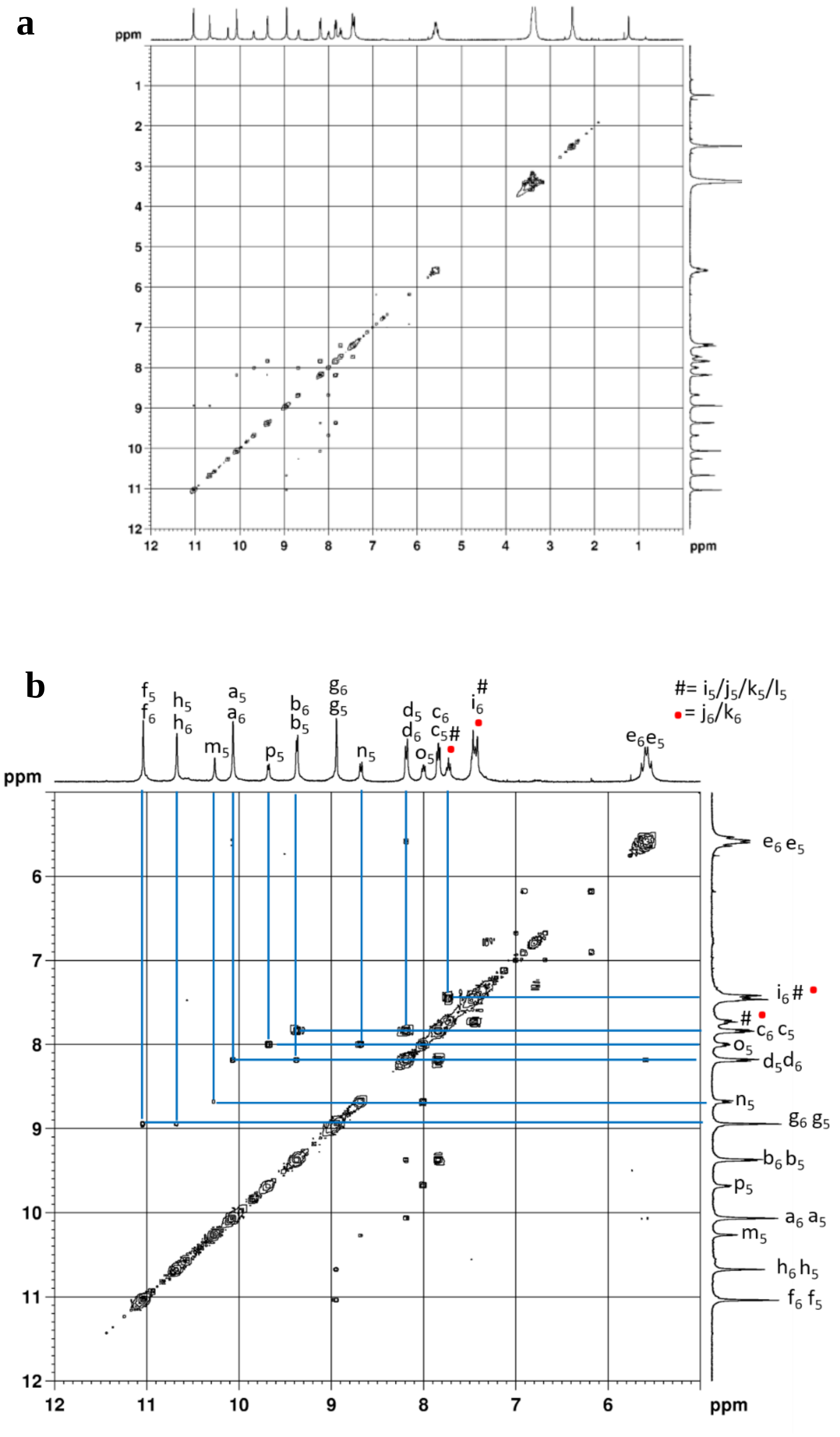

Supplementary Figure 73. H-H COSY spectrum (500 MHz, DMSO-d_6, 300 K) for **a** the complex **5a**, and **b** expansion of the spectrum.

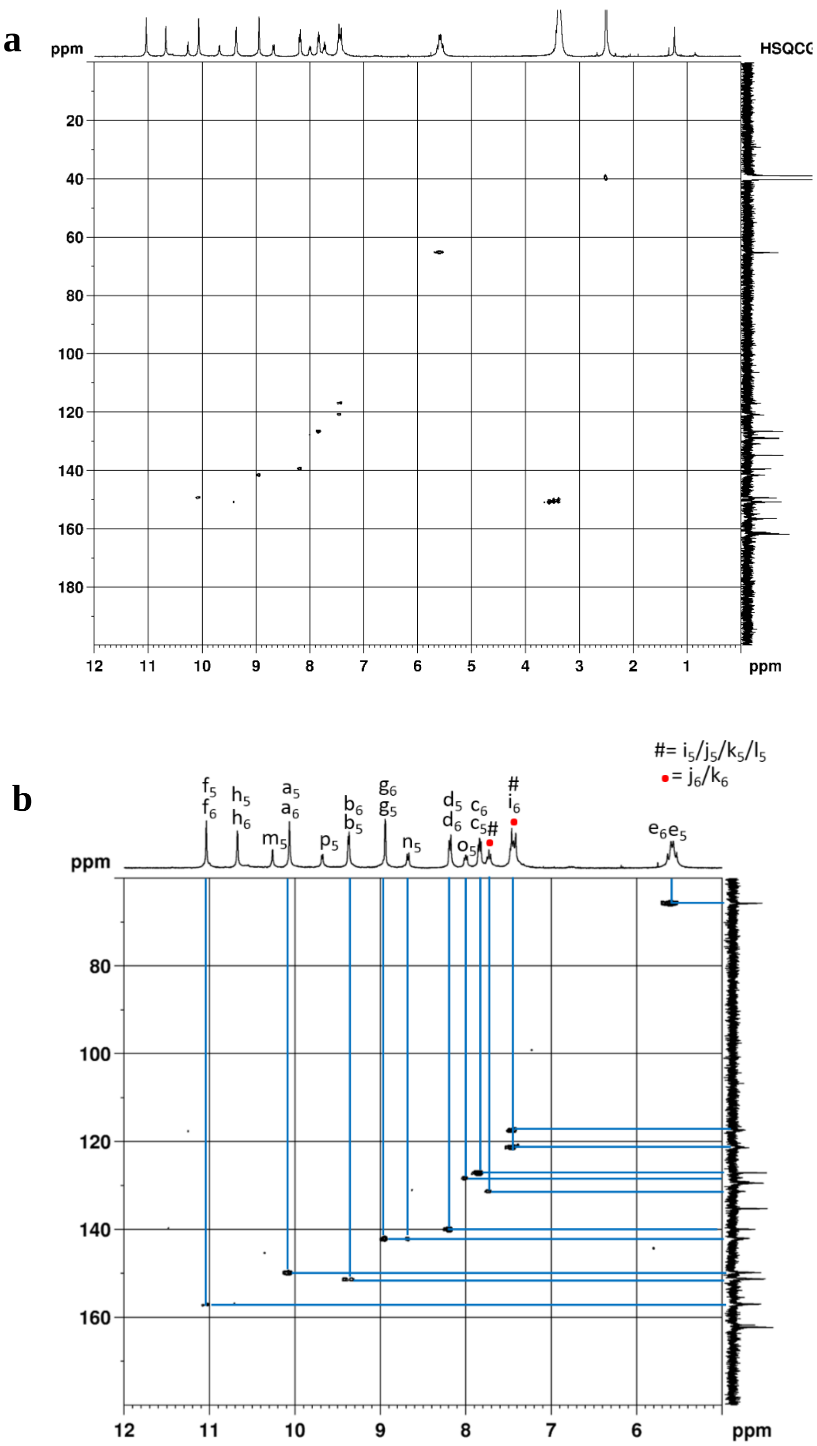

Supplementary Figure 74. C-H COSY spectrum (500 MHz, DMSO-d_6, 300 K) for **a** the complex **5a**, and **b** expansion of the spectrum.

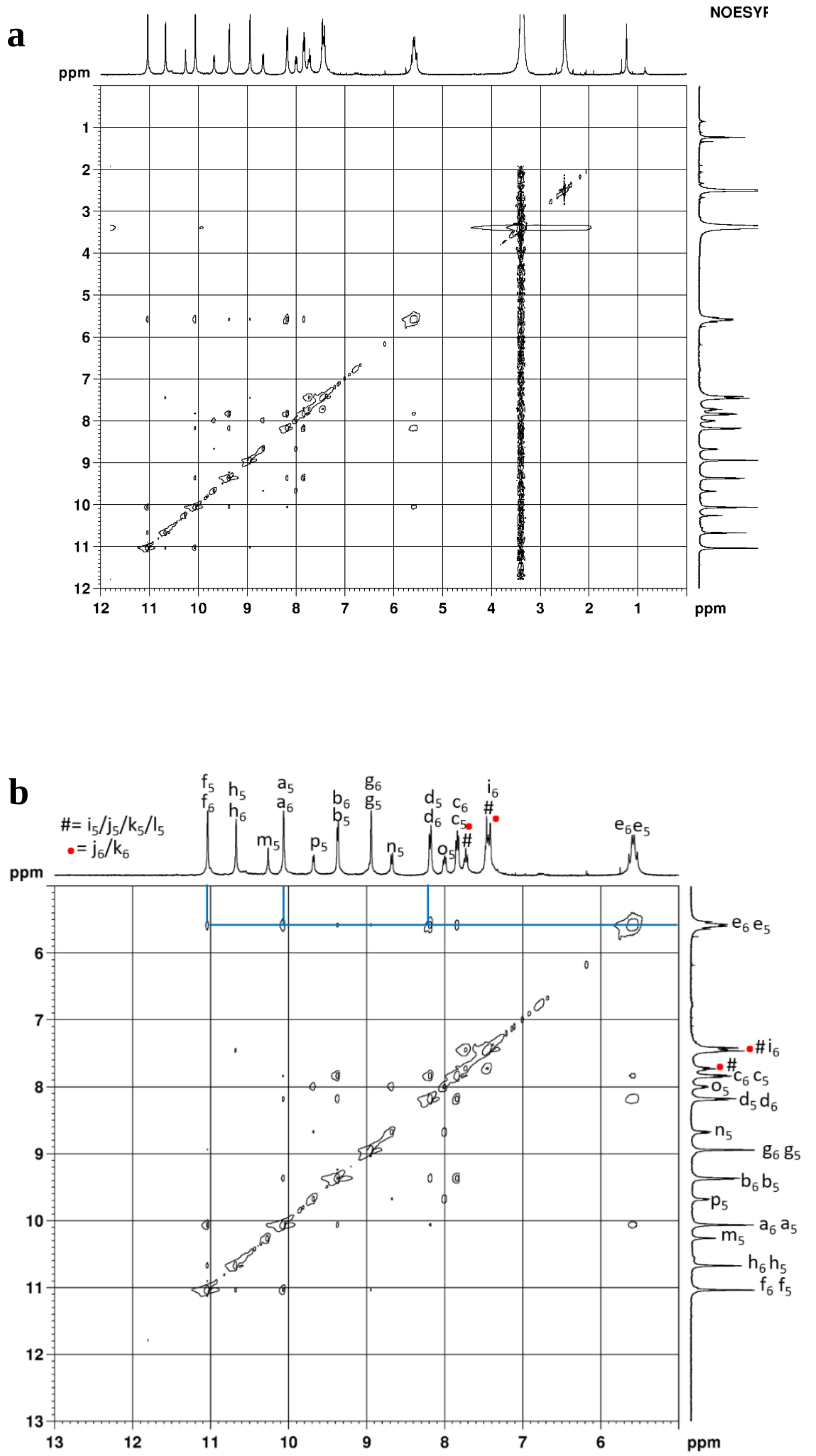

Supplementary Figure 75. NOESY spectrum (500 MHz, DMSO-d_6, 300 K) for **a** the complex **5a**, and **b** expansion of the spectrum.

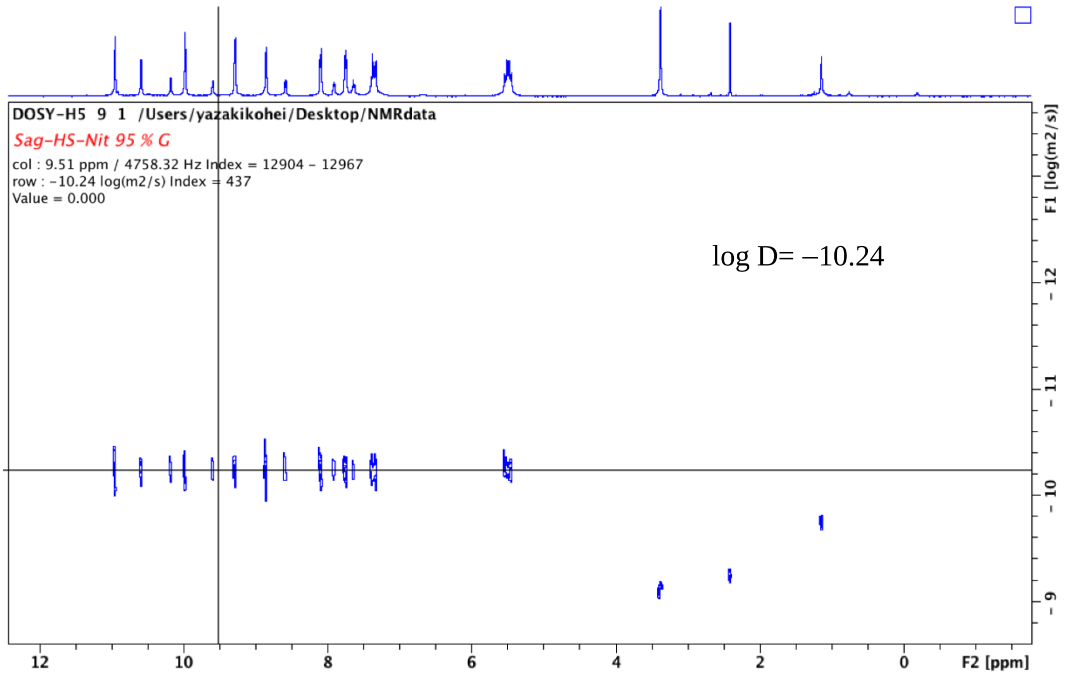

Supplementary Figure 76. ^{1}H DOSY NMR spectrum for the complex $[(NO_3)_2 \subset Pd_5(\mathbf{L5})_4(\mathbf{L6})_2](NO_3)_8$, **5a** showing single band with diffusion co-efficient (D) value of 5.75 x 10^{-11} m^2 s^{-1}.

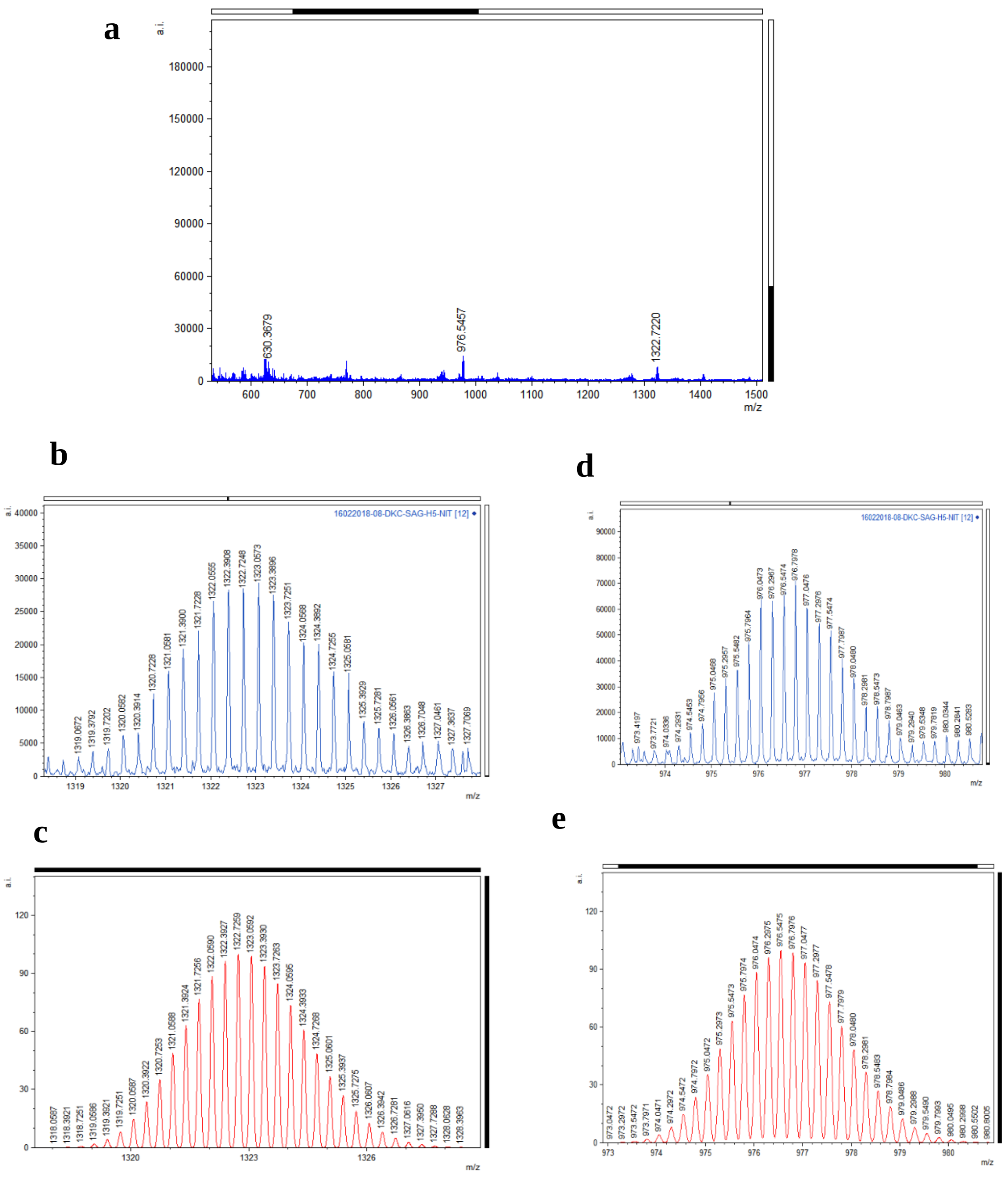

Supplementary Figure 77. ESI mass spectrum for $[(NO_3)_2\subset Pd_5(\mathbf{L5})_4(\mathbf{L6})_2](NO_3)_8$, **5a** showing **a** full spectrum, **b** experimental isotopic pattern for $[\mathbf{5a}–3NO_3]^{3+}$, **c** theoretical isotopic pattern for $[\mathbf{5a}–3NO_3]^{3+}$, **d** experimental isotopic pattern for $[\mathbf{5a}–4NO_3]^{4+}$, and **e** theoretical isotopic pattern for $[\mathbf{5a}–4NO_3]^{4+}$.

Characterization of the complexes [(X)$_2$⊂Pd$_5$(L5)$_4$(L6)$_2$](NO$_3$)$_8$, 5b-5d

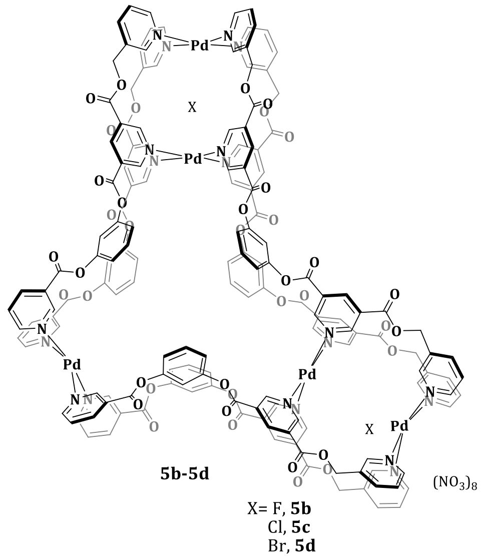

Portionwise addition of TBAF to [(NO$_3$)$_2$⊂Pd$_5$(L5)$_4$(L6)$_2$](NO$_3$)$_8$, 5a for the synthesis of the complex [(F)$_2$⊂Pd$_5$(L5)$_4$(L6)$_2$](NO$_3$)$_8$, 5b

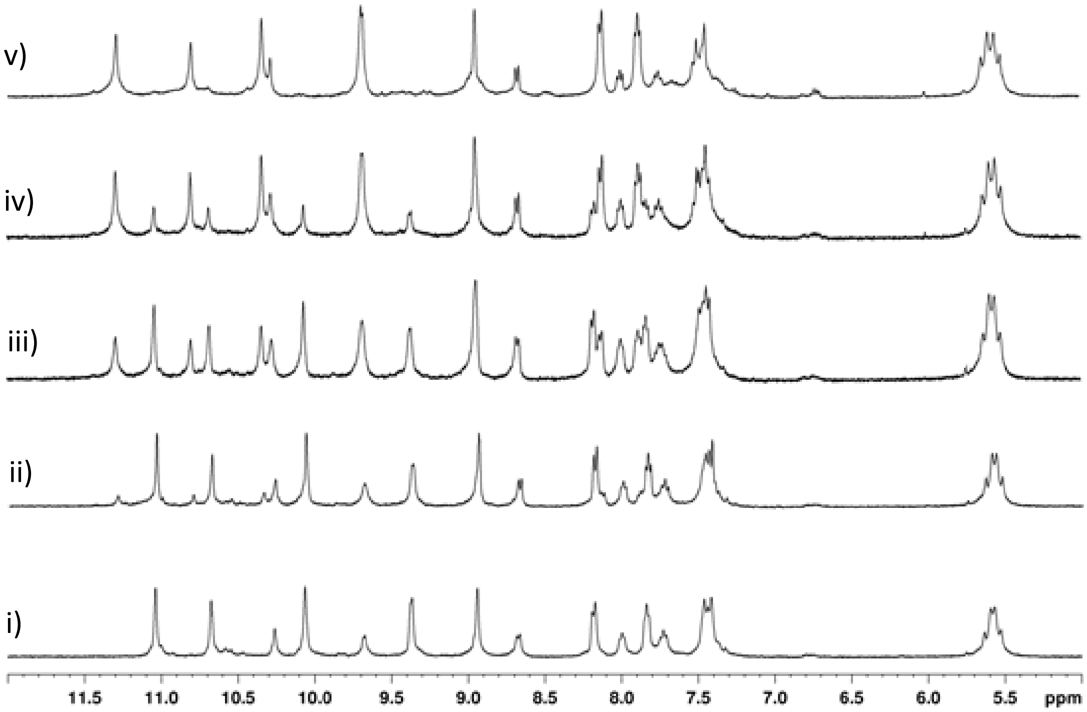

Supplementary Figure 78. Partial ^{1}H NMR spectra (400 MHz, DMSO-d_6, 300 K) for (i) [(NO$_3$)$_2$⊂Pd$_5$(**L5**)$_4$(**L6**)$_2$](NO$_3$)$_8$, **5a**; portionwise addition of TBAF to **5a** with (ii) 0.5 equiv. (iii) 1 equiv. (iv) 1.5 equiv. Exclusive formation of (v) [(F)$_2$⊂Pd$_5$(**L5**)$_4$(**L6**)$_2$](NO$_3$)$_8$, **5b** upon addition of 2 equiv.

Portionwise addition of TBACl to $[(NO_3)_2 \subset Pd_5(L5)_4(L6)_2](NO_3)_8$, 5a for the synthesis of the complex $[(Cl)_2 \subset Pd_5(L5)_4(L6)_2](NO_3)_8$, 5c

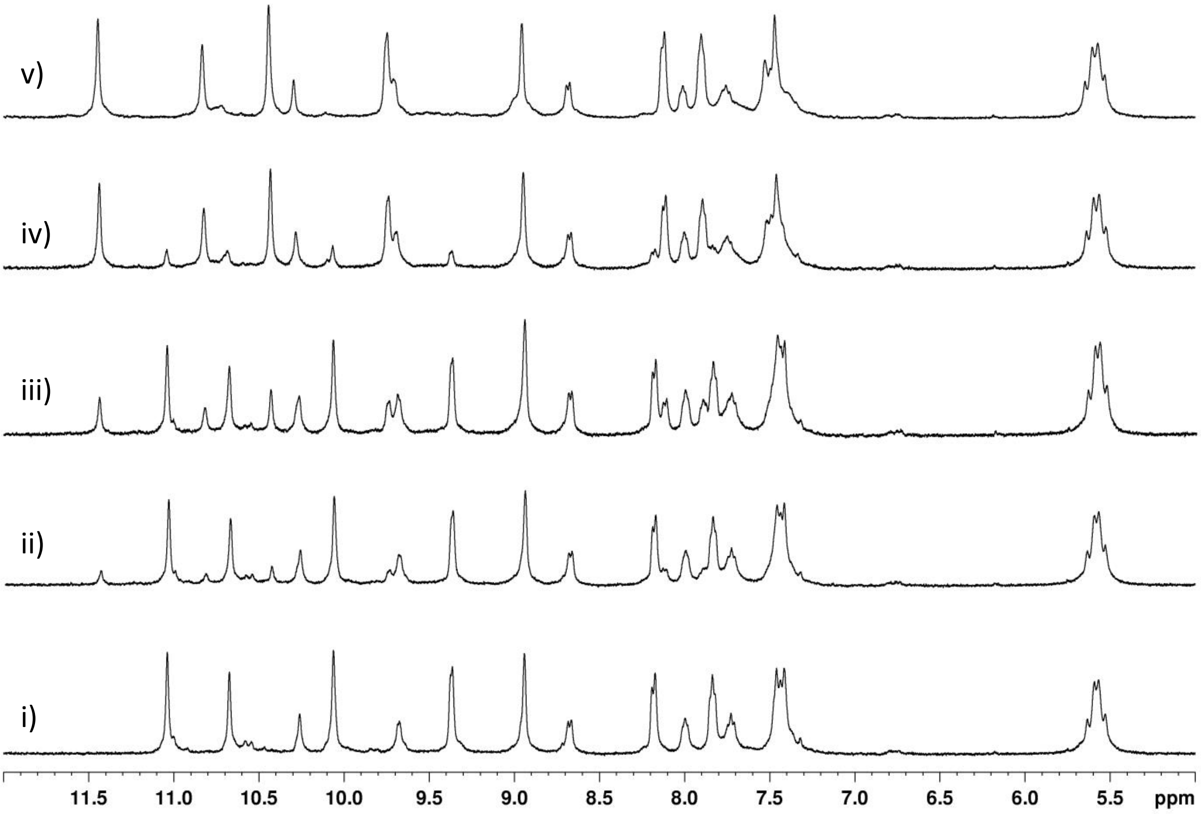

Supplementary Figure 79. Partial ^{1}H NMR spectra (400 MHz, DMSO-*d*6, 300 K) for (i) $[(NO_3)_2 \subset Pd_5(\mathbf{L5})_4(\mathbf{L6})_2](NO_3)_8$, **5a**; portionwise addition of TBACl to **5a** (ii) 0.5 equiv. (iii) 1 equiv. (iv) 1.5 equiv. Exclusive formation of (v) $[(Cl)_2 \subset Pd_5(\mathbf{L5})_4(\mathbf{L6})_2](NO_3)_8$, **5c** upon addition of 2 equiv.

Portionwise addition of TBABr to $[(NO_3)_2\subset Pd_5(L5)_4(L6)_2](NO_3)_8$, 5a for the synthesis of the complex $[(Br)_2\subset Pd_5(L5)_4(L6)_2](NO_3)_8$, 5d

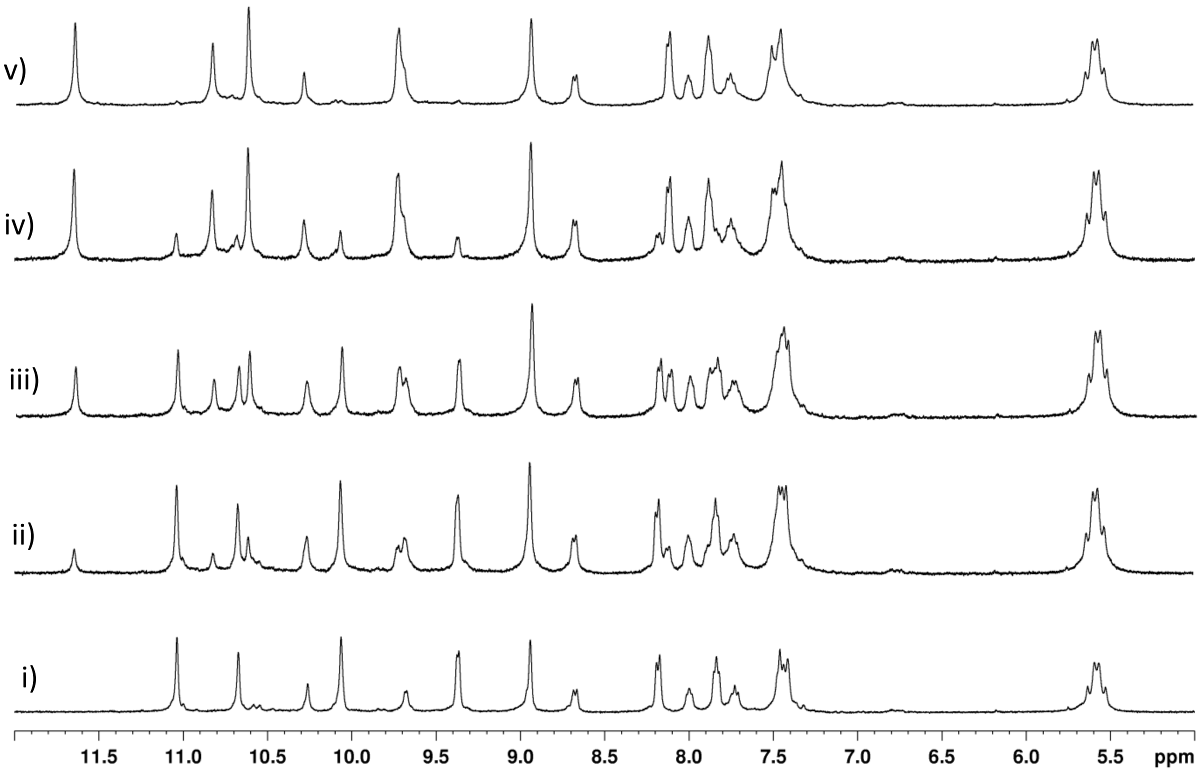

Supplementary Figure 80. Partial ^{1}H NMR spectra (400 MHz, DMSO-d_6, 300 K) for (i) $[(NO_3)_2\subset Pd_5(\mathbf{L5})_4(\mathbf{L6})_2](NO_3)_8$, **5a**; portionwise addition of TBABr to **5a** (ii) 0.5 equiv. (iii) 1 equiv. (iv) 1.5 equiv. Exclusive formation of (v) $[(Br)_2\subset Pd_5(\mathbf{L5})_4(\mathbf{L6})_2](NO_3)_8$, **5d** upon addition of 2 equiv.

Stacking diagram showing ^{1}H NMR spectra of 5a-5d

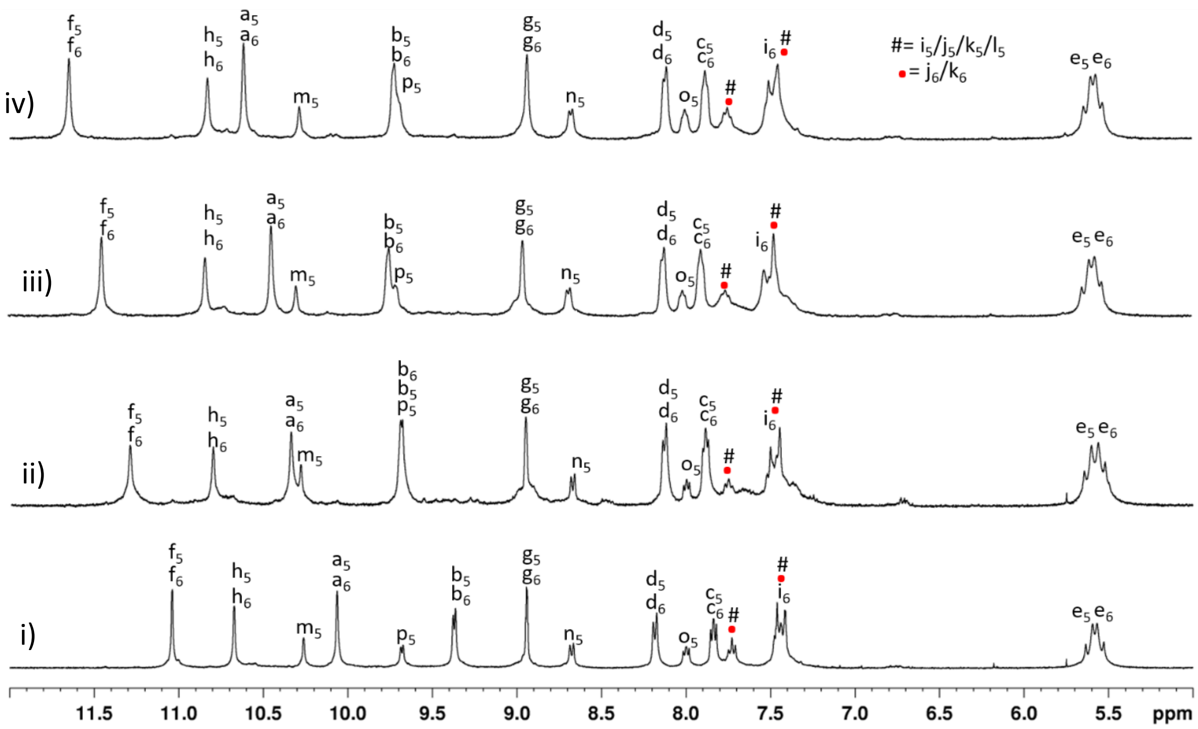

Supplementary Figure 81. 400 MHz partial ^{1}H NMR spectra in DMSO-d_6 for (i) $[(NO_3)_2{\subset}Pd_5$(**L5**)$_4$(**L6**)$_2](NO_3)_8$, **5a**; (ii) $[(F)_2{\subset}Pd_5$(**L5**)$_4$(**L6**)$_2](NO_3)_8$, **5b**; (iii) $[(Cl)_2{\subset}Pd_5$(**L5**)$_4$(**L6**)$_2](NO_3)_8$, **5c** and (iii) $[(Br)_2{\subset}Pd_5$(**L5**)$_4$(**L6**)$_2](NO_3)_8$, **5d**.

Supplementary Table 3. ^{1}H NMR chemical shift of selected protons in ppm for various complexes **5a-5d** (DMSO-d_6) for comparison with ligand **L5** and **L6**.

Proton	**L5/L6**	$[(NO_3)_2{\subset}Pd_5$(**L5**)$_4$(**L6**)$_2](NO_3)_8$, **5a**	$[(F)_2{\subset}Pd_5$(**L5**)$_4$(**L6**)$_2]$ $(NO_3)_8$, **5b**	$[(Cl)_2{\subset}Pd_5$(**L5**)$_4$(**L6**)$_2]$ $(NO_3)_8$, **5c**	$[(Br)_2{\subset}Pd_5$(**L5**)$_4$(**L6**)$_2]$ $(NO_3)_8$, **5d**
H_{a5}/H_{a6}	8.75/8.75	10.06/10.06	10.34/10.34	10.43/10.43	10.62/10.62
H_{b5}/H_{b6}	8.58/8.58	9.37/9.37	9.69/9.69	9.74/9.74	9.72/9.72
H_{f5}/H_{f6}	9.41**or**9.48 /9.41**or**9.48	11.04/11.04	11.29/11.29	11.44/11.44	11.65/11.65
H_{h5}/H_{h6}	9.41**or**9.48 /9.41**or**9.48	10.67/10.67	10.80/10.80	10.82/10.82	10.83/10.83

Characterization of the complexes $[(X)_2\subset Pd_5(L5)_2(L6)_4](NO_3)_8$, 5a'-5d'

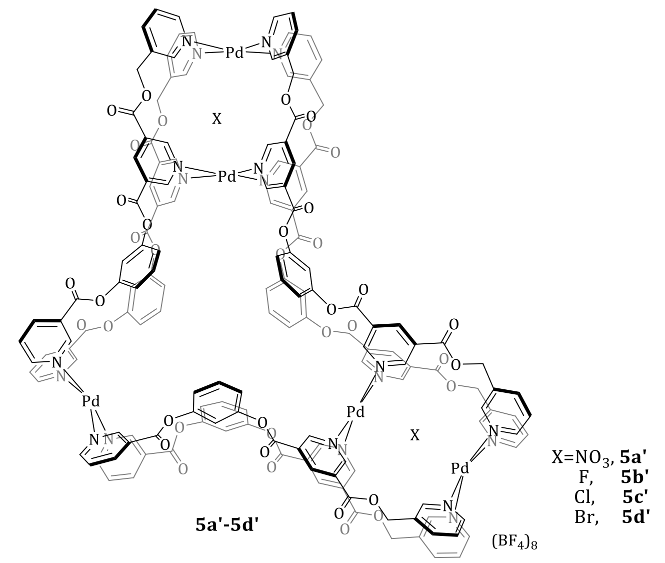

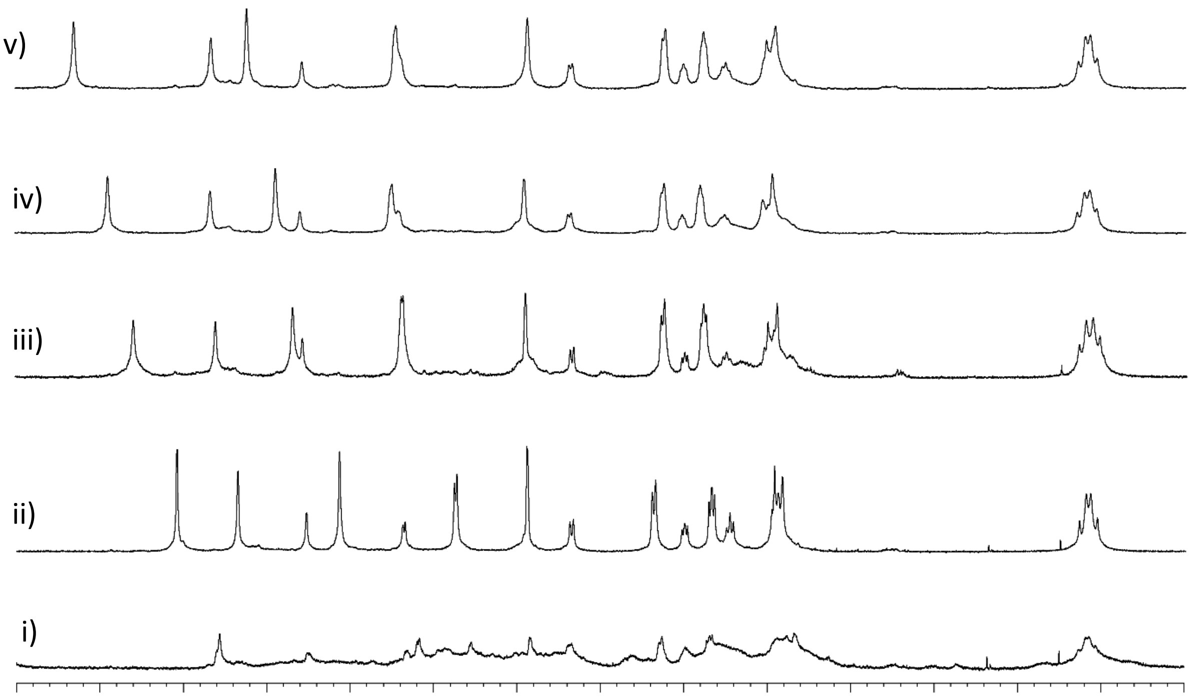

Supplementary Figure 82. 400 MHz partial ^{1}H NMR spectra in DMSO-d_6 for (i) oligomers formed upon complexation of $Pd(BF_4)_2$ with **L5** and **L6** at 5:4:2 ratio; upon addition of appropriate TBAX to the oligomer resulting in the formation of (ii) $[(NO_3)_2\subset Pd_5($**L5**$)_4($**L6**$)_2](BF_4)_8$, **5a'** (when X=NO_3); (iii) $[(F)_2\subset Pd_5($**L5**$)_4($**L6**$)_2](BF_4)_8$, **5b'** (when X=F); (iv) $[(Cl)_2\subset Pd_5($**L5**$)_4($**L6**$)_2](BF_4)_8$, **5c'** (when X=Cl) and (v) $[(Br)_2\subset Pd_5($**L5**$)_4($**L6**$)_2](BF_4)_8$, **5d'** (when X=Br).

Monitoring the formation of the complex $[(NO_3)_3\subset Pd_6(L6)_6](NO_3)_9$, 6a at room temperature

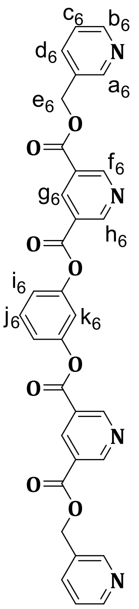

L6

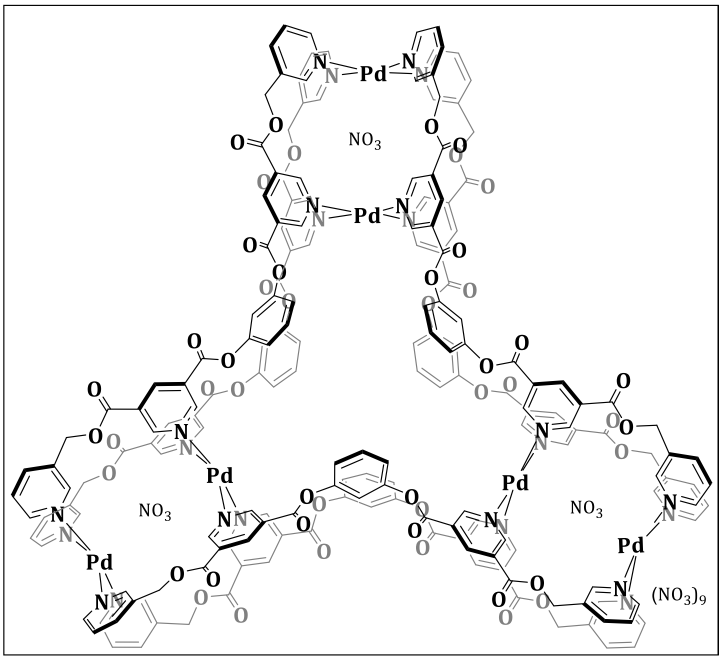

6a

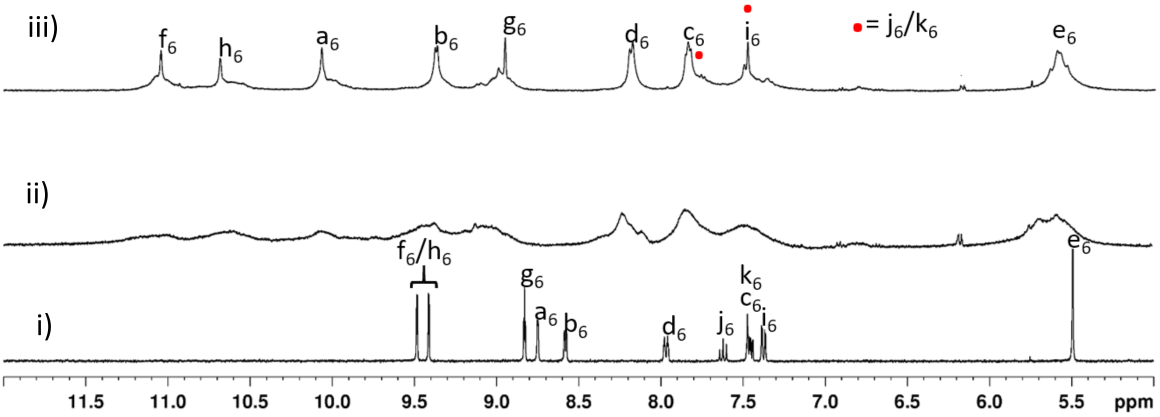

Supplementary Figure 83. 400 MHz partial ^{1}H NMR spectra (400 MHz, DMSO-d_6, 300 K) of: (i) ligand **L6**, (ii)-(iii) monitoring the reaction of **L6** and $Pd(NO_3)_2$ in 1:1 ratio after (ii) 30 min, (iii) 60 min, formation of complex $[(NO_3)_3\subset Pd_6(\mathbf{L6})_6](NO_3)_9$, **6a**.

Monitoring the formation of the complex [$(NO_3)_3 \subset Pd_6(L6)_6](NO_3)_9$, 6a at 70 °C

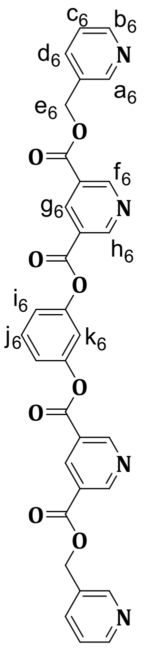

L6

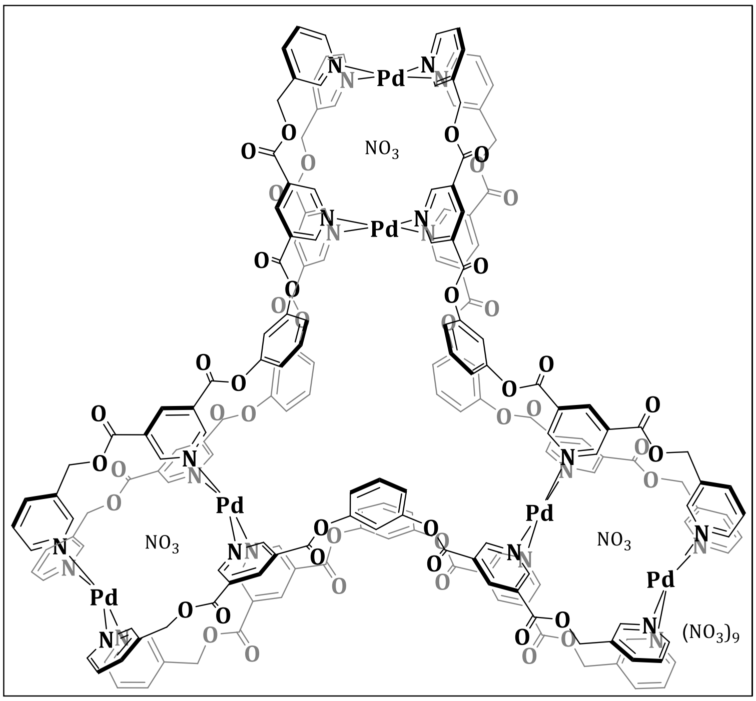

6a

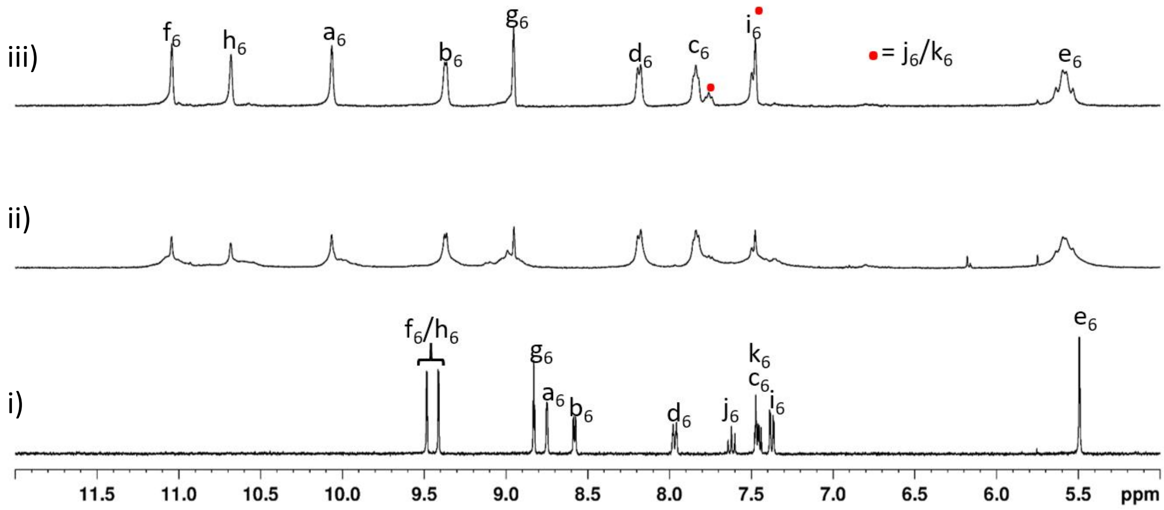

Supplementary Figure 84. 400 MHz partial ^{1}H NMR spectra (400 MHz, DMSO-d_6, 300 K) of: (i) ligand **L6**, (ii)-(iii) monitoring the reaction of **L6** and $Pd(NO_3)_2$ in 1:1 ratio after (ii) 10 min of heating; (iii) 20 min of heating, formation of complex [$(NO_3)_3 \subset Pd_6$(**L6**)$_6](NO_3)_9$, **6a**.

Characterisation of the complex [$(NO_3)_3 \subset Pd_6(L6)_6](NO_3)_9$, 6a.

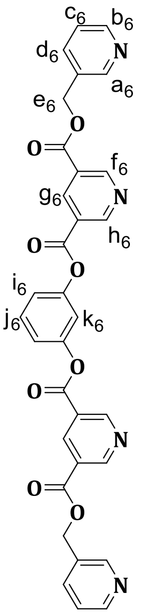

L6

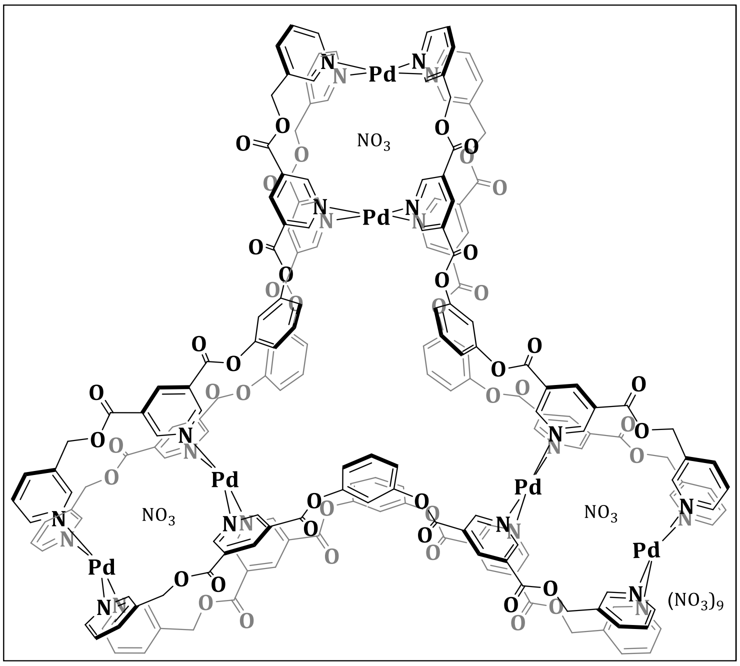

6a

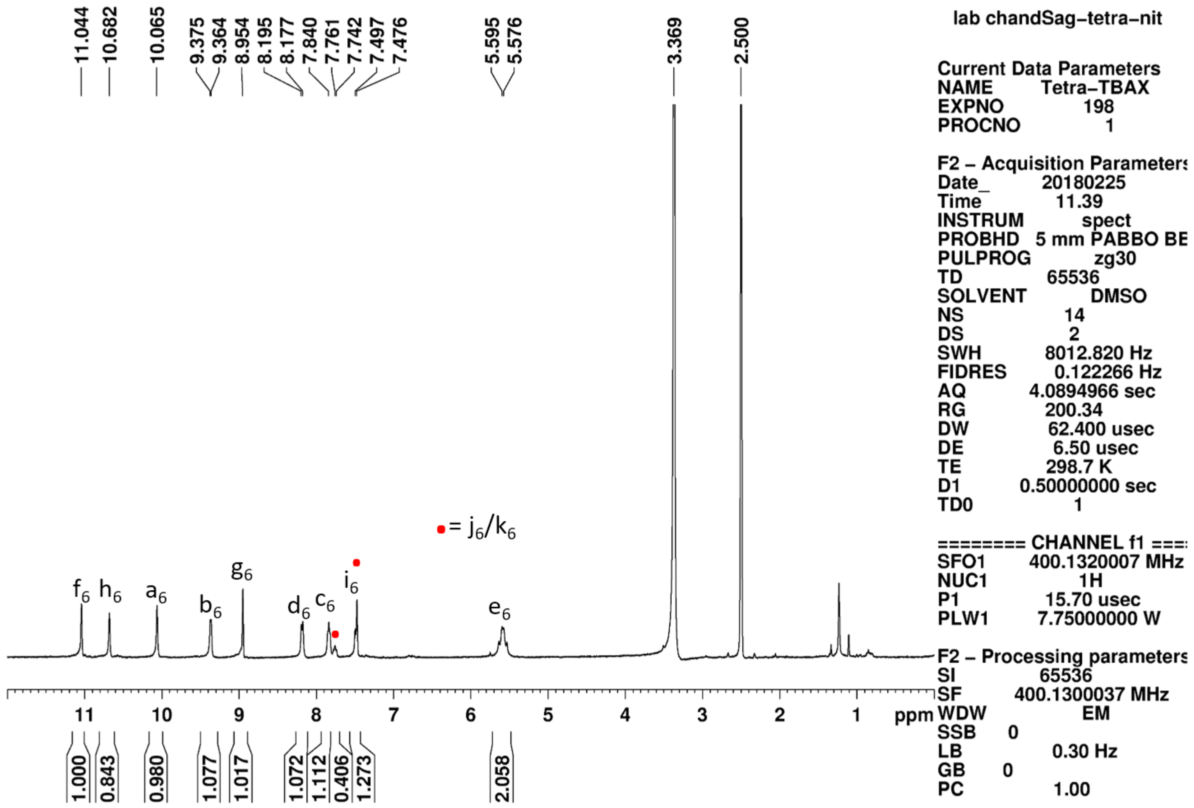

Supplementary Figure 85. ^1H NMR spectrum (500 MHz, DMSO-d_6, 300 K) for **6a**. (Concentration: 5 mM with respect to palladium(II) source).

a

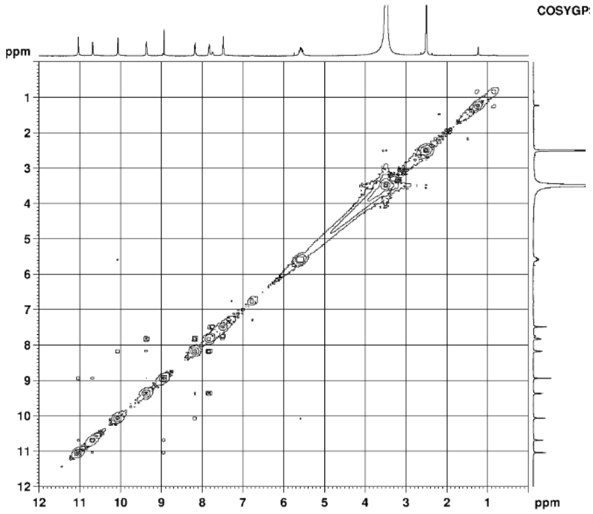

b

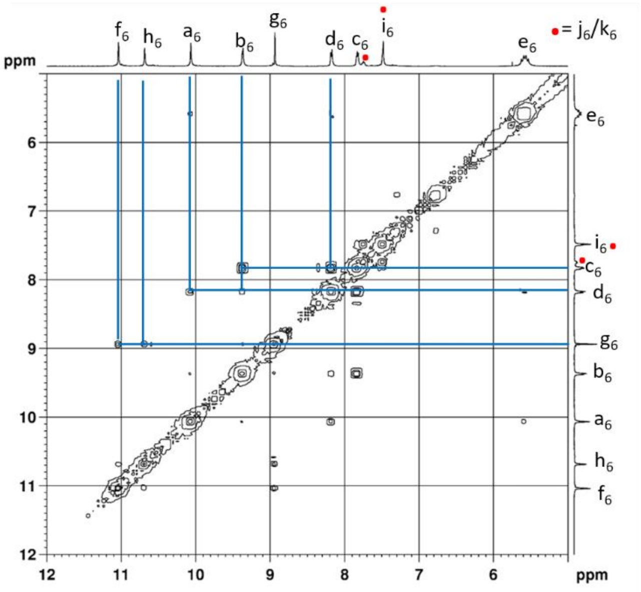

Supplementary Figure 86. H-H COSY spectrum (500 MHz, DMSO-*d*$_6$, 300 K) for **a** the complex **6a**, and **b** expansion of the spectrum.

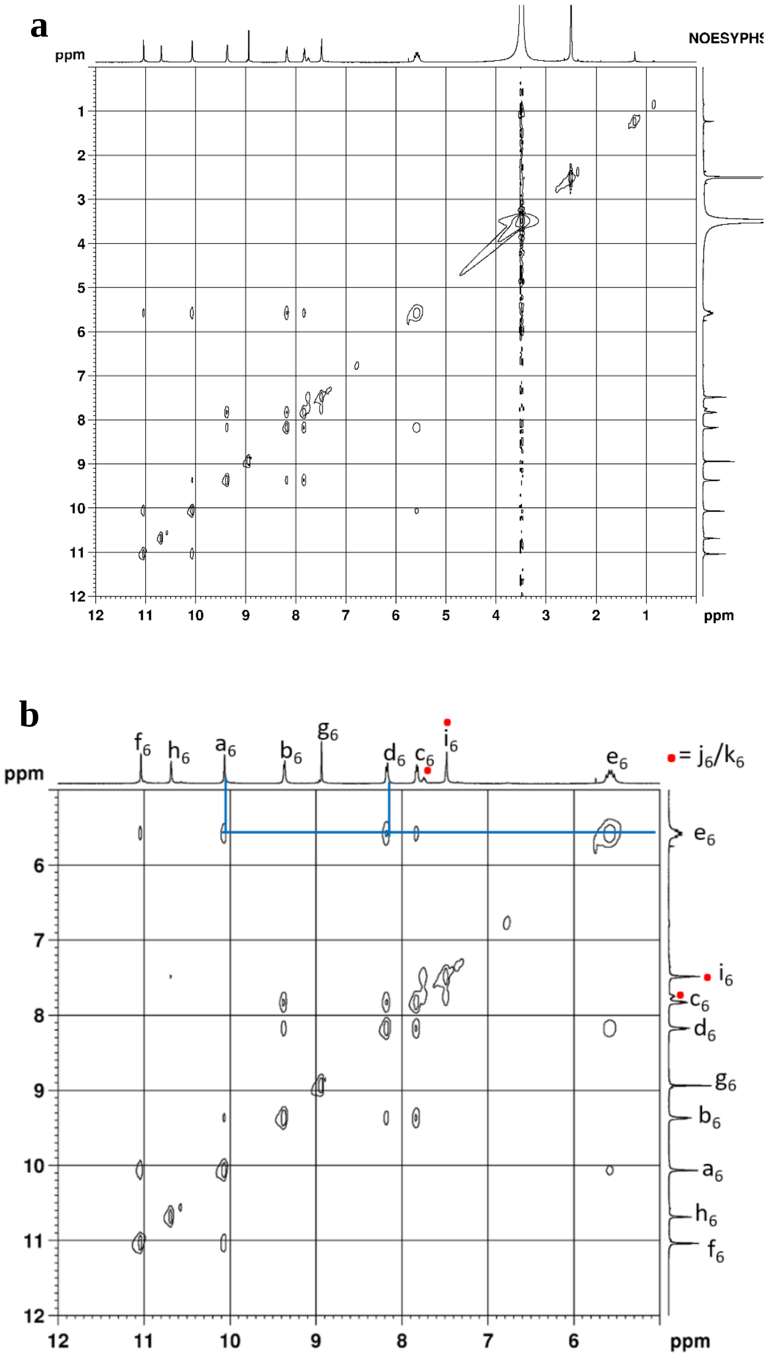

Supplementary Figure 87. NOESY spectrum (500 MHz, DMSO-d_6, 300 K) for **a** the complex **6a**, and **b** expansion of the spectrum.

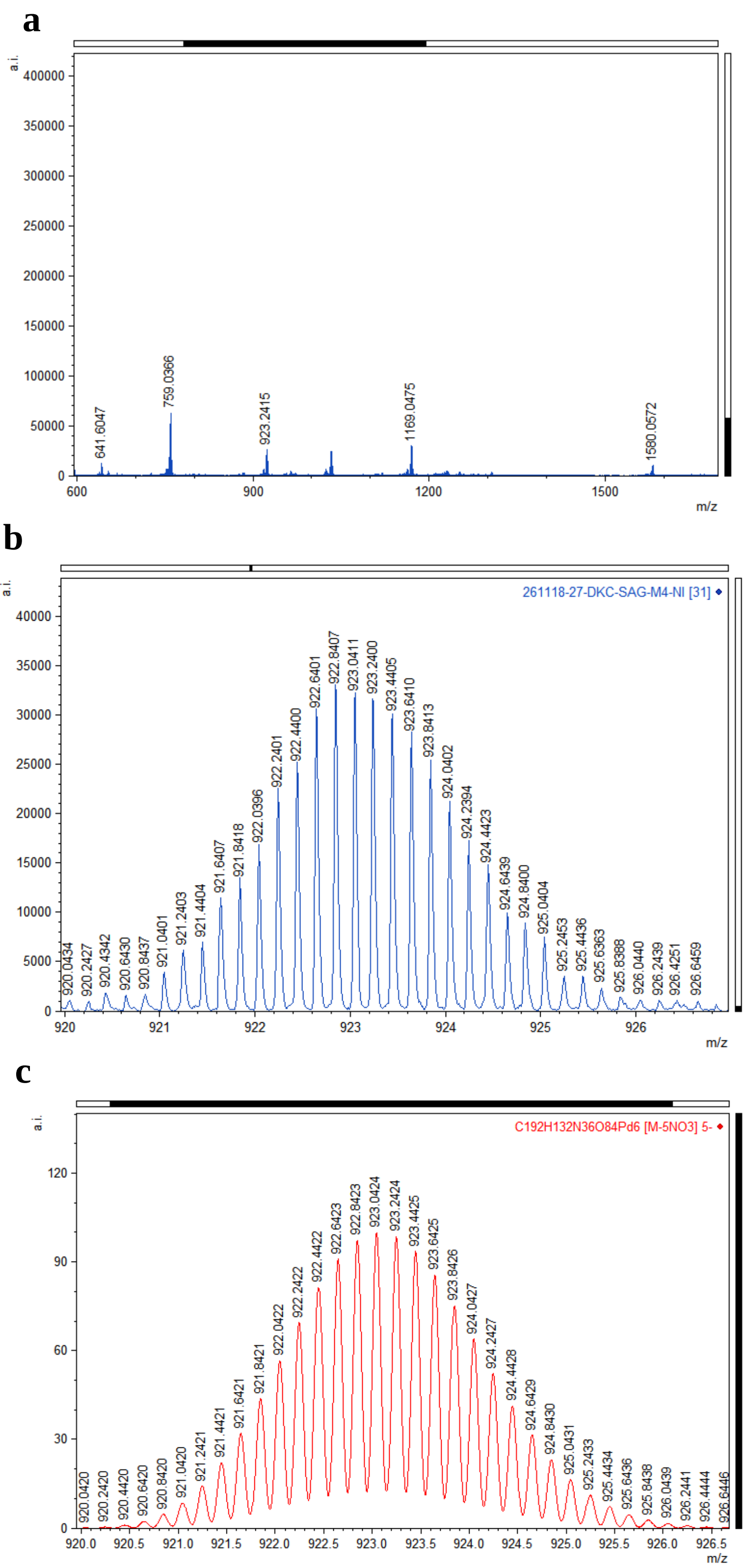

Supplementary Figure 88. ESI mass spectrum for $[(NO_3)_3 \subset Pd_6(\mathbf{L6})_6](NO_3)_9$, **6a** showing **a** full spectrum, **b** experimental isotopic pattern for $[\mathbf{6a}–5NO_3]^{5+}$, **c** theoretical isotopic pattern for $[\mathbf{6a}–5NO_3]^{5+}$.

Characterization of the complexes $[(X)_3\subset Pd_6(L6)_6](NO_3)_9$, 6b-6d

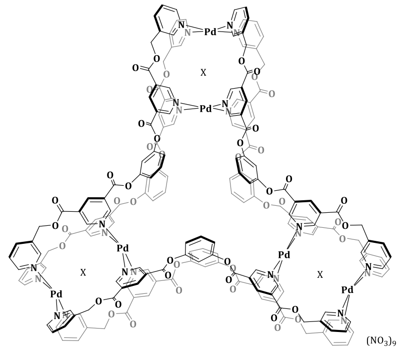

Portionwise addition of TBAF to $[(NO_3)_3\subset Pd_6(L6)_6](NO_3)_9$, 6a for the synthesis of the complex $[(F)_3\subset Pd_6(L6)_6](NO_3)_9$, 6b

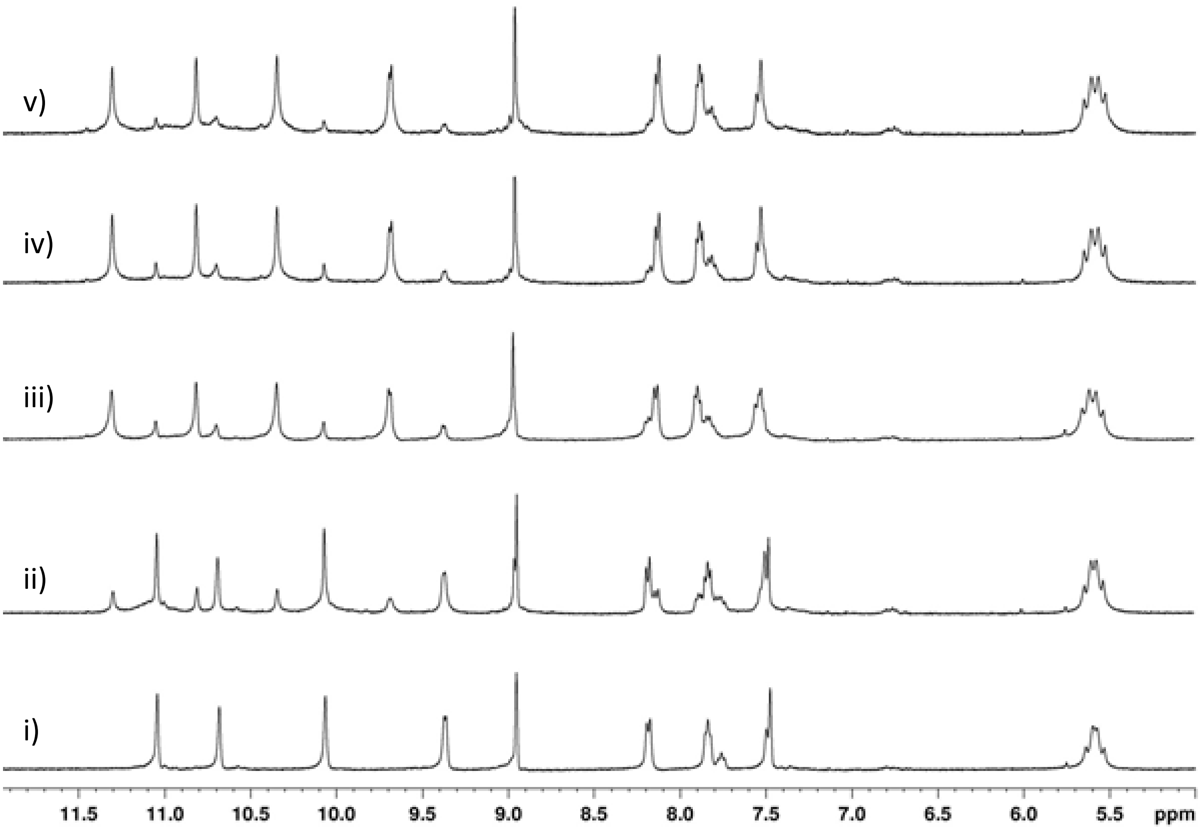

Supplementary Figure 89. Partial ^{1}H NMR spectra (400 MHz, DMSO-d_6, 300 K) for (i) $[(NO_3)_3\subset Pd_6($**L6**$)_6](NO_3)_9$, **6a**; portionwise addition of TBAF to **6a** (ii) 0.5 equiv. (iii) 1 equiv. (iv) 1.5 equiv. (v) 2 equiv.; Exclusive formation of (v) $[(F)_3\subset Pd_6($**L6**$)_6](NO_3)_9$, **6b** upon addition of 3 equiv.

Portionwise addition of TBACl to [$(NO_3)_3$⊂Pd_6(L6)$_6$]$(NO_3)_9$, 6a for the synthesis of the complex [$(Cl)_3$⊂Pd_6(L6)$_6$]$(NO_3)_9$, 6c

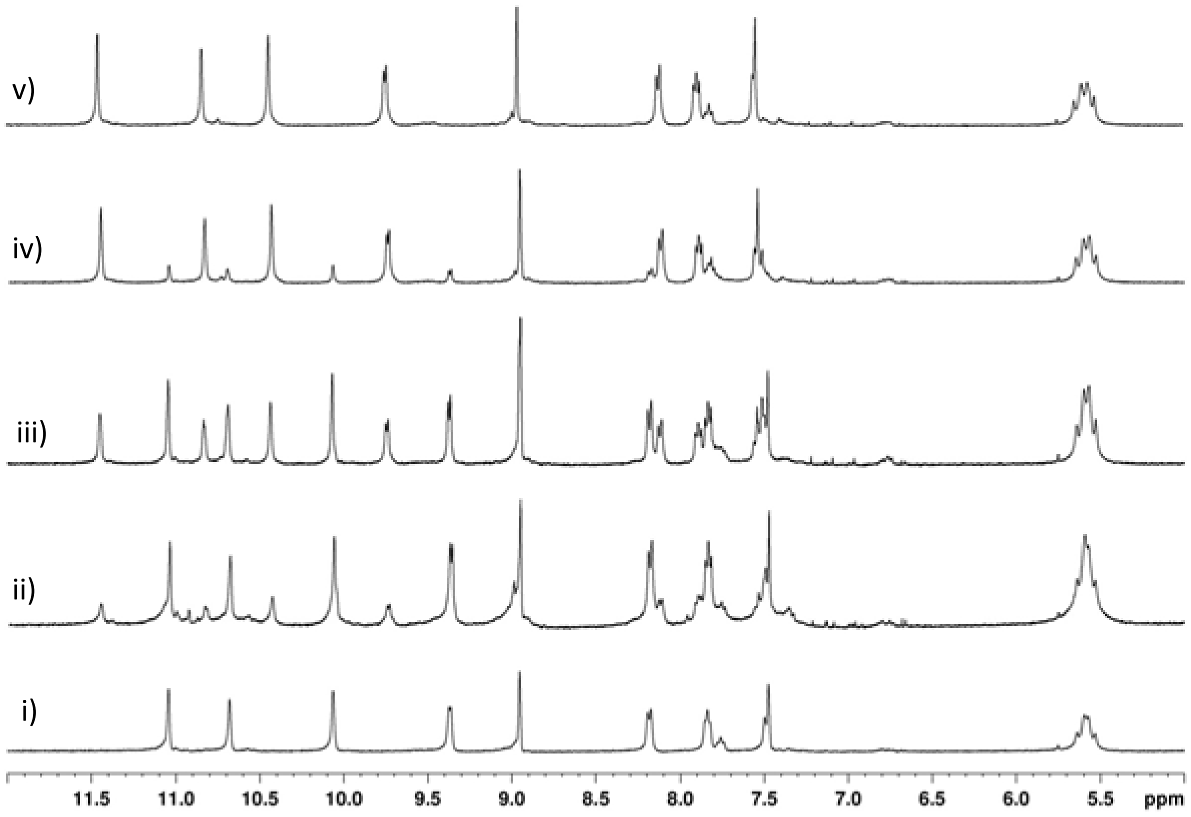

Supplementary Figure 90. Partial ^{1}H NMR spectra (400 MHz, DMSO-d_6, 300 K) for (i) [$(NO_3)_3$⊂Pd_6(**L6**)$_6$]$(NO_3)_9$, **6a**; portionwise addition of TBACl to **6a** (ii) 0.5 equiv. (iii) 1 equiv. (iv) 1.5 equiv. (v) 2 equiv.; Exclusive formation of (v) [$(Cl)_3$⊂Pd_6(**L6**)$_6$]$(NO_3)_9$, **6c** upon addition of 3 equiv.

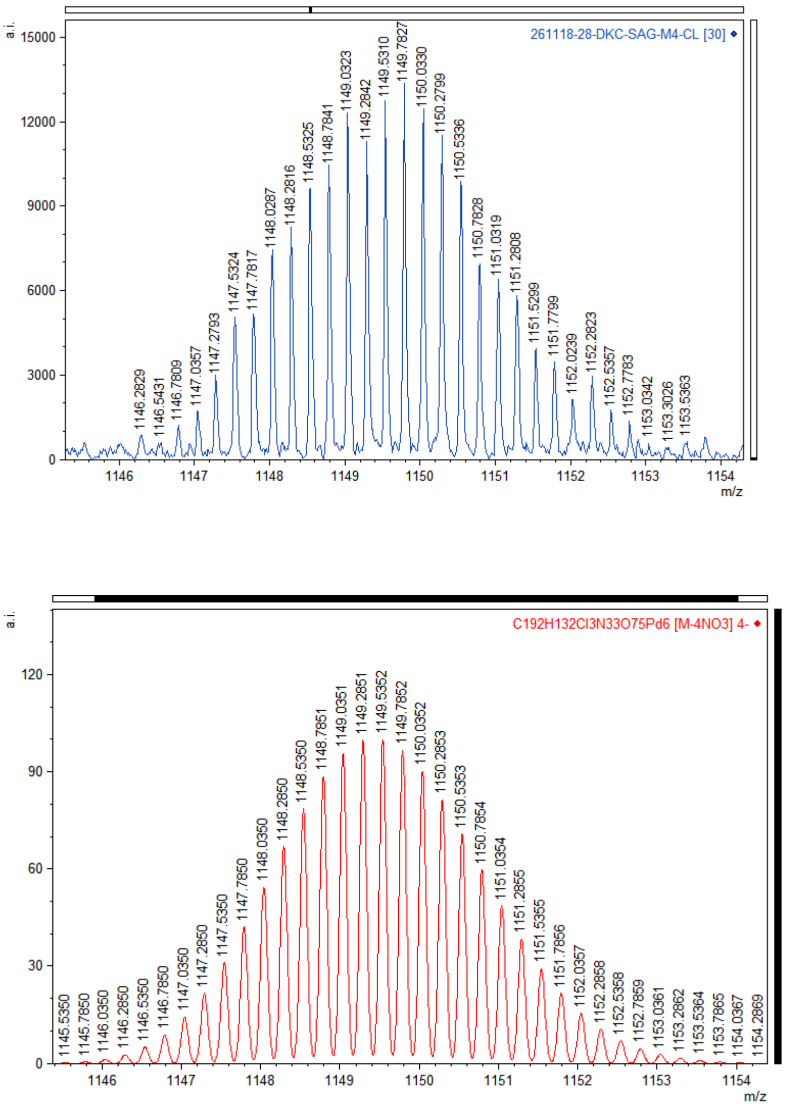

Supplementary Figure 91. ESI mass spectrum for $[(Cl)_3 \subset Pd_6(\mathbf{L6})_6](NO_3)_9$, **6c** showing **a** experimental isotopic pattern for $[\mathbf{6c}–4NO_3]^{4+}$, and **b** theoretical isotopic pattern for $[\mathbf{6c}–4NO_3]^{4+}$.

Portionwise addition of TBABr to $[(NO_3)_3\subset Pd_6(L6)_6](NO_3)_9$, 6a for the synthesis of the complex $[(Br)_3\subset Pd_6(L6)_6](NO_3)_9$, 6d

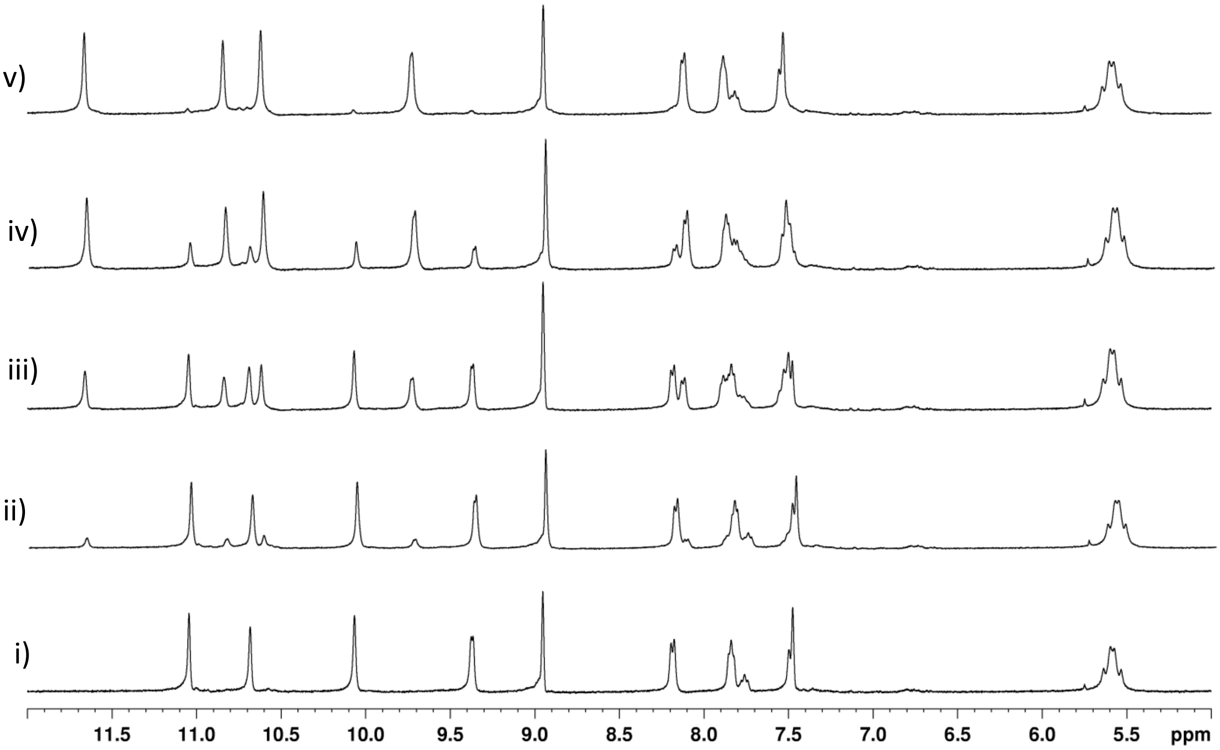

Supplementary Figure 92. Partial ^{1}H NMR spectra (400 MHz, DMSO-d_6, 300 K) for (i) $[(NO_3)_3\subset Pd_6(\mathbf{L6})_6](NO_3)_9$, **6a**; portionwise addition of TBABr to **6a** (ii) 0.5 equiv. (iii) 1 equiv. (iv) 1.5 equiv. (v) 2 equiv.; Exclusive formation of (v) $[(Br)_3\subset Pd_6(\mathbf{L6})_6](NO_3)_9$, **6d** upon addition of 3 equiv.

Stacking diagram showing ^{1}H NMR spectra of 6a-6d

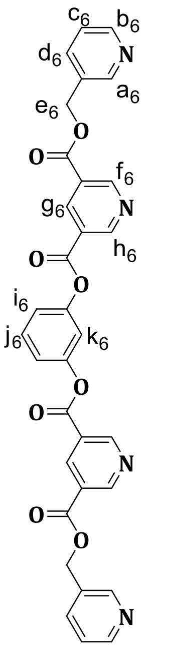

L6

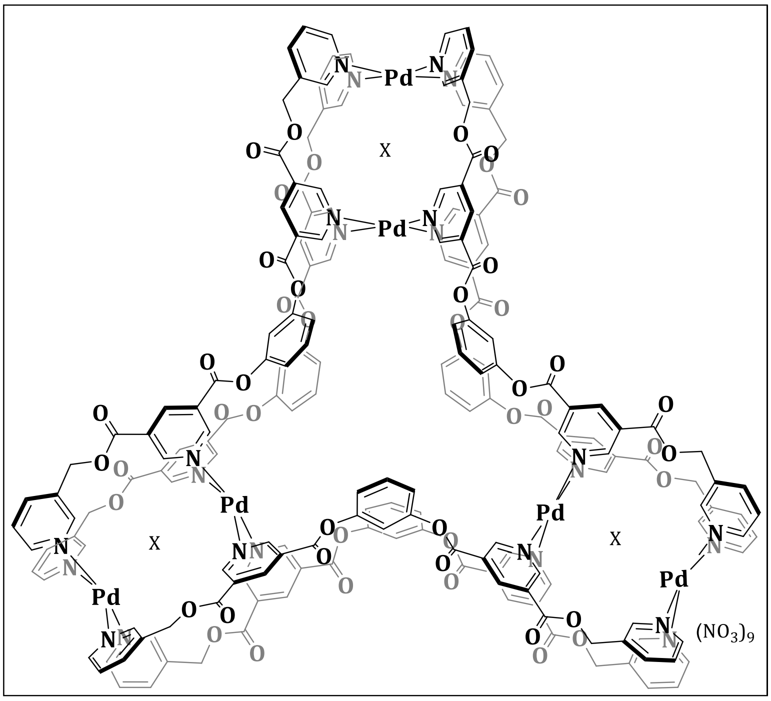

6a-6d

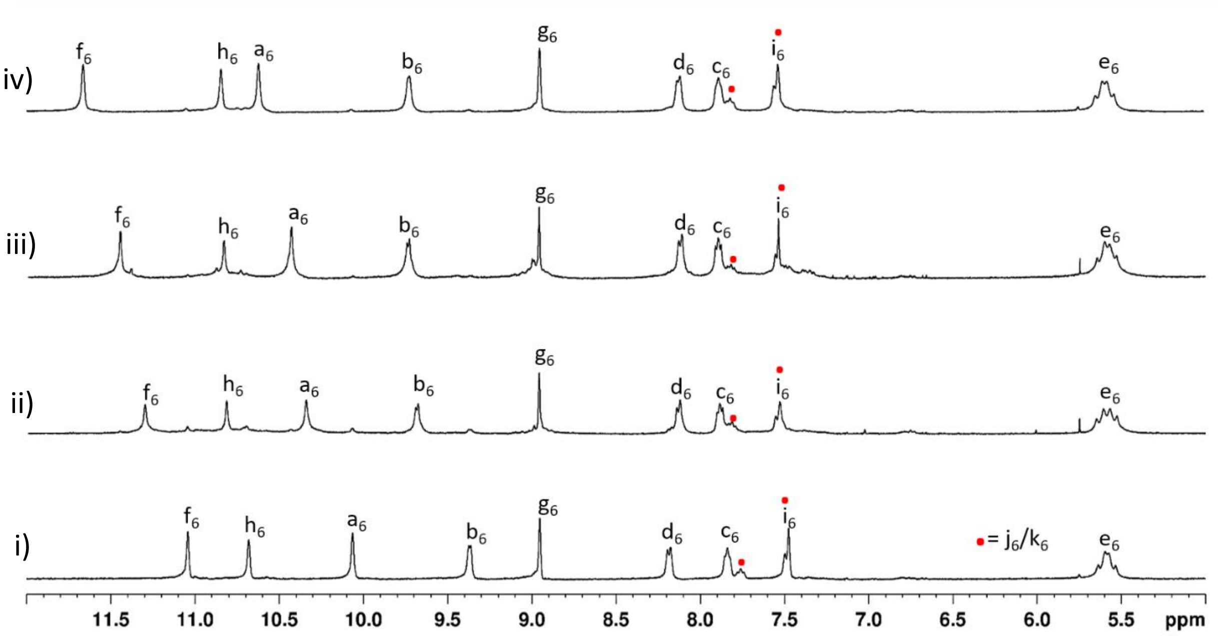

Supplementary Figure 93. 400 MHz partial ^{1}H NMR spectra in DMSO-d_6 for (i) $[(NO_3)_3\subset Pd_6($**L6**$)_6](NO_3)_9$, **6a**; (ii) $[(F)_3\subset Pd_6($**L6**$)_6](NO_3)_9$, **6b**; (iii) $[(Cl)_3\subset Pd_6($**L6**$)_6](NO_3)_9$, **6c** and (iii) $[(Br)_3\subset Pd_6($**L6**$)_6](NO_3)_9$, **6d**.

Supplementary Table 4. ^{1}H NMR chemical shift of selected protons in ppm for various complexes **6a-6d** (DMSO-d_6) for comparison with **L6**.

Proton	**L6**	$[(NO_3)_3\subset Pd_6(\mathbf{L6})_6](NO_3)_9$, **6a**	$[(F)_3\subset Pd_6(\mathbf{L6})_6](NO_3)_9$, **6b**	$[(Cl)_3\subset Pd_6(\mathbf{L6})_6](NO_3)_9$, **6c**	$[(Br)_3\subset Pd_6(\mathbf{L6})_6](NO_3)_9$, **6d**
H_{a6}	8.75	10.06	10.34	10.43	10.62
H_{b6}	8.58	9.37	9.70	9.75	9.72
H_{f6}	9.41 or 9.48	11.04	11.30	11.44	11.66
H_{h6}	9.41 or 9.48	10.68	10.82	10.84	10.84

Characterization of the complexes $[(X)_3 \subset Pd_6(L6)_6](BF_4)_9$, 6a'-6d'

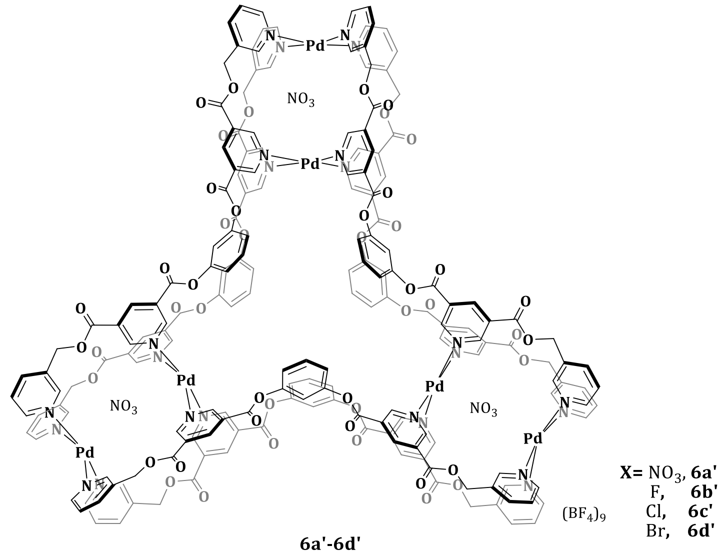

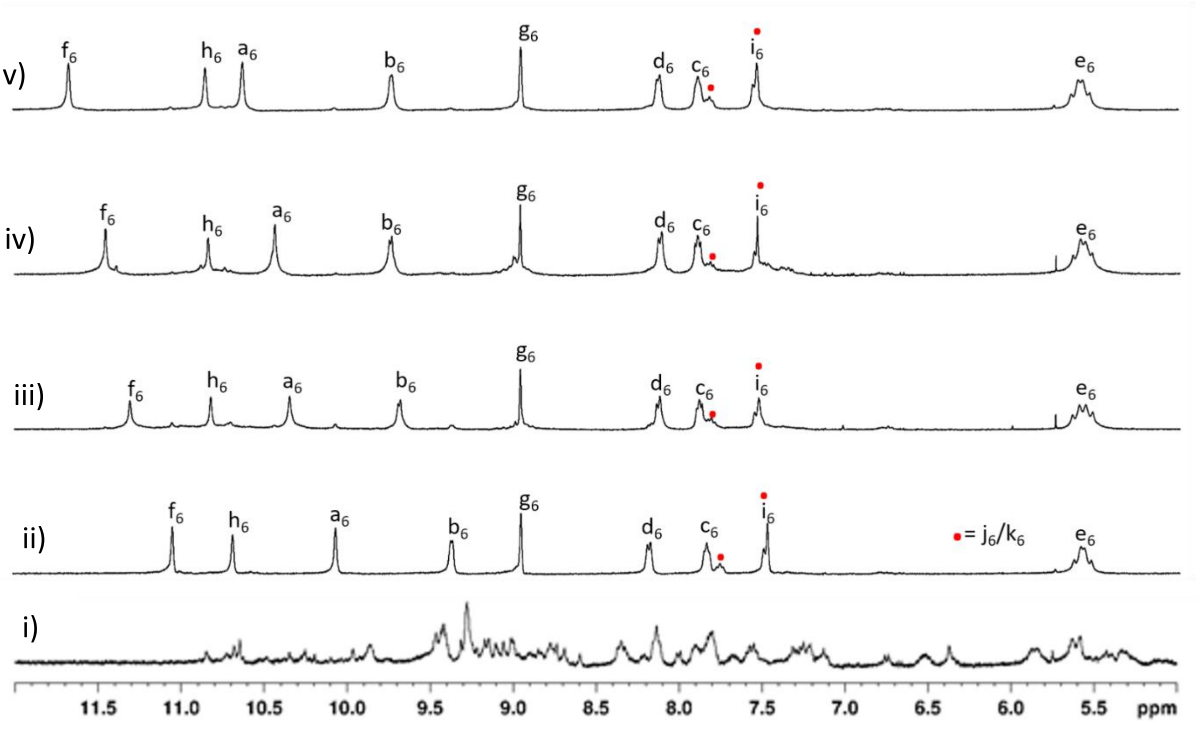

Supplementary Figure 94. 400 MHz partial ^{1}H NMR spectra in DMSO-d_6 for (i) oligomers formation upon complexation of $Pd(BF_4)_2$ with **L6** at 6:6 ratio; addition of appropriate TBAX to the oligomer resulting in the formation of (ii) $[(NO_3)_3 \subset Pd_6(\mathbf{L6})_6](BF_4)_9$, **6a'** (when X=$NO_3$); (iii) $[(F)_3 \subset Pd_6(\mathbf{L6})_6](BF_4)_9$, **6b'** (when X=F) (iv) $[(Cl)_3 \subset Pd_6(\mathbf{L6})_6](BF_4)_9$, **6c' (**when X=Cl) and (v) $[(Br)_3 \subset Pd_6(\mathbf{L6})_6](BF_4)_9$, **6d'** (when X=Br).

Characterisation of the complexes $[NO_3\subset Pd_3(L5)_4](NO_3)_5$, 4e and $[(NO_3)_2\subset Pd_6(L5)_8](NO_3)_{10}$, 4f.

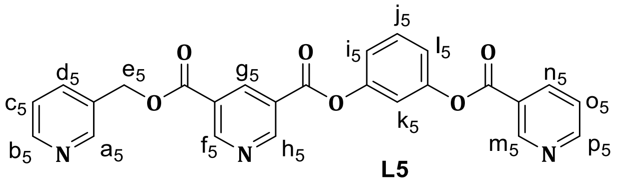

Supplementary Discussion 2. When complexation of ligand **L5** was carried out with $Pd(NO_3)_2$ in DMSO-d_6, and the ^{1}H NMR spectrum obtained was compared with the free ligand **L5**, it suggested the metal-ligand coordination as a result of which there is a downfield shift of α-pyridine protons. The spectrum of the formed complex gave more than one set of peaks for certain protons i.e., H_a, H_f, H_m and H_h that are clearly distinguishable indicating more than one complex is present. The chemical structure of these complexes are shown below in supporting figure **95**. Formation of complexes **4e** and **4f** of different nuclearity was further supported by concentration variation experiment, where appreciable change was observed for H_f proton highlighted in red box, supporting figure **102**. Variable temperature NMR didn't provide any information. Since with increase in concentration the height of the peak at 11.03 ppm increases, it corresponds to **4f** while the peak at 11.00 ppm corresponds to the lower nuclearity complex **4e**. The nuclearity of these two complexes is further supported by ESI-MS data as given in the following supporting figure **103**.

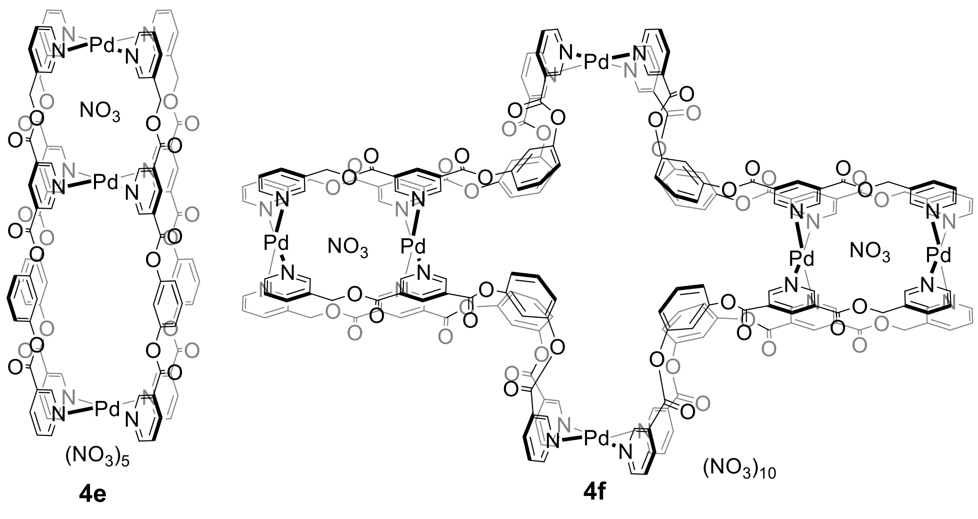

Supplementary Figure 95. Proposed structures of complexes formed by ligand **L5** upon complexation with palladium(II).

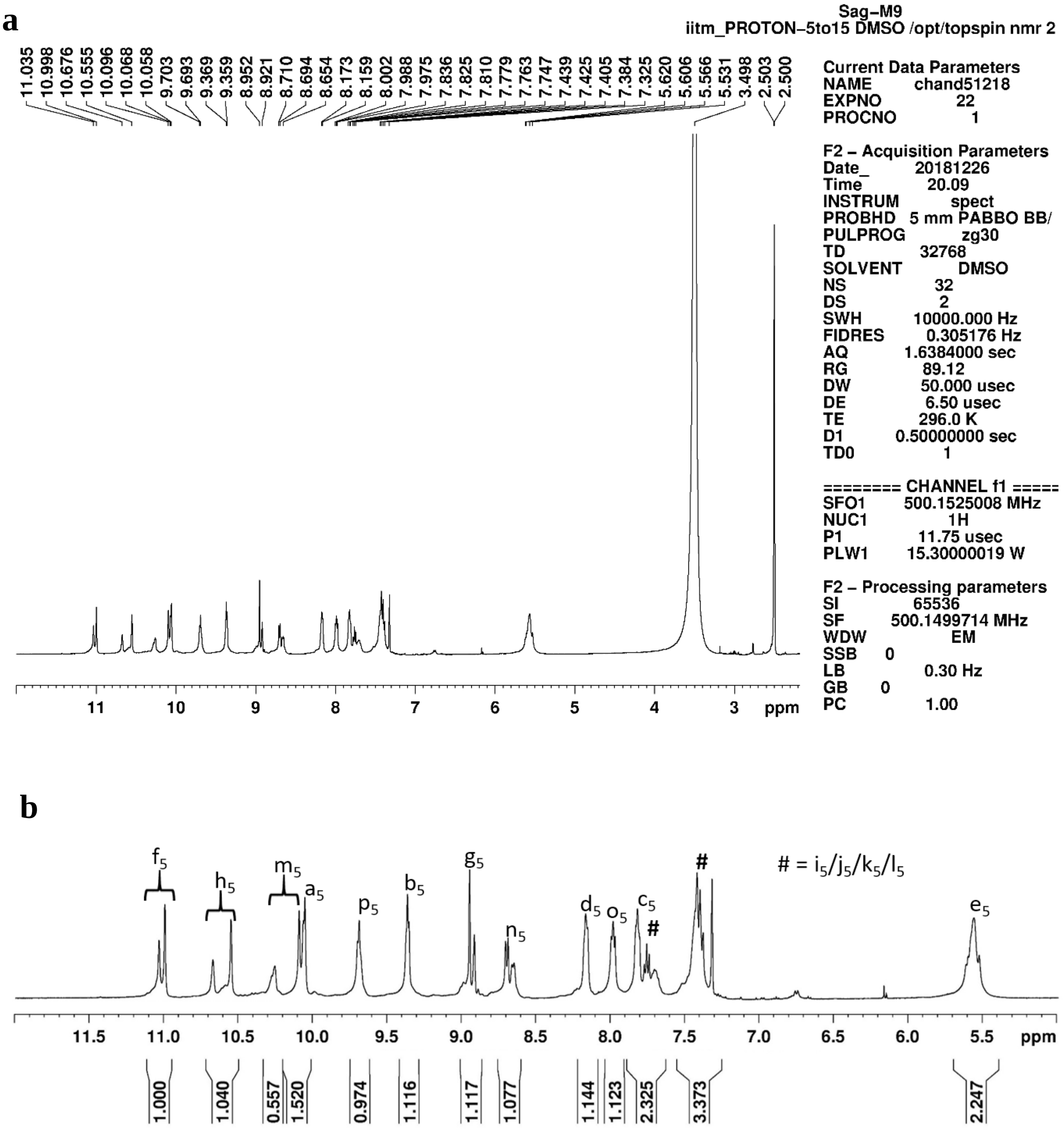

Supplementary Figure 96. ^{1}H NMR spectrum (500 MHz, DMSO-d_6, 300 K) for **a** mixture of **4e** and **4f**. (Concentration: 10 mM with respect to palladium(II) source), and **b** expansion of the spectrum.

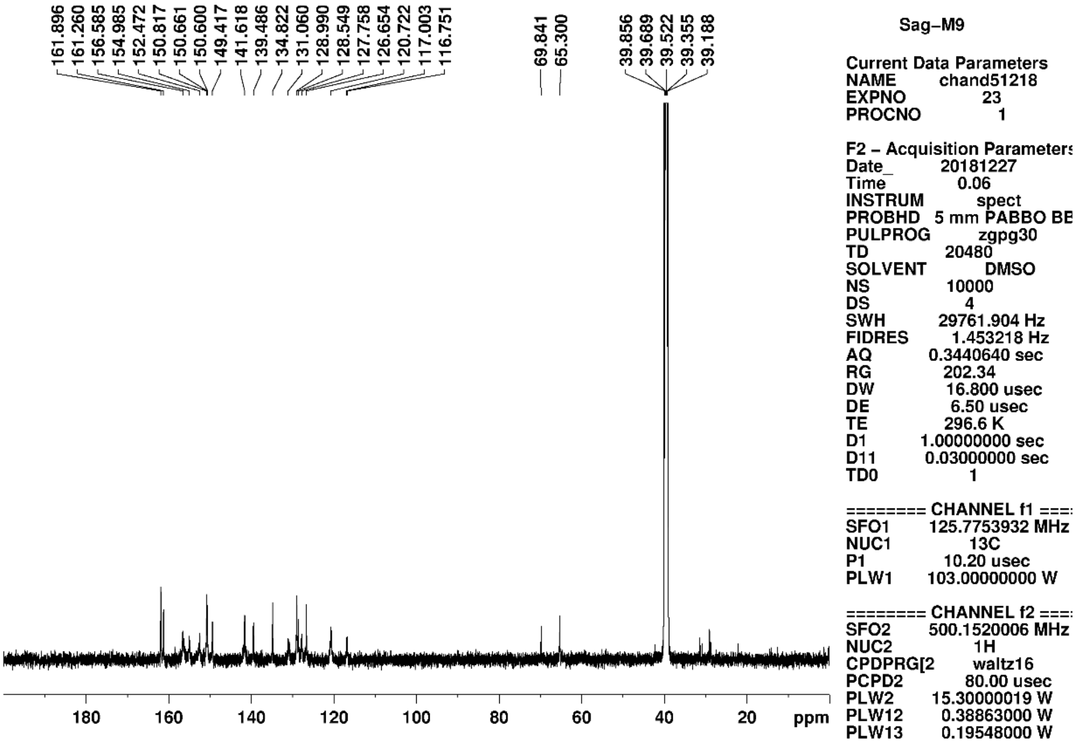

Supplementary Figure 97. ^{13}C NMR spectrum (125 MHz, DMSO-d_6, 300 K) for a mixture of **4e** and **4f**, all the peaks are not visible because of poor solubility and some of the peaks are overlapped.

a

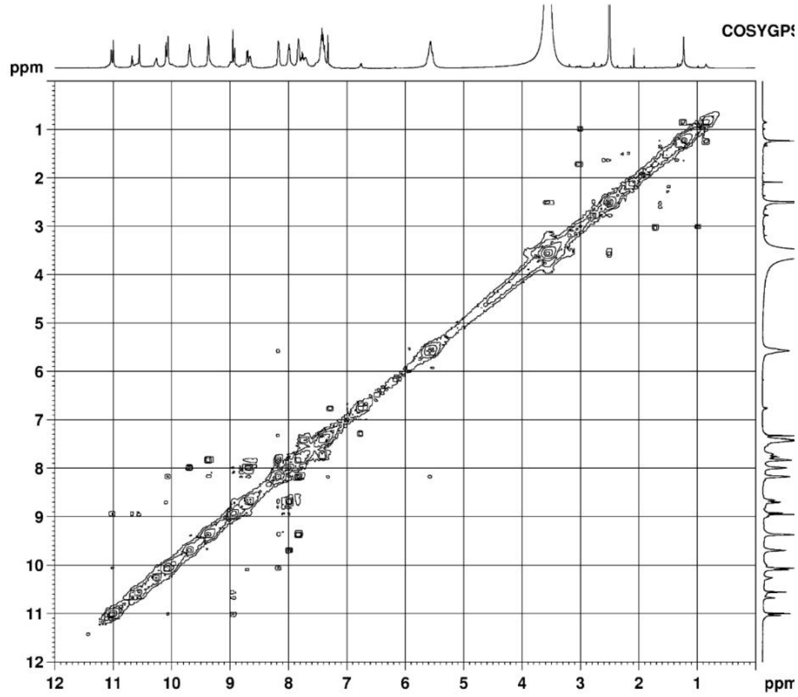

b

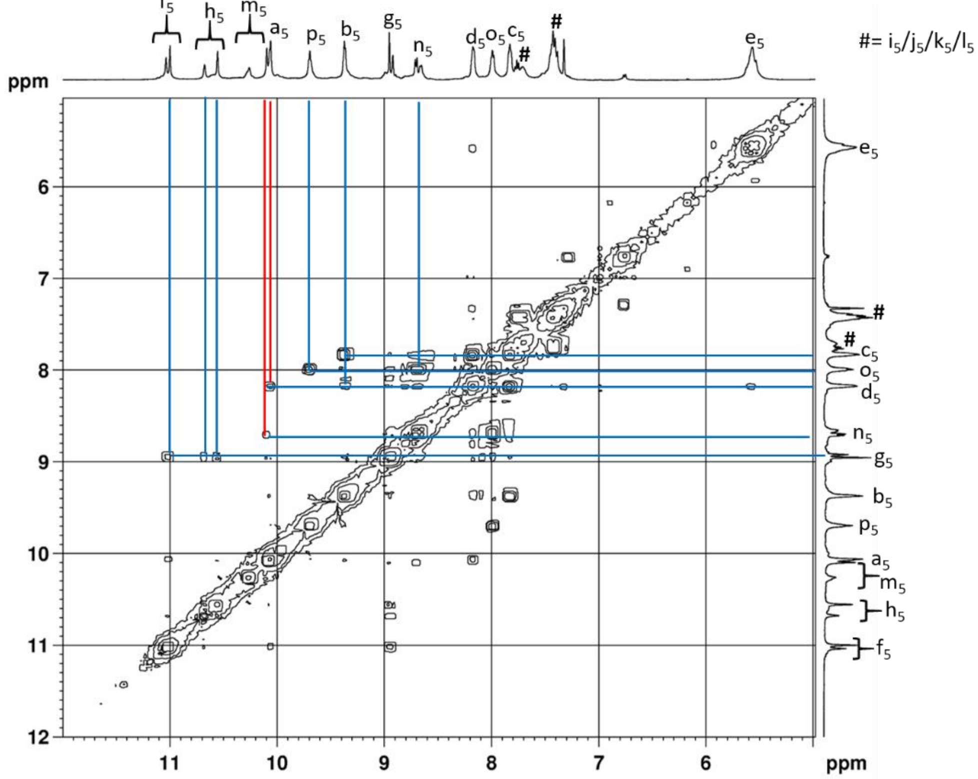

Supplementary Figure 98. H-H COSY spectrum (500 MHz, DMSO-d_6, 300 K) for **a** mixture of **4e** and **4f**, and **b** expansion of the spectrum.

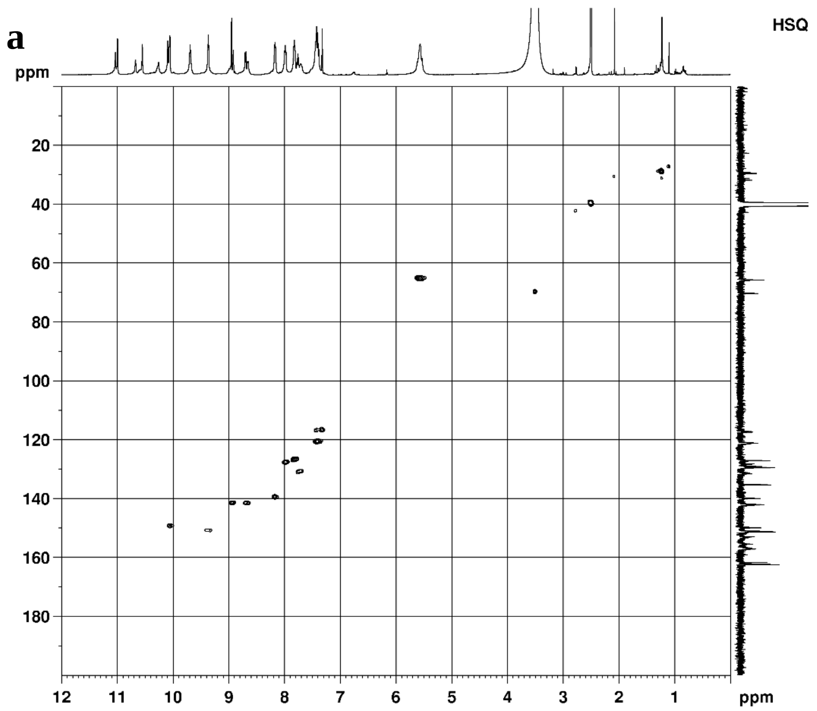

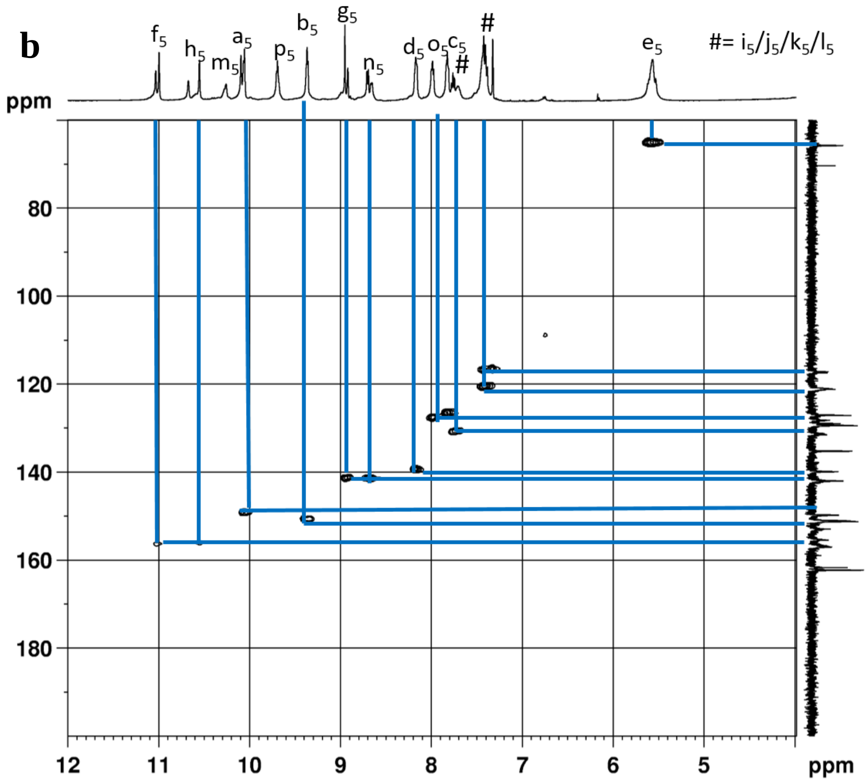

Supplementary Figure 99. C-H COSY spectrum (500 MHz, DMSO-d_6, 300 K) for **a** mixture of **4e** and **4f**, and **b** expansion of the spectrum.

a

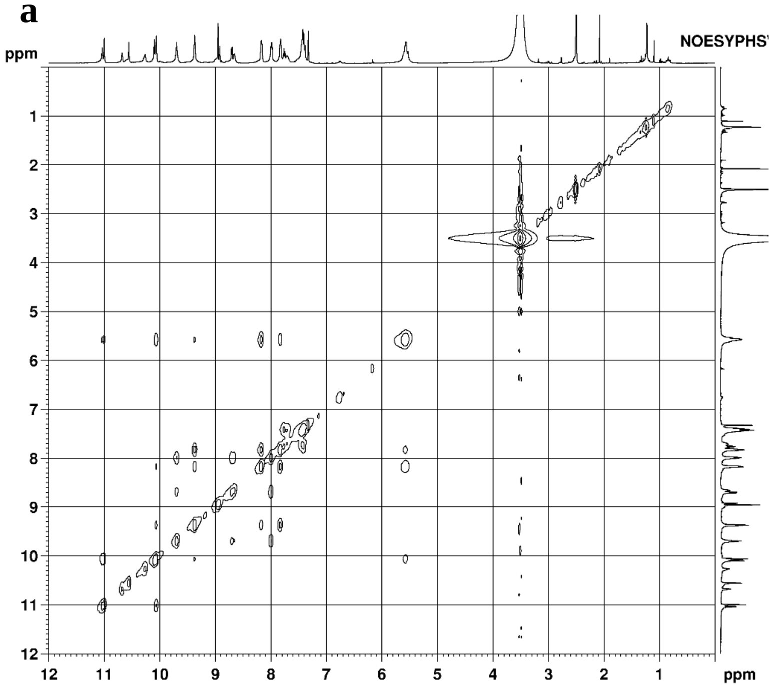

b

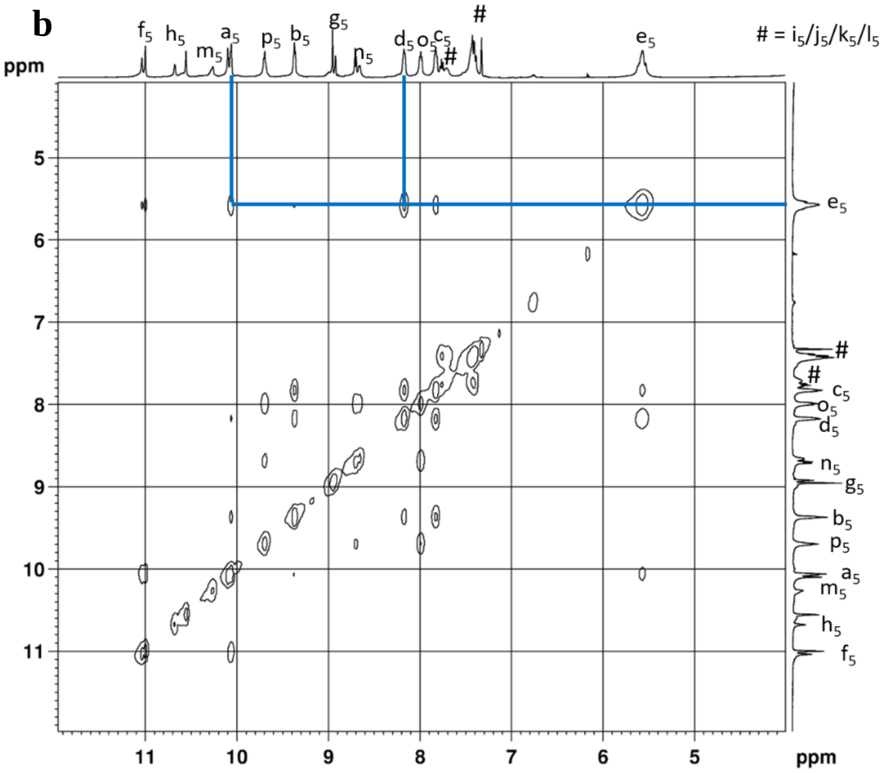

Supplementary Figure 100. NOESY spectrum (500 MHz, DMSO-d_6, 300 K) for **a** mixture of **4e** and **4f**, and **b** expansion of the spectrum.

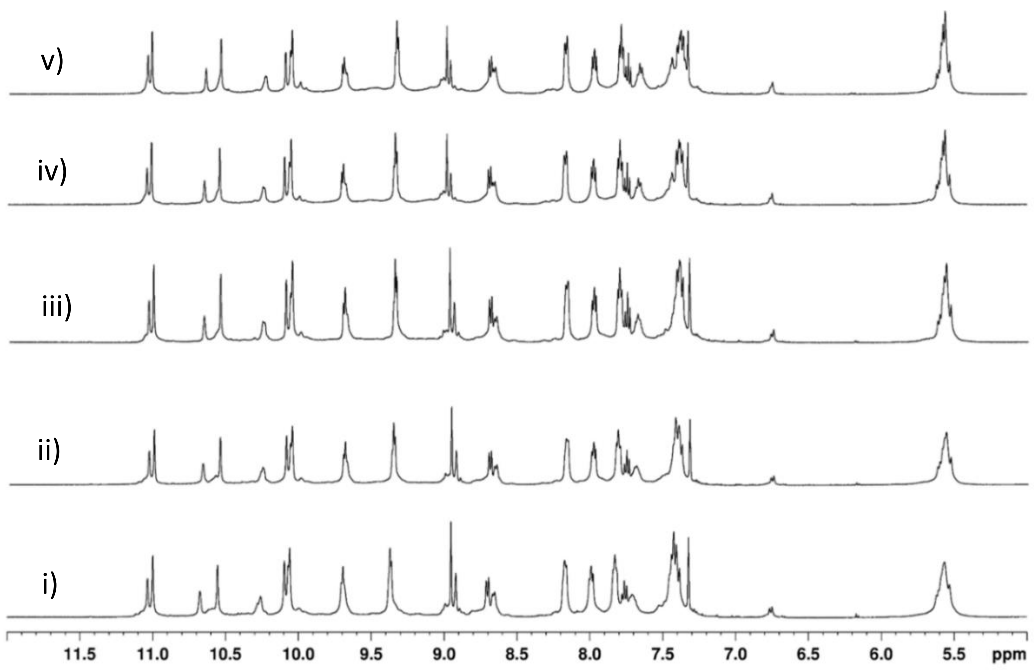

Supplementary Figure 101. 500 MHz partial ^{1}H NMR spectra for a mixture of **4e** and **4f** recorded at variable temperature i) 30 °C ii) 50 iii) 70 °C iv) 90 °C and v) 100 °C.

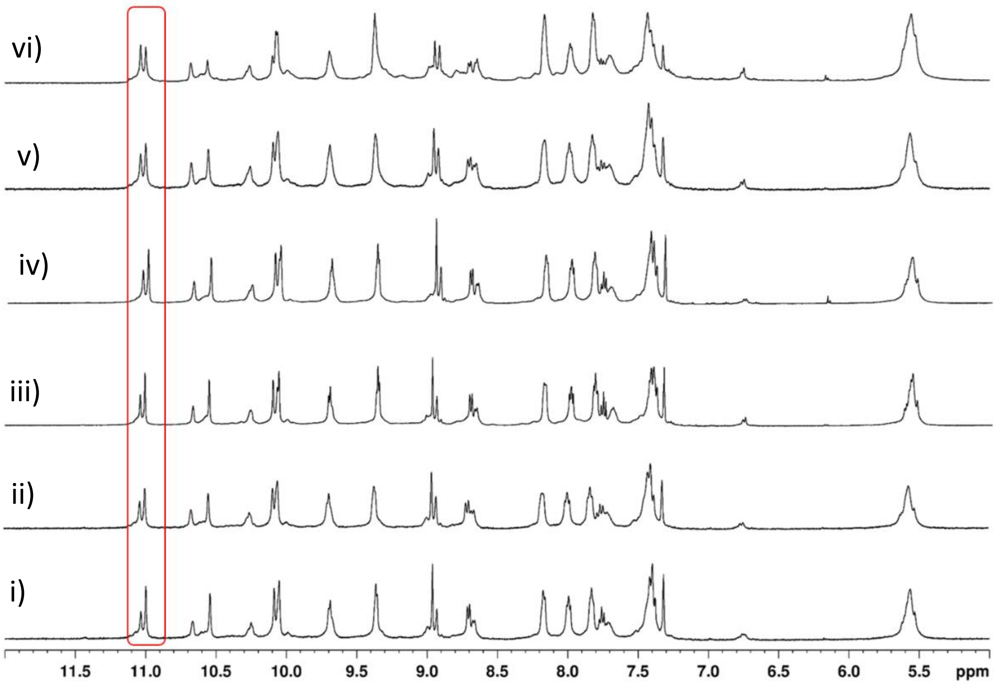

Supplementary Figure 102. Partial ^{1}H NMR spectra (400 MHz, DMSO-d_6, 300 K) for a mixture of **4e** and **4f** @ (i) 5 mM; (ii) 10 mM ; (iii) 15 mM ; (iv) 20 mM; (v) 25 mM and (vi) 30 mM concentration with respect to $Pd(NO_3)_2$. ***(**Concentration of **4e** and **4f**: 10 mM with respect to palladium(II) source).

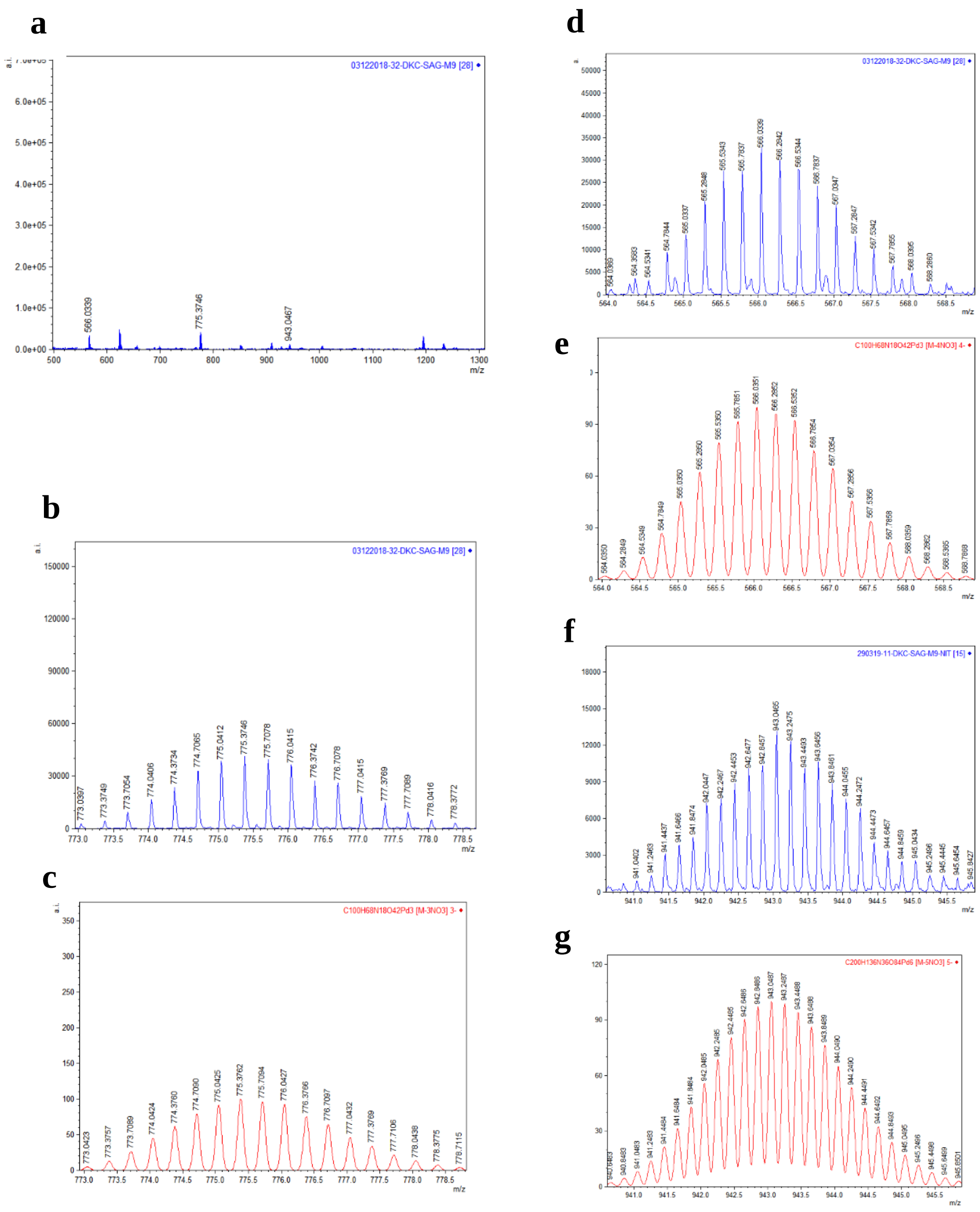

Supplementary Figure 103. ESI mass spectrum for $[NO_3{\subset}Pd_3(\mathbf{L5})_4](NO_3)_5$, **4e** and $[(NO_3)_2{\subset}Pd_6(\mathbf{L5})_8](NO_3)_{10}$, **4f**. showing **a** full spectrum, **b** experimental isotopic pattern for $[\mathbf{4e}-3NO_3]^{3+}$, **c** theoretical isotopic pattern for $[\mathbf{4e}-3NO_3]^{3+}$, **d** experimental isotopic pattern for $[\mathbf{4e}-4NO_3]^{4+}$, **e** theoretical isotopic pattern for $[\mathbf{4e}-4NO_3]^{4+}$, **f** experimental isotopic pattern for $[\mathbf{4f}-5NO_3]^{5+}$, and **g** theoretical isotopic pattern for $[\mathbf{4f}-5NO_3]^{5+}$.

Supplementary Discussion 3. The ESI-MS spectrum analysis suggest the existence of trinuclear **4e (**Supporting Figure 103a,b**)** and hexanuclear species **4f (**Supporting Figure 103c**)** in the solution state. Thus based on the NMR analysis and ESI-MS spectrum we propose the complex formed by ligand **L5** to be a mixture of **4e** and **4f**.

Characterisation of the complexes $[Cl\subset Pd_3(L5)_4](NO_3)_5$, 4g and $[(Cl)_2\subset Pd_6(L5)_8](NO_3)_{10}$, 4h.

Supplementary Discussion 4. When the complex **4e** and **4f** reacted with AgCl the spectra obtained showed down field shift of selected α pyridine protons indicating the formation of **4e** and **4f** complexes with encapsulated Cl^{-} ions **(**Supporting Figure S104**).** The formation of chloride encapsulated complexes **4g** and **4h** was further supported by ESI-MS data.

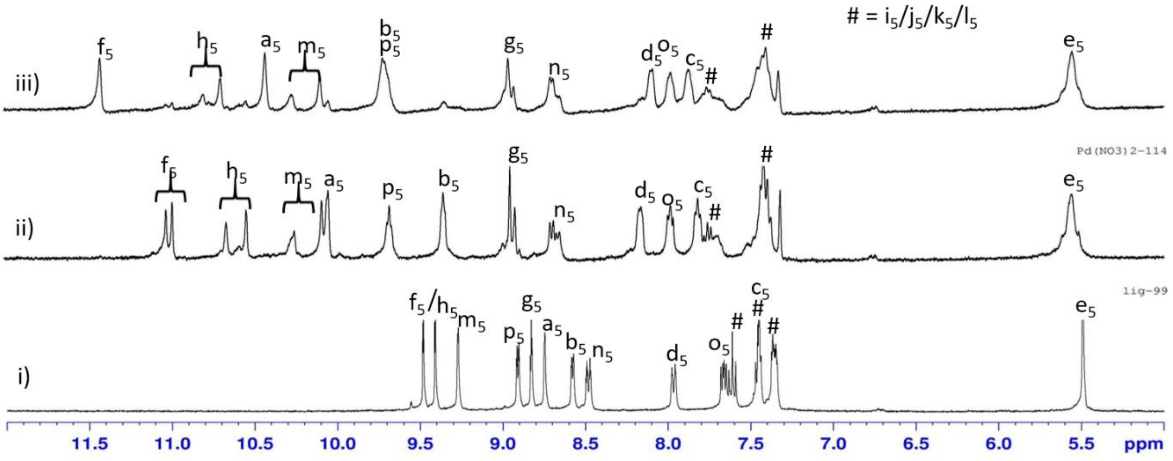

Supplementary Figure 104. Partial ^{1}H NMR spectra (400 MHz, DMSO-d_6, 300 K) for i) **L5**; ii) mixture of **4e** and **4f** and iii) addition of AgCl to **4e** and **4f** leading to the formation of **4g** and **4h**.

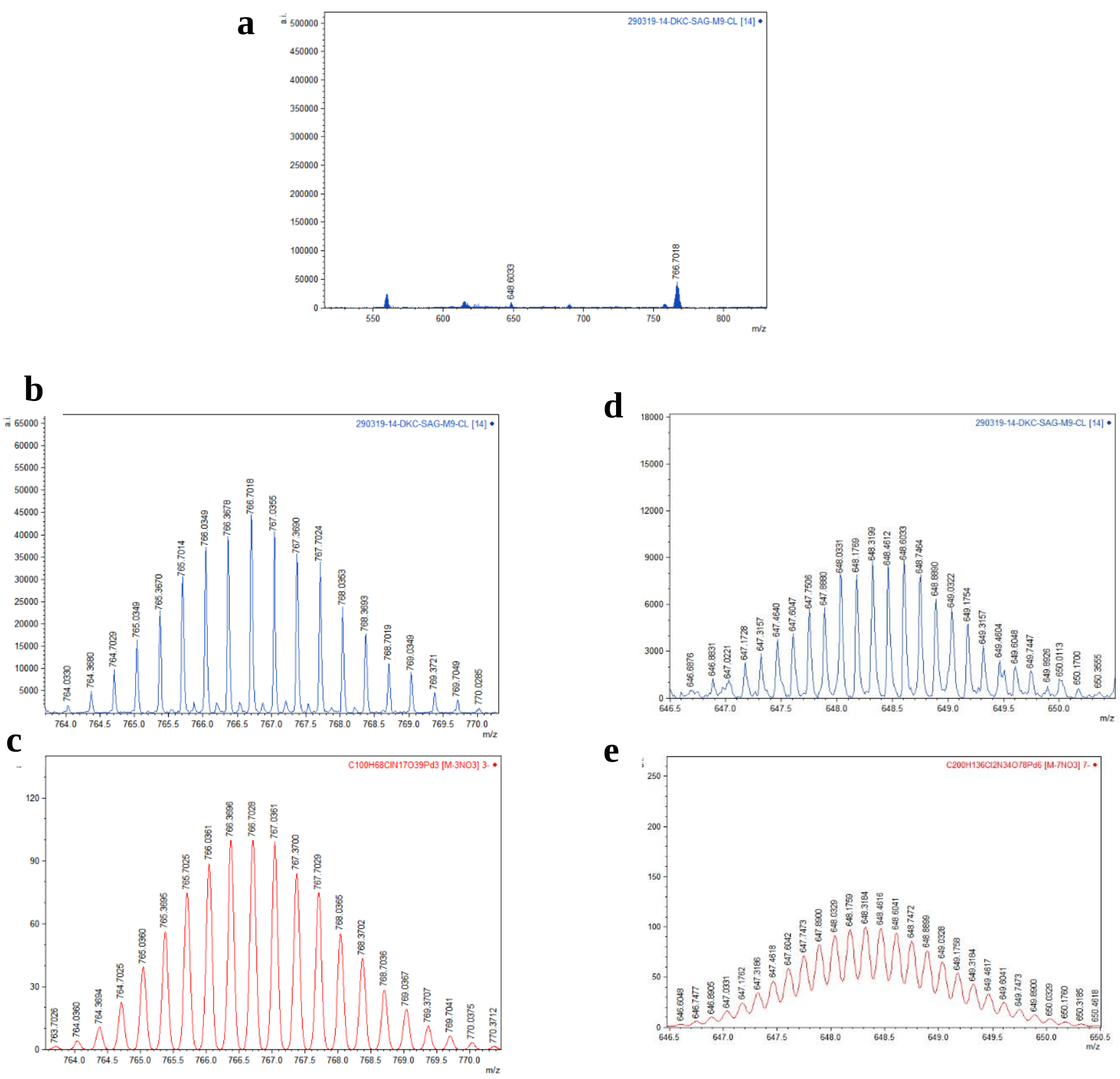

Supplementary Figure 105. ESI mass spectrum for [Cl⊂Pd_3(**L5**)$_4$]$(NO_3)_5$, **4g** and [$(Cl)_2$⊂Pd_6(**L5**)$_8$]$(NO_3)_{10}$, **4h** showing **a** full spectrum, **b** experimental isotopic pattern for [**4g** –$3NO_3$]$^{3+}$, **c** theoretical isotopic pattern for [**4g** –$3NO_3$]$^{3+}$, **d** experimental isotopic pattern for [**4h**–$7NO_3$]$^{7+}$, and **e** theoretical isotopic pattern for [**4h**–$7NO_3$]$^{7+}$.

Supplementary Discussion 5. Detailed analysis of the ESI-MS data revealed that the ligand **L5** upon complexation with palladium(II) formed mixture of products out of which the presence of trinuclear and hexanuclear complexes were supported by the ESI-MS spectrum. Similarly the mixture of complexes with encapsulated Cl^{-} formed by ligand **L5** with palladium(II) showed the presence of trinuclear and hexanuclear complexes.

Cage-fusion reactions

Cartoon representations of cationic portions of cages.

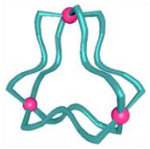

$[Pd_3(L3)_6](NO_3)_6$, **3a**

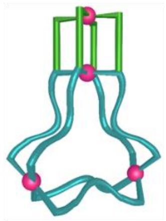

$[NO_3 \subset Pd_4(L3)_2(L5)_4](NO_3)_7$, **4a**

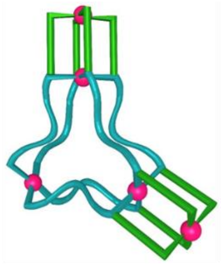

$[(NO_3)_2 \subset Pd_5(L5)_4(L6)_2](NO_3)_8$, **5a**

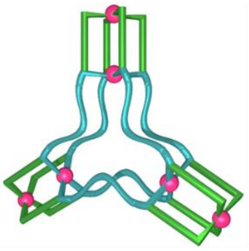

$[(NO_3)_3 \subset Pd_6(L6)_6](NO_3)_9$, **6a**

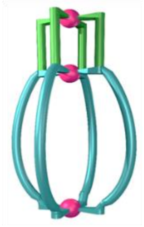

$[NO_3 \subset Pd_3(L5)_4](NO_3)_5$, **4e**

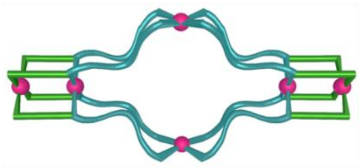

$[(NO_3)_2 \subset Pd_6(L5)_8](NO_3)_{10}$, **4f**

The complexes **4e** and **4f** coexist. However, only **4e** is used for simplicity and calculation in the equations shown below.

Fusion of cages are shown below:

- Fusion of homoleptic complexes

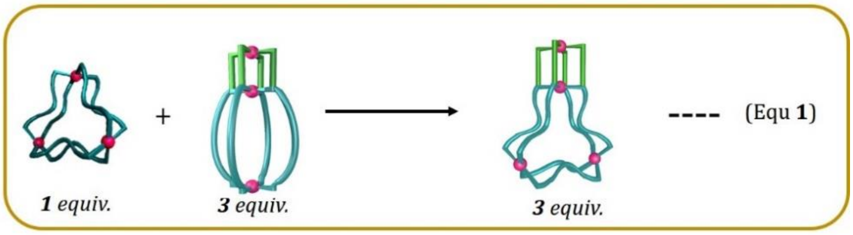

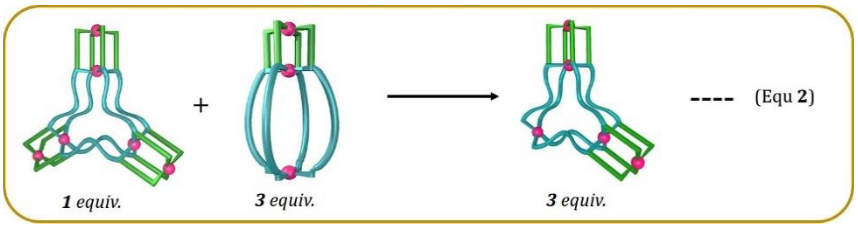

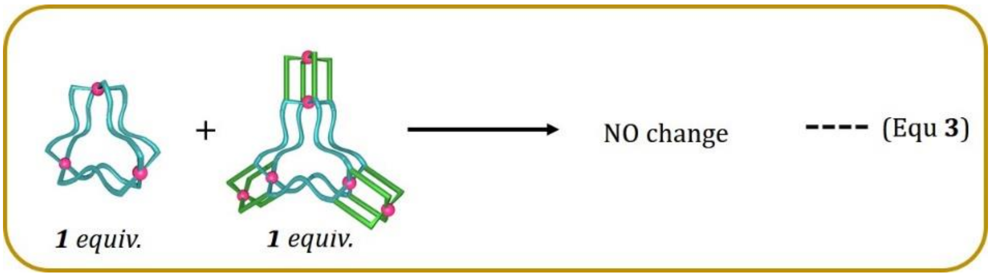

- Fusion of heteroleptic complexes

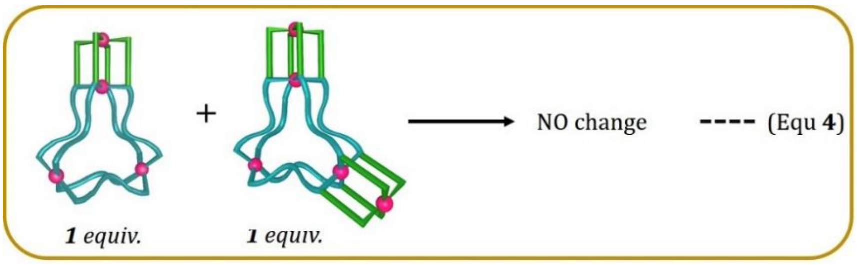

- Fusion of homoleptic and heteroleptic complexes

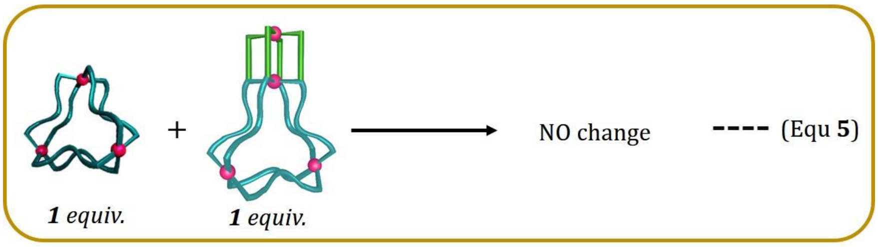

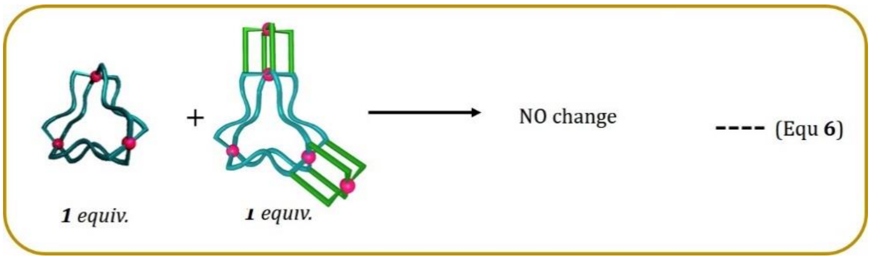

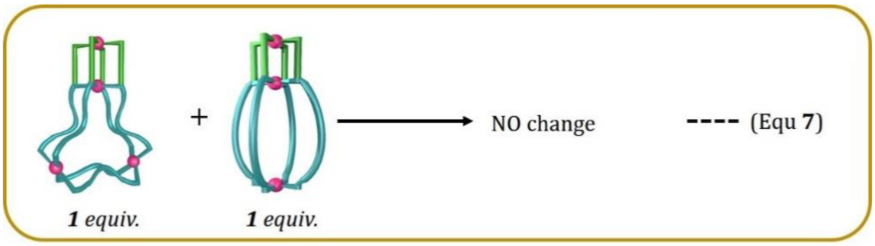

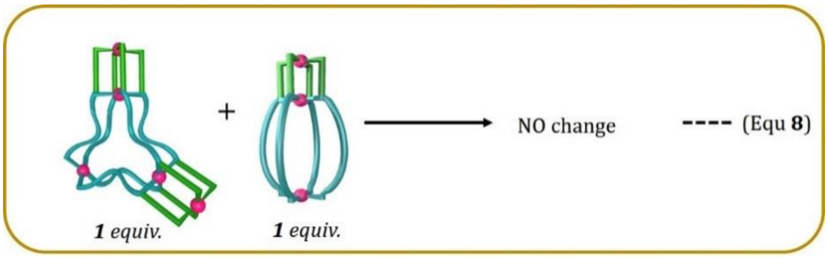

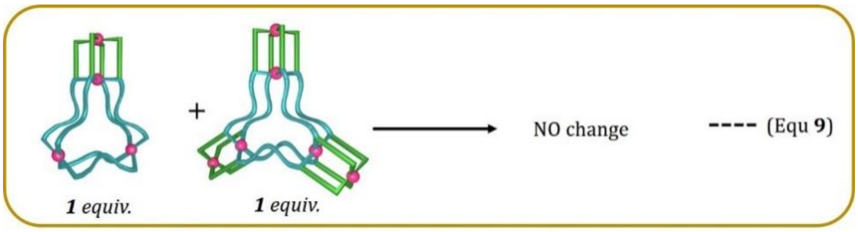

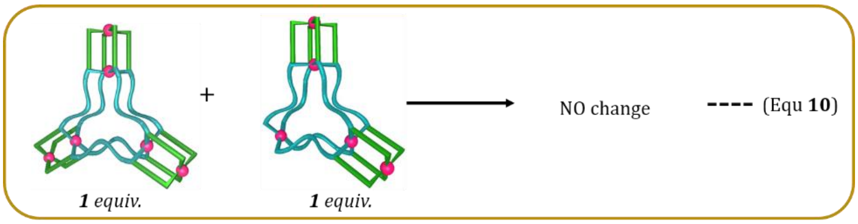

Fusion of 3a and 4e: Feasible to form 4a

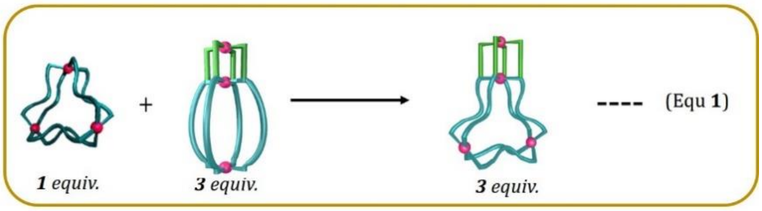

Solution 1: To the solution of ligand **L3** (0.80 mg, 0.0025 mmol, 6 equiv.) in 0.5 mL of DMSO-d_6, $Pd(NO_3)_2$ (0.28 mg, 0.0013 mmol, 3 equiv.) was added and stirred at room temperature for 10 min to obtain the trinuclear complex $[Pd_3(\mathbf{L3})_6](NO_3)_6$,**3a**.

Solution 2: To a solution of ligand **L5** (2.28 mg, 0.005 mmol, 12 equiv.) in 0.5 mL of DMSO-d_6, $Pd(NO_3)_2$ (0.87 mg, 0.0037 mmol, 9 equiv.) was added and stirred at 70 °C for 1 h to obtain the proposed complex $[NO_3\subset Pd_3(\mathbf{L5})_4](NO_3)_5$, **4e** and $[(NO_3)_2\subset Pd_6(\mathbf{L5})_8](NO_3)_{10}$, **4f**.

The heteroleptic complex $[NO_3\subset Pd_4(\mathbf{L3})_2(\mathbf{L5})_4](NO_3)_7$, **4a** was obtained by stirring the mixture of the two solutions as mentioned above containing the complexes **3a**, **4e** and **4f** at room temperature for 4h. Whereas, it took 20 mins for the transformation to occur at 70 °C.

Formation of **4a** at room temperature

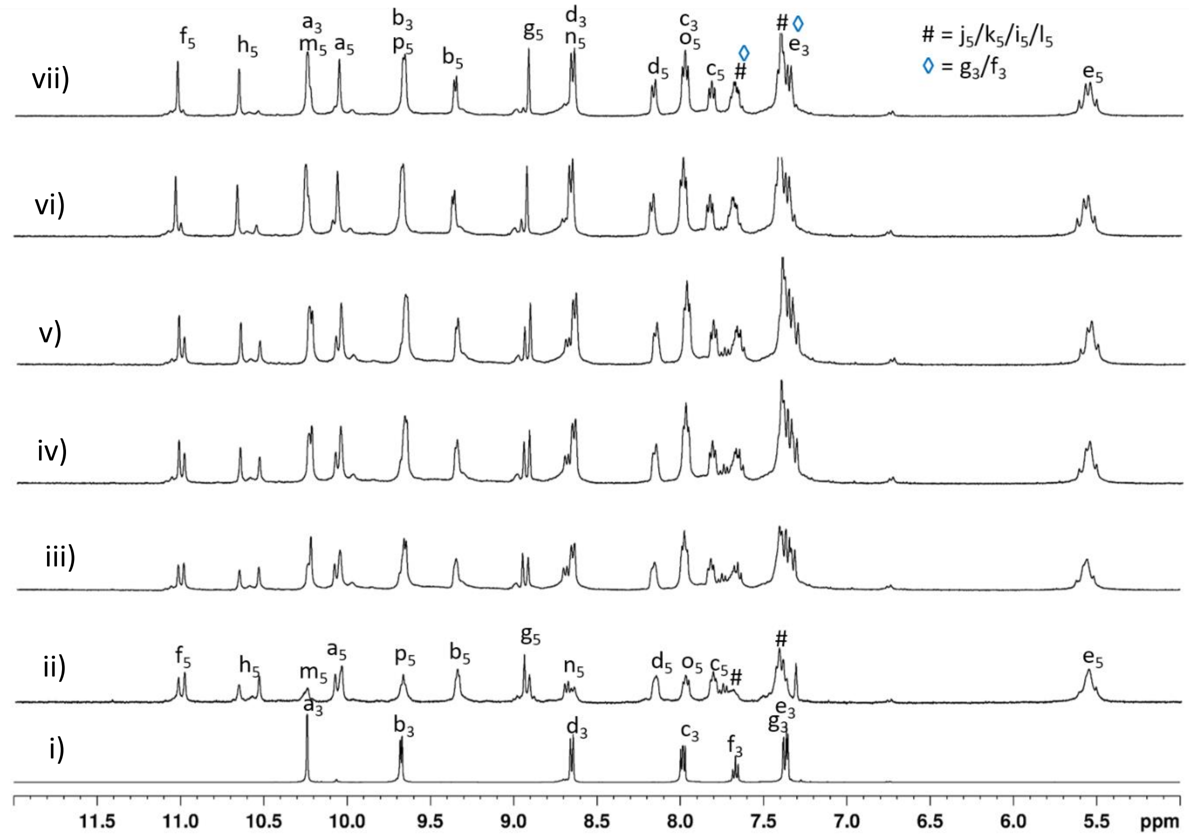

Supplementary Figure 106. Partial ^{1}H NMR spectra (400 MHz, DMSO-*d*$_6$, 300 K) of: (i) trinuclear complex $[Pd_3($**L3**$)_6](NO_3)_6$, **3**a (ii) proposed complex $[NO_3{\subset}Pd_3($**L5**$)_4](NO_3)_5$, **4e**; (iii)-(vii) After mixing trinuclear complex $[Pd_3($**L3**$)_6](NO_3)_6$, **3**a and proposed complex $[NO_3{\subset}Pd_3($**L5**$)_4](NO_3)_5$, **4e** monitoring the reaction (iii) immediately; after (iv) 1 h., (v) 2 h.,(vi) 3 h., (vii) 4 h the complex $[NO_3{\subset}Pd_4($**L3**$)_2($**L5**$)_4](NO_3)_7$, **4a**.

Formation of **4a** at 70 °C

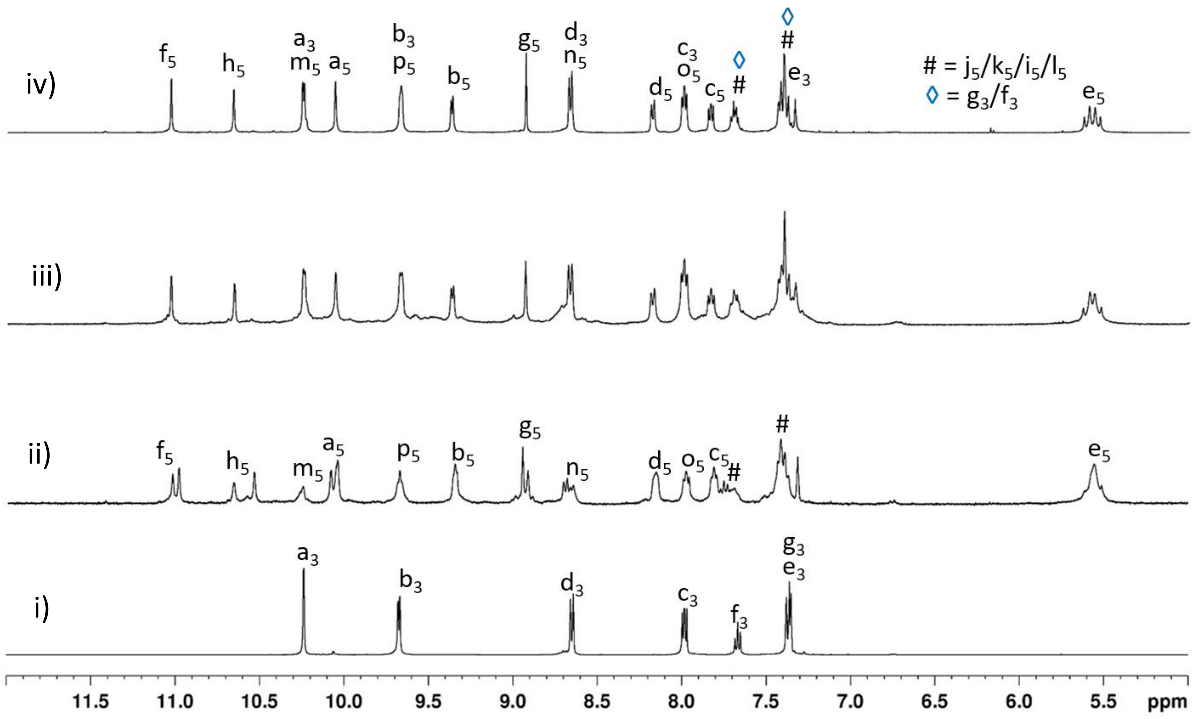

Supplementary Figure 107. Partial ^{1}H NMR spectra (400 MHz, DMSO-d_6, 300 K) of: (i) trinuclear complex $[Pd_3(\mathbf{L3})_6](NO_3)_6$, **3**a proposed complex $[NO_3\subset Pd_3(\mathbf{L5})_4](NO_3)_5$, **4e**; (iii)-(iv) After mixing trinuclear complex $[Pd_3(\mathbf{L3})_6](NO_3)_6$, **3**a and proposed complex $[NO_3\subset Pd_3(\mathbf{L5})_4](NO_3)_5$, **4e** monitoring the reaction progress at 70 °C (iii) 10 min (iv) 20 min the complex $[NO_3\subset Pd_4(\mathbf{L3})_2(\mathbf{L5})_4](NO_3)_7$, **4a**.

Fusion of 6a and 4e: Feasible to form 5a

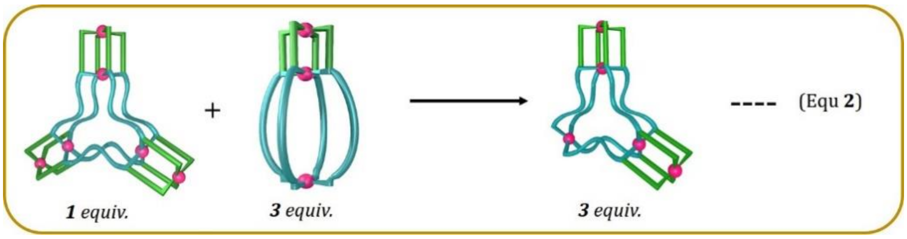

Solution 1: To the solution of ligand **L6** (1.18 mg, 0.0019 mmol, 6 equiv.) in 0.5 mL of DMSO-d_6, $Pd(NO_3)_2$ (0.46 mg, 0.0019 mmol, 6 equiv.) was added and stirred at 70 °C for 20 mins to obtain the hexanuclear complex $[(NO_3)_3 \subset Pd_6(\mathbf{L6})_6](NO_3)_9$, **6a**.

Solution 2: To a solution of ligand **L5** (1.82 mg, 0.004 mmol, 6 equiv.) in 0.5 mL of DMSO-d_6, $Pd(NO_3)_2$ (0.69 mg, 0.003 mmol, 4.5 equiv.) was added and stirred at 70 °C for 1 h to obtain the proposed complex $[NO_3 \subset Pd_3(\mathbf{L5})_4](NO_3)_5$, **4e** and $[(NO_3)_2 \subset Pd_6(\mathbf{L5})_8](NO_3)_{10}$, **4f**.

The heteroleptic complex $[(NO_3)_2 \subset Pd_5(\mathbf{L5})_4(\mathbf{L6})_2](NO_3)_8$, **5a** was obtained by stirring the mixture of the two solutions as mentioned above containing the complexes **4e, 4f** and **6a** for 3 h at 70 °C.

Formation of **5a** not feasible at room temperature

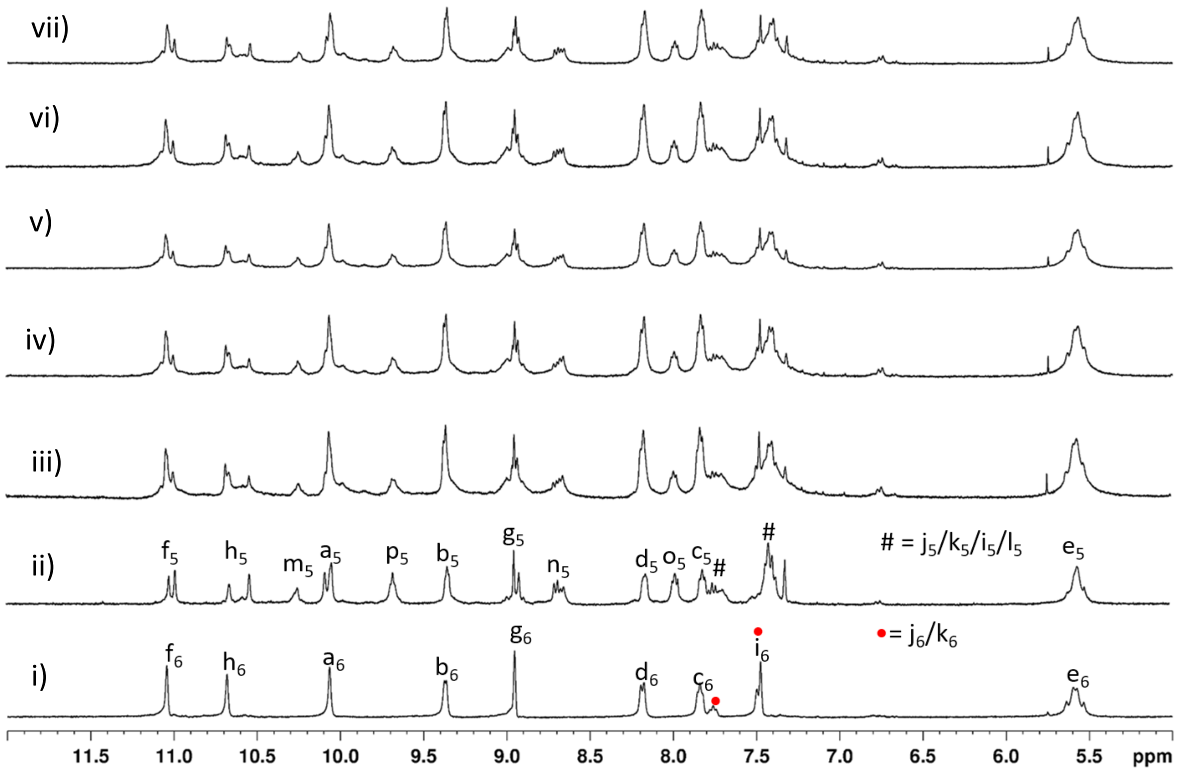

Supplementary Figure 108. Partial ^{1}H NMR spectra (400 MHz, DMSO-*d*$_6$, 300 K) of: (i) hexanuclear complex $[(NO_3)_3\subset Pd_6(\mathbf{L6})_6](NO_3)_9$, **6a** (ii) proposed complex $[NO_3\subset Pd_3(\mathbf{L5})_4](NO_3)_5$, **4e**; (iii)-(vii) After mixing hexanuclear complex $[(NO_3)_3\subset Pd_6(\mathbf{L6})_6](NO_3)_9$, **6a** and proposed complex $[NO_3\subset Pd_3(\mathbf{L5})_4](NO_3)_5$, **4e** monitoring the reaction after (iii) 6 h (iv) 24 h., (v) 72 h., (vi) 5 days., (vii) 15 days there was no visible change.

Formation of **5a** at 70 °C

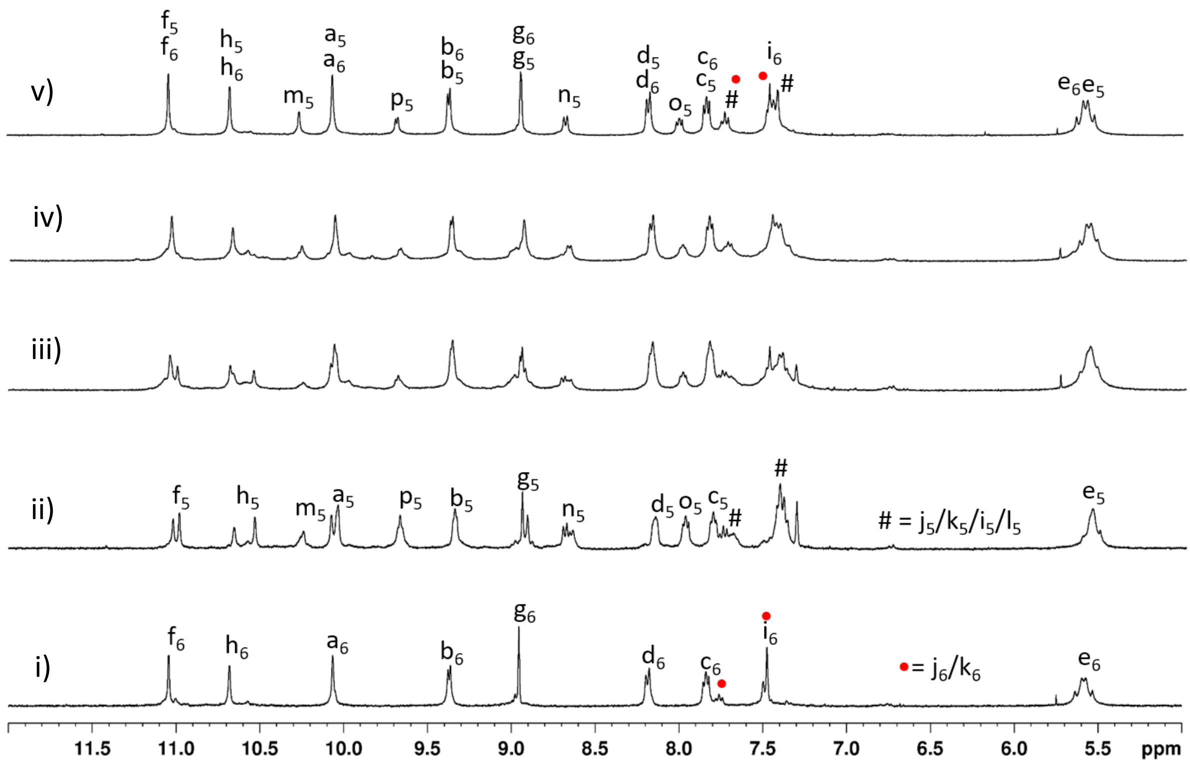

Supplementary Figure 109. Partial ^{1}H NMR spectra (400 MHz, DMSO-d_6, 300 K) of: (i) hexanuclear complex [(NO$_3$)$_3$⊂Pd$_6$(**L6**)$_6$](NO$_3$)$_9$, **6a** (ii) proposed complex [NO$_3$⊂Pd$_3$(**L5**)$_4$](NO$_3$)$_5$, **4e**; (iii)-(vii) After mixing hexanuclear complex [(NO$_3$)$_3$⊂Pd$_6$(**L6**)$_6$](NO$_3$)$_9$, **6a** and proposed complex [NO$_3$⊂Pd$_3$(**L5**)$_4$](NO$_3$)$_5$, **4e** monitoring the reaction after heating at 70 °C (iii) 1 h (iv) 2 h (v) 3 h complex [(NO$_3$)$_2$⊂Pd$_5$(**L5**)$_4$(**L6**)$_2$](NO$_3$)$_8$, **5a**.

Fusion of 3a and 6a: Not feasible and no changes

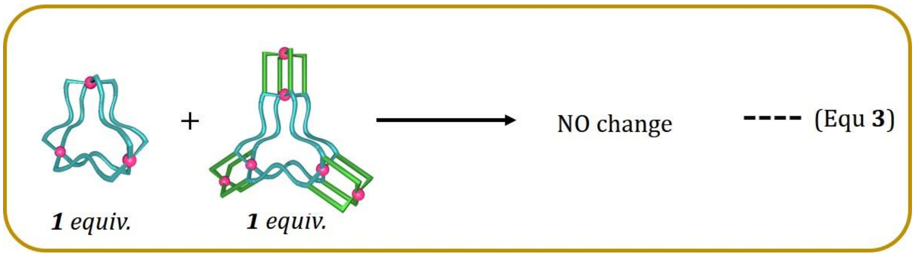

Solution 1: To the solution of ligand **L3** (3.20 mg, 0.010 mmol, 6 equiv.) in 0.5 mL of DMSO-*d*$_6$, Pd$(NO_3)_2$ (1.15 mg, 0.005 mmol, 3 equiv.) was added and stirred at room temperature for 10 mins to obtain the trinuclear complex [Pd$_3$(**L3**)$_6$]$(NO_3)_6$, **3**a.

Solution 2: Ligand **L6** (2.95 mg, 0.005 mmol, 6 equiv.) was dissolved in 0.5 mL of DMSO-*d*$_6$, to which Pd$(NO_3)_2$ (1.15 mg, 0.005 mmol, 6 equiv.) was added and stirred at 70 °C for 20 mins to obtain the complex [$(NO_3)_3$⊂Pd$_6$(**L6**)$_6$]$(NO_3)_9$, **6a**.

Upon stirring the mixture of the two solutions as mentioned above **(**containing the complexes **3a** and **6a)** for 24 h at 70 °C there was no appreciable change observed.

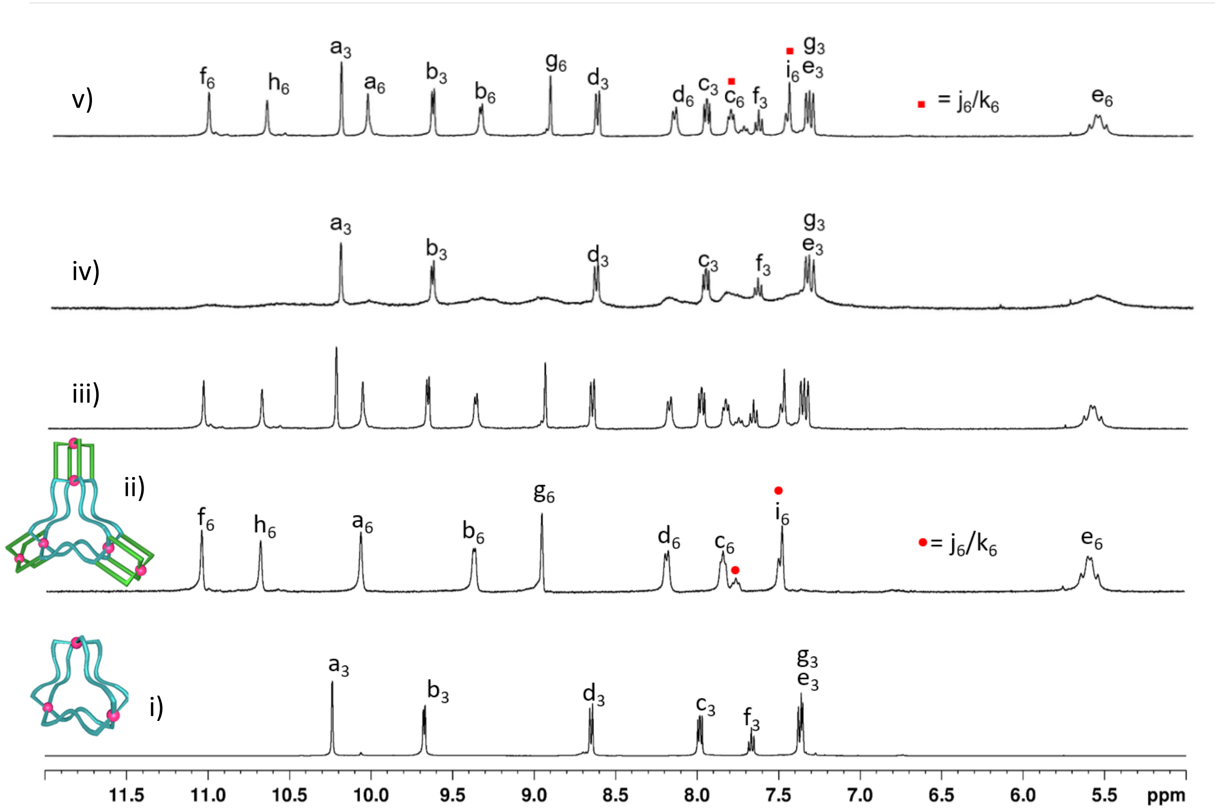

Supplementary Figure 110. Partial ^{1}H NMR spectra (400 MHz, DMSO-d_6, 300 K) of: (i) trinuclear complex [Pd_3(**L3**)$_6$](NO_3)$_6$, **3**a (ii) hexanuclear complex [(NO_3)$_3$⊂Pd_6(**L6**)$_6$](NO_3)$_9$, **6a**; (iii) after mixing trinuclear complex [Pd_3(**L3**)$_6$](NO_3)$_6$, **3**a and hexanuclear complex [(NO_3)$_3$⊂Pd_6(**L6**)$_6$](NO_3)$_9$, **6a** and heating at 70 °C for 12 h, no change was observed, (iv) ligand **L3** and **L6** with Pd(NO_3)$_2$ at 1:2 ratio mixed immediately and recorded after 5 mins; (v) after heating the mixture at 70 °C for 12 h resulted a mixture of trinuclear complex [Pd_3(**L3**)$_6$](NO_3)$_6$, **3**a and hexanuclear complex [(NO_3)$_3$⊂Pd_6(**L6**)$_6$](NO_3)$_9$, **6a**.

Fusion of 4a and 5a: Not feasible and no changes

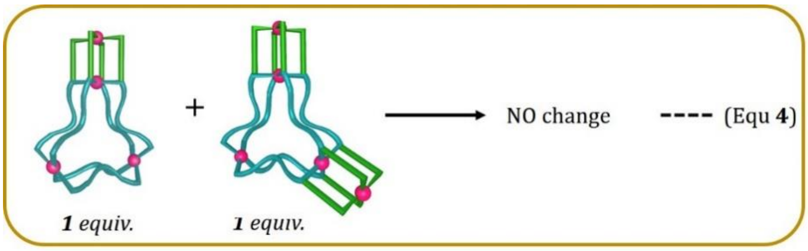

Solution 1: Mixture of ligand **L5** (2.28 mg, 0.005 mmol, 4 equiv.) and **L3** (0.80 mg, 0.0025 mmol, 2 equiv.) dissolved in 0.5 mL of DMSO-d_6, to which solution $Pd(NO_3)_2$ (1.15 mg, 0.005 mmol, 4 equiv.) was added and stirred at 70 °C for 1 h to obtain the complex $[(NO_3)\subset Pd_4(\mathbf{L3})_2(\mathbf{L5})_4](NO_3)_7$, **4a**.

Solution 2: Ligands **L5** (1.82 mg, 0.004 mmol, 4 equiv.) and **L6** (1.18 mg, 0.002 mmol, 2 equiv.) were dissolved in 0.5 mL of DMSO-d_6, to which $Pd(NO_3)_2$ (1.15 mg, 0.005 mmol, 5 equiv.) was added and stirred at 70 °C for 1 h to obtain the complex $[(NO_3)_2\subset Pd_5(\mathbf{L5})_4(\mathbf{L6})_2](NO_3)_8$, **5a**.

Upon stirring the mixture of the two solutions as mentioned above (containing the complexes **4a** and **5a)** for 24 h at 70 °C there was no appreciable change observed.

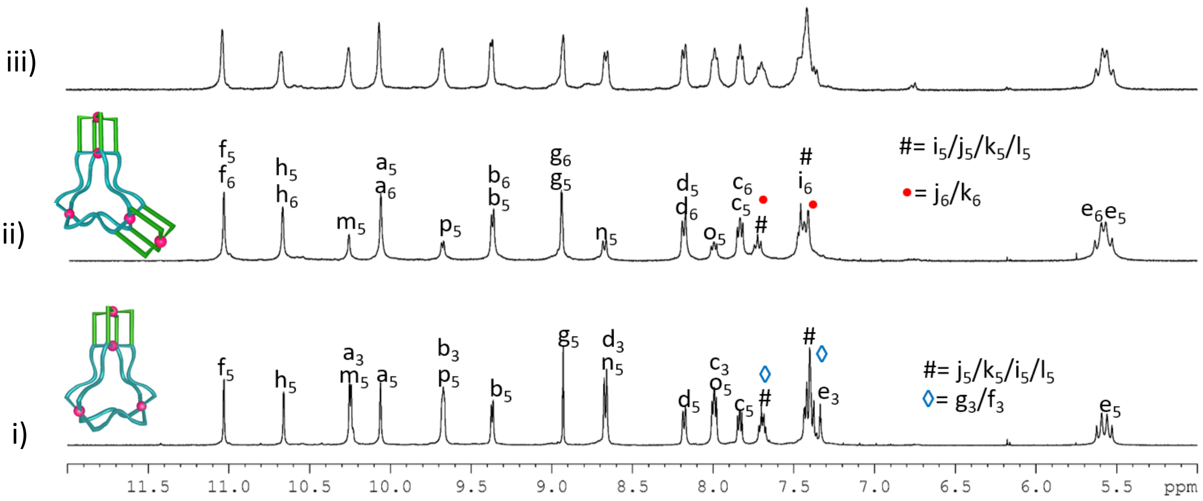

Supplementary Figure 111. Partial ^{1}H NMR spectra (400 MHz, DMSO-d_6, 300 K) of: (i) complex $[(NO_3)\subset Pd_4(\mathbf{L3})_2(\mathbf{L5})_4](NO_3)_7$, **4a**; (ii) pentanuclear complex $[(NO_3)_2\subset Pd_5(\mathbf{L5})_4(\mathbf{L6})_2](NO_3)_8$, **5a**; (iii) after mixing $[(NO_3)\subset Pd_4(\mathbf{L3})_2(\mathbf{L5})_4](NO_3)_7$, **4a** and pentanuclear complex $[(NO_3)_2\subset Pd_5(\mathbf{L5})_4(\mathbf{L6})_2](NO_3)_8$, **5a**; and heating at 70 °C for 12 h, no change was observed.

Fusion of 3a and 4a: Not feasible and no changes

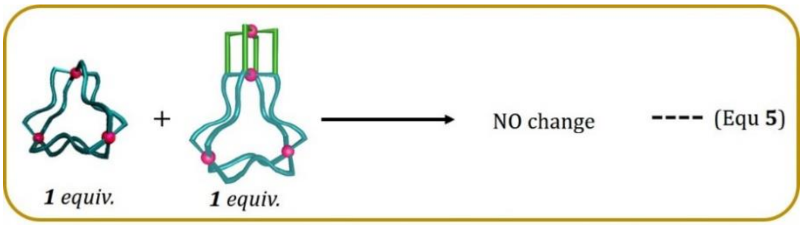

Solution 1: To the solution of ligand **L3** (3.20 mg, 0.010 mmol, 6 equiv.) in 0.5 mL of DMSO-d_6, $Pd(NO_3)_2$ (1.15 mg, 0.005 mmol, 3 equiv.) was added and stirred at room temperature for 10 mins to obtain the trinuclear complex $[Pd_3(\mathbf{L3})_6](NO_3)_6$, **3**a.

Solution 2: Ligand **L5** (2.28 mg, 0.005 mmol, 4 equiv.) and **L3** (0.80 mg, 0.0025 mmol, 2 equiv.) were dissolved in 0.5 mL of DMSO-d_6, to which $Pd(NO_3)_2$ (1.15 mg, 0.005 mmol, 4 equiv.) was added and stirred at 70 °C for 1 h to obtain the complex $[NO_3{\subset}Pd_4(\mathbf{L3})_2(\mathbf{L5})_4](NO_3)_7$, **4a**.

Upon stirring the mixture of the two solutions as mentioned above (containing the complexes **3a** and **4a)** for 24 h at 70 °C there was no appreciable change observed.

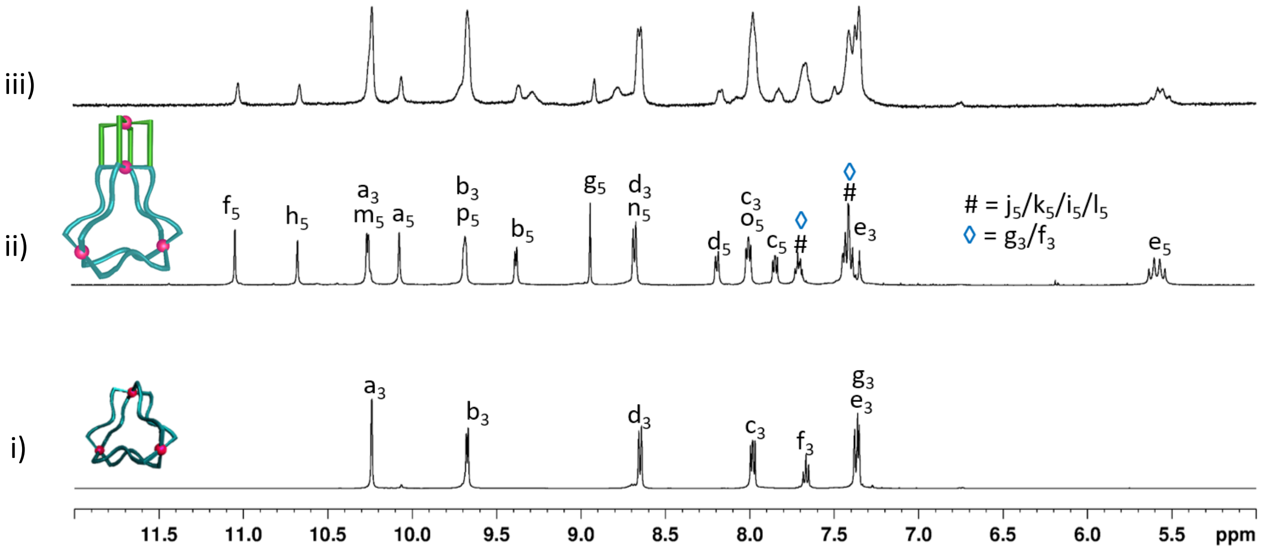

Supplementary Figure 112. Partial ^{1}H NMR spectra (400 MHz, DMSO-d_6, 300 K) of: (i) trinuclear complex $[Pd_3(\mathbf{L3})_6](NO_3)_6$, **3**a (ii) complex $[(NO_3){\subset}Pd_4(\mathbf{L3})_2(\mathbf{L5})_4](NO_3)_7$, **4a**; (iii) after mixing trinuclear complex $[Pd_3(\mathbf{L3})_6](NO_3)_6$, **3**a and complex $[(NO_3){\subset}Pd_4(\mathbf{L3})_2(\mathbf{L5})_4](NO_3)_7$, **4a** and heating at 70 °C for 12 h, no change was observed.

Fusion of 3a and 5a: Not feasible and no changes

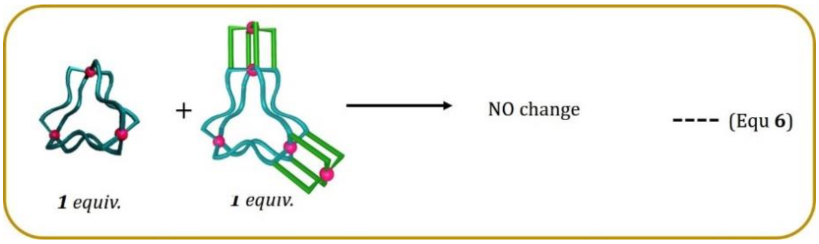

Solution 1: To the solution of ligand **L3** (3.20 mg, 0.010 mmol, 6 equiv.) in 0.5 mL of DMSO-*d*$_6$, Pd(NO_3)$_2$ (1.15 mg, 0.005 mmol, 3 equiv.) was added and stirred at room temperature for 10 mins to obtain the trinuclear complex [Pd$_3$(**L3**)$_6$](NO_3)$_6$, **3**a.

Solution 2: Ligand **L5** (1.82 mg, 0.004 mmol, 4 equiv.) and **L6** (1.18 mg, 0.002 mmol, 2 equiv.) were dissolved in 0.5 mL of DMSO-*d*$_6$, to which Pd(NO_3)$_2$ (1.15 mg, 0.005 mmol, 5 equiv.) was added and stirred at 70 °C for 1 h to obtain the complex [(NO_3)$_2$⊂Pd$_5$(**L5**)$_4$(**L6**)$_2$](NO_3)$_8$, **5a**.

Upon stirring the mixture of the two solutions as mentioned above (containing the complexes **3a** and **5a)** for 24 h at 70 °C there was no appreciable change observed.

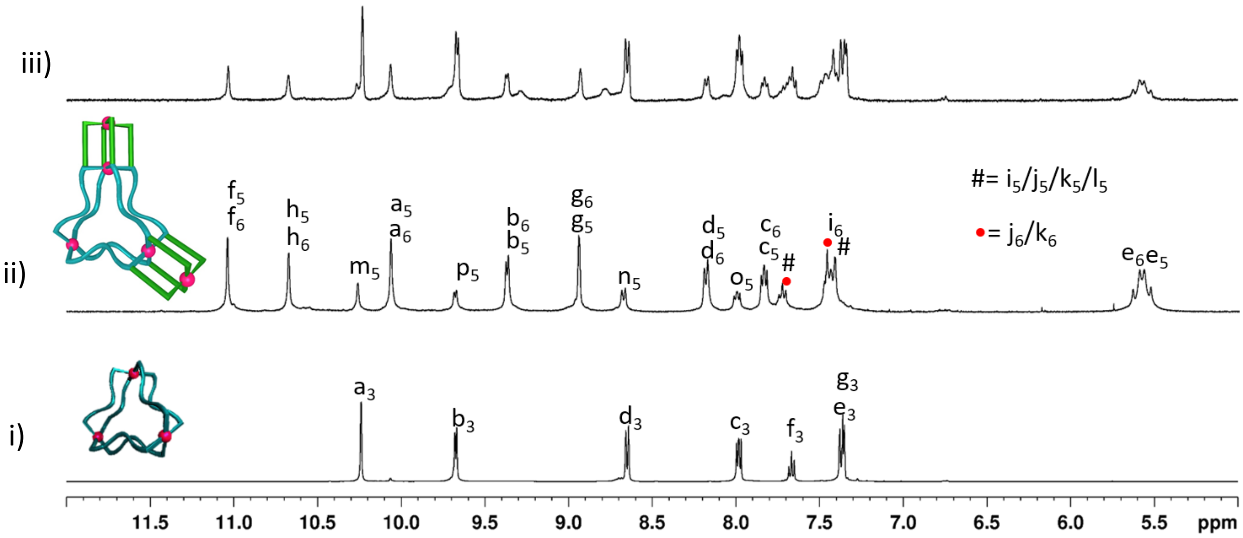

Supplementary Figure 113. Partial ^{1}H NMR spectra (400 MHz, DMSO-*d*$_6$, 300 K) of: (i) trinuclear complex [Pd$_3$(**L3**)$_6$](NO_3)$_6$, **3**a (ii) pentanuclear complex [(NO_3)$_2$⊂Pd$_5$(**L5**)$_4$(**L6**)$_2$](NO_3)$_8$, **5a**; (iii) after mixing trinuclear complex [Pd$_3$(**L3**)$_6$](NO_3)$_6$, **3**a and pentanuclear complex [(NO_3)$_2$⊂Pd$_5$(**L5**)$_4$(**L6**)$_2$](NO_3)$_8$, **5a** and heating at 70 °C for 12 h.

Fusion of 4a and 4e: Not feasible and no changes

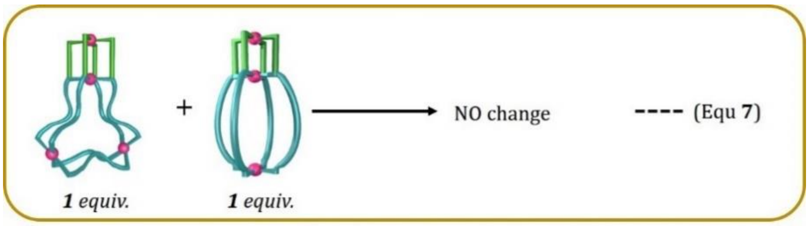

Solution 1: Mixture of ligand **L5** (2.28 mg, 0.005 mmol, 4 equiv.) and **L3** (0.80 mg, 0.0025 mmol, 2 equiv.) was dissolved in 0.5 mL of DMSO-d_6, to which $Pd(NO_3)_2$ (1.15 mg, 0.005 mmol, 4 equiv.) was added and stirred at 70 °C for 1 h to obtain the complex $[NO_3\subset Pd_4$(**L3**)$_2$(**L5**)$_4](NO_3)_7$, **4a**.

Solution 2: To another solution of ligand **L5** (3.02 mg, 0.007 mmol, 4 equiv.) in 0.5 mL of DMSO-d_6, $Pd(NO_3)_2$ (1.15 mg, 0.005 mmol, 3 equiv.) was added and stirred at 70 °C for 1 h to obtain the proposed complexes $[NO_3\subset Pd_3$(**L5**)$_4](NO_3)_5$, **4e** and $[(NO_3)_2\subset Pd_6$(**L5**)$_8](NO_3)_{10}$, **4f**.

Upon stirring the mixture of the two solutions as mentioned above containing the complexes **4a** and **4e** for 24 h at 70 °C there was no appreciable change observed.

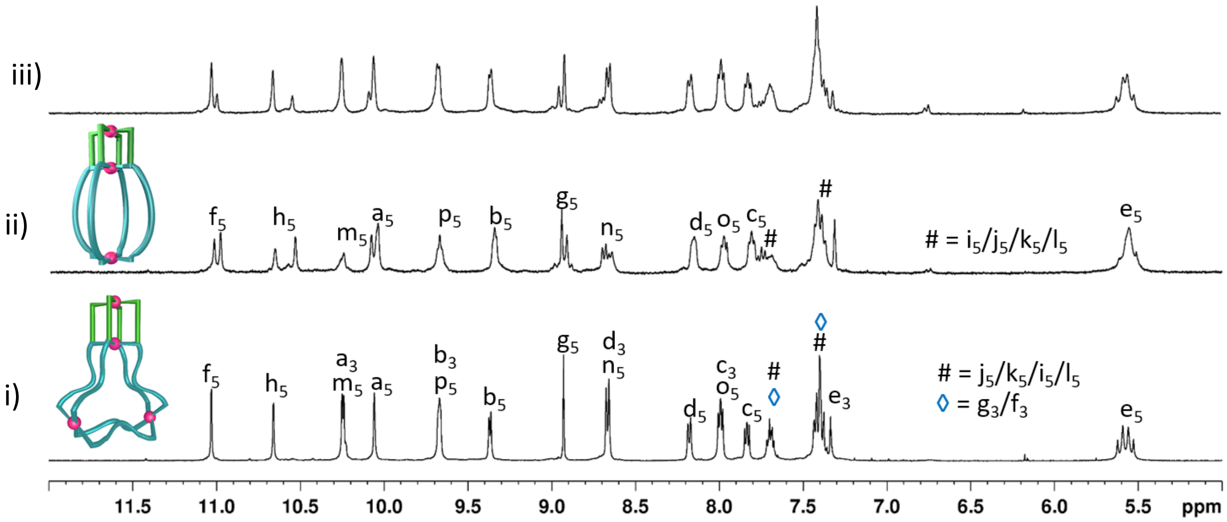

Supplementary Figure 114. Partial ^{1}H NMR spectra (400 MHz, DMSO-d_6, 300 K) of: (i) complex $[(NO_3)\subset Pd_4$(**L3**)$_2$(**L5**)$_4](NO_3)_7$, **4a**; (ii) proposed complex $[NO_3\subset Pd_3$(**L5**)$_4](NO_3)_5$, **4e**; (iii) after mixing tetranuclear complex $[NO_3\subset Pd_4$(**L3**)$_2$(**L5**)$_4](NO_3)_7$, **4a** and proposed complex $[NO_3\subset Pd_3$(**L5**)$_4](NO_3)_5$, **4e**; and heating at 70 °C for 12 h, no change was observed.

Fusion of 5a and 4e: Not feasible and no changes

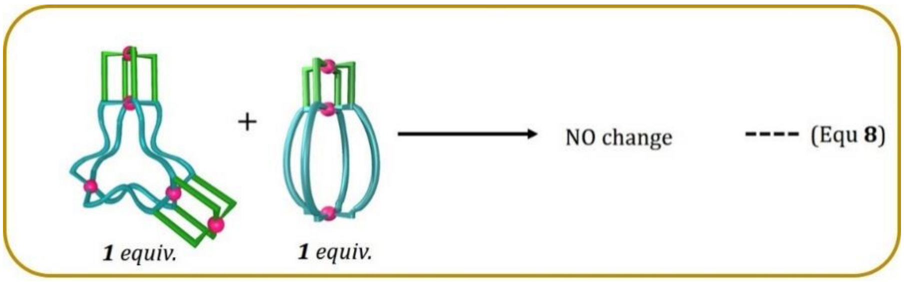

Solution 1: Mixture of ligand **L5** (1.82 mg, 0.004 mmol, 4 equiv.) and **L6** (1.18 mg, 0.002 mmol, 2 equiv.) was dissolved in 0.5 mL of DMSO-d_6, to which Pd$(NO_3)_2$ (1.15 mg, 0.005 mmol, 5 equiv.) was added and stirred at 70 °C for 1 h to obtain the complex $[(NO_3)_2 \subset Pd_5$(**L5**)$_4$(**L6**)$_2](NO_3)_8$, **5a**.

Solution 2: To another solution of ligand **L5** (3.02 mg, 0.007 mmol, 4 equiv.) in 0.5 mL of DMSO-d_6, Pd$(NO_3)_2$ (1.15 mg, 0.005 mmol, 3 equiv.) was added and stirred at at 70 °C for 1 h to obtain the proposed complexes $[NO_3 \subset Pd_3$(**L5**)$_4](NO_3)_5$, **4e** and $[(NO_3)_2 \subset Pd_6$(**L5**)$_8](NO_3)_{10}$, **4f**.

Upon stirring the mixture of the two solutions as mentioned above containing the complexes **5a** and **4e** for 24 h at 70 °C there was no appreciable change observed.

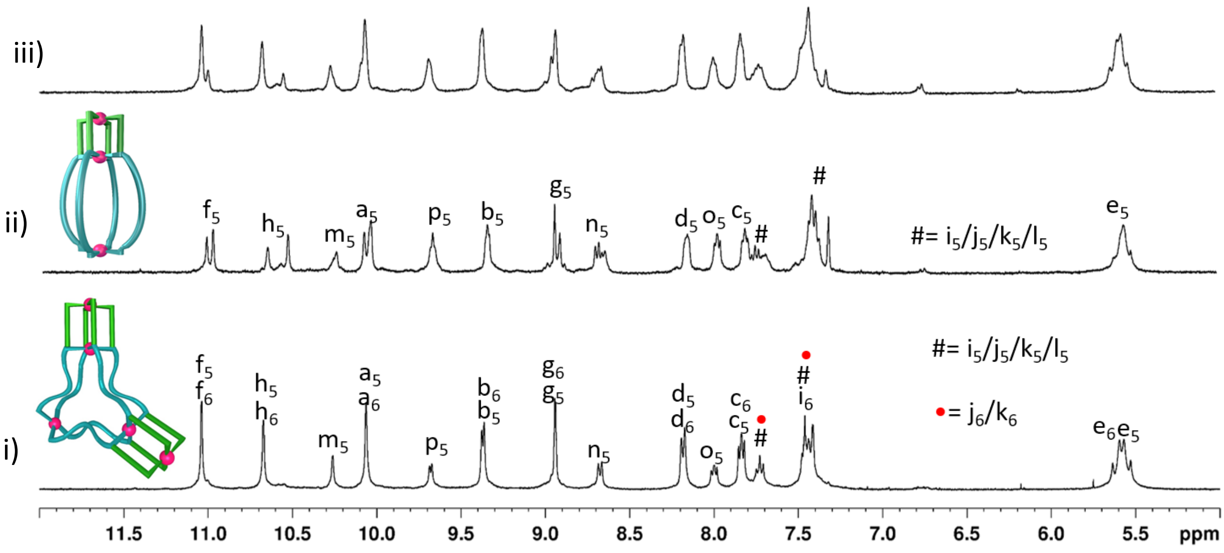

Supplementary Figure 115. Partial ^{1}H NMR spectra (400 MHz, DMSO-d_6, 300 K) of: (i) pentanuclear complex $[(NO_3)_2 \subset Pd_5$(**L5**)$_4$(**L6**)$_2](NO_3)_8$, **5a**; (ii) proposed complex $[NO_3 \subset Pd_3$(**L5**)$_4](NO_3)_5$, **4e**; (iii) after mixing pentanuclear complex $[(NO_3)_2 \subset Pd_5$(**L5**)$_4$(**L6**)$_2](NO_3)_8$, **5a** and proposed complex $[NO_3 \subset Pd_3$(**L5**)$_4](NO_3)_5$, **4e**; and heating at 70 °C for 12 h, no change was observed.

Fusion of 4a and 6a: Not feasible and no changes

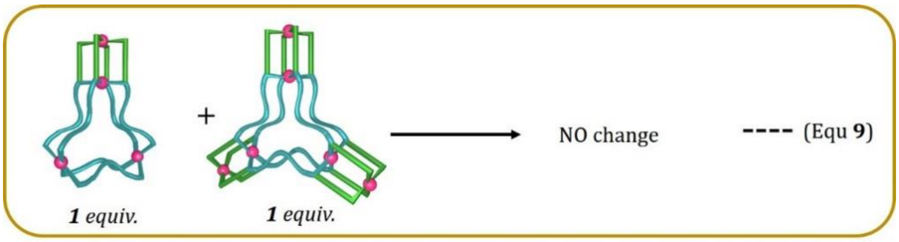

Solution 1: Mixture of ligand **L5** (2.28 mg, 0.005 mmol, 4 equiv.) and **L3** (0.80 mg, 0.0025 mmol, 2 equiv.) was dissolved in 0.5 mL of DMSO-d_6, to which $Pd(NO_3)_2$ (1.15 mg, 0.005 mmol, 4 equiv.) was added and stirred at 70 °C for 1 h to obtain the complex $[(NO_3)\subset Pd_4(\mathbf{L3})_2(\mathbf{L5})_4](NO_3)_7$, **4a**.

Solution 2: To another solution of ligand **L6** (2.95 mg, 0.005 mmol, 6 equiv.) was dissolved in 0.5 mL of DMSO-d_6, $Pd(NO_3)_2$ (1.15 mg, 0.005 mmol, 6 equiv.) was added and stirred at 70 °C for 20 min to obtain the complex $[(NO_3)_3\subset Pd_6(\mathbf{L6})_6](NO_3)_9$, **6a**.

Upon stirring the mixture of the two solutions as mentioned above (containing the complexes **4a** and **6a**) for 24 h at 70 °C there was no appreciable change observed.

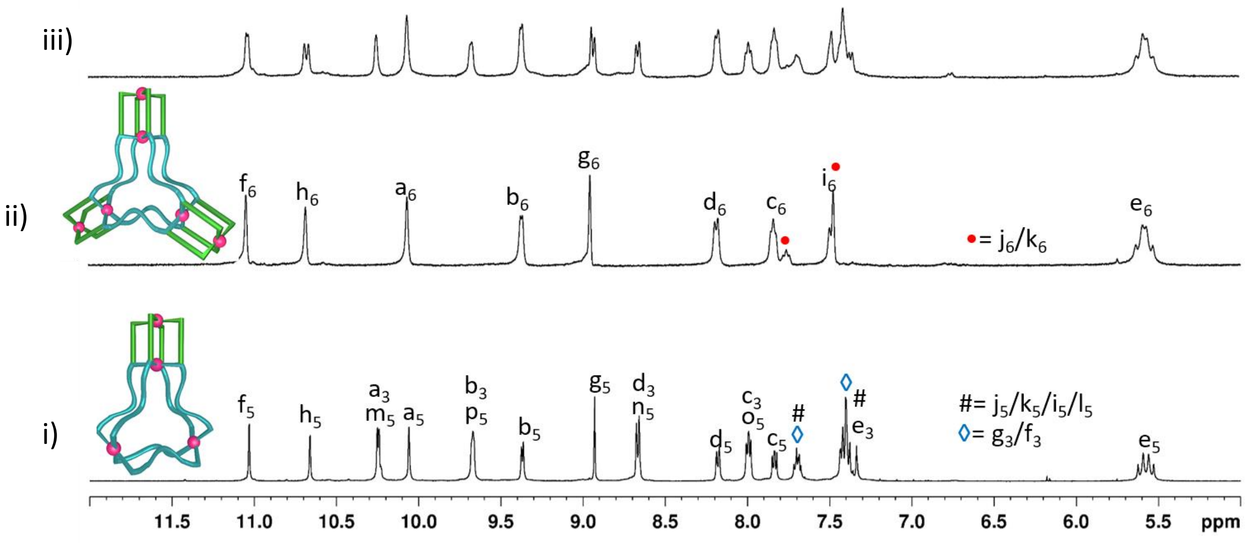

Supplementary Figure 116. Partial ^{1}H NMR spectra (400 MHz, DMSO-d_6, 300 K) of: (i) complex $[NO_3\subset Pd_4(\mathbf{L3})_2(\mathbf{L5})_4](NO_3)_7$, **4a**; (ii) hexanuclear complex $[(NO_3)_3\subset Pd_6(\mathbf{L6})_6](NO_3)_9$, **6a**; (iii) after mixing $[NO_3\subset Pd_4(\mathbf{L3})_2(\mathbf{L5})_4](NO_3)_7$, **4a** and hexanuclear complex $[(NO_3)_3\subset Pd_6(\mathbf{L6})_6](NO_3)_9$, **6a**; and heating at 70 °C for 12 h.

Fusion of 6a and 5a: Not feasible and no changes

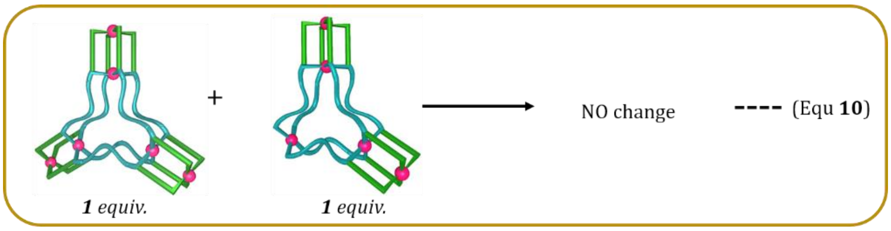

Solution 1: To a solution of ligand **L6** (2.95 mg, 0.005 mmol, 6 equiv.) in 0.5 mL of DMSO-*d*$_6$, Pd(NO_3)$_2$ (1.15 mg, 0.005 mmol, 6 equiv.) was added and stirred at 70 °C for 20 min to obtain the complex [(NO_3)$_3$⊂Pd$_6$(**L6**)$_6$](NO_3)$_9$, **6a**.

Solution 2: Mixture of ligand **L5** (1.82 mg, 0.004 mmol, 4 equiv.) and **L6** (1.18 mg, 0.002 mmol, 2 equiv.) was dissolved in 0.5 mL of DMSO-*d*$_6$, to which Pd(NO_3)$_2$ (1.15 mg, 0.005 mmol, 5 equiv.) was added and stirred at 70 °C for 1 h to obtain the complex [(NO_3)$_2$⊂Pd$_5$(**L5**)$_4$(**L6**)$_2$](NO_3)$_8$, **5a**.

Upon stirring the mixture of the two solutions as mentioned above (containing the complexes **5a** and **6a**) for 24 h at 70 °C there was no appreciable change observed.

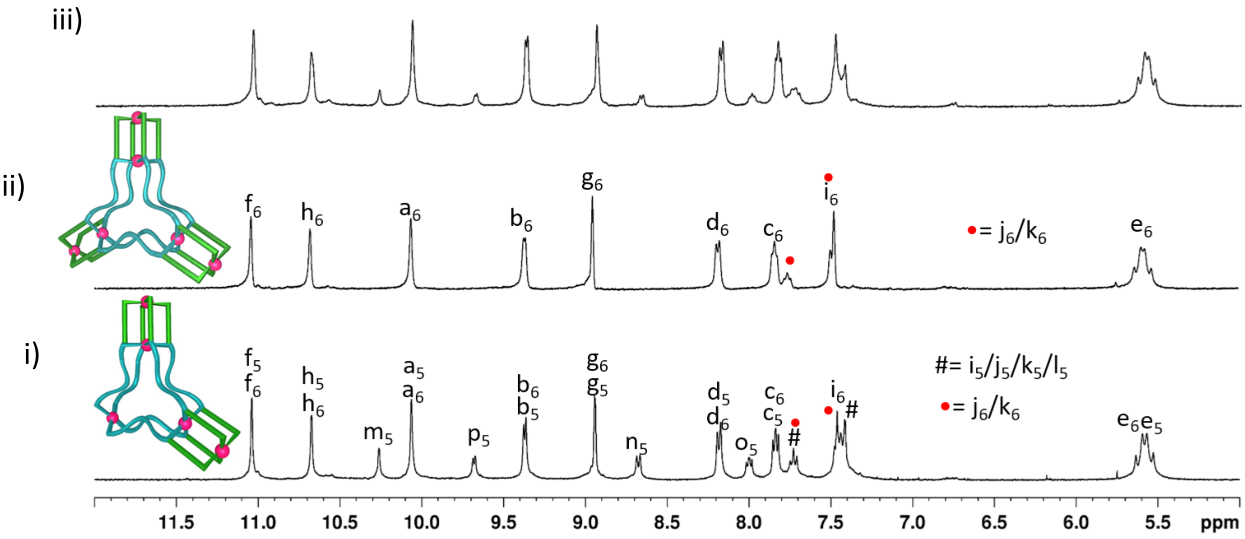

Supplementary Figure 117. Partial ^{1}H NMR spectra (400 MHz, DMSO-*d*$_6$, 300 K) of: (i) pentanuclear complex [(NO_3)$_2$⊂Pd$_5$(**L5**)$_4$(**L6**)$_2$](NO_3)$_8$, **5a**; (ii) hexanuclear complex [(NO_3)$_3$⊂Pd$_6$(**L6**)$_6$](NO_3)$_9$, **6a**; (iii) after mixing pentanuclear complex [(NO_3)$_2$⊂Pd$_5$(**L5**)$_4$(**L6**)$_2$](NO_3)$_8$, **5a** and hexanuclear complex [(NO_3)$_3$⊂Pd$_6$(**L6**)$_6$](NO_3)$_9$, **6a**; and heating at 70 °C for 12 h, no change was observed.

Ligand-displacement-induced cage-to-cage transformations

Cartoon representation of ligands and cationic portions of cages

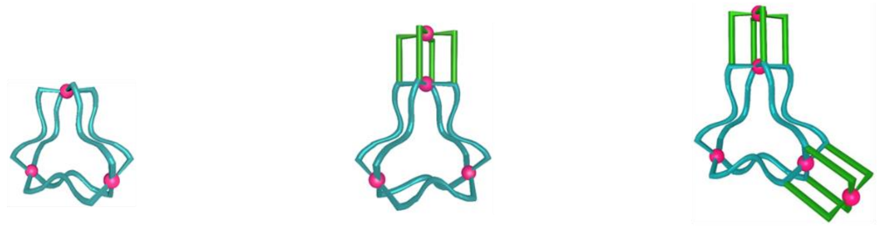

$[Pd_3(L3)_6](NO_3)_6$, **3a** $[NO_3\subset Pd_4(L3)_2(L5)_4](NO_3)_7$, **4a** $[(NO_3)_2\subset Pd_5(L5)_4(L6)_2](NO_3)_8$, **5a**

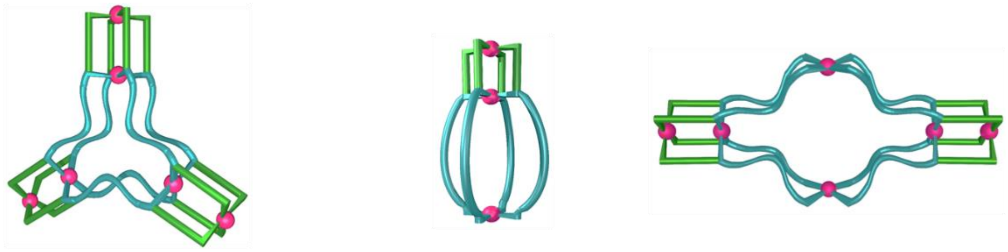

$[(NO_3)_3\subset Pd_6(L6)_6](NO_3)_9$, **6a** $[NO_3\subset Pd_3(L5)_4](NO_3)_5$, **4e** $[(NO_3)_2\subset Pd_6(L5)_8](NO_3)_{10}$, **4f**

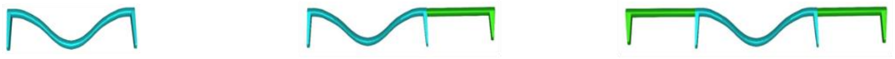

L3 **L5** **L6**

The complexes **4e** and **4f** coexist, however, only **4e** is shown for simplicity.

Displacement reactions are shown below:

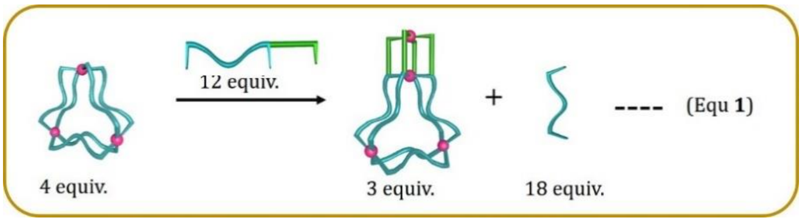

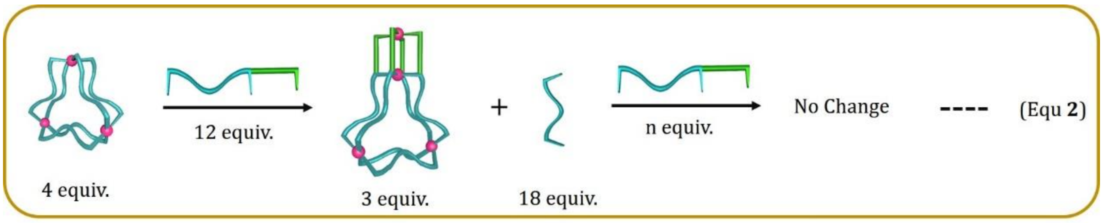

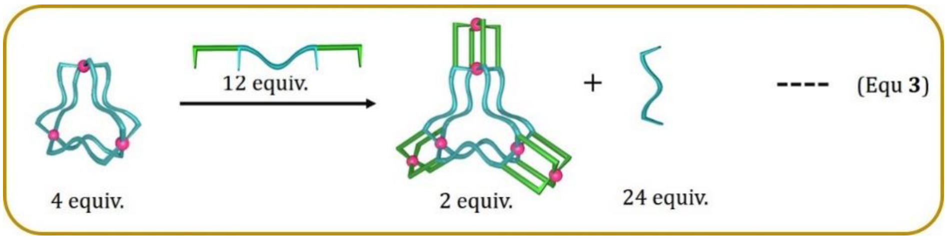

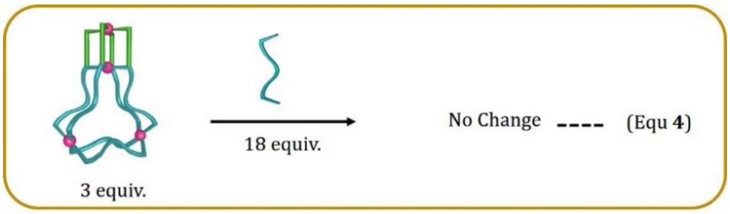
18 equiv.
No Change
(Equ 4)
3 equiv.

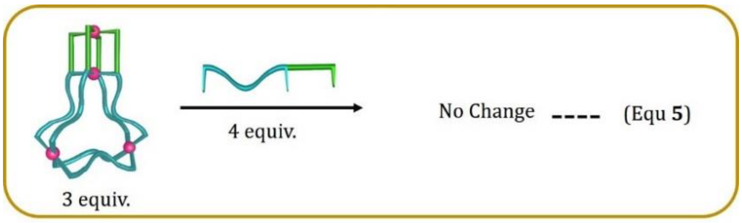
4 equiv.
No Change
(Equ 5)
3 equiv.

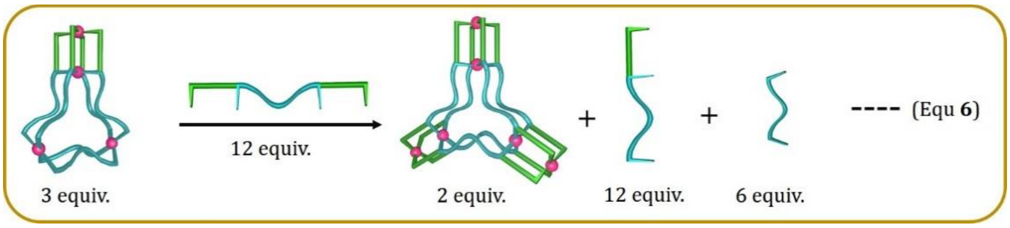
12 equiv.
(Equ 6)
3 equiv.
2 equiv.
12 equiv.
6 equiv.

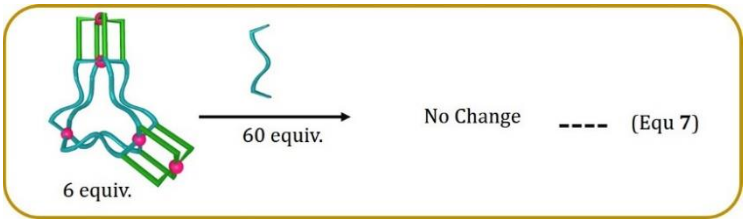
60 equiv.
No Change
(Equ 7)
6 equiv.

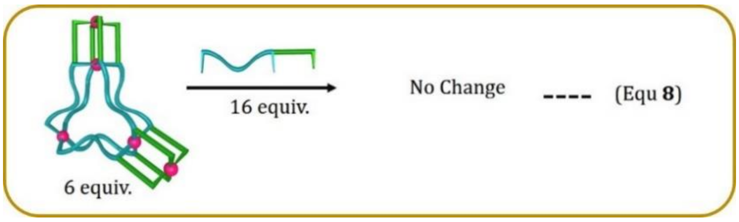
16 equiv.
No Change
(Equ 8)
6 equiv.

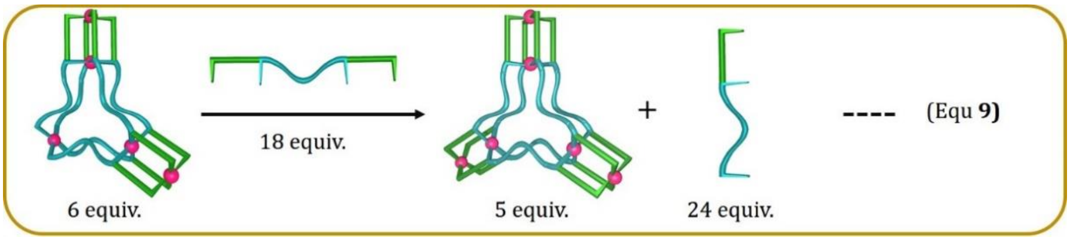
18 equiv.
(Equ 9)
6 equiv.
5 equiv.
24 equiv.

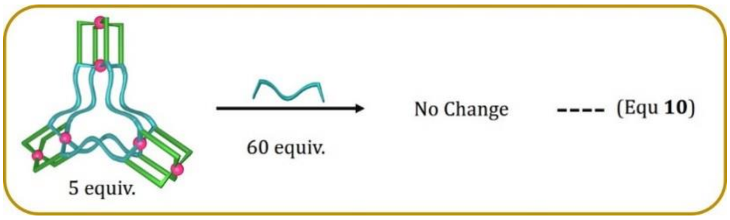
No Change
(Equ 10)
60 equiv.
5 equiv.

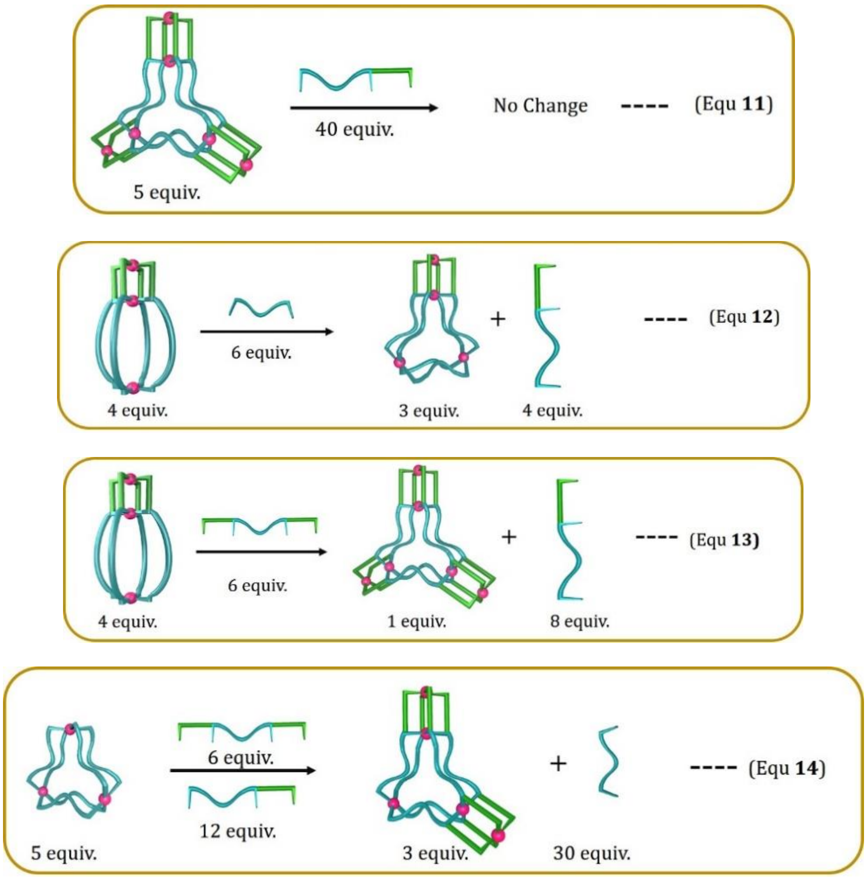
40 equiv.
No Change
---- (Equ **11**)
5 equiv.
6 equiv.
+
---- (Equ **12**)
4 equiv.
3 equiv.
4 equiv.
6 equiv.
+
---- (Equ **13**)
4 equiv.
1 equiv.
8 equiv.
6 equiv.
12 equiv.
+
---- (Equ **14**)
5 equiv.
3 equiv.
30 equiv.

Interaction of L5 with complex 3a: Feasible to form 4a and L3

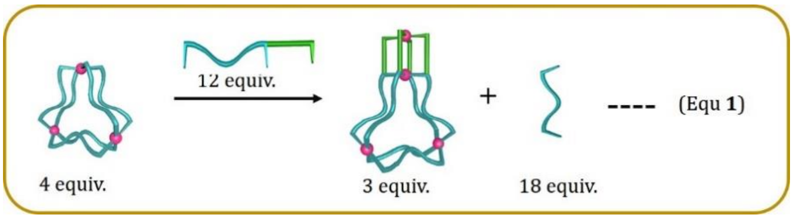

To the solution of ligand **L3** (3.20 mg, 0.010 mmol) in 0.5 mL of DMSO-d_6, $Pd(NO_3)_2$ (1.15 mg, 0.005 mmol) was added and stirred at room temperature for 10 min to obtain the trinuclear complex $[Pd_3($**L3**$)_6](NO_3)_6$, **3a**. To the formed trinuclear complex in DMSO-d_6, ligand **L5** (2.28 mg, 0.005 mmol)was added and heated at 70 °C for 1h, which led to the formation of heteroleptic complex $[(NO_3)\subset Pd_4($**L3**$)_2($**L5**$)_4](NO_3)_7$, **4a** with the release of free ligand **L3**.

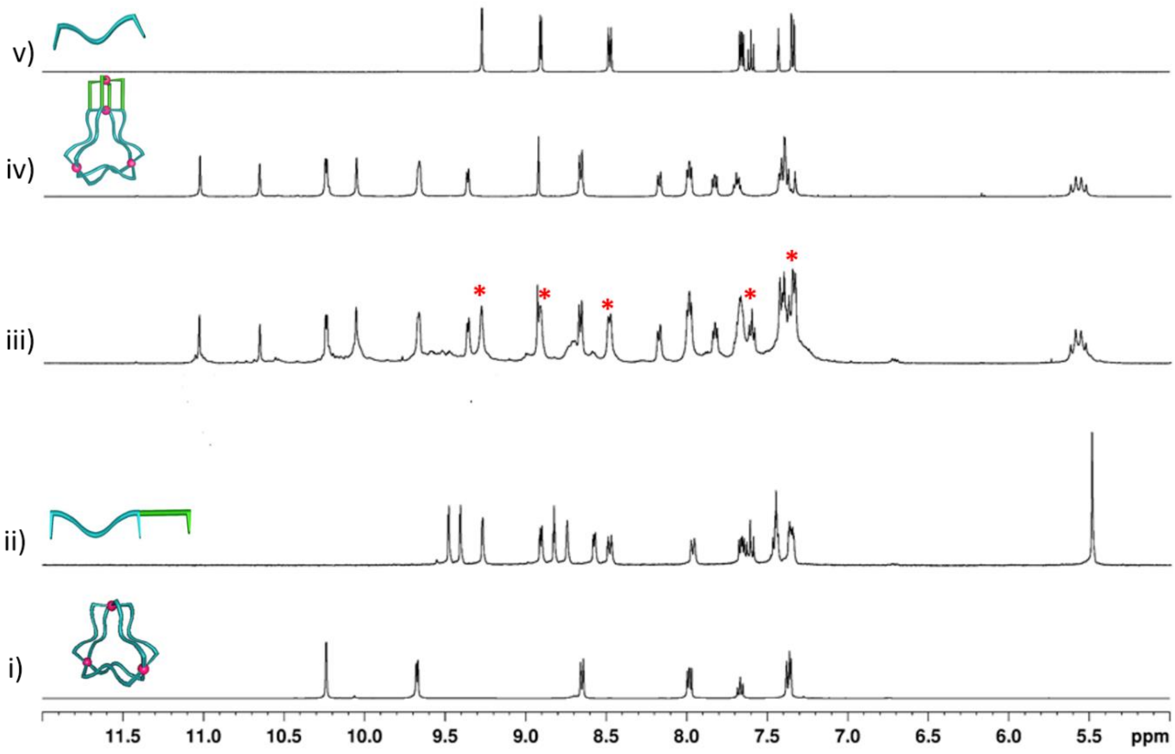

Supplementary Figure 118. Partial ^{1}H NMR spectra (400 MHz, DMSO-d_6, 300 K) of: (i) $[Pd_3($**L3**$)_6](NO_3)_6$, **3a**; (ii) ligand **L5**; (iii) after mixing ligand **L5** with **3a** and heating at 70 °C for 1 h, $[NO_3\subset Pd_4($**L3**$)_2($**L5**$)_4](NO_3)_7$, **4a** and free ligand **L3** (* represents free ligand **L3**) was obtained (iv) isolated $[NO_3\subset Pd_4($**L3**$)_2($**L5**$)_4](NO_3)_7$, **4a** and (v) **L3**.

Interaction of L5 with a mixture of complex 4a and L3 (of equation 1): No changes

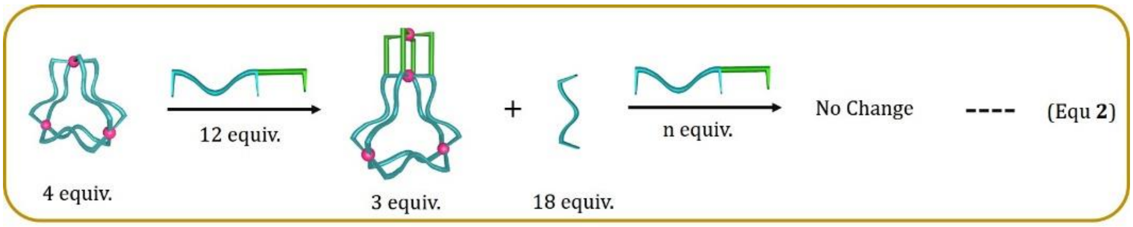

To the solution of ligand **L3** (3.20 mg, 0.010 mmol) in 0.5 mL of DMSO-d_6, $Pd(NO_3)_2$ (1.15 mg, 0.005 mmol) was added and stirred at room temperature for 10 min to obtain the trinuclear complex [Pd_3(**L3**)$_6$](NO_3)$_6$, **3a**. To the formed trinuclear complex in DMSO-d_6, **L5** (2.28 mg, 0.005 mmol)was added and heated at 70 °C for 1h, which led to the formation of heteroleptic complex [NO_3⊂Pd_4(**L3**)$_2$(**L5**)$_4$](NO_3)$_7$, **4a** with the release of free ligand **L3**. To this reaction mixture further excess of ligand **L5** (1.14 mg, 0.0025 mmol) was added and was heated at 70 °C for 6h, but no significant change was observed.

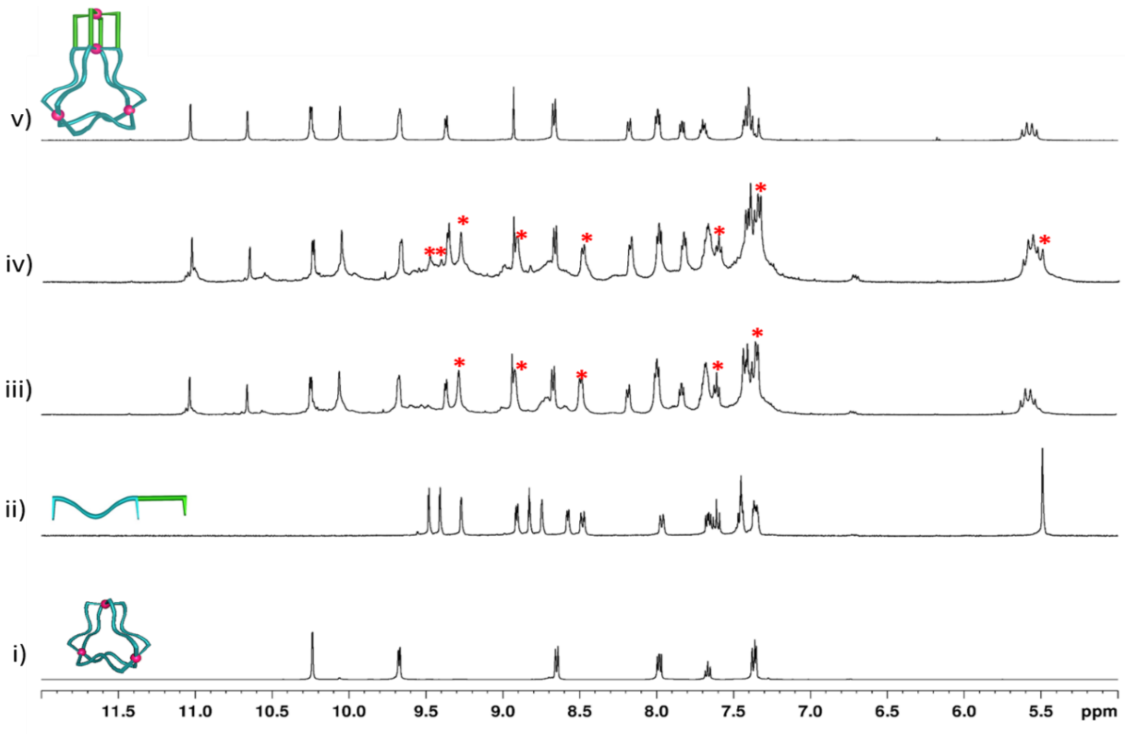

Supplementary Figure 119. Partial ^{1}H NMR spectra (400 MHz, DMSO-d_6, 300 K) of: (i) [Pd_3(**L3**)$_6$](NO_3)$_6$, **3a**; (ii) **L5**; (iii) after mixing **L5** with **3a** and heating at 70 °C for 1 h, [NO_3⊂Pd_4(**L3**)$_2$(**L5**)$_4$](NO_3)$_7$, **4a** and free ligand **L3** (* represents free ligand **L3**) was obtained (iv) after mixing excess **L5** with **3a** and heating at 70 °C for 6 h, [NO_3⊂Pd_4(**L3**)$_2$(**L5**)$_4$](NO_3)$_7$, **4a** remained in solution along with **L3** and **L5** (* represents free ligand **L3** and **L5**) and (v) isolated [NO_3⊂Pd_4(**L3**)$_2$(**L5**)$_4$](NO_3)$_7$, **4a**.

Interaction of L6 with complex 3a: Feasible to form 6a and L3

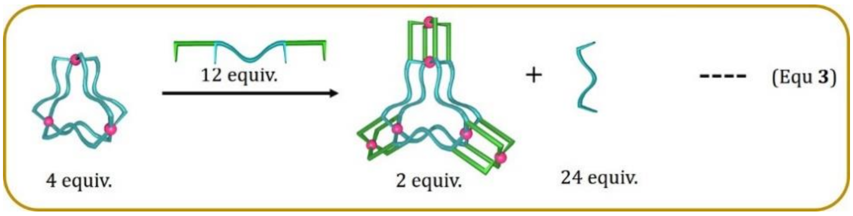

To the solution of ligand **L3** (3.20 mg, 0.010 mmol) in 0.5 mL of DMSO-d_6, $Pd(NO_3)_2$ (1.15 mg, 0.005 mmol) was added and stirred at room temperature for 10 min to obtain the trinuclear complex $[Pd_3($**L3**$)_6](NO_3)_6$, **3a**. To the formed trinuclear complex in DMSO-d_6, ligand **L6** (2.95 mg, 0.005 mmol) was added and heated at 70 °C for 1h, which led to the formation of complex $[(NO_3)_3\subset Pd_6($**L6**$)_6](NO_3)_9$, **6a** with the release of free ligand **L3**.

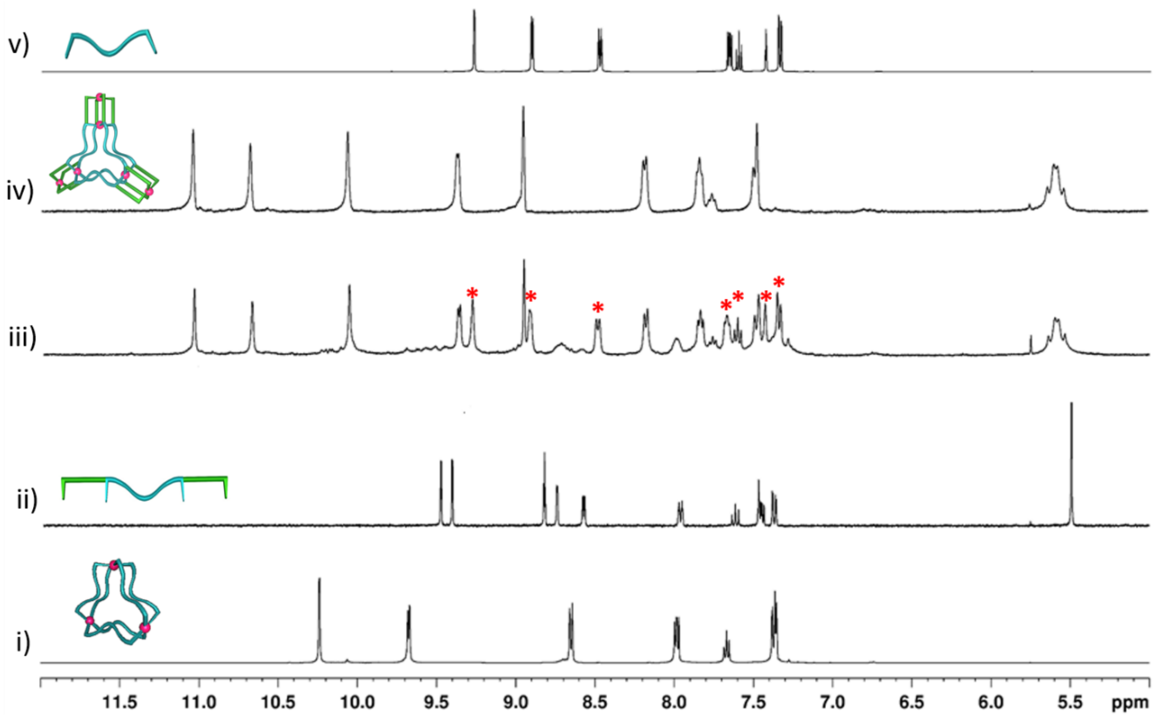

Supplementary Figure 120. Partial ^{1}H NMR spectra (400 MHz, DMSO-d_6, 300 K) of: (i) $[Pd_3($**L3**$)_6](NO_3)_6$, **3a**; (ii) **L6**; (iii) after mixing **L6** with **3a** and heating at 70 °C for 1 h, it gave $[(NO_3)_3\subset Pd_6($**L6**$)_6](NO_3)_9$, **6a** and free ligand **L3** (* represents free ligand **L3**) (iv) isolated $[(NO_3)_3\subset Pd_6($**L6**$)_6](NO_3)_7$, **6a** and (v) **L3**.

Interaction of L3 with complex 4a: No changes

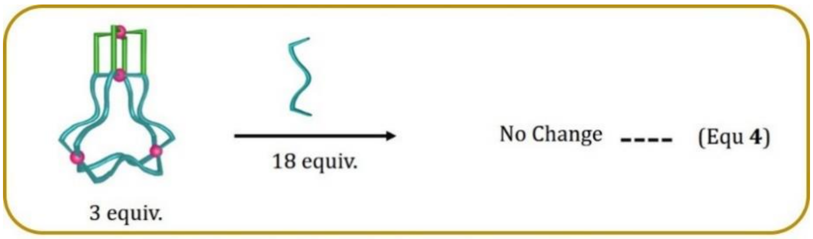

Mixture of ligands **L5** (2.28 mg, 0.005 mmol) and **L3** (0.80 mg, 0.0025 mmol) was dissolved in 0.5 mL of DMSO-d_6, to which solution $Pd(NO_3)_2$ (1.15 mg, 0.005 mmol) was added and stirred at 70 °C for 1 h to obtain the complex $[(NO_3)\subset Pd_4($**L3**$)_2($**L5**$)_4](NO_3)_7$, **4a**. To the formed complex **4a** in DMSO-d_6, ligand **L3** (2.40 mg, 0.0075 mmol) was added and heated at 70 °C for 12h, but there was no visible change.

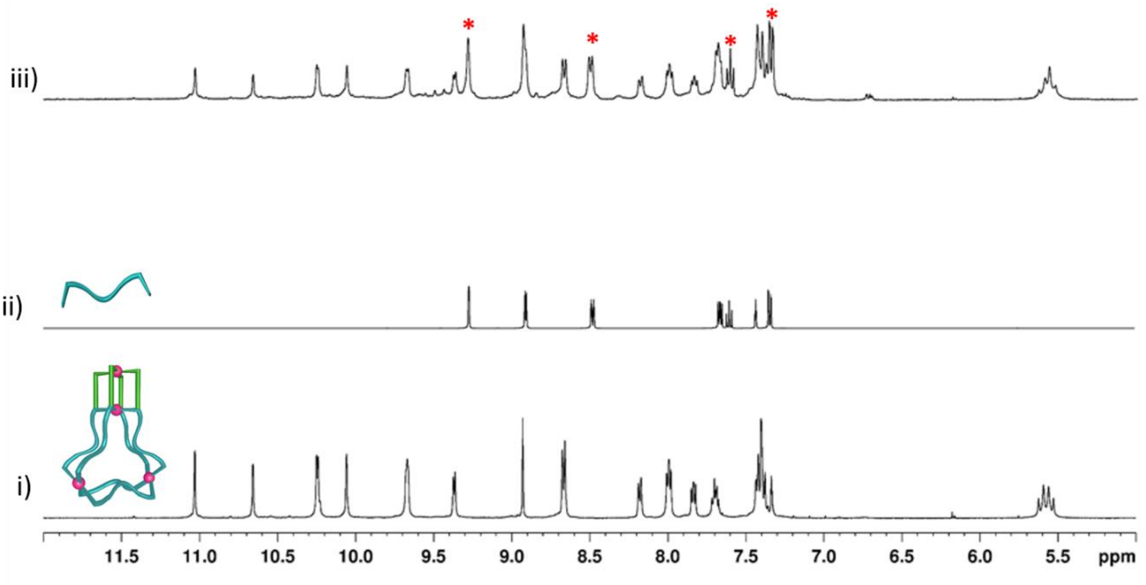

Supplementary Figure 121. Partial ^{1}H NMR spectra (400 MHz, DMSO-d_6, 300 K) of: (i) $[NO_3\subset Pd_4($**L3**$)_2($**L5**$)_4](NO_3)_7$, **4a**; (ii) **L3**; (iii) after mixing **L3** with **4a** and heating at 70 °C for 12 h, there was no change, (* represents free ligand **L3**).

Interaction of L5 with complex 4a: No changes

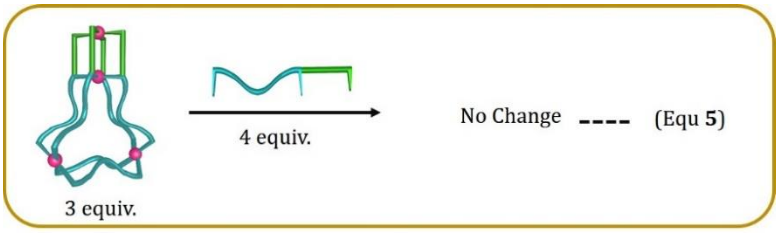

Mixture of ligands **L5** (2.28 mg, 0.005 mmol) and **L3** (0.80 mg, 0.0025 mmol) was dissolved in 0.5 mL of DMSO-d_6, to which $Pd(NO_3)_2$ (1.15 mg, 0.005 mmol) was added and stirred at 70 °C for 1 h to obtain the complex $[(NO_3)\subset Pd_4$(**L3**)$_2$(**L5**)$_4](NO_3)_7$, **4a**. To the formed complex **4a** in DMSO-d_6, ligand **L5** (0.75 mg, 0.0016 mmol) was added and heated at 70 °C for 12h, but there was no visible change.

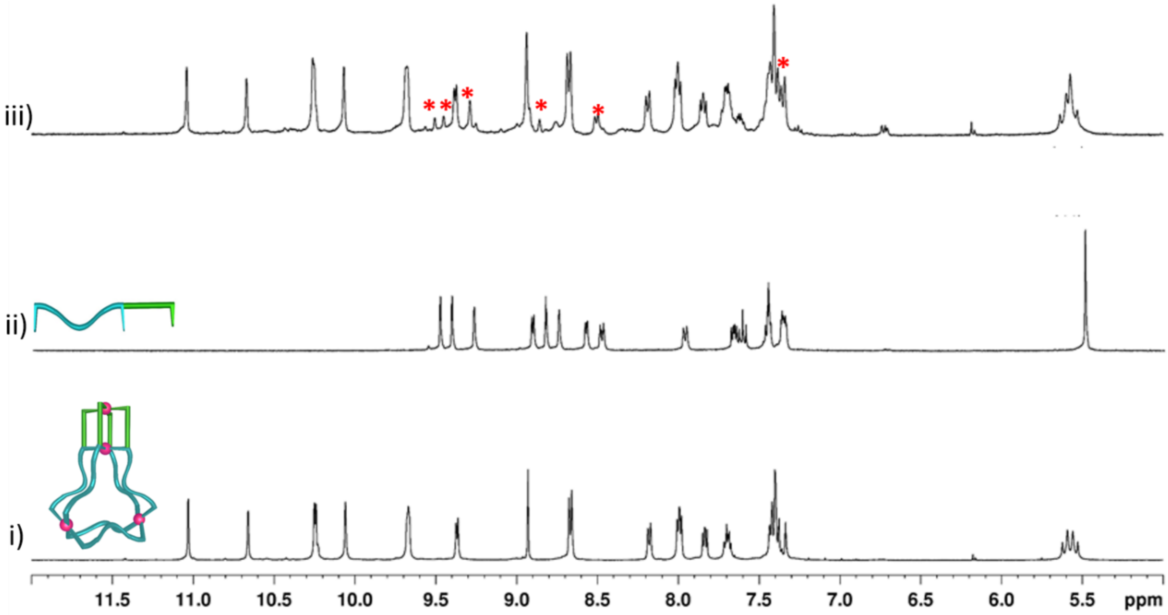

Supplementary Figure 122. Partial ^{1}H NMR spectra (400 MHz, DMSO-d_6, 300 K, TMS as external standard) of: (i) $[NO_3\subset Pd_4$(**L3**)$_2$(**L5**)$_4](NO_3)_7$, **4a**; (ii) **L5**; (iii) after mixing **L5** with **4a** and heating at 70 °C for 12 h, there was no change (* represents free ligand **L3** and **L5**).

Interaction of L6 with complex 4a: Feasible to form 6a, L3 and L5

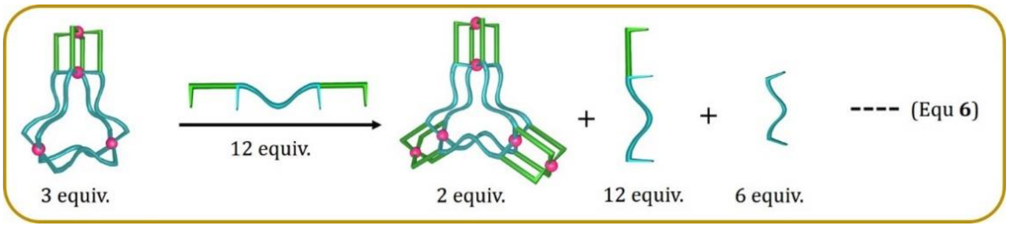

Mixture of ligands **L5** (2.28 mg, 0.005 mmol) and **L3** (0.80 mg, 0.0025 mmol) was dissolved in 0.5 mL of DMSO-d_6, to which $Pd(NO_3)_2$ (1.15 mg, 0.005 mmol) was added and stirred at 70 °C for 1 h to obtain the complex $[(NO_3)\subset Pd_4(\mathbf{L3})_2(\mathbf{L5})_4](NO_3)_7$, **4a**. To the formed complex **4a** in DMSO-d_6, ligand **L6** (2.95 mg, 0.005 mmol) was added and heated at 70 °C for 1h, which led to the formation of complex $[(NO_3)_3\subset Pd_6(\mathbf{L6})_6](NO_3)_9$, **6a** with the release of free ligands **L3** and **L5**.

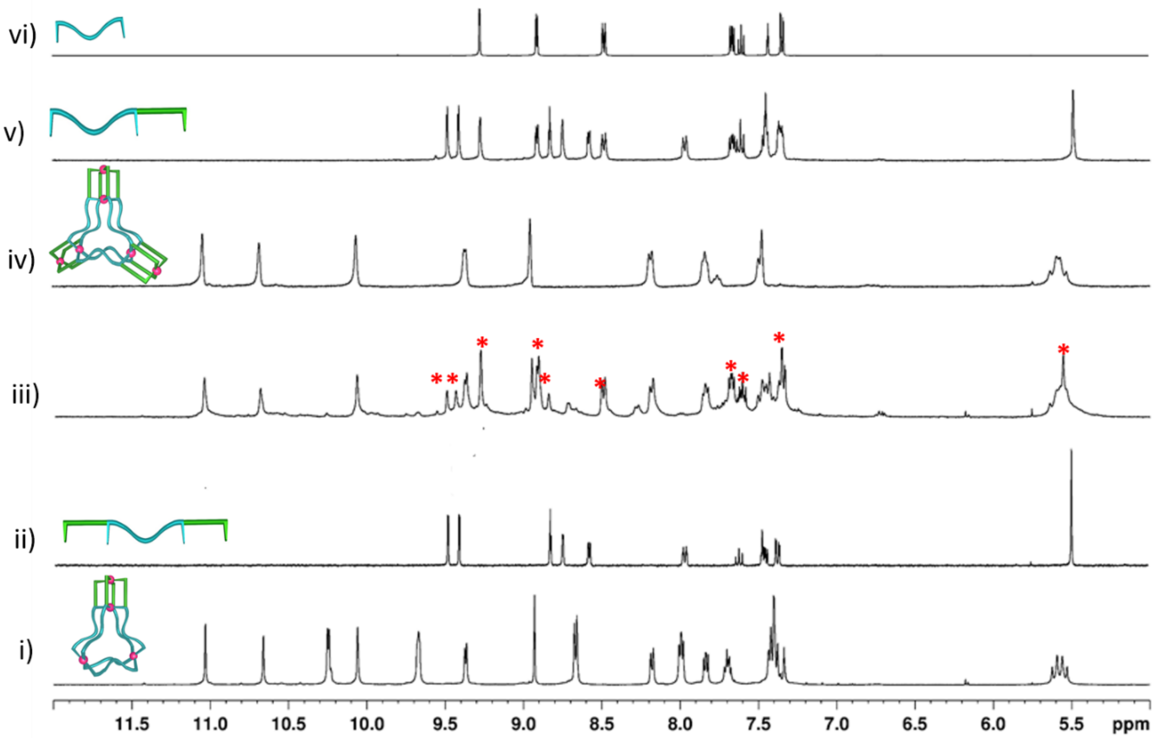

Supplementary Figure 123. Partial ^{1}H NMR spectra (400 MHz, DMSO-d_6, 300 K) of: (i) $[NO_3\subset Pd_4(\mathbf{L3})_2(\mathbf{L5})_4](NO_3)_7$, **4a**; (ii) **L6**; (iii) after mixing **L6** with **4a** and heating at 70 °C for 1 h, $[(NO_3)_3\subset Pd_6(\mathbf{L6})_6](NO_3)_9$, **6a**; was obtained along with free ligand **L3** and **L5** (* represents free ligand **L3** and **L5**) (iv) isolated $[(NO_3)_3\subset Pd_6(\mathbf{L6})_6](NO_3)_9$, **6a** (v) **L5** and (vi) **L3**.

Interaction of L3 with complex 5a: No changes

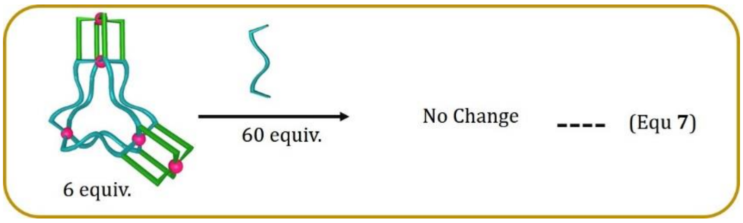

Mixture of ligands **L5** (1.82 mg, 0.004 mmol) and **L6** (1.18 mg, 0.002 mmol) was dissolved in 0.5 mL of DMSO-d_6, to which $Pd(NO_3)_2$ (1.15 mg, 0.005 mmol) was added and stirred at 70 °C for 1 h to obtain the complex $[(NO_3)_2\subset Pd_5($**L5**$)_4($**L6**$)_2](NO_3)_8$, **5a**. To the formed complex **5a** in DMSO-d_6, ligand **L3** (3.20 mg, 0.010 mmol) was added and heated at 70 °C for 12h. There was no visible change and the pentanuclear complex **5a** remained intact.

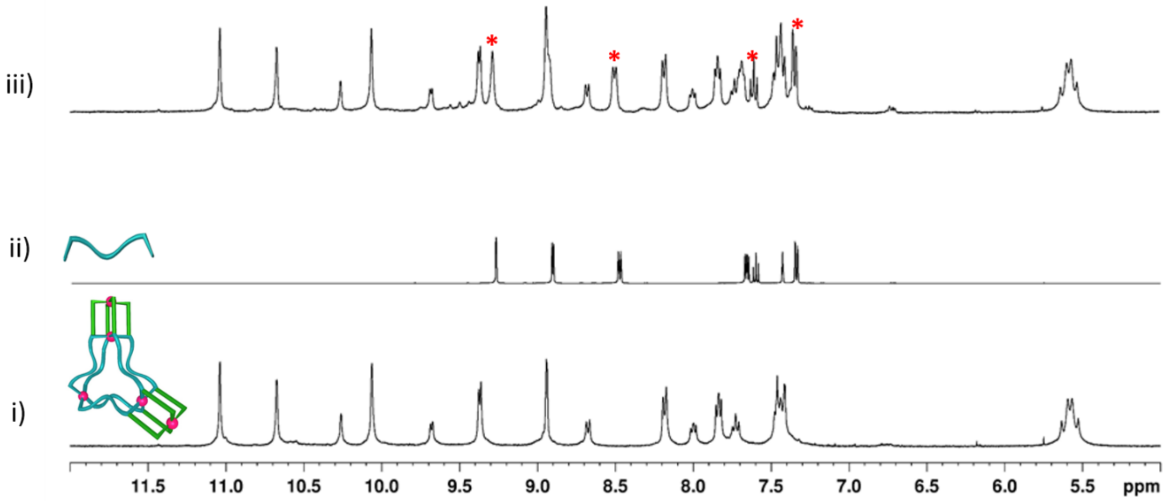

Supplementary Figure 124. Partial ^{1}H NMR spectra (400 MHz, DMSO-d_6, 300 K) of: (i) $[(NO_3)_2\subset Pd_5($**L5**$)_4($**L6**$)_2](NO_3)_8$, **5a**; (ii) **L3**; (iii) after mixing **L3** with complex **5a** and heating at 70 °C for 12 h, complex **5a** remained intact along with free ligand **L3** (* represents free ligand **L3**).

Interaction of L5 with complex 5a: No changes

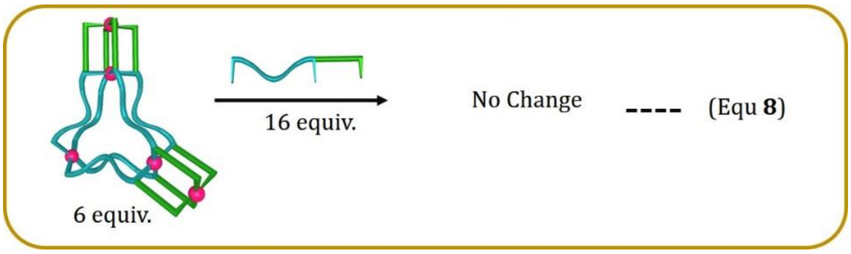

Mixture of ligands **L5** (1.82 mg, 0.004 mmol) and **L6** (1.18 mg, 0.002 mmol) was dissolved in 0.5 mL of DMSO-d_6, which Pd(NO_3)$_2$ (1.15 mg, 0.005 mmol) was added and stirred at 70 °C for 1 h to obtain the complex [(NO_3)$_2$⊂Pd_5(**L5**)$_4$(**L6**)$_2$](NO_3)$_8$, **5a**. To the formed complex **5a** in DMSO-d_6, ligand **L5** (1.21 mg, 0.0026 mmol) was added and heated at 70 °C for 12h. There was no visible change and the penta nuclear complex **5a** remained intact.

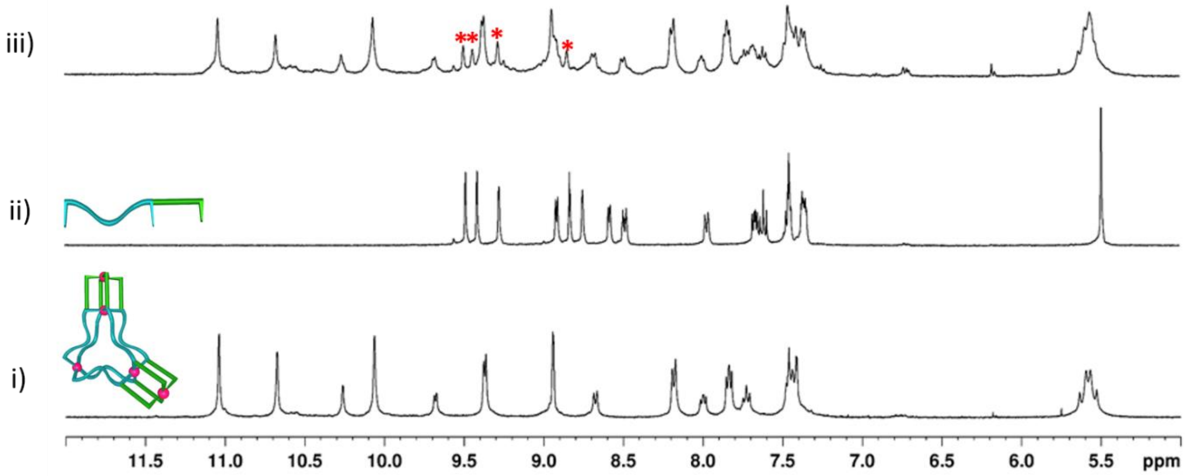

Supplementary Figure 125. Partial ^{1}H NMR spectra (400 MHz, DMSO-d_6, 300 K) of: (i) [(NO_3)$_2$⊂Pd_5(**L5**)$_4$(**L6**)$_2$](NO_3)$_8$, **5a**; (ii) **L5**; (iii) after mixing **L5** with**5a** and heating at 70 °C for 12 h, complex **5a** remained intact along with free ligand **L5** (* represents free ligand **L5**).

Interaction of L6 with complex 5a: Feasible to form 6a and L5

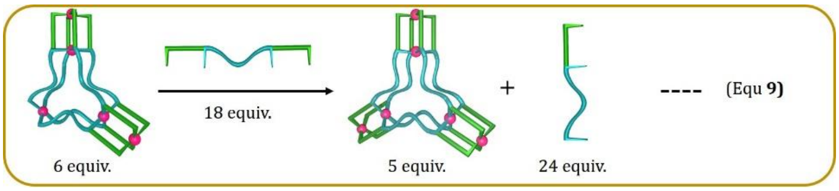

Mixture of ligands **L5** (1.82 mg, 0.004 mmol) and **L6** (1.18 mg, 0.002 mmol) was dissolved in 0.5 mL of DMSO-d_6, which $Pd(NO_3)_2$ (1.15 mg, 0.005 mmol) was added and stirred at 70 °C for 1 h to obtain the complex $[(NO_3)_2\subset Pd_5(\mathbf{L5})_4(\mathbf{L6})_2](NO_3)_8$, **5a**. To the formed complex **5a** in DMSO-d_6, ligand **L6** (1.77 mg, 0.003 mmol) was added and heated at 70 °C for 1h, which led to the formation of complex **6a** and free ligand **L5** remained in solution.

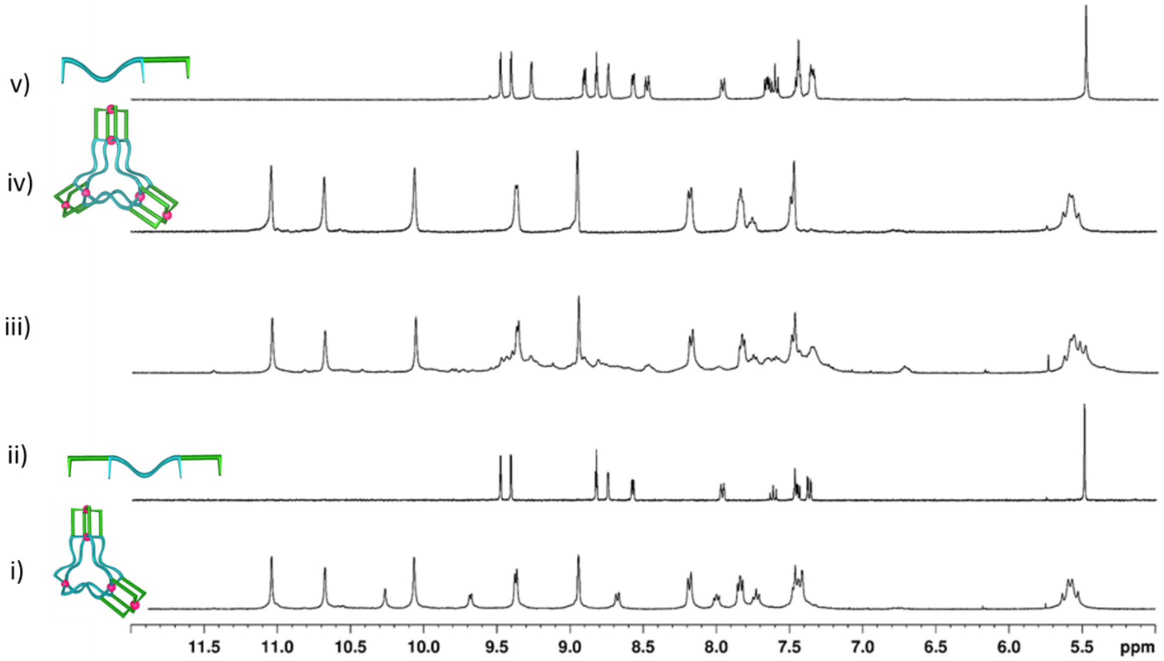

Supplementary Figure 126. Partial ^{1}H NMR spectra (400 MHz, DMSO-d_6, 300 K) of: (i) $[(NO_3)_2\subset Pd_5(\mathbf{L5})_4(\mathbf{L6})_2](NO_3)_8$, **5a**; (ii) **L6**; (iii) after mixing **L6** with **5a** and heating at 70 °C for 1 h, complex $[(NO_3)_3\subset Pd_6(\mathbf{L6})_6](NO_3)_9$, **6a** was obtained and ligand **L5** remained in solution (iv) isolated **6a** and (v) **L5**. (The ligand peaks are not well distinguishable.)

Interaction of L3 with complex 6a: No changes

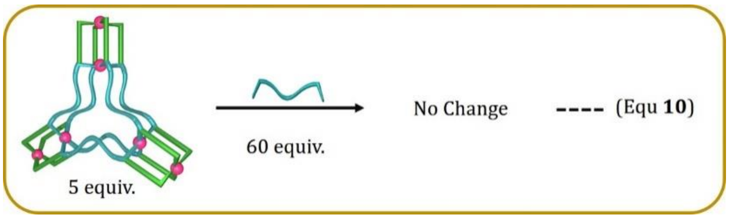

To a solution of ligand **L6** (2.95 mg, 0.005 mmol, 6 equiv.) in 0.5 mL of DMSO-d_6, $Pd(NO_3)_2$ (1.15 mg, 0.005 mmol, 6 equiv.) was added and stirred at 70 °C for 20 min to obtain the complex $[(NO_3)_3\subset Pd_6($**L6**$)_6](NO_3)_9$, **6a**. To the formed complex **6a** in DMSO-d_6, ligand **L3** (3.20 mg, 0.010 mmol) was added and heated at 70 °C for 12h, it showed no change, the formed complex **6a** remained intact.

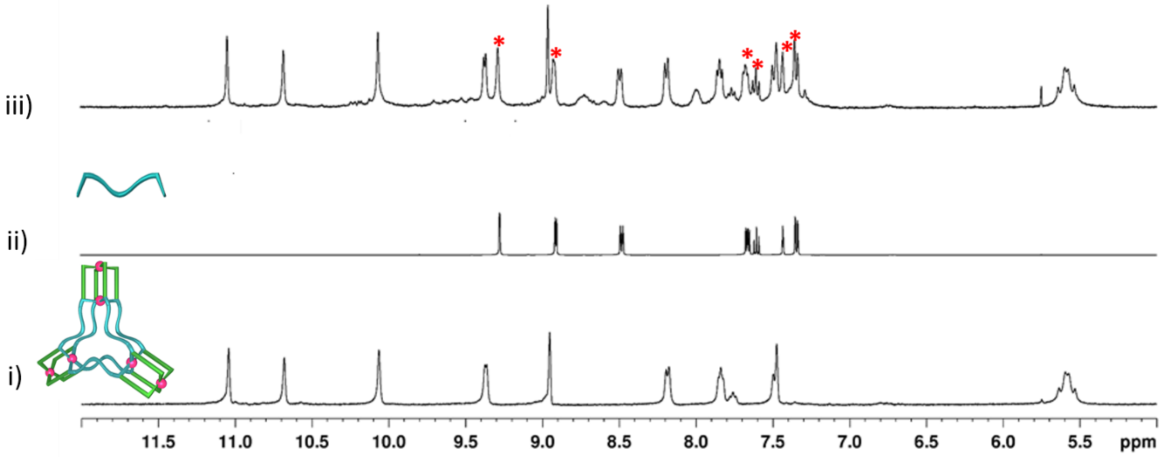

Supplementary Figure 127. Partial ^{1}H NMR spectra (400 MHz, DMSO-d_6, 300 K) of: (i) $[(NO_3)_3\subset Pd_6($**L6**$)_6](NO_3)_9$, **6a**; (ii) **L3**; (iii) after mixing **L3** with **6a** and heating at 70 °C for 12 h, there was no change in complex $[(NO_3)_3\subset Pd_6($**L6**$)_6](NO_3)_9$, **6a** and ligand **L3** remained in solution. (* represents free ligand **L3**).

Interaction of L5 with complex 6a: No changes

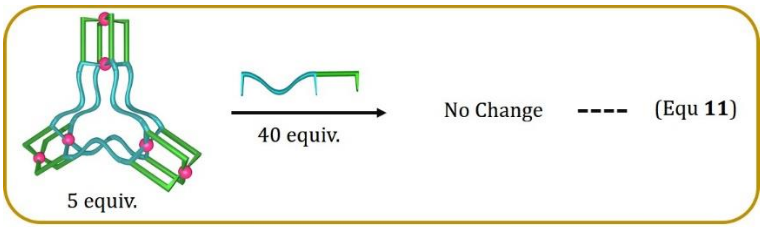

To a solution of ligand **L6** (2.95 mg, 0.005 mmol, 6 equiv.) dissolved in 0.5 mL of DMSO-d_6, $Pd(NO_3)_2$ (1.15 mg, 0.005 mmol, 6 equiv.) was added and stirred at 70 °C for 20 mins to obtain the complex $[(NO_3)_3{\subset}Pd_6($**L6**$)_6](NO_3)_9$, **6a**. To the formed complex **6a** in DMSO-d_6, ligand **L5** (3.04 mg, 0.0067 mmol) was added and heated at 70 °C for 12h, it showed no change and the formed complex **6a** remained intact.

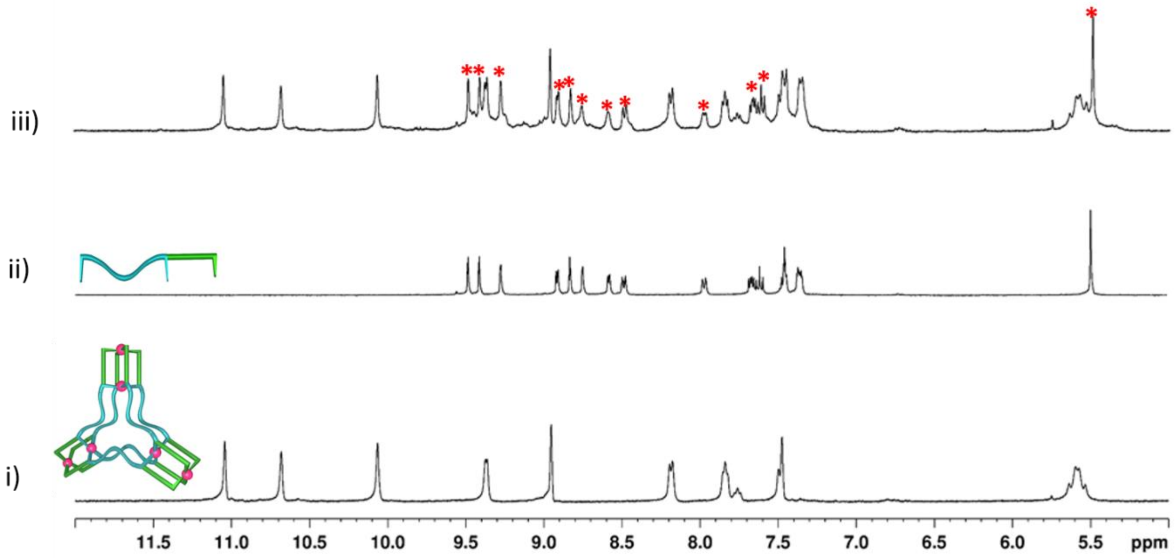

Supplementary Figure 128. Partial ^{1}H NMR spectra (400 MHz, DMSO-d_6, 300 K) of: (i) $[(NO_3)_3{\subset}Pd_6($**L6**$)_6](NO_3)_9$, **6a**; (ii) **L5**; (iii) after mixing **L5** with **6a** and heating at 70 °C for 12 h, there was no change in complex $[(NO_3)_3{\subset}Pd_6($**L6**$)_6](NO_3)_9$, **6a** and ligand **L5** remained in solution. (* represents free ligand **L5**).

Interaction of L3 with complex 4e: Feasible to form 4a and L3

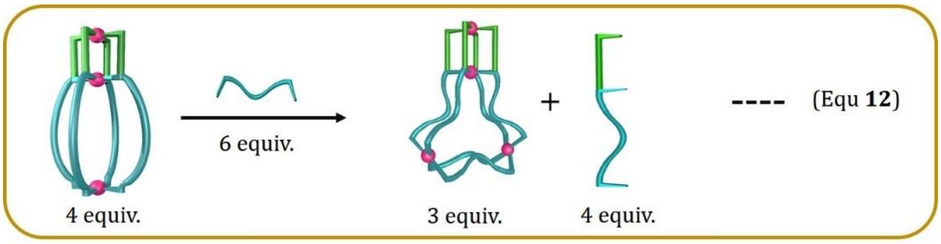

To the solution of ligand **L5** (3.20 mg, 0.010 mmol) in 0.5 mL of DMSO-d_6, Pd(NO_3)$_2$ (1.15 mg, 0.005 mmol) was added and stirred at 70 °C for 1 h to obtain the trinuclear complex [NO_3⊂Pd_3(**L5**)$_4$](NO_3)$_5$, **4e**. To the formed trinuclear complex in DMSO-d_6 ligand **L3** (2.95 mg, 0.005 mmol) was added and heated at 70 °C for 1h, which led to the formation of tetranuclear complex [(NO_3)⊂Pd_4(**L3**)$_2$(**L5**)$_4$](NO_3)$_7$, **4a**.

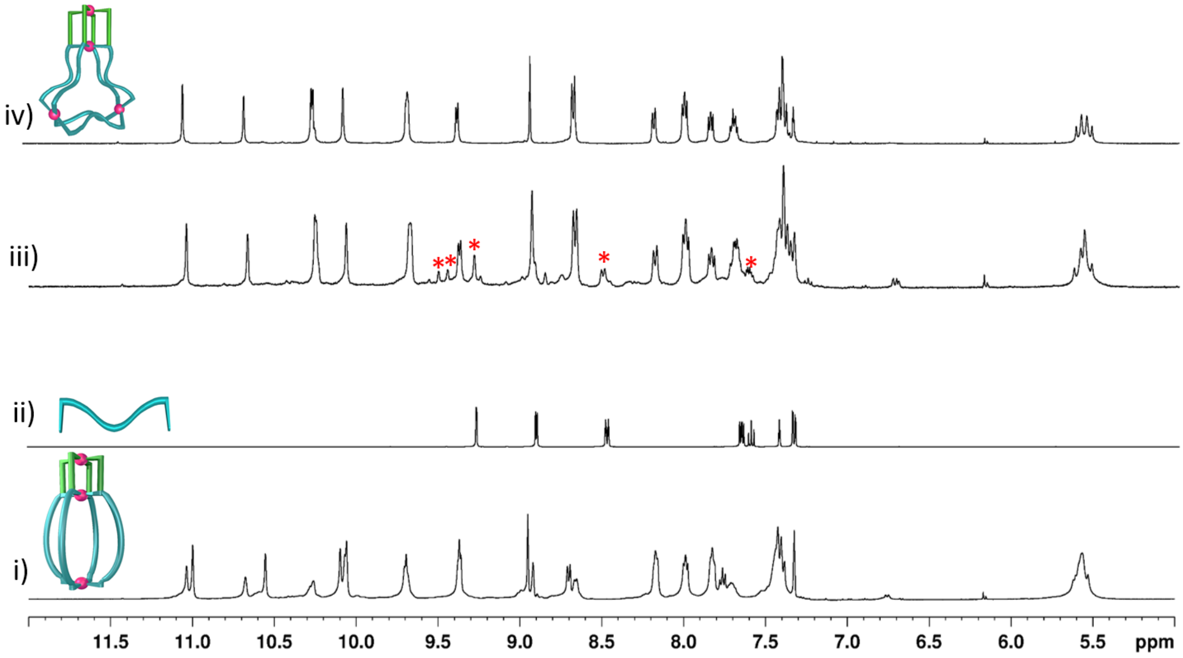

Supplementary Figure 129. Partial ^{1}H NMR spectra (400 MHz, DMSO-d_6, 300 K) of: (i) proposed complex [(NO_3)⊂Pd_3(**L5**)$_4$](NO_3)$_5$, **4e**; (ii) **L3**; (iii) after mixing **L3** to complex **4e** and heating at 70 °C for 1 h, complex [NO_3⊂Pd_4(**L3**)$_2$(**L5**)$_4$](NO_3)$_7$, **4a** was formed (* represents free ligand **L5**) and (iv) isolated [NO_3⊂Pd_4(**L3**)$_2$(**L5**)$_4$](NO_3)$_7$, **4a**.

Interaction of L6 with complex 4e: Feasible to form 6a and L5

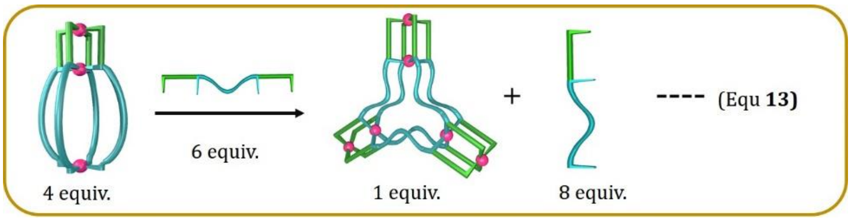

To the solution of ligand **L5** (3.20 mg, 0.010 mmol) in 0.5 mL of DMSO-d_6, $Pd(NO_3)_2$ (1.15 mg, 0.005 mmol) was added and stirred at 70 °C for 1 h to obtain the trinuclear complex $[NO_3 \subset Pd_3(\mathbf{L5})_4](NO_3)_5$, **4e**. To the formed trinuclear complex in DMSO-d_6 ligand **L6** (2.95 mg, 0.005 mmol) was added and heated at 70 °C for 1h, which led to the formation of hexanuclear complex $[(NO_3)_3 \subset Pd_6(\mathbf{L6})_6](NO_3)_9$, **6a** with the release of free ligand **L5**.

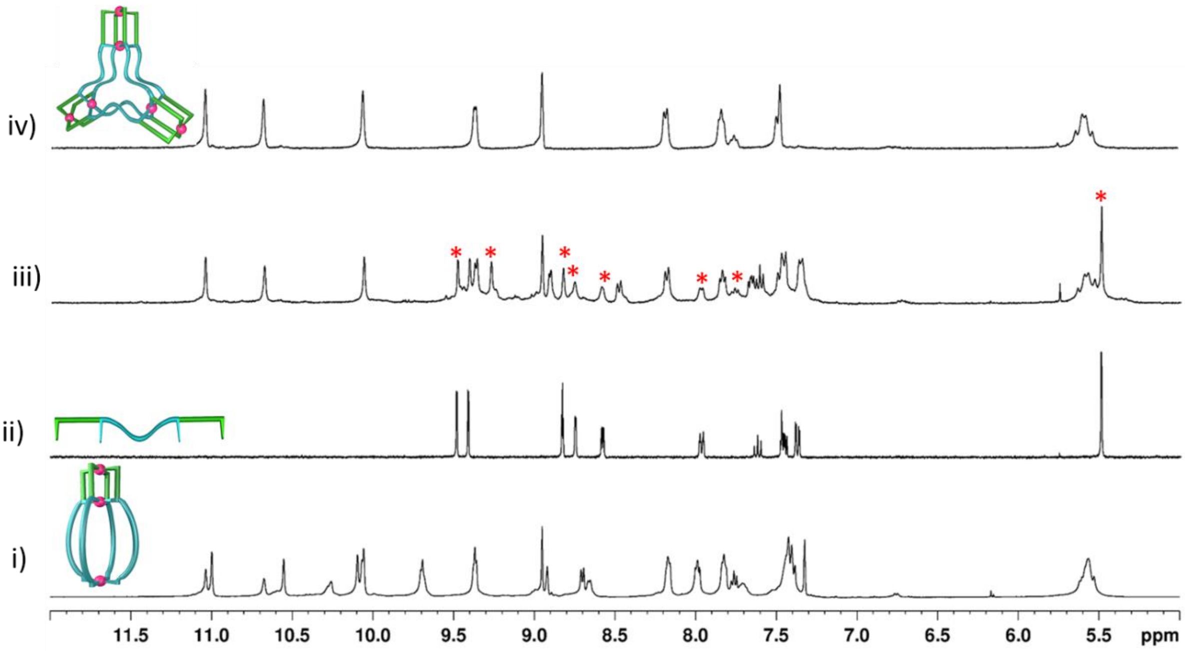

Supplementary Figure 130. Partial ^{1}H NMR spectra (400 MHz, DMSO-d_6, 300 K) of: (i) proposed complex $[(NO_3) \subset Pd_3(\mathbf{L5})_4](NO_3)_5$, **4e**; (ii) **L6**; (iii) after mixing **L6** with complex **3a*** and heating at 70 °C for 30 min, hexanuclear complex $[(NO_3)_3 \subset Pd_6(\mathbf{L6})_6](NO_3)_9$, **6a** was formed (* represents free ligand **L5**) and (iv) isolated $[(NO_3)_3 \subset Pd_6(\mathbf{L6})_6](NO_3)_9$, **6a**.

Interaction of L5 and L6 with complex 3a: Feasible to form 5a and L3

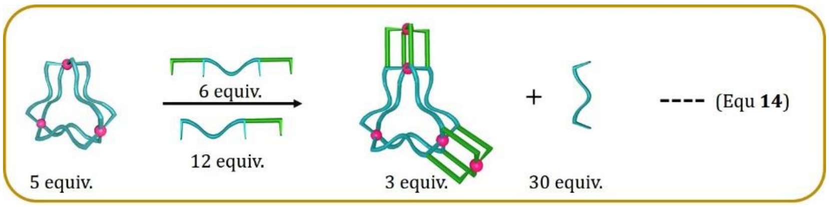

To the solution of ligand **L3** (3.20 mg, 0.010 mmol) in 0.5 mL of DMSO-d_6, Pd$(NO_3)_2$ (1.15 mg, 0.005 mmol) was added and stirred at room temperature for 10 mins to obtain the trinuclear complex [Pd$_3$(**L3**)$_6$]$(NO_3)_6$, **3a**. To the formed trinuclear complex in DMSO-d_6, **L5** (1.82 mg, 0.0038 mmol) and **L6** (1.18 mg, 0.0019 mmol) were added and heated at 70 °C for 1h, which led to the formation of [$(NO_3)_2$⊂Pd$_5$(**L5**)$_4$(**L6**)$_2$]$(NO_3)_8$, **5a** with the release of free ligand **L3**.

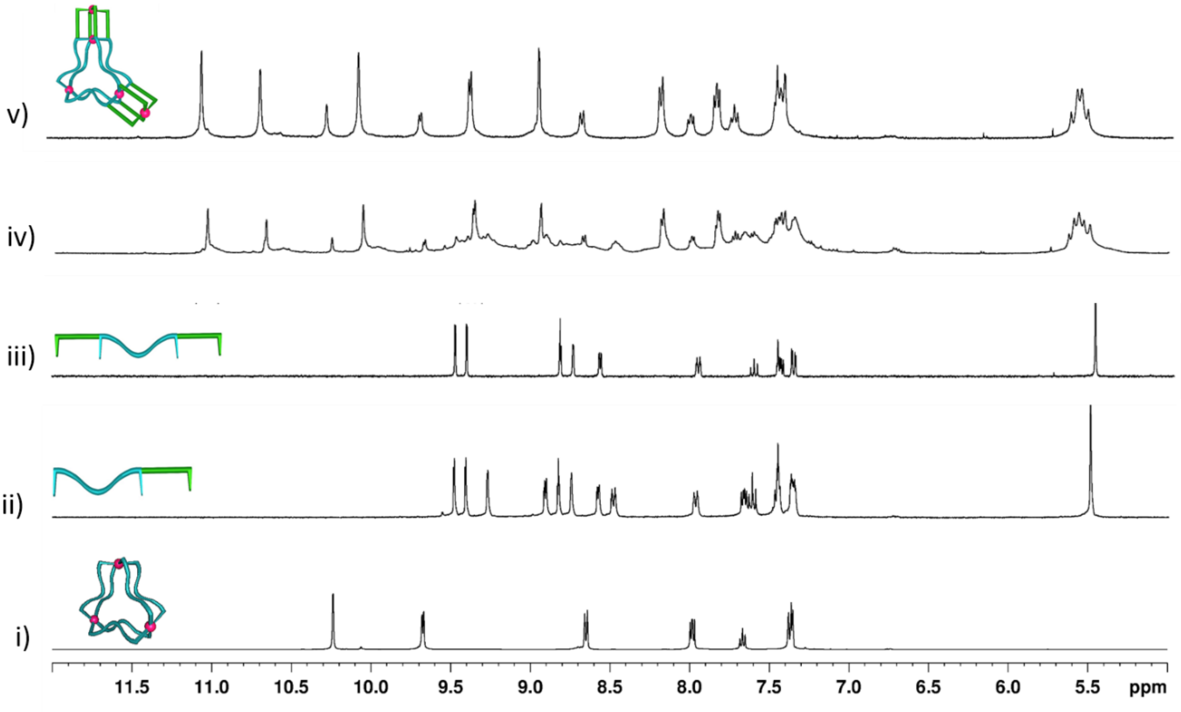

Supplementary Figure 131. Partial ^{1}H NMR spectra (400 MHz, DMSO-d_6, 300 K) of: (i) [Pd$_3$(**L3**)$_6$]$(NO_3)_6$, **3a**; (ii) **L5**; (iii) **L6**; (iv) after mixing **L5** and **L6** with **3a** and heating at 70 °C for 1 h, it gave [$(NO_3)_2$⊂Pd$_5$(**L5**)$_4$(**L6**)$_2$]$(NO_3)_8$, **5a** was formed along with free ligand **L3** which appeared as broad signal and (v) [$(NO_3)_2$⊂Pd$_5$(**L5**)$_4$(**L6**)$_2$]$(NO_3)_8$, **5a**

PM6 optimised structures of some of the complexes

The metallosupramolecular structures were supported by geometry optimizations on the semiempirical PM6 level of theory by using the software Gaussian 09.[2]

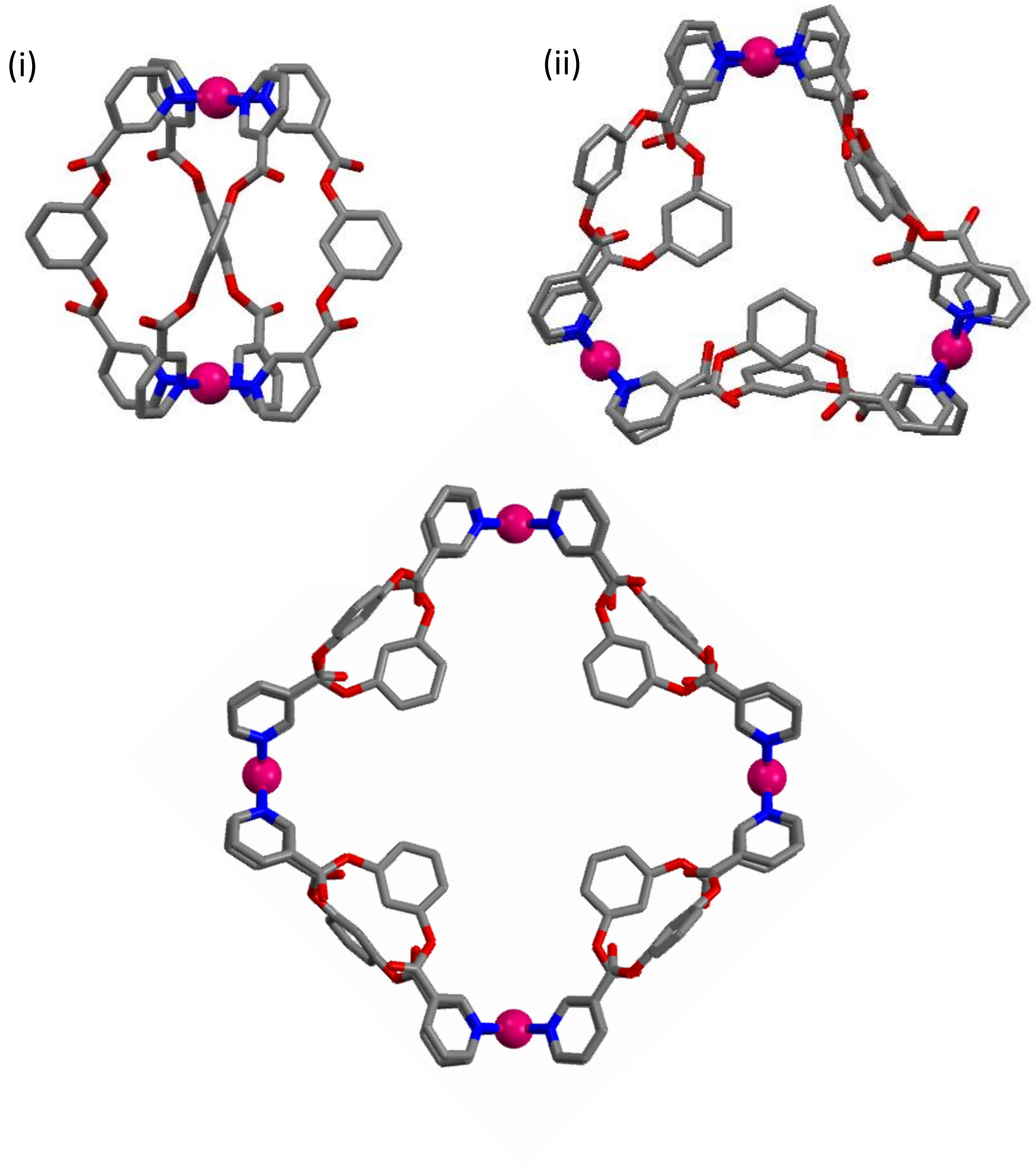

Supplementary Figure 132. PM6 optimised structures for the cationic part of the complexes **3g**, **3a** and **3h:** (i) $[Pd_2(\mathbf{L3})_4]^{4+}$, (ii) $[Pd_3(\mathbf{L3})_6]^{6+}$ and (iii) $[Pd_4(\mathbf{L3})_8]^{8+}$ (hydrogen atoms are not shown for clarity). Where red color represents oxygen atoms, blue color represents the nitrogen atoms, grey color represents the carbon atoms and cyan color represents the palladium atoms.

(i) (ii)

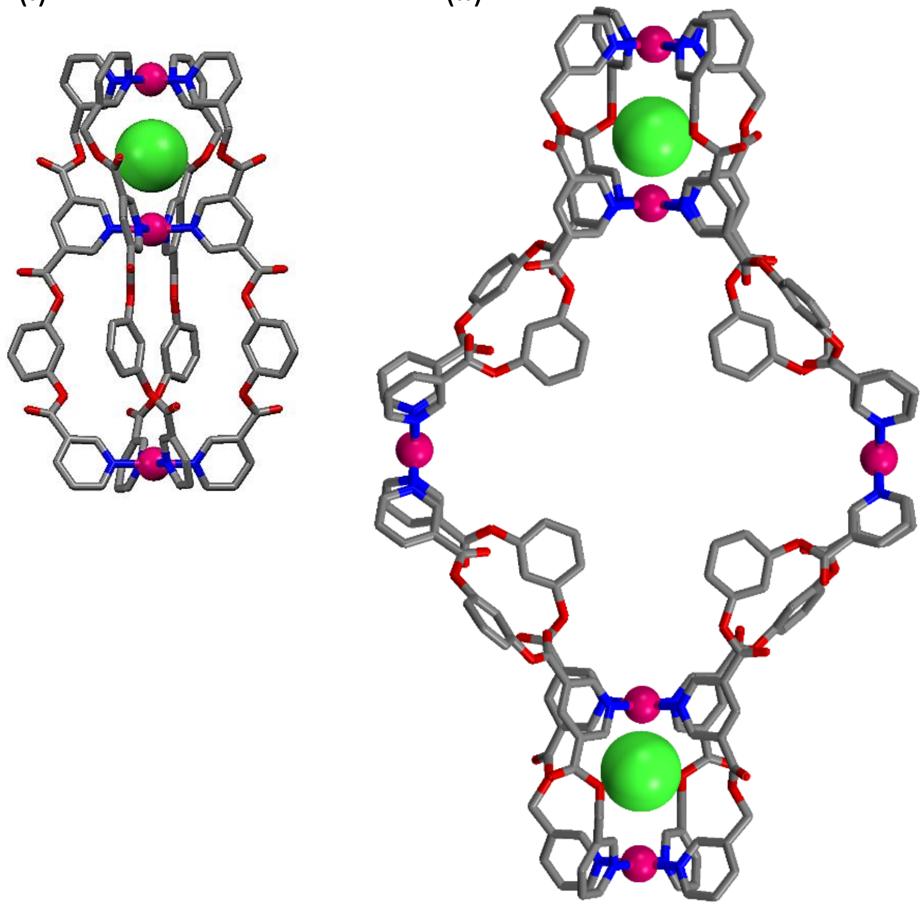

Supplementary Figure 133.. PM6 optimised structures for the cationic part of the complexes **4g** and **4h**: (i) [Cl⊂Pd_3(**L5**)$_4$]$^{5+}$ and (ii) [$(Cl)_2$⊂Pd_6(**L5**)$_8$]$^{10+}$ (hydrogen atoms are not shown for clarity). Where red color represents oxygen atoms, blue color represents the nitrogen atoms, grey color represents the carbon atoms, green color represents the chloride atom/atoms and cyan color represents the palladium atoms.

Single crystal X-ray diffraction study

Single-crystal X-ray diffraction data were recorded for all the crystals on a Bruker AXS Kappa Apex III CMOS dual source diffractometer using Cu-Kα (λ = 1.54178 Å) radiation for **3a** and **4acII** and Mo-Kα (λ=0.71073 Å) radiation for the other crystals. The crystals were mounted on the goniometer head using a fiber loop and optically centered. The automatic cell determination routine was employed to collect reflections (36 frames at three different orientations of the detector in case of Mo source and 40 frames at two different orientations of the detector in case of Cu source) and used for determining the unit cell parameters. Further, intensity data for structure determination were collected through an optimized strategy which gave an average 4-fold redundancy. The program APEX3-SAINT (Bruker, 2016) was used for integrating the frames. Semi-empirical absorption correction (multi-scan) based on symmetry equivalent reflections was applied using the SADABS program (Bruker, 2016). The structures were solved by direct methods and refined by full matrix least squares, based on F^2 using SHELX-2014 software package[3] and the program WinGX.[4] Molecular and packing diagrams were generated using Mercury CSD.[5] Geometrical calculations were performed using PLATON.[6] ORTEPs were prepared using ORTEP-3.[4] The crystallographic data are summarised in Table **S5a** and **S5b**.

Supplementary Table 5. Summary of crystallographic data for crystals of **2c**, **3a** and **4acI**.

	**2c**	**3a**	**4acI**
Chemical Formula	$C_{48}H_{32}ClN_{10}O_{18}Pd_2$	$C_{124}H_{120}N_{17}O_{47}Pd_3S_8$	$C_{158}H_{146}Cl_{0.35}N_{23.65}O_{62.94}Pd_4S_8$
M_r	1285.08	3176.04	4077.68
Temp. (K)	173(2)	273(2)	223(2)
Crystal system	Tetragonal	Monoclinic	Triclinic
Space group	P-42_1m	$P2_1/c$	P-1
Morphology	block	block	Plate
Crystal size (mm)	0.25 × 0.20 × 0.15	0.10 × 0.10 × 0.15	0.10 × 0.10 × 0.15
a (Å)	17.417(2)	18.498(2)	14.760(5)
b (Å)	17.417(2)	19.453(2)	21.882(8)
c (Å)	10.0740(13)	43.806(6)	35.338(13)
α (°)	90	90	73.785(15)
β (°)	90	91.104(6)	83.65(2)
γ (°)	90	90	80.621(14)
V (Å^3)	3056.0(8)	15760(3)	10787(7)
Z<br>D_{calc} (g cm^{-3})	2<br>1.397	4<br>1.339	2<br>1.255
μ(mm^{-1})<br>$F(000)$	0.704<br>1286	4.411<br>6500	0.489<br>4162
T_{min}<br>T_{max}	0.578<br>0.735	0.527<br>0.745	0.633<br>0.745
h, k, l (min,max)	(-20,20), (-20,20), (-11,11)	(-20,19), (-23,23), (-35,53)	(-17,17), (-26,26), (-42,42)
Reflns collected	62496	55963	237450
Unique reflns	2800	26361	38383
Observed reflns<br>R_{int}	2562<br>0.0927	14694<br>0.0880	25843<br>0.0928
No. of parameters	190	1780	2289
GoF	2.112	1.251	1.033
$R_1[I > 2\sigma(I)]$	0.1259	0.1342	0.0972
$WR_2[I > 2\sigma(I)]$	0.3770	0.3451	0.2623
R_1_all data	0.1354	0.1986	0.1369
Wr_2_all data	0.4114	0.3899	0.2964
$\Delta\rho_{max}$, $\Delta\rho_{min}$ (e Å^{-3})	1.95, -0.69	2.90, -1.48	2.54, -2.01
CCDC No.	1941617	1941618	1941619

Supplementary Table 6. Summary of crystallographic data for crystals of **4acII**, **5c** and **6c**.

	**4acII**	**5c**	**6c**
Chemical Formula	$C_{184}H_{176}Cl_{0.24}N_{22.76}O_{61.28}Pd_4S_9$	$C_{180}H_{148}Cl_{10}N_{26}O_{65}Pd_5S_4$	$C_{202}H_{162}Cl_3N_{28}O_{65}Pd_6S_5$
M_r	4409.23	4729.98	4926.64
Temp. (K)	223(2)	220(2)	273(2)
Crystal system	Monoclinic	Triclinic	Monoclinic
Space group	$P2_1$	P-1	$P2_1/m$
Morphology	Block	block	Block
Crystal size (mm)	0.15 × 0.25 × 0.20	0.20 × 0.25 × 0.30	0.15 × 0.20 × 0.25
a (Å)	14.8878(9)	14.297(7)	19.347(3)
b (Å)	32.740(2)	30.58(2)	40.756(6)
c (Å)	24.0875(16)	33.486(16)	21.600(3)
α (°)	90	105.33(2)	90
β (°)	106.428(4)	94.55(2)	112.654(5)
γ (°)	90	100.81(3)	90
V (Å^3)	11261.6(12)	13740(13)	15718(4)
Z<br>D_{calc} (g cm^{-3})	2<br>1.300	2<br>1.143	2<br>1.041
μ(mm^{-1})<br>*F(000)*	4.018<br>4523	0.517<br>4788	0.457<br>4994
T_{min}<br>T_{max}	0.395<br>0.753	0.631<br>0.744	0.612<br>0.719
h, k, l (min,max)	(-17,16), (-39,38), (-28,28)	(-14,14), (-30,30), (-33,33)	(-23,23), (-48,48), (-25,25)
Reflns collected	161480	308669	262999
Unique reflns	39461	29151	28027
Observed reflns<br>R_{int}	31644<br>0.1262	18211<br>0.1394	21478<br>0.1033
No. of parameters	2478	2563	1381
GoF	1.026	1.030	1.022
$R_1[I > 2\sigma(I)]$	0.0807	0.0829	0.0889
$WR_2[I > 2\sigma(I)]$	0.2037	0.2105	0.2384
R_1_all data	0.0996	0.1398	0.1111
Wr_2_all data	0.2216	0.2605	0.2600
$\Delta\rho_{max}$, $\Delta\rho_{min}$ (e Å^{-3})	2.24, -0.99	1.79, -1.21	1.64, -1.83
CCDC No.	1941620	1941621	1941622

Crystal structure of [Cl⊂Pd_2(L2)$_4$](NO_3)$_3$, 2c. X-ray diffraction-quality single crystals of **2c** were obtained by slow diffusion of 1,2-dichloroethane and CCl_4 into a solution of the complex in DMSO:CH_3CN (1:1 *v*/*v*). The asymmetric unit in crystals of **2c** (tetragonal, *P*-42_1*m*) contains 1/4th molecule of the binuclear palladium(II) complex. A chloride ion was located inside the cavity of the cage and a nitrate was present outside. The electron density corresponding to other anions and disordered solvent molecules was squeezed. Appropriate DFIX, FLAT and SADI instructions were applied during the least-squares refinement to maintain sensible molecular geometry of the pyridine rings and the nitrate ion. All hydrogen atoms were placed in geometrically idealized positions (C–H = 0.95 Å for pyridine H atoms) and refined isotropically

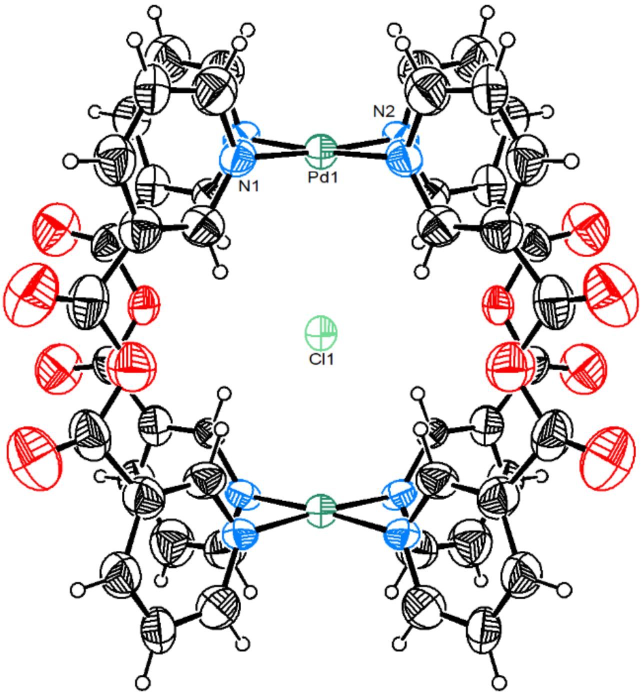

Supplementary Figure 134. ORTEP of the molecule in crystals of **2c**. Thermal ellipsoids are drawn at 30% probability and hydrogen atoms are depicted as small spheres of arbitrary radii. Solvent molecules and anions outside the cavity of the Pd_2L_4 cage have been omitted for clarity.

Crystal structure of $[Pd_3(L3)_6](NO_3)_6$, 3a. X-ray diffraction-quality single crystals of **3a** were obtained by slow diffusion of toluene:acetonitrile (1:1) into a solution of **3a** in DMSO. The asymmetric unit in crystals of **3a** (monoclinic, $P2_1/c$) contains a molecule of the trinuclear palladium(II) complex. Five nitrate anions and eight DMSO molecules were also located. The anions are located outside the cavity, whereas the cavity was occupied by four number of DMSO molecules. The electron density corresponding to other anions and disordered solvent molecules was squeezed. Appropriate DFIX, DANG, FLAT and SADI instructions were applied during the least-squares refinement to maintain sensible molecular geometry. RIGU and SIMU restraints were used to maintain reasonable ADPs for the bonded atoms. All hydrogen atoms were placed in geometrically idealized positions (C–H = 0.93 Å for pyridine and aromatic H atoms and C-H = 0.96 Å for methyl H atoms) and refined isotropically.

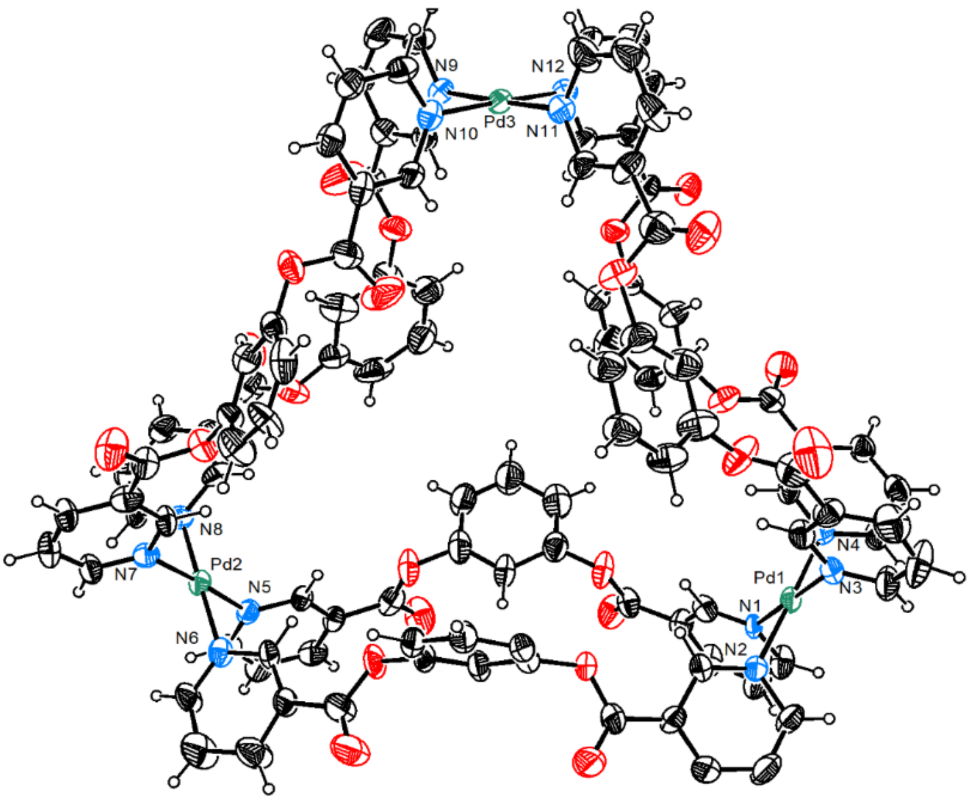

Supplementary Figure 135. ORTEP of the cation in crystals of **3a.** Thermal ellipsoids are shown at 30% probability and hydrogen atoms are depicted as small spheres of arbitrary radii. Solvent molecules and anions have been omitted for clarity.

Crystal structure of $[(Cl)_{0.35}(NO_3)_{0.65}\subset Pd_4(L3)_2(L5)_4](NO_3)_7$, 4acI. To a solution of complex **4a** in DMSO-d_6, AgCl was added and the suspension was stirred for 20 min at room temperature. The AgCl was separated by centrifugation and the supernatant was used for crystallization. X-ray diffraction-quality single crystals of the complex were obtained by slow diffusion of benzene:toluene (1:1 *v*/*v*). into the supernatant liquid. The asymmetric unit in crystals of **4acI** (triclinic, *P*-1) contains a molecule of the tetranuclear palladium(II) complex. A chloride and a nitrate anion which show substitutional disorder were located in the smaller cavity, their occupancy ratio being, 35:65, whereas the bigger trinuclear cavity contains four DMSO molecules. Complex containing exclusively Cl^- was not obtained as the time given for the replacement of nitrate anion from the smaller cavity by chloride anion was not sufficient. Seven nitrate anions were located outside the cavity. Four DMSO molecules and a benzene molecule were also located outside the cavities of the cages. The electron density corresponding to other disordered solvent molecules was squeezed. Appropriate DFIX, DANG, FLAT and SADI instructions were applied during the least-squares refinement to maintain sensible molecular geometry. RIGU and SIMU restraints were used to maintain reasonable ADPs for the bonded atoms. All hydrogen atoms were placed in geometrically idealized positions (C–H = 0.94 Å for pyridine and aromatic H atoms, C–H = 0.98 Å for methylene H atoms and C-H = 0.97 Å for methyl H atoms) and refined isotropically.

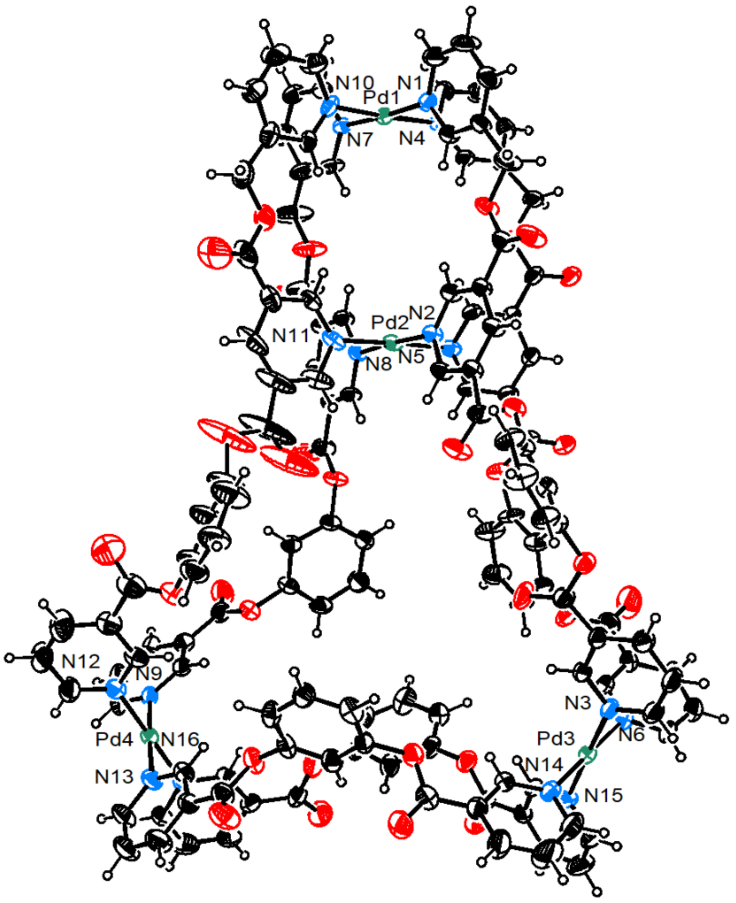

Supplementary Figure 136. ORTEP of the cation in crystals of **4acI.** Thermal ellipsoids are shown at 30% probability and hydrogen atoms are depicted as small spheres of arbitrary radii. Solvent molecules and anions have been omitted for clarity.

Crystal structure of [(Cl)$_{0.25}$(NO$_3$)$_{0.75}$⊂Pd$_4$(L3)$_2$(L5)$_4$](NO$_3$)$_7$, 4acII. To a solution of complex **4a** in DMSO-*d*$_6$, AgCl was added and the suspension was stirred for 20 min at room temperature. The AgCl was separated by centrifugation and the supernatant was used for crystallization. X-ray diffraction-quality single crystals of the complex were obtained by slow diffusion of benzene into the supernatant liquid. Structure solution revealed subtle differences in the structures of **4acI** and **4acII**. The asymmetric unit in crystals of **4acII** (monoclinic, $P2_1$) contains a molecule of the tetranuclear palladium(II) complex. However, the structure was refined as two-component inversion twin with TWIN, BASF instructions (Flack parameter: 0.113(10)). A chloride and a nitrate anion which show substitutional disorder were located in the smaller cavity, their occupancy ratio being, 25:75, whereas the bigger trinuclear cavity contains four DMSO molecules. Complex containing exclusively Cl^- was not obtained as the time given for the replacement of nitrate anion from the smaller cavity by chloride anion was not sufficient. Six nitrate anions were located outside the cavity. Five DMSO molecules and five benzene molecules were also located outside the cavities of the cages. The electron density corresponding to other disordered solvent molecules was squeezed. Appropriate DFIX, DANG, and SADI instructions were applied during the least-squares refinement to maintain sensible molecular geometry. RIGU, SIMU and ISOR restraints were used to maintain reasonable ADPs for the bonded atoms. All hydrogen atoms were placed in geometrically idealized positions (C–H = 0.94 Å for pyridine and aromatic H atoms, C–H = 0.98 Å for methylene H atoms and C-H = 0.97 Å for methyl H atoms) and refined isotropically.

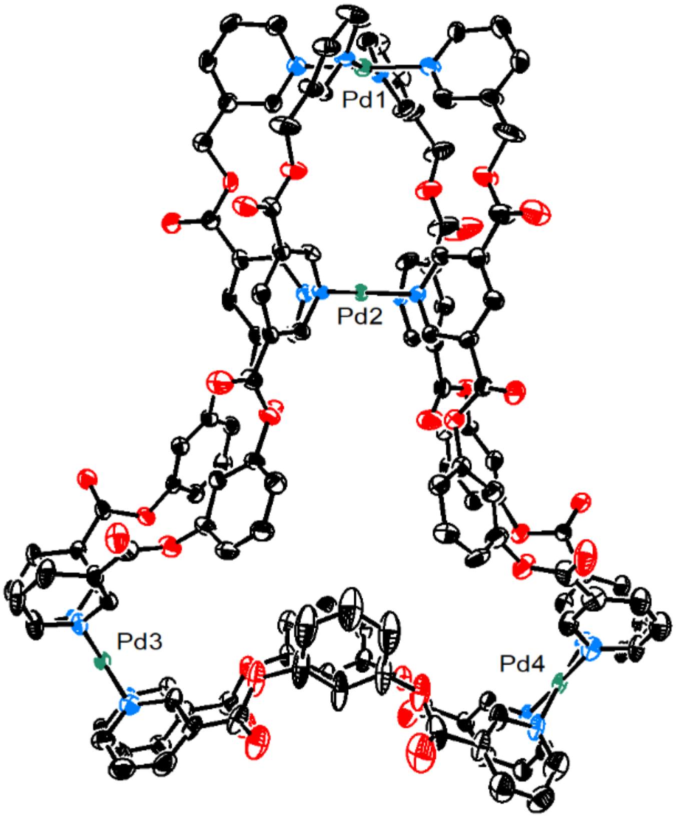

Supplementary Figure 137. ORTEP of the cation in crystals of **4acII.** Thermal ellipsoids are shown at 30% probability. Hydrogen atoms, solvent molecules and anions have been omitted for clarity.

Crystal structure of [(Cl)$_2$⊂Pd$_5$(L5)$_4$(L6)$_2$](NO$_3$)$_8$, 5c. X-ray diffraction-quality single crystals of the complex **5c** were grown by slow diffusion of CCl_4:dioxane (1:1 *v*/*v*) into the solution of the complex **5c** in DMSO. The asymmetric unit in crystals of **5c** (triclinic, *P*-1) contains a molecule of the pentanuclear palladium(II) complex. The two smaller cavities each contain a chloride ion, while the central cavity contains four DMSO molecules. Six nitrate anions were located outside the cavities along with one 1,4-dioxane and two CCl_4 molecules. The electron density corresponding to other anions and disordered solvent molecules was squeezed. Appropriate DFIX, DANG, FLAT and SADI instructions were applied during the least-squares refinement to maintain sensible molecular geometry. RIGU and SIMU restraints were used to maintain reasonable ADPs for the bonded atoms. All hydrogen atoms were placed in geometrically idealized positions (C–H = 0.94 Å for pyridine and aromatic H atoms of the ligand, C–H = 0.98 Å for methylene H atoms and C-H = 0.97 Å for methyl H atoms) and refined isotropically.

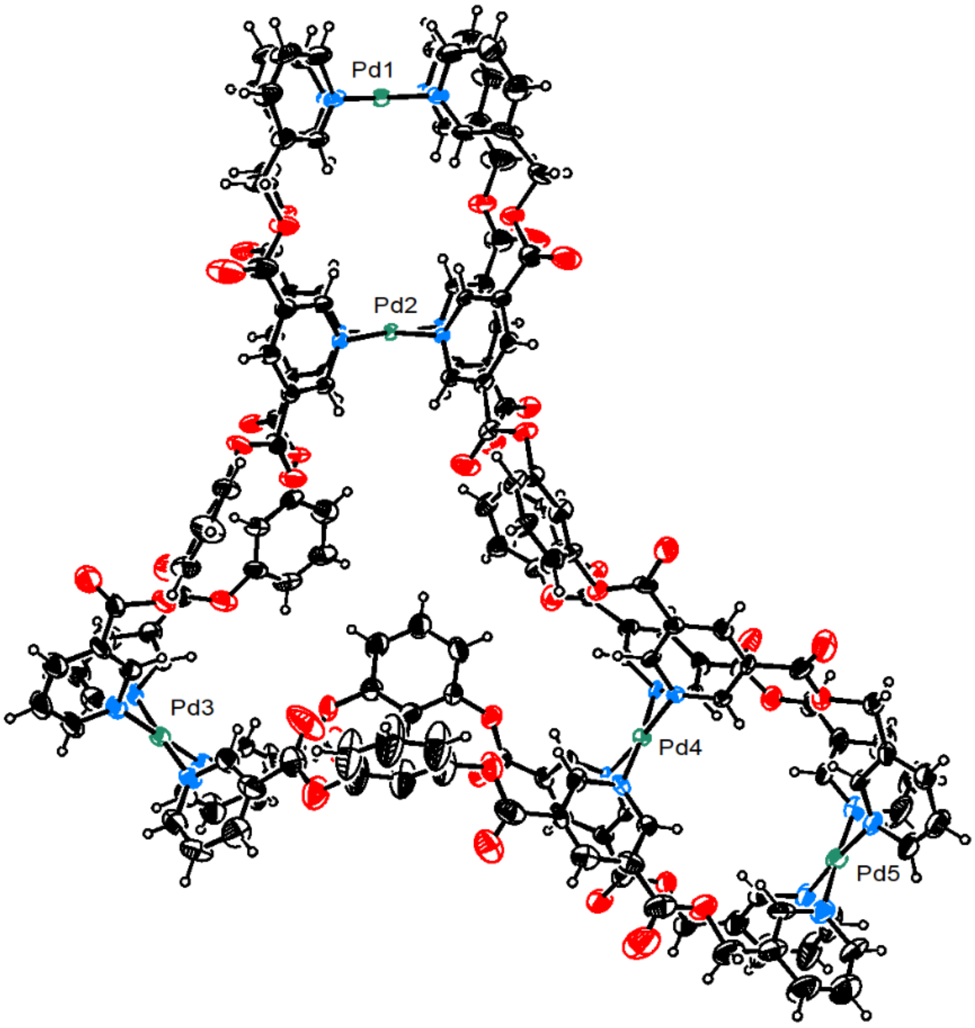

Supplementary Figure 138. ORTEP of the cation in crystals of **5c**. Thermal ellipsoids are shown at 30% probability and hydrogen atoms are depicted as small spheres of arbitrary radii. Solvent molecules and anions have been omitted for clarity.

Crystal structure of [$(Cl)_3 \subset Pd_6(L6)_6](NO_3)_9$, 6c. X-ray diffraction-quality single crystals of the complex **6c** were obtained by slow diffusion of dichloroethane into the solution of the complex **6c** in DMSO. The asymmetric unit in crystals of **6c** (monoclinic, *P*-1) contains half a molecule of the hexanuclear palladium(II) complex. Two chloride (one with full and one with half occupancies) and two nitrate ions were located along with three DMSO molecules. The two palladium atoms with full occupancy and two with half occupancy are seen in the asymmetric unit. The full molecule contains three chloride anions each in the smaller cavity, whereas the central cavity contains three molecules of DMSO. The electron density corresponding to other anions and disordered solvent was squeezed. Appropriate DFIX, DANG, and SADI instructions were applied during the least-squares refinement to maintain sensible molecular geometry. RIGU and SIMU restraints were used to maintain reasonable ADPs for the bonded atoms. All hydrogen atoms were placed in geometrically idealized positions (C–H = 0.93 Å for pyridine and aromatic H atoms, C–H = 0.97 Å for methylene H atoms and C-H = 0.96 Å for methyl H atoms) and refined isotropically.

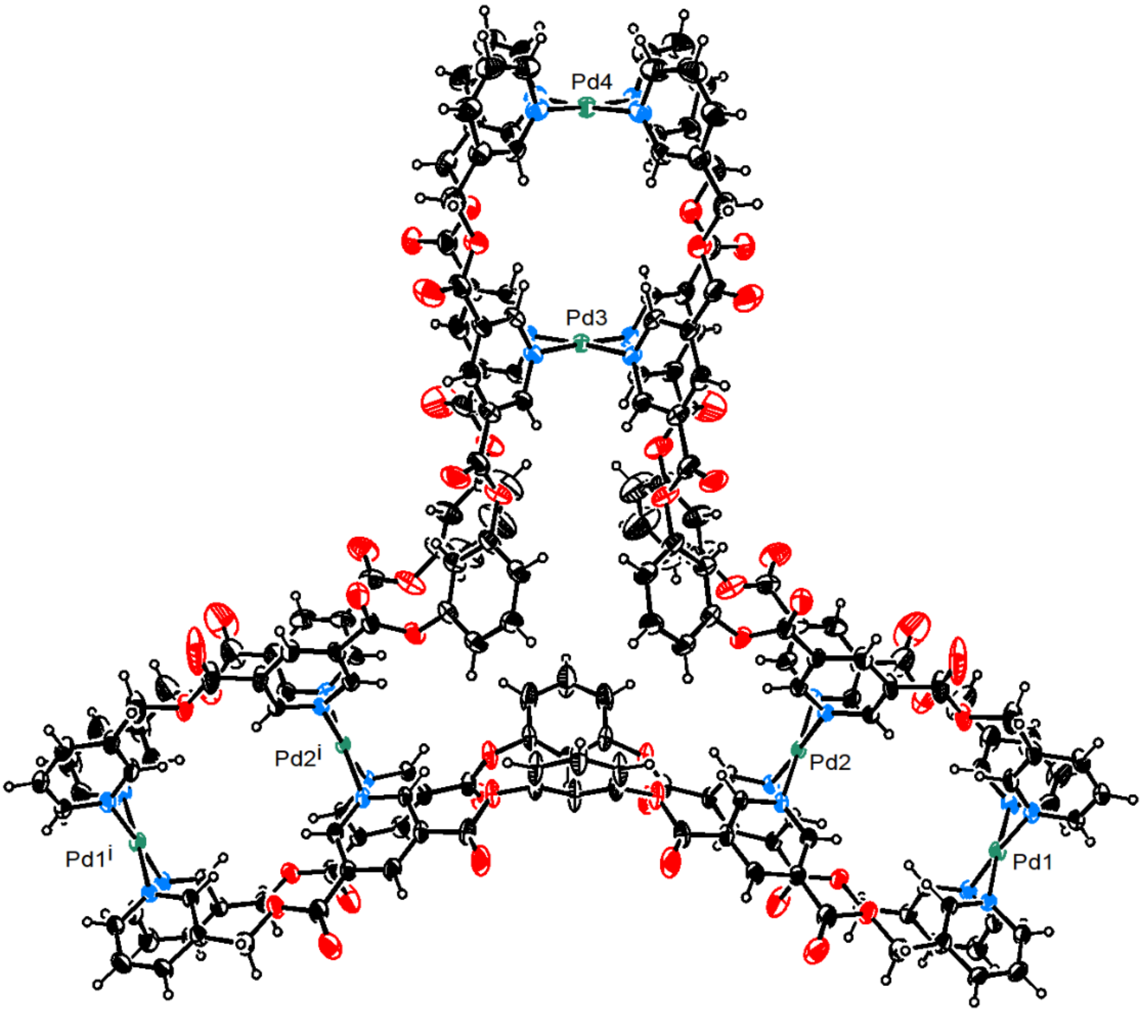

Supplementary Figure 139. ORTEP of the cation in crystals of **6c**. Thermal ellipsoids are shown at 30% probability and hydrogen atoms are depicted as small spheres of arbitrary radii. Solvent molecules and anions have been omitted for clarity.

Crystal structures of 2c, 3a, 4acI, 5c and 6c emphasizing the encapsulated guests.

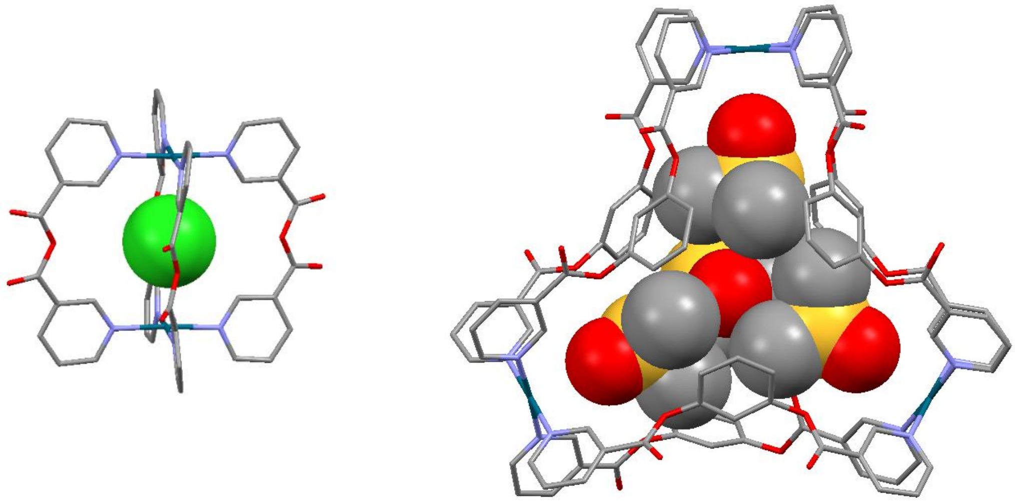

Supplementary Figure 140. Crystal structures showing (a) one chloride as guest in the cationic part of the cage **2c** (b) four DMSO molecules as guests in the cationic part of **3a**, (hydrogen atoms and exohedrally located counter-anions/solvents are excluded for clarity).

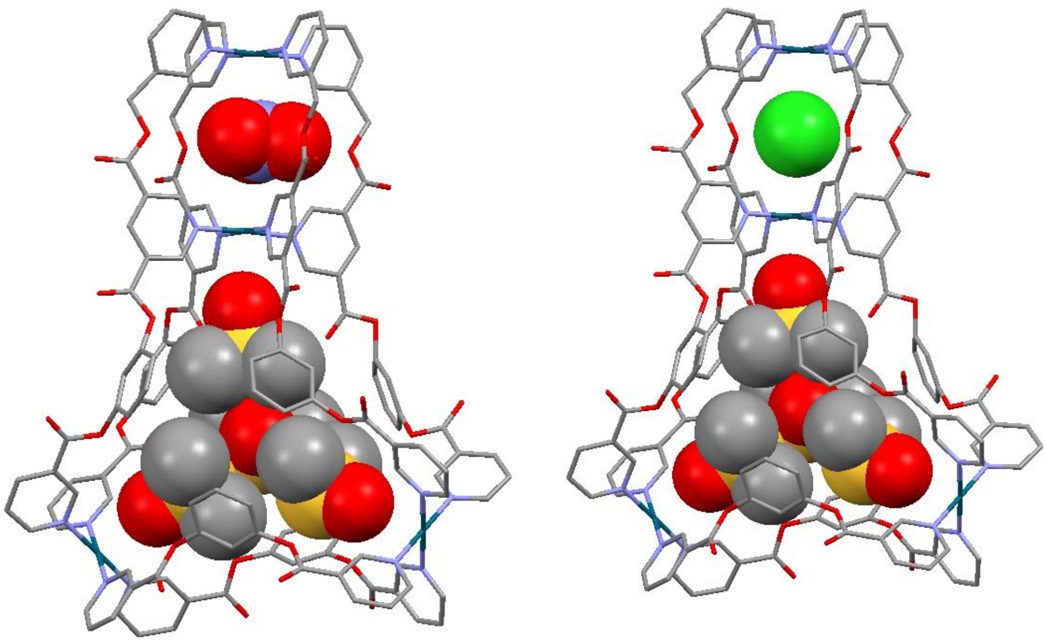

Supplementary Figure 141. Crystal structure showing (a) one nitrate or chloride (occupancies 65:35) as the guest in the smaller cavity and four DMSO molecules in the bigger cavity of the cage **4acI**. (hydrogen atoms and exohedrally located counter-anions/solvents are excluded for clarity).

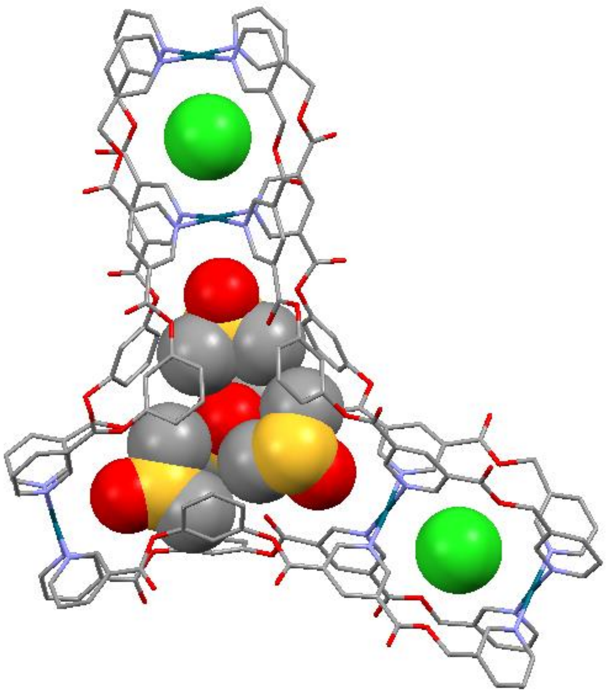

Supplementary Figure 142. Crystal structure showing two chlorides as the guest one each in the two smaller cavities and four DMSO molecules in the bigger cavity of the cage **5c**. (hydrogen atoms and exohedrally located counter-anions/solvents are excluded for clarity).

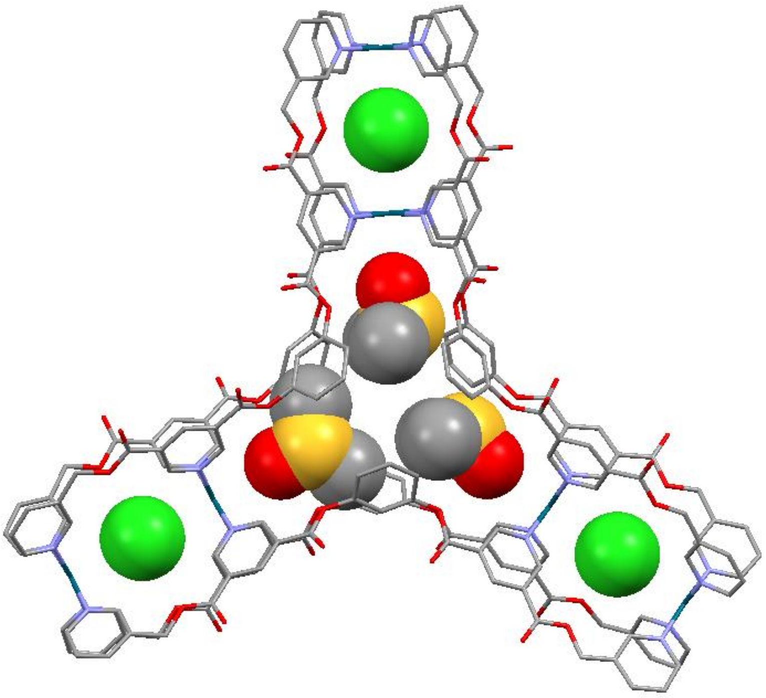

Supplementary Figure 143. Crystal structure showing three chlorides as the guest one each in the three smaller cavities and four DMSO molecules in the bigger cavity of the cage **6c**. (hydrogen atoms and exohedrally located counter-anions/solvents are excluded for clarity).

Supplementary References

1. S. Bandi, A. K. Pal, G. S. Hanan and D. K. Chand., *Chem. Eur. J.* **2014**, *20*, 13122-13126.
2. *Gaussian 09*, Revision A.02; Gaussian, Inc.: Wallingford, CT, 2009.
3. G. M. Sheldrick, *Acta Cryst. Sec. C.*, 2015, **71**, 3-8.
4. L. J. Farrugia, *J. Appl. Cryst.*, 2012, **45**, 849-854.
5. C. F. Macrae, P. R. Edgington, P. McCabe, E. Pidcock, G. P. Shields, R. Taylor, M. Towler, J. van de Streek, *J. Appl. Cryst*., 2006, **39**, 453-457.
6. A. L. Spek, *Acta Crystr*. *Sec. D*, 2009, **65**, 148-155.
